# An ATG12‐ATG5‐TECPR1 E3‐like complex regulates unconventional LC3 lipidation at damaged lysosomes

**DOI:** 10.15252/embr.202356841

**Published:** 2023-06-29

**Authors:** Dale P Corkery, Sergio Castro‐Gonzalez, Anastasia Knyazeva, Laura K Herzog, Yao‐Wen Wu

**Affiliations:** ^1^ Department of Chemistry Umeå University Umeå Sweden; ^2^ Umeå Centre for Microbial Research Umeå University Umeå Sweden

**Keywords:** autophagy, lysophagy, lysosome, membrane repair, TECPR1, Autophagy & Cell Death, Organelles, Post-translational Modifications & Proteolysis

## Abstract

Lysosomal membrane damage represents a threat to cell viability. As such, cells have evolved sophisticated mechanisms to maintain lysosomal integrity. Small membrane lesions are detected and repaired by the endosomal sorting complex required for transport (ESCRT) machinery while more extensively damaged lysosomes are cleared by a galectin‐dependent selective macroautophagic pathway (lysophagy). In this study, we identify a novel role for the autophagosome‐lysosome tethering factor, TECPR1, in lysosomal membrane repair. Lysosomal damage promotes TECPR1 recruitment to damaged membranes via its N‐terminal dysferlin domain. This recruitment occurs upstream of galectin and precedes the induction of lysophagy. At the damaged membrane, TECPR1 forms an alternative E3‐like conjugation complex with the ATG12‐ATG5 conjugate to regulate ATG16L1‐independent unconventional LC3 lipidation. Abolishment of LC3 lipidation via ATG16L1/TECPR1 double knockout impairs lysosomal recovery following damage.

## Graphical abstract

TECPR1 is recruited to damaged lysosomal membranes where it assembles into an alternative E3‐like complex with the ATG5‐ATG12 conjugate to regulate ATG16L1‐independent LC3 lipidation and promote lysosome repair.

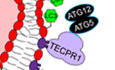

## Introduction

Lysosomes are membrane‐bound acidic organelles that play an essential role in the degradation of macromolecules received from autophagic, endocytic, or phagocytic pathways. Degradation is mediated by a family of lysosomal hydrolases capable of inducing acute cell death if inadvertently released into the cytosol (Kroemer & Jäättelä, 2005). To protect against the deleterious effects of lysosomal membrane permeabilization (LMP), cells employ sophisticated response mechanisms to detect, repair, remove, and replace damaged lysosomes.

Detection of damaged lysosomes is mediated by Ca^2+^ release and by rapid changes to the lipid profile of damaged membranes. With an approximately 5,000‐fold higher concentration of Ca^2+^ (0.6 mM), as compared to the cytosol (50–150 nM; Christensen *et al*, 2002), lysosomal ruptures cause a localized increase in cytosolic Ca^2+^, which triggers recruitment of the Endosomal Sorting Complex Required for Transport (ESCRT) membrane repair machinery (Jimenez *et al*, 2014; Scheffer *et al*, 2014; Radulovic *et al*, 2018; Skowyra *et al*, 2018). In addition, recent reports have identified a localized increase in phosphatidylinositol‐4‐phosphate (PtdIns(4)P), phosphatidylserine (PS), cholesterol (Radulovic *et al*, 2022; Tan & Finkel, 2022), and sphingomyelin (Niekamp *et al*, 2022) on the membranes of damaged lysosomes. Rapid changes in lipid composition regulate membrane contact site (MCS) formation and lipid‐dependent repair pathways.

If membrane damage is too extensive to be repaired, damaged lysosomes are sequestered and degraded by a selective macroautophagic pathway termed lysophagy. During macroautophagy (autophagy hereafter), two ubiquitin‐like ATG conjugation systems composed of core autophagy‐related (ATG) genes (ATG3, ATG5, ATG7, ATG10, ATG12, and ATG16L1) control the conjugation of LC3/GABARAP proteins (mammalian ATG8 proteins) to phosphatidylethanolamine (PE) on double‐membraned autophagosomes (Klionsky *et al*, 2021). Lysophagy is initiated by the recruitment of a family of β‐galactoside‐binding lectins (galectins) to intraluminal glycans exposed by membrane rupture (Paz *et al*, 2010; Thurston *et al*, 2012). Galectins serve as a platform to recruit core autophagy regulators to orchestrate the localized autophagic sequestration of damaged lysosomes (Thurston *et al*, 2012; Chauhan *et al*, 2016). Briefly, galectins recruit ubiquitin ligases leading to the extensive modification of lysosomal proteins with lysine 63 (K63)‐ and K48‐linked ubiquitin chains (Fujita *et al*, 2013; Papadopoulos *et al*, 2017). Ubiquitinated proteins are bound by autophagic receptors (Maejima *et al*, 2013), which, themselves, bind lipidated LC3 at the expanding autophagosomal membrane, leading to engulfment of the damaged lysosome into double‐membraned autophagosomes. Autophagosome biogenesis is coupled to membrane damage via Ub‐dependent recruitment of the autophagosome initiation complex (ULK1/ATG13/FIP200/ATG101) to the damaged membrane (Fujita *et al*, 2013). Recruitment of the E3‐like conjugation complex (ATG12‐ATG5‐ATG16L1) occurs via direct interactions between ATG16L1 and FIP200 and/or ATG16L1 and ubiquitin (Fujita *et al*, 2013) or directly via Galectin‐3 (Gal3) recruitment (Chauhan *et al*, 2016; Jia *et al*, 2020).

In addition to its role in lysophagy, the E3‐like conjugation complex has been shown to regulate the unconventional LC3 lipidation onto damaged lysosomal membranes, independent of the autophagy machinery upstream of the ATG conjugation system (Nakamura *et al*, 2020). This parallel LC3‐dependent pathway regulates calcium efflux essential for the induction of TFEB‐dependent lysosome biogenesis. Thus, the autophagic machinery is implicated in both the removal and replacement of damaged lysosomes.

Here, we identify Tectonin beta‐propeller repeat‐containing protein 1 (TECPR1) as a novel player in the cellular response to lysosomal membrane damage. TECPR1 is a lysosomal protein that has been implicated in autophagosome‐lysosome fusion via interaction with the ATG12‐ATG5 conjugate located on autophagosomal membranes (Ogawa *et al*, 2011; Chen *et al*, 2012; Wetzel *et al*, 2020). We report a sphingomyelin‐dependent enrichment of TECPR1 at lysosomes in response to lysosomal membrane damage. Enrichment occurs upstream of the galectin‐dependent lysophagy pathway and recruits the ATG12‐ATG5 conjugate to regulate ATG16L1‐independent LC3 lipidation at the damaged membrane. Abolishment of LC3 lipidation via ATG16L1/TECPR1 double knockout impairs the restoration of lysosomal function following damage. These observations identify a novel function of TECPR1 as a member of an alternative E3‐like conjugation complex functioning in the cellular response to LMP.

## Results and Discussion

### TECPR1 is recruited to lysosomes in response to membrane damage

**Figure 1:**
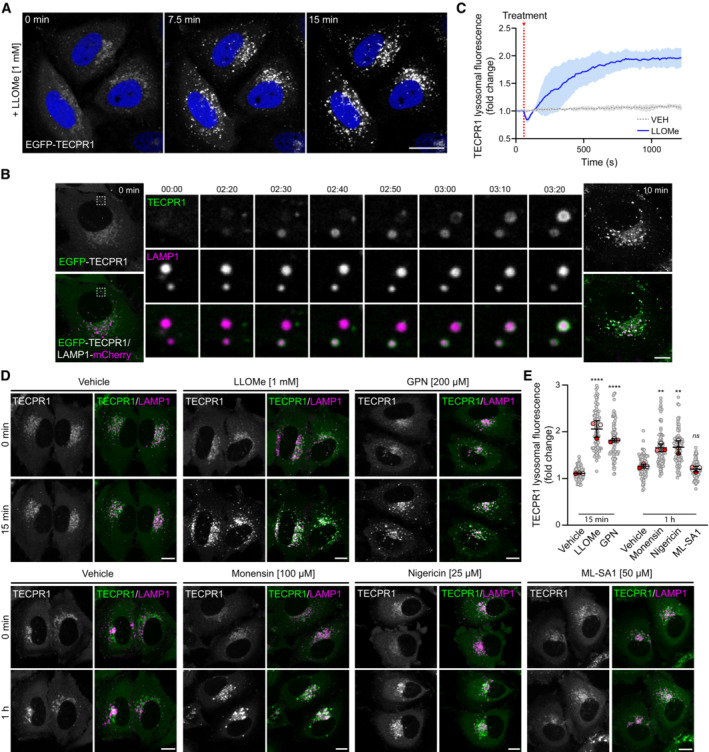
TECPR1 is recruited to lysosomes in response to membrane damage Representative live‐cell fluorescent images of HeLa cells transfected with EGFP‐TECPR1 and treated with 1 mM LLOMe for the indicated time. Nuclei were stained with Hoechst 33342. Scale bar = 20 μm.Representative live‐cell fluorescent images of HeLa cells co‐transfected with EGFP‐TECPR1 and LAMP1‐mCherry and treated with 1 mM LLOMe for the indicated time. Scale bar = 10 μM.Quantification of EGFP‐TECPR1 fluorescence intensity at the lysosome following treatment with LLOMe. Data are shown as mean ± SD from three independent experiments (*n* ≥ 8 cells per replicate). Images were captured every 10 s.Representative live‐cell fluorescent images of HeLa cells co‐transfected with EGFP‐TECPR1 and LAMP1‐mCherry and treated as indicated. Scale bars = 10 μM.Quantification of the fold change in EGFP‐TECPR1 lysosomal fluorescence from (D). Gray points represent individual cells from three independent experiments. Red points represent the means of individual experiments (*n* > 25 cells per experiment). Bars represent the mean ± SD from the three experiments. Significance was determined from biological replicates using a one‐way ANOVA with Tukey's multiple comparisons tests. **P < 0.005, ****P < 0.0001, *ns* = not significant. Source data are available online for this figure.

TECPR1 is a PtdIns(4)P‐binding lysosomal protein implicated in autophagosome‐lysosome fusion (Chen *et al*, 2012; Wetzel *et al*, 2020). Recent evidence suggests that PtdIns(4)P enrichment following LMP plays an essential role in mediating membrane repair (Radulovic *et al*, 2022; Tan & Finkel, 2022). To determine whether TECPR1 responds to LMP, HeLa cells were transiently transfected with EGFP‐TECPR1 and treated with the lysosomal membrane‐damaging agent L‐leucyl‐L‐leucine O‐methyl ester (LLOMe) (Thiele & Lipsky, 1990). Within minutes of LLOMe treatment, we observed a dramatic change in TECPR1 cellular localization (Fig 1A). Co‐transfection with lysosomal marker LAMP1 confirmed the change in localization was due to the rapid enrichment of TECPR1 at the lysosome following membrane damage (Fig 1B and C). To explore the specificity of TECPR1 recruitment, we assessed its lysosomal enrichment in response to a variety of lysosomal stressors (Fig 1D and E). The most robust and rapid recruitment was observed with LMP induced by treatment with either LLOMe or, Glycyl‐L‐phenylalanine‐beta‐naphthylamide (GPN) (Berg *et al*, 1994). Treatment with monensin or nigericin, carboxylic ionophores that alter lysosomal ionic balance to promote swelling and LC3 lipidation (Jacquin *et al*, 2017), induced TECPR1 lysosomal enrichment but required extended treatment times as compared to LLOMe or GPN. Treatment with ML‐SA1, an agonist of the lysosomal calcium channel TRPML1, failed to induce TECPR1 enrichment suggesting Ca^2+^ release alone is not sufficient to promote recruitment.

### TECPR1 lysosomal recruitment is dependent on its N‐terminal dysferlin domain

**Figure 2:**
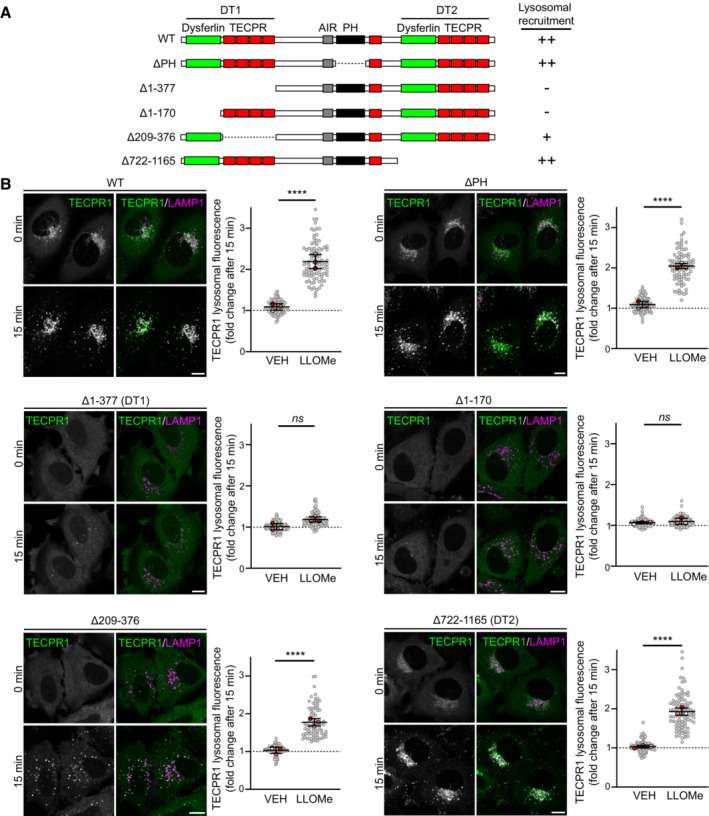
TECPR1 lysosomal recruitment is dependent on its N‐terminal dysferlin domain Domain architecture of wild‐type TECPR1 and deletion mutants.Representative live‐cell fluorescent images of HeLa cells co‐transfected with the indicated EGFP‐TECPR1 deletion mutant and LAMP1‐mCherry before and after treatment with 1 mM LLOMe. Scale bars = 10 μM. To the right is the corresponding quantification of the fold change in EGFP‐TECPR1 lysosomal fluorescence intensity after a 15‐min treatment with vehicle or 1 mM LLOMe. Gray points represent individual cells from three independent experiments. Red points represent the means of individual experiments (*n* > 25 cells per experiment). Bars represent the mean ± SD from the three experiments. Fold change value of 1 is indicated by a dotted line. Significance was determined from biological replicates using a one‐way ANOVA with Tukey's multiple comparisons tests. ****P < 0.0001, *ns* = not significant. Source data are available online for this figure.

To determine the region of TECPR1 responsible for lysosomal recruitment, we generated a series of TECPR1 deletion mutants and assessed their ability to translocate to the lysosome in response to LLOMe‐induced membrane damage (Fig 2). TECPR1 contains a phosphoinositide‐binding pleckstrin homology (PH) domain shown previously to bind PtdIns(3)P (Chen *et al*, 2012) and PtdIns(4)P (Wetzel *et al*, 2020). Deletion of the PH domain (TECPR1^ΔPH^) did not prevent TECPR1 lysosomal translocation after damage (Fig 2B) suggesting LMP‐dependent PtdIns(4)P enrichment (Tan & Finkel, 2022) is not responsible for TECPR1 recruitment.

Both the N‐ and C‐terminal regions of TECPR1 contain a dysferlin domain followed by four β‐propeller repeats (TECPR) (Fig 2A). Deletion of the N‐terminal dysferlin‐TECPR (DT1) domain (TECPR1^Δ1–377^) abolished lysosomal translocation, while deletion of the C‐terminal DT2 domain (TECPR1^Δ722–1165^) had no effect (Fig 2B). To further narrow down the region responsible for TECPR1 translocation we generated two additional mutants, one lacking the dysferlin domain of DT1 (TECPR1^Δ1–170^) and one lacking the β‐propeller repeats (TECPR1^Δ209–376^). Deletion of the β‐propeller repeats disrupted the lysosomal localization of TECPR1 in untreated cells but did not prevent lysosomal enrichment in response to damage (Fig 2B). By contrast, deletion of the dysferlin domain (TECPR1^Δ1–170^) both disrupted lysosomal localization and prevented damage‐induced enrichment. Thus, we can conclude that the N‐terminal dysferlin domain of TECPR1 is essential for its lysosomal recruitment in response to damage.

**Figure EV1:**
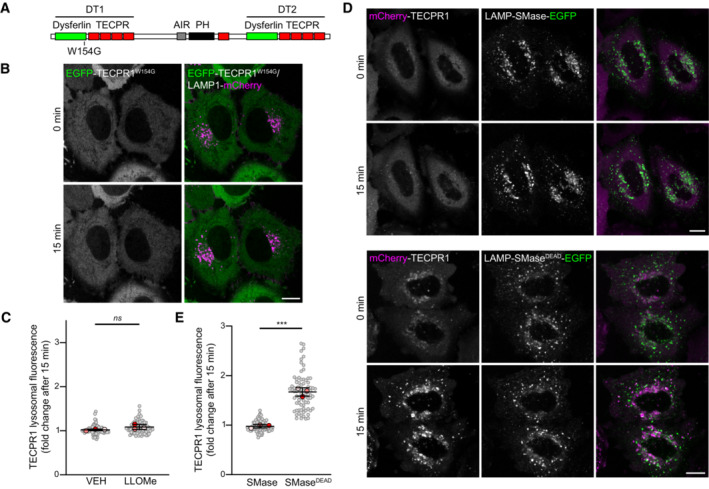
A sphingomyelin binding domain in the N‐terminal dysferlin domain of TECPR1 mediates lysosomal recruitment Location of the W154G mutation in TECPR1.Representative live‐cell fluorescent images of HeLa cells co‐transfected with EGFP‐TECPR1^W154G^ and LAMP1‐mCherry before and after treatment with 1 mM LLOMe. Scale bar = 10 μM.Quantification of the fold change in EGFP‐TECPR1 lysosomal fluorescence intensity after a 15‐min treatment with vehicle or 1 mM LLOMe. Gray points represent individual cells from three independent experiments. Red points represent the means of individual experiments (*n* > 25 cells per experiment). Bars represent the mean ± SD from the three experiments. Significance was determined from biological replicates using Student's *t*‐tests. *ns* = not significant.Representative live‐cell fluorescent images of HeLa cells co‐transfected with mCherry‐TECPR1 and LAMP1‐SMase‐EGFP (top) or LAMP‐SMase^DEAD^‐EGFP (bottom) before and after treatment with 1 mM LLOMe. Scale bar = 10 μM.Quantification of the fold change in mCherry‐TECPR1 lysosomal fluorescence intensity after a 15‐min treatment with vehicle or 1 mM LLOMe (from D). Gray points represent individual cells from three independent experiments. Red points represent the means of individual experiments (*n* > 25 cells per experiment). Bars represent the mean ± SD from the three experiments. Significance was determined from biological replicates using Student's *t*‐tests. *** = 0.0002.

In a companion manuscript, Boyle *et al* (2023) observed TECPR1 recruitment to damaged phagosomal membranes in response to bacterial infection. This recruitment is mediated by a sphingomyelin binding domain identified in the N‐terminal dysferlin domain of TECPR1. Lysosomal damage was recently shown to induce a rapid sphingomyelin translocation from the luminal to cytoplasmic membrane surface (Niekamp *et al*, 2022), suggesting sphingomyelin enrichment at damaged membranes could represent a conserved TECPR1 recruitment mechanism. To confirm, we introduced a point mutation at W154 of TECPR1, shown by Boyle *et al* (2023) to be essential for sphingomyelin binding. This single point mutation was sufficient to block TECPR1 lysosomal enrichment in response to LLOMe treatment (Fig EV1A–C). Furthermore, the ectopic expression of sphingomyelinase (SMase) from *Bacillus cereus* targeted to the cytosolic surface of the lysosome (Niekamp *et al*, 2022) was sufficient to inhibit LMP‐dependent TECPR1 recruitment (Fig EV1D and E). These data confirm sphingomyelin‐dependent TECPR1 recruitment to damaged lysosomal membranes.

### Recruitment of TECPR1 precedes intraluminal glycan exposure

**Figure 3:**
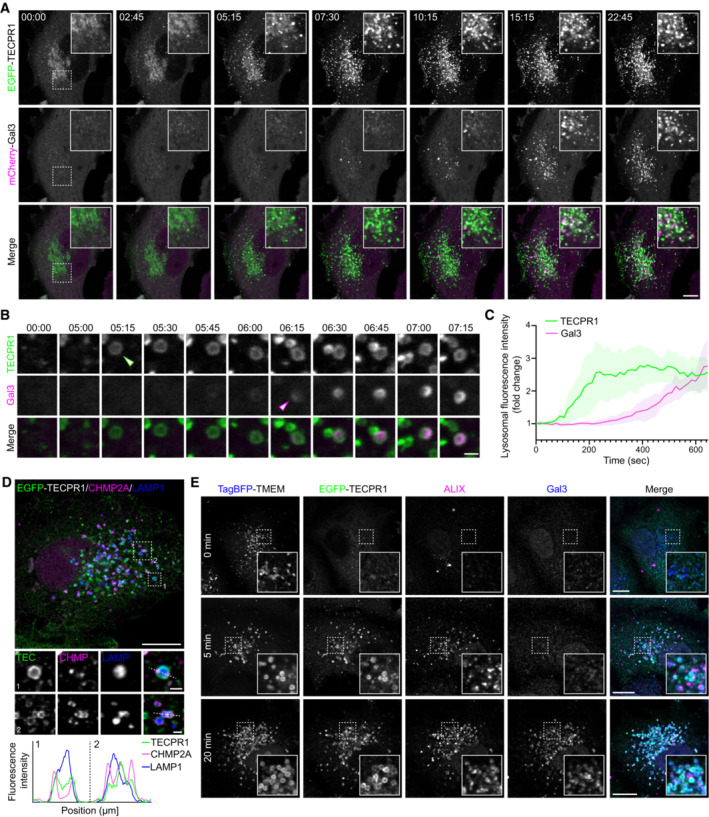
TECPR1 recruitment to damaged lysosomes precedes galectin recruitment A, BRepresentative live‐cell fluorescent images of HeLa cells co‐transfected with EGFP‐TECPR1 and mCherry‐Gal3 and treated with 1 mM LLOMe for the indicated time. Arrowheads in (B) indicate the appearance of TECPR1 (green arrow) and Gal3 (magenta arrow). Scale bars = 10 μM for (A) and 1 μM for (B).CQuantification of TECPR1 and Gal3 recruitment to damaged lysosomes. Data are presented as mean ± SD from five independent experiments (each experiment represents a single cell with at least three individual lysosomes quantified).DRepresentative confocal image of a HeLa cell transfected with EGFP‐TECPR1, treated with LLOMe for 5 min, and immunostained for CHMP2A and LAMP1. Scale bars = 10 μm for whole image and 1 μm for insets. Fluorescence intensity profiles of the indicated channels across the dotted lines are shown in the lower subpanel.ERepresentative confocal images of a HeLa cells transfected with EGFP‐TECPR1 and TagBFP‐TMEM192 (a lysosomal/late endosomal protein), treated with 1 mM LLOMe for the indicated time, and immunostained for ALIX and Gal3. Scale bars = 10 μm. Source data are available online for this figure.

Two independent pathways regulate the repair or removal of damaged lysosomal membranes. Small disruptions are rapidly detected and repaired by the ESCRT machinery (Skowyra *et al*, 2018), while more extensive damage induces an autophagy‐dependent pathway for the removal of damaged membranes (Thurston *et al*, 2012; Maejima *et al*, 2013; Chauhan *et al*, 2016). The latter is initiated by the recruitment of Galectin‐family carbohydrate‐binding proteins to exposed intraluminal glycans on extensively damaged lysosomes. There, they act as a signaling platform to induce autophagic degradation of the damaged membrane. To determine the timing of TECPR1 recruitment, live‐cell imaging was performed in cells co‐transfected with EGFP‐TECPR1 and mCherry‐Gal3. TECPR1 enrichment was observed within minutes of LLOMe addition and appears to plateau after 10 min of exposure (Figs 1C and 3A). In agreement with previously published reports (Radulovic *et al*, 2018), we observed slower recruitment kinetics for Gal3 with enrichment beginning after 10 min LLOMe exposure (Fig 3A). Furthermore, high‐resolution imaging of individual lysosomes revealed that TECPR1 and Gal3 target the same lysosome, but TECPR1 recruitment occurs several minutes prior to Gal3 (Fig 3B and C). This suggests that TECPR1 recruitment occurs in response to small ruptures, independent of the galectin‐regulated autophagy pathway.

We next investigated the relationship between ESCRT machinery and TECPR1 recruitment. Lysosomes damaged after 5 min of LLOMe exposure were enriched for both EGFP‐TECPR1 and the ESCRT‐III complex member CHMP2A (Fig 3D). Interestingly, TECRP1 appears uniformly distributed on the lysosomal membrane while CHMP2A enrichment is restricted to more defined subdomains (Fig 3D). Co‐staining for Gal3 and ESCRT‐III binding protein ALIX confirmed that TECPR1 and the ESCRT machinery are both recruited within 5 min of LLOMe treatment, prior to Gal3 recruitment. At 20 min post‐LLOMe treatment TECPR1, ALIX, and Gal3 are all enriched at lysosomes (Fig 3E). Collectively, these data suggest that TECPR1 recruitment represents an early event in the lysosomal membrane repair pathway.

### TECPR1 recruits the ATG5‐ATG12 complex to catalyze ATG16L1‐independent LC3 lipidation onto damaged lysosomal membranes

**Figure 4:**
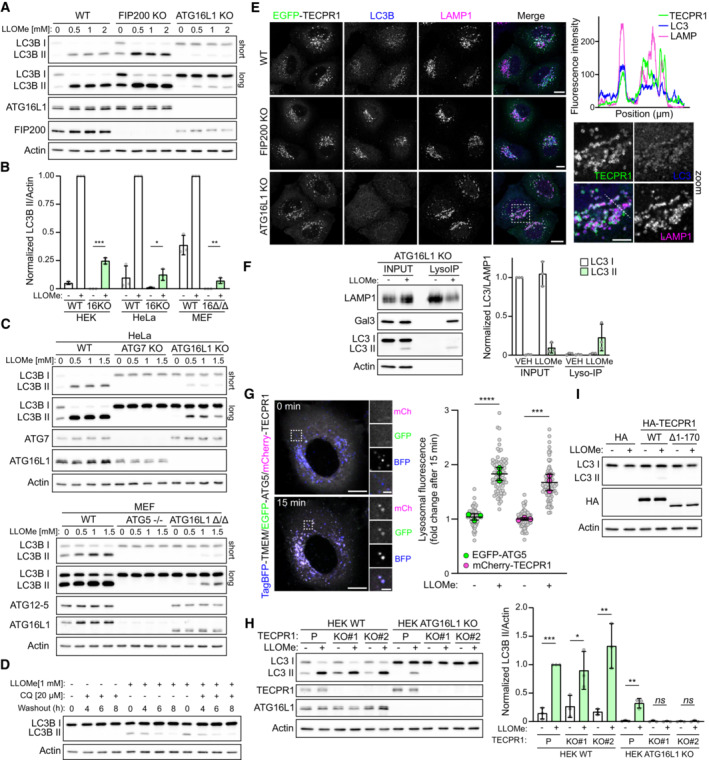
TECPR1 recruits the ATG5‐ATG12 complex to damaged lysosomes to catalyze LC3 lipidation onto damaged membranes Western blot analysis of LC3 lipidation status in wild‐type (WT), FIP200 KO, and ATG16L1 KO HeLa cells treated with the indicated concentrations of LLOMe for 30 min.Quantification of LC3‐II levels in HEK, HeLa, and MEF WT and ATG16L1‐deficient cell lines treated with and without 1 mM LLOMe for 30 min. Bars show mean ± SD from three biologically independent experiments represented as data points. Significance was determined from biological replicates using Student's *t*‐tests. *P = 0.0177, **P = 0.0082, ***P = 0.0001. Corresponding Western blots are found in EV2B.Western blot analysis of LC3 lipidation status in WT, ATG7 KO, and ATG16L1 KO HeLa cells (top) and WT, ATG5^−/−^ and ATG16L1^Δ/Δ^ MEFs (bottom) treated with the indicated concentration of LLOMe for 30 min.Western blot analysis of LC3 lipidation status in ATG16L1 KO HeLa cells treated with 1 mM LLOMe for 30 min followed by washout in the presence or absence of 20 μM chloroquine.Representative confocal images of HeLa WT, FIP200 KO, and ATG16L1 KO cells transfected with EGFP‐TECPR1, treated with 1 mM LLOMe for 30 min and immunostained for LC3 and LAMP1. Scale bars = 10 μm for whole images and 5 μm for the zoom. Fluorescence intensity profiles for the indicated channels across the dotted line are shown in the upper right subpanel.Western blot analysis of lyso‐IP samples collected from HeLa ATG16KO cells treated with or without 1 mM LLOMe for 30 min. Bars show mean ± SD from three biologically independent experiments represented as data points.Representative live‐cell fluorescent images of HeLa cells co‐transfected with TagBFP‐TMEM192, EGFP‐ATG5, and mCherry‐TECPR1, before and after a 15‐min treatment with 1 mM LLOMe. Scale bars = 10 μm for whole images and 2 μm for zoom. To the right is the corresponding quantification of the fold change in ATG5/TECPR1 lysosomal fluorescence intensity after a 15‐min treatment with vehicle or 1 mM LLOMe. Gray points represent individual cells from three independent experiments. Green/magenta points represent the means of individual experiments (*n* > 25 cells per experiment). Bars represent the mean ± SD from the three experiments. Significance was determined from biological replicates using a one‐way ANOVA with Tukey's multiple comparisons tests. ****P < 0.0001, ***P = 0.0002.Western blot analysis of LC3 lipidation status in HEK TECPR1/ATG16L1 KO cells treated with 1 mM LLOMe for 30 min. To the right is the corresponding quantification of LC3‐II protein levels. Bars show mean ± SD from three or four biologically independent experiments, which are represented as data points. Significance was determined from biological replicates using a one‐way ANOVA with Tukey's multiple comparisons tests. ***P = 0.0001, **P < 0.01, *P = 0.0467.Western blot analysis of LC3 lipidation status in HEK TECPR1/ATG16L1 double‐KO cells transfected with the indicated plasmid and treated with 1 mM LLOMe 30 min. Data are representative of three independent experiments. Source data are available online for this figure.

**Figure EV2:**
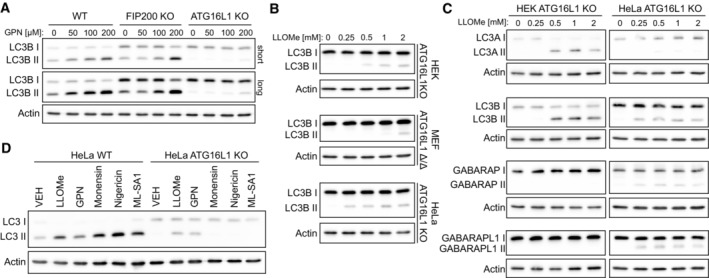
ATG8 lipidation in ATG16L1‐deficient cell lines Western blot analysis of LC3 lipidation status in wild‐type (WT), FIP200 KO, and ATG16L1 KO HeLa cells treated with the indicated concentrations of GPN for 30 min.Western blot analysis of LC3B lipidation status in HEK, MEF, and HeLa ATG16L1‐deficient cell lines treated with the indicated concentration of LLOMe for 30 min.Western blot analysis of ATG8 lipidation status in HEK and HeLa ATG16L1‐deficient cell lines treated with the indicated concentration of LLOMe for 30 min.Western blot analysis of LC3 lipidation status in HeLa WT and ATG16L1 KO cells treated as indicated.

TECPR1 forms a complex with the ATG12‐ATG5 conjugate (Behrends *et al*, 2010) and has been implicated in autophagosome maturation by promoting autophagosome‐lysosome fusion (Chen *et al*, 2012; Wetzel *et al*, 2020). In the context of lysosomal damage, autophagy plays an important role in both the sequestration of damaged membranes (Maejima *et al*, 2013) and the TFEB‐dependent induction of lysosome biogenesis (Nakamura *et al*, 2020). To determine whether TECPR1 was playing a role in the autophagic response to lysosomal damage, we first assessed LC3B lipidation status in a series of autophagy‐deficient cell lines treated with lysosomal damaging agents. In agreement with previous findings (Nakamura *et al*, 2020), lysosome damage‐induced LC3B lipidation in both wild‐type (WT), and basal autophagy‐deficient FIP200 KO HeLa cell lines (Figs 4A and EV2A). Surprisingly, we observed residual LC3B lipidation in HeLa cells deficient for the E3‐like complex member, ATG16L1 (Figs 4A and EV2A). ATG16L1 is a membrane‐binding protein that functions as a scaffold for LC3 lipidation by recruiting the ATG12‐ATG5 conjugate to target membranes (Fujita *et al*, 2008; Lystad *et al*, 2019; Pantoom *et al*, 2021). As ATG16L1 is considered essential for LC3 lipidation, we validated the observed ATG16L1‐independent LC3B lipidation phenotype in three ATG16L1‐deficient cell lines (Figs 4B and EV2B). HeLa ATG16L1 KO, HEK ATG16L1 KO, and ATG16L1‐deficient (Δ/Δ) MEF cells all displayed residual LC3B lipidation after LLOMe treatment, which ranged from 7 to 25% that of the matched WT cell line (Figs 4B and EV2B). While this lipidation is independent of ATG16L1, it is dependent on other components of the ATG conjugation systems as knockout of ATG5 or ATG7 completely blocked LC3‐II accumulation in LLOMe‐treated cells (Fig 4C). Interestingly, ATG16L1‐independent lipidation is not specific to LC3B as we observed cell line‐dependent lipidation of LC3A, GABARAP, and GABARAPL1 in ATG16L1‐deficient HeLa/HEK cells treated with LLOMe (Fig EV2C). Of all lysosomal stressors tested (Fig 1D), ATG16L1‐independent LC3 lipidation was observed only in cells treated with LLOMe and GPN (Fig EV2D) suggesting lipidation is coupled to LMP.

To gain insight into the function of the lipidated LC3, cells were treated with LLOMe for 30 min to induce lipidation followed by washout of the compound in the presence and absence of chloroquine to inhibit lysosomal function. The lipidated LC3 was resolved within 8 h of LLOMe washout, even in the presence of chloroquine, suggesting a nondegradative function for this lipidated pool of LC3 (Fig 4D). To identify the localization of lipidated LC3B, HeLa WT, FIP200‐, and ATG16L1 KO cells were transfected with EGFP‐TECPR1, treated with LLOMe, and immunostained for LC3B and LAMP1 (Fig 4E). LC3B accumulation at TECPR1‐enriched lysosomes was apparent in ATG16L1 KO cells, although significantly reduced in comparison to WT and FIP200 KO cells, consistent with the LC3B western blot data (Fig 4B). Furthermore, lysosomal immunoprecipitation (Lyso‐IP) performed in ATG16L1 KO cells confirmed the accumulation of lipidated LC3 (LC3‐II) on membranes damaged by LLOMe (Fig 4F).

**Figure EV3:**
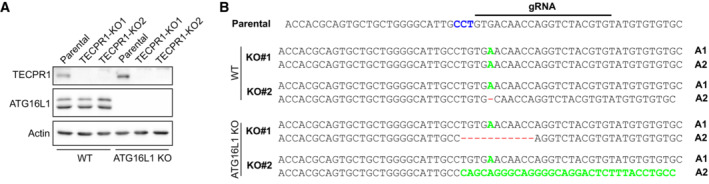
Validation of HEK TECPR1 KO cell lines Western blot analysis of TECPR1 and ATG16L1 protein levels in CRISPR‐Cas9 clones.Sequencing results from genomic PCR of TECPR1 exon 3 from CRISPR‐Cas9 clones. Green represents insertions and red dashes represent deletions.

**Figure EV4:**
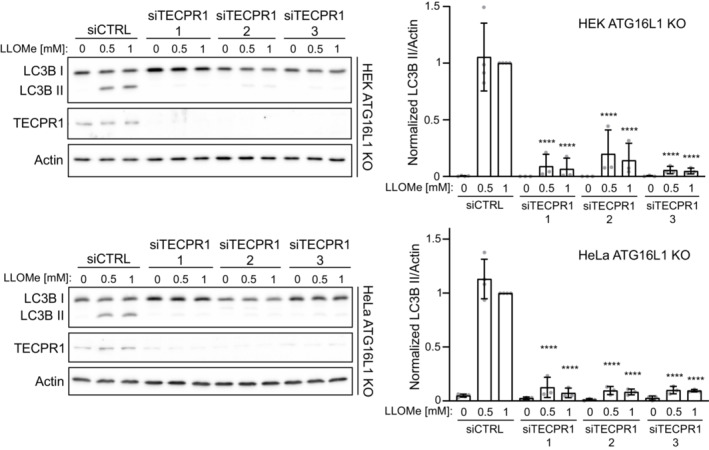
siRNA‐mediated TECPR1 knockdown prevents ATG16L1‐independent LC3 lipidation Western blot analysis of LC3 lipidation status in HEK (top) and HeLa (bottom) ATG16 KO cells transfected with TECPR1 siRNAs for 72 h and treated with the indicated concentration of LLOMe for 30 min. To the right is the corresponding quantification of LC3‐II protein levels. Bars show mean ± SD from three or four biologically independent experiments, which are represented as data points. Significance was determined from biological replicates using a one‐way ANOVA with Tukey's multiple comparisons tests. ****P < 0.0001.

ATG16L1 and TECPR1 form mutually exclusive complexes with the ATG12‐ATG5 conjugate (Chen *et al*, 2012), suggesting that ATG12‐ATG5‐TECPR1 could serve as an E3‐like complex in response to lysosomal membrane damage. Co‐transfection of EGFP‐ATG5 and mCherry‐TECPR1 confirmed TECPR1's ability to recruit ATG5 to lysosomes damaged by LLOMe (Fig 4G). To confirm TECPR1 was responsible for ATG16L1‐independent LC3 lipidation we used CRISPR‐Cas9 to generate HEK TECPR1 KO and HEK ATG16L1/TECPR1 double‐KO cell lines (Fig EV3A and B). The residual LC3 lipidation observed in ATG16L1 KO cells treated with LLOMe is abolished with knockout, or siRNA‐mediated knockdown, of TECPR1 (Figs 4H and EV4). In the presence of ATG16L1, TECPR1 knockout did not reduce LMP‐induced LC3 lipidation as would be expected if TECPR1‐dependent lipidation represented a distinct pool of LC3‐II. However, given that loss of TECPR1 has been shown to impair autophagosome‐lysosome fusion (Chen *et al*, 2012), we cannot exclude the possibility that loss of TECPR1‐dependent LC3 lipidation is masked by elevated ATG16L1‐dependent LC3 lipidation as a consequence of impaired autophagy flux. To further confirm the recruitment of TECPR1 as the stimulus for ATG16L1‐independent LC3 lipidation, we transfected ATG16L1/TECPR1 double‐KO cells with TECPR1^WT^ or TECPR1^Δ1–170^ lacking the N‐terminal dysferlin domain required for recruitment in response to LMP (Fig 2). TECPR1^WT^ restored LMP‐induced LC3 lipidation while TECPR1^Δ1–170^ did not (Fig 4I) confirming TECPR1 acts in complex with ATG5‐ATG12 to catalyze the conjugation of lipidated LC3 onto damaged lysosomal membranes following LMP.

Identification of an alternative ATG12‐ATG5‐TECPR1 complex that functions as the E3‐like enzyme in membrane damage‐induced LC3 lipidation challenges the paradigm that ATG16L1 is essential for ATG12‐ATG5 targeting and subsequent LC3 lipidation in autophagy (Mizushima *et al*, 2003; Fujita *et al*, 2008). TECPR1 and ATG16L1 have been shown to exist in two mutually exclusive complexes with ATG12‐ATG5 (Chen *et al*, 2012). Our data identify a minor fraction (7–25%) of total LC3 lipidation that is regulated by ATG12‐ATG5‐TECPR1 (Fig 4B), suggesting that ATG12‐ATG5‐ATG16L1 remains the main contributor to LC3 lipidation on damaged lysosomes. Future studies determining how TECPR1 association with damaged membranes specifically promotes assembly of the alternative E3‐like complex will be important in understanding potential functions within and beyond the membrane damage response.

### TECPR1/ATG16L1 are required for efficient lysosomal recovery after damage

**Figure 5:**
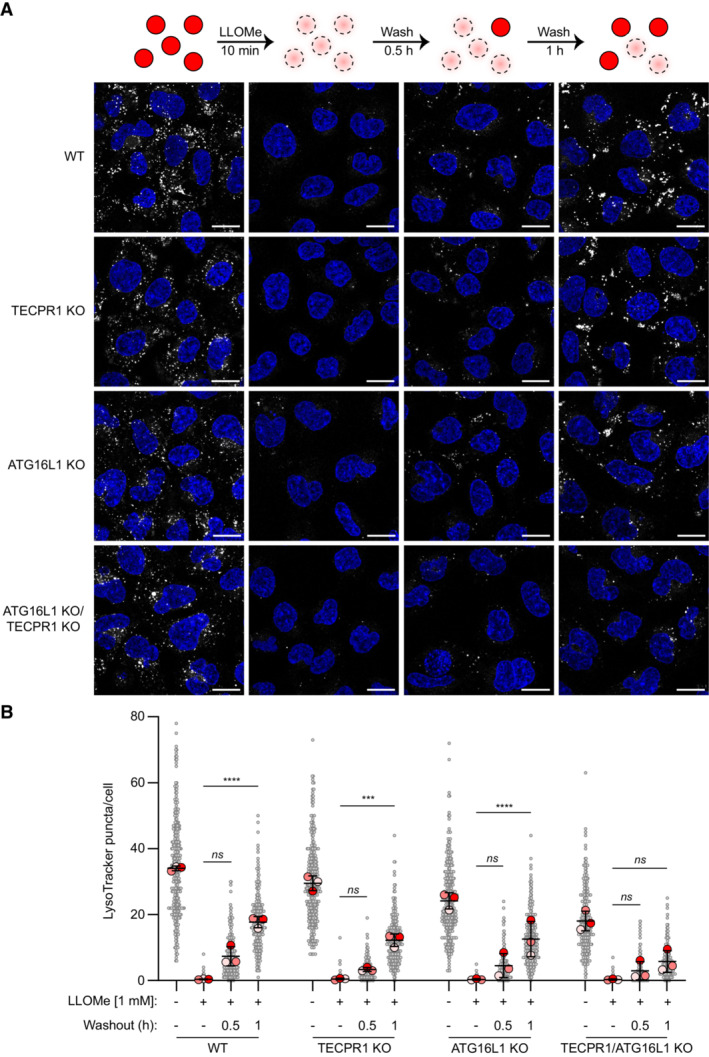
TECPR1/ATG16L1 are required for efficient lysosomal recovery after damage Representative live‐cell images of HEK cells treated as indicated and stained with LysoTracker Red. Nuclei were stained with Hoechst 33342. Scale bars = 20 μm.Quantification of LysoTracker Red puncta from (A). Gray points represent individual cells from three independent experiments. Red points represent the means of individual experiments (*n* > 100 cells per experiment). Bars represent the mean ± SD from the three experiments. Significance was determined from biological replicates using a one‐way ANOVA with Tukey's multiple comparisons tests. ****P < 0.0001, ***P = 0.0002, *ns* = not significant. Source data are available online for this figure.

To determine whether TECPR1 plays a role in resolving lysosomal membrane damage, HEK WT, TECPR1 KO, ATG16L1 KO, and TECPR1/ATG16L1 double‐KO cells were labeled with LysoTracker Red, pulsed with LLOMe for 10 min and allowed to recover for 30 or 60 min in the presence of LysoTracker. Restoration of lysosomal function was assessed by the recovery of Lysotracker staining (Fig 5A and B). Single knockout of either ATG16L1 or TECPR1 did not significantly impair lysosomal recovery. The knockout of both led to a reduction in basal LysoTracker staining and a significant impairment in lysosomal recovery following LMP. These data confirm the requirement for lipidated LC3 in lysosomal recovery from LMP and suggests a certain degree of functional redundancy between ATG16L1 and TECPR1 E3‐like complexes.

Activation of the ESCRT‐dependent membrane repair pathway in response to lysosomal damage is well established (Bohannon & Hanson, 2020). What remains to be determined is the exact mechanism through which the ESCRT machinery repairs membrane perforations. Injuries in the nanometer range have the ability to spontaneously reseal, driven by lipid disorder at the curved edges of a disruption (Cooper & McNeil, 2015). Larger injuries require active membrane repair mechanisms aimed at reducing membrane tension around the site of injury to reduce the pore size and promote spontaneous repair. One hypothesis for ESCRT machinery function at the site of lysosomal injury is in the prevention of pore expansion via ESCRT‐III filament assembly around the injury site (Bohannon & Hanson, 2020). How lipidated LC3 contributes to membrane repair remains unknown, but *in vitro* studies have identified a role for yeast Atg8 in mediating membrane tethering and hemifusion in response to PE conjugation (Nakatogawa *et al*, 2007). In this study, PE conjugation was shown to promote its multimerization followed by membrane tethering and hemifusion. Conjugation of LC3 to membranes or using synthetic LC3‐PE has also been shown to mediate membrane tethering and fusion/hemifusion *in vitro* (Weidberg *et al*, 2011; Yang *et al*, 2013). Thus, we propose that lipidated LC3 at the site of injury could cooperate in the repair process by reducing membrane tension via multimerization and tethering of the pore. Alternatively, lipidated LC3 could serve to recruit and/or activate binding partners upon lysosomal damage. For example, lipidated LC3 interacts with the lysosomal calcium channel TRPML1, which facilitates calcium efflux essential for the activation of TFEB, a master regulator of lysosomal biogenesis (Nakamura *et al*, 2020). Upon lysosomal membrane damage induced by SARS‐CoV‐2 ORF3a, *Mycobacterium tuberculosis*, or proteopathic tau, lipidated ATG8s recruit stress granule proteins that contribute to mTOR inactivation (Jia *et al*, 2022). Future studies characterizing the function of the TECPR1‐regulated pool of lipidated LC3 will be important in furthering our understanding of noncanonical functions of the autophagy machinery.

## Materials and Methods

### Antibodies and reagents

Antibodies used in this study were from the following sources: LC3A (#4599, WB: 1:1,000), LC3B (#2775, WB: 1:1,000), GABARAP (#13733, WB: 1:1,000), GABARAPL1 (#26632, WB: 1:1,000), ATG16L1 (#8089, WB: 1:1,000), FIP200 (#12436, WB: 1:1,000), ATG7 (#8558, WB: 1:1,000), ATG12—mouse‐specific (#2011, WB: 1:1,000), TECPR1 (#8097, WB: 1:1,000), LAMP1 (#15665, IF: 1:100) and Gal3 (#87985, IF: 1:400) antibodies were purchased from Cell Signaling Technology. Anti‐beta‐actin antibody (A2228, WB: 1:10,000) was purchased from Sigma‐Aldrich. LC3 (PM036, IF: 1:500) antibody was purchased from MBL International. ALIX antibody (634502, IF: 1:200) was purchased from BioLegend. CHMP2A antibody (10477‐1‐AP, IF: 1:100) was purchased from Proteintech. EGFP antibody (Cat# A10262, IF: 1:500) and goat anti‐rabbit‐HRP (Cat# 31460, WB: 1:10,000), and goat anti‐mouse‐HRP (Cat# 31430, WB: 1:10,000) antibodies were purchased from Thermo Fisher. Alexa Fluor 488/568/647 conjugated secondary antibodies for immunofluorescence were purchased from Thermo Fisher.

Reagents used in this study were from the following sources: Leu‐Leu methyl ester hydrobromide (LLOMe, L7393), monensin sodium salt (M5273), ML‐SA1 (SML0627) and chloroquine (CQ, C6628) from Sigma‐Aldrich; Gly‐Phe beta‐naphthylamide (GPN, J64718.MC), Lysotracker Red DND‐99 (L7528) and Hoechst 33342 (H1399) from Thermo Fisher, Nigericin sodium salt (16485717) from Fisher Scientific.

### Cells and cell culture

HeLa cells (WT, FIP200 KO, ATG7 KO, ATG16L1 KO) were a kind gift from Tomatsu Yoshimori—Osaka University, Osaka, Japan (Nakamura *et al*, 2020). ATG5 KO MEFs were a kind gift from Noboru Mizushima –Tokyo Medical and Dental University, Tokyo, Japan (Kuma *et al*, 2004). ATG16 Δ/Δ MEFs were a kind gift from Shizuo Akira—Osaka University, Osaka, Japan (Saitoh *et al*, 2008). FIP200 KO MEFs were a kind gift from Jun‐Lin Guan, University of Cincinnati, USA (Gan *et al*, 2006). HEK293 (WT, ATG16KO) was a kind gift from Anne Simonsen—University of Oslo, Oslo, Norway (Lystad *et al*, 2019). All cells were cultured in Dulbecco's modified Eagle medium (DMEM) (Sigma‐Aldrich) supplemented with 10% fetal bovine serum (FBS), 1% penicillin/streptomycin, and nonessential amino acids at 37°C with 5% CO_2_. No cell line authentication was performed. Cells were routinely tested for mycoplasma contamination using the LookOut mycoplasma PCR detection kit (Sigma‐Aldrich).

### Plasmids

EGFP‐TECPR1 and EGFP‐TECPR1^ΔPH^ were a kind gift from Thomas Wollert—Institute Pasteur, Paris, France (Wetzel *et al*, 2020). All EGFP‐TECPR1 mutants were derived from EGFP‐TECRP1 using the following primers:

**Table:** 

Mutant	Primers
EGFP‐TECPR1^Δ1–377^	Fwd: GAGACTCGAGGCGCCCGAGAGTGTGACC
	Rev: CGACTGCAGAATTCGAAGC
EGFP‐TECPR1^Δ1–170^	Fwd: GAGACTCGAGGCTCCCGGGACATCTGGG
	Rev: CGACTGCAGAATTCGAAGC
EGFP‐TECPR1^Δ209–376^	Fwd: GGTAGAGCAGCTGCGGCCCGAGAGTGTGACC
	Rev: AGCTGCTCTACCCACAGGCTCCTCCGTGATCTC
EGFP‐TECPR1^Δ722–1165^	Fwd: GTCCGGACTCAGATCTCGAGGC
	Rev: GAGAAAGCTTTCACTTCCGGCTCTCGCAGC
EGFP‐TECPR1^W154G^	Fwd: CGAAAGACAAGAAGGGGAATTCTTGTGTGC
	Rev: GCACACAAGAATTCCCCTTCTTGTCTTTCG

LAMP1‐SMase‐EGFP and LAMP1‐SMase^DEAD^‐EGFP were a kind gift from Joost C. M. Holthuis—University of Osnabrück, Osnabrück, Germany (Niekamp *et al*, 2022). mCherry‐TECPR1 was generated by PCR amplifying TECPR1 from EGFP‐TECPR1 and subcloning into the mCherry‐C1 (Clontech) plasmid using Kpn2I/SalI restriction sites. HA‐TECPR1^WT^ and HA‐TECPR1^Δ1–170^ were generated by PCR amplifying TECPR1^WT/Δ1–170^ from EGFP‐TECPR1/EGFP‐TECPR1^Δ1–170^ inserting an HA tag in the forward primer. LAMP1‐mCherry was generated by PCR amplifying human LAMP1 from cDNA and subcloning into the mCherry‐N1 (Clontech) vector using NheI/XhoI restriction sites. mCherry‐Gal3 was generated by PCR amplifying Gal3 from EGFP‐Gal3 (gift from Tamotsu Yoshimori—Addgene plasmid #73080; http://n2t.net/addgene:73080; RRID:Addgene_73080) (Maejima *et al*, 2013) and subcloning into the mCherry‐C1 (Clontech) vector using BsrGI/BamHI restriction sites. TagBFP‐TMEM192 was generated by PCR amplifying TMEM192 from pLJC5‐Tmem192‐3xHA (a gift from David Sabatini—Addgene plasmid #102930; http://n2t.net/addgene:102930; RRID:Addgene_102930) (Abu‐Remaileh *et al*, 2017) and subcloning into the TagBFP‐C1 plasmid (Evrogen). EGFP‐ATG5 was generated by PCR amplifying human ATG5 from cDNA and subcloning into the EGFP‐C2 (Clontech) vector using BamHI/XhoI restriction sites. pSpCas9(BB)‐2A‐Puro (PX459) V2.0 was a gift from Feng Zhang (Addgene plasmid #62988; http://n2t.net/addgene:62988; RRID:Addgene_62988) (Ran *et al*, 2013). All plasmids were verified by Sanger sequencing.

### Transfection

Transfection of DNA constructs was performed using X‐tremeGENE HP transfection reagent (Sigma‐Aldrich, 6366236001) according to the manufacturer's directions. Transfection of siRNAs was performed using Lipofectamine RNAiMAX transfection reagent (Thermo Fisher, 13778150) according to the manufacturer's directions. Cells were incubated for 72‐h post‐transfection with siRNAs before being treated with LLOMe and harvested. siRNAs were purchased from Thermo Fisher (siCTRL: 4390843, siTECPR1‐1: ID#126821, siTECRP1‐2: ID#126822, siTECPR1‐3: ID#126823).

### Immunofluorescence and live‐cell imaging

Cells were grown on no. 1.5 glass coverslips and fixed in 4% paraformaldehyde for 10 min at room temperature. Cells were washed three times with PBS containing 1.5 mg/mL glycine, permeabilized in 0.25% Triton X‐100 for 5 min, and washed three times with PBS. Cells were blocked with 5% donkey serum for 30 min followed by a 1‐ to 2‐h incubation with primary antibody at room temperature. Cells were washed three times with PBS and incubated with Alexa Fluor conjugated secondary antibodies for 30 min at room temperature. Cells were washed three times with PBS and mounted on slides using ProLong Diamond antifade mountant (Thermo Fisher, P36970).

For live‐cell imaging, cells were seeded on μ‐Slide 8 well or glass bottom 35 mm dishes (Ibidi) and incubated for 24 h. Imaging was performed in DMEM without phenol red (Sigma‐Aldrich) and supplemented with 20 mM HEPES.

Imaging was performed on a Leica SP8 FALCON inverted confocal system (Leica Microsystems) equipped with a HC PL APO 63×/1.40 oil immersion lens and a temperature‐controlled hood maintained at 37°C and 5% CO_2_, for live‐cell imaging. The microscope was controlled by Leica Application Suite X (LASX). Hoechst 33342/TagBFP were excited using a 405 nm Diode laser, and EGFP/Alexa488, mCherry/Alexa568, and Alexa647 fluorescence were excited using a tuned white light laser. Scanning was performed in line‐by‐line sequential mode.

### Lysosomal immunoprecipitation

Lyso‐IP was carried out as described previously (Abu‐Remaileh *et al*, 2017) with a few modifications. Briefly, HeLa ATG16KO cells stably expressing TMEM192‐HA were seeded in 15 cm plates and treated with 1 mM LLOMe (or vehicle) for 30 min. Cells were harvested by scraping in PBS supplemented with 1× complete protease inhibitor (Roche). Cells were pelleted, re‐suspended in PBS supplemented with protease inhibitor, and lysed using 30 strokes of a Dounce tissue grinder. Lysosomes were immunoprecipitated using Pierce Anti‐HA magnetic beads (Thermo Fisher) according to the manufacturer's directions. Samples were eluted by boiling the beads with 2× Laemmeli buffer for 10 min.

### Generation of CRISPR KO cell lines

Oligonucleotides encoding a gRNA targeting exon 3 of TECPR1 (CACGTAGACCTGGTTGTCAC) were annealed and cloned into pSpCas9(BB)‐2A‐Puro (PX459) V2.0. HEK WT and ATG16L1 KO cells were transiently transfected and selected with puromycin for 48 h, and clonal cell lines were isolated by limiting dilution. TECPR1 KO cells were identified by immunoblotting and verified by genomic PCR amplification and sequencing of TECPR1 exon 3 (Fig EV3).

### LysoTracker repair assay

HEK WT/ATG16L1 KO/TECPR1 KO/ATG16L1 TECPR1 double‐KO cells were seeded in μ‐Slide 8 glass bottom slides (Ibidi) and incubated for 24 h. Lysosomes were labeled with LysoTracker Red (Thermofisher) (0.75 μl in 10 mL media) for 30 min. Cells were treated with 1 mM LLOMe for 10 min, washed, and allowed to recover for 30 or 60 min in the presence of LysoTracker. Nuclei were stained with Hoechst 33342 for 2 min prior to imaging.

### Image analysis

Fluorescent images were analyzed using ImageJ—FIJI distribution (NIH). TECPR1 lysosomal enrichment was determined in cells co‐transfected with EGFP‐TECPR1 and LAMP1‐mCherry. Five fields of view were imaged and stored using the multipoint feature in LASX. LLOMe was added to a final concentration of 1 mM, cells were incubated for 15 min, and fields were re‐imaged. mCherry images were used to generate an ROI in which the mean gray value of EGFP was quantified. Data are represented as the fold change in EGFP mean gray value for individual cells.

### Immunoblotting

Cells were lysed in ice‐cold lysis buffer (20 mM Tris–HCl pH8, 300 mM KCl, 10% Glycerol, 0.25% Nonidet P‐40, 0.5 mM EDTA, 1 mM PMSF, 1× complete protease inhibitor (Roche)), passed six times through a 21G needle and cleared by centrifugation (20 min/18,213 *g*/4°C). Lysates were subjected to SDS–PAGE and transferred to a 0.2 μm nitrocellulose membrane (Bio‐Rad) using a Trans‐Blot Turbo transfer system (Bio‐Rad). Membranes were blocked using 5% skim milk in TBST and incubated with primary antibody overnight at 4°C. Protein detection was carried out using chemiluminescence (Bio‐Rad) and imaged using a ChemiDoc imaging system (Bio‐Rad).

### Quantification and statistical analysis

Data are shown as mean ± standard deviation (SD). Statistical significance was determined by one‐way ANOVA or by Student's *t*‐tests (two‐tailed, unpaired), as indicated in the corresponding figure legend, using GraphPad Prism v.9.0.0. *P < 0.05, **P < 0.01, ***P < 0.001, ns = not significant. No blinding was performed.

## Author contributions

**Dale P Corkery:** Conceptualization; data curation; formal analysis; validation; investigation; visualization; writing – original draft; writing – review and editing. **Sergio Castro‐Gonzalez:** Investigation; writing – review and editing. **Anastasia Knyazeva:** Investigation; writing – review and editing. **Laura K Herzog:** Investigation; writing – review and editing. **Yao‐Wen Wu:** Conceptualization; formal analysis; supervision; funding acquisition; project administration; writing – review and editing.

## Disclosure and competing interests statement

The authors declare that they have no conflict of interest.

## Supporting information



Expanded View Figures PDF

PDF+

Source Data for Figure 1

Source Data for Figure 2

Source Data for Figure 3

Source Data for Figure 4

Source Data for Figure 5

## Data Availability

This study includes no data deposited in external repositories.
