# Supplementary material for: An ATG12‐ATG5‐TECPR1 E3‐like complex regulates unconventional LC3 lipidation at damaged lysosomes
Source: EMBO Rep. 2023 Jun 29;24(9):e56841. doi: 10.15252/embr.202356841 (PMC10481663; doi:10.15252/embr.202356841)
Supplement: Supplementary file 1 — Expanded View Figures PDF [file EMBR-24-e56841-s006.pdf]

# Expanded View Figures

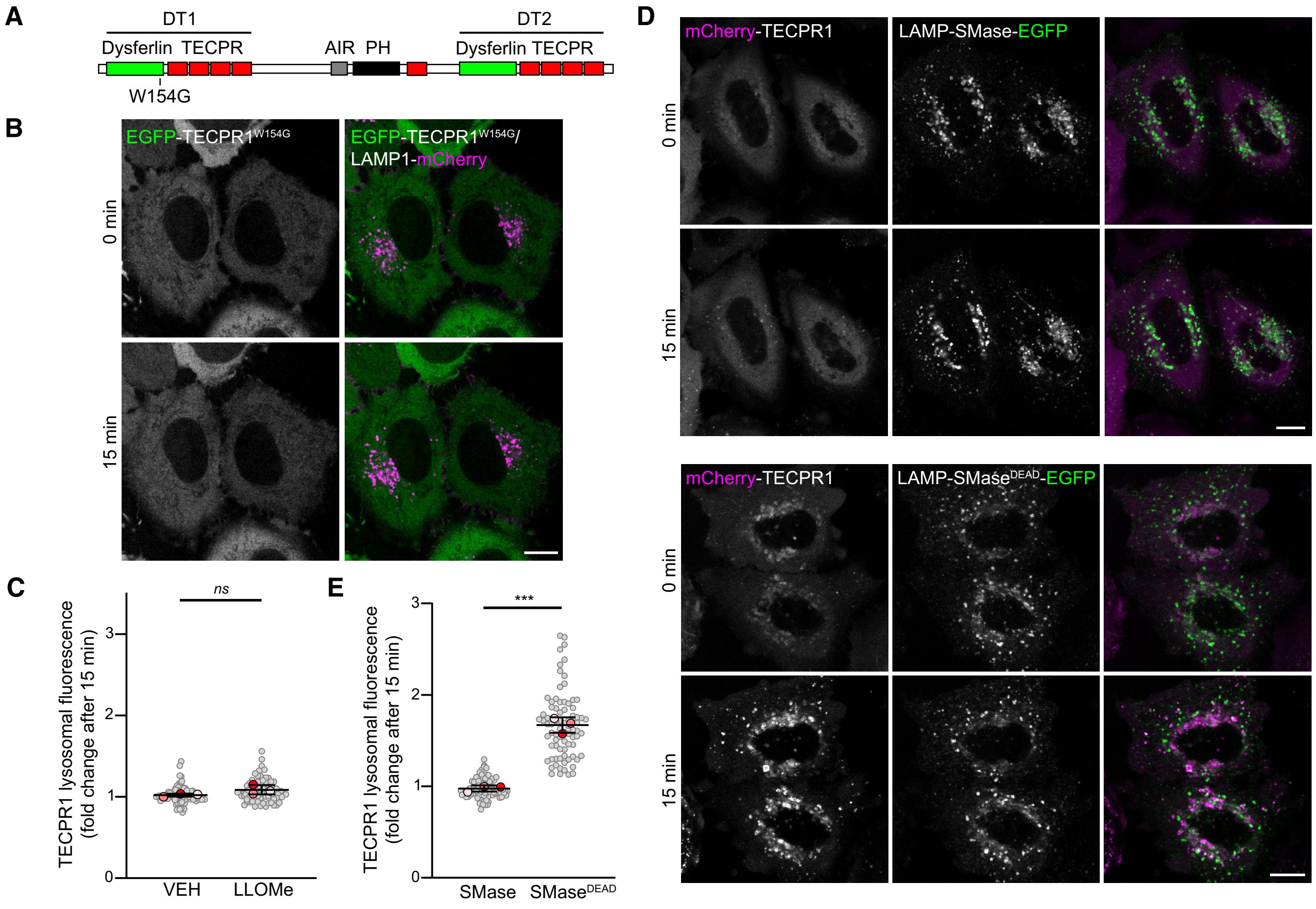


**Figure EV1. A sphingomyelin binding domain in the N-terminal dysferlin domain of TECPR1 mediates lysosomal recruitment.**

- A Location of the W154G mutation in TECPR1.
- B Representative live-cell fluorescent images of HeLa cells co-transfected with EGFP-TECPR1$^{W154G}$ and LAMP1-mCherry before and after treatment with 1 mM LLOMe. Scale bar = 10 μM.
- C Quantification of the fold change in EGFP-TECPR1 lysosomal fluorescence intensity after a 15-min treatment with vehicle or 1 mM LLOMe. Gray points represent individual cells from three independent experiments. Red points represent the means of individual experiments ($n$ > 25 cells per experiment). Bars represent the mean ± SD from the three experiments. Significance was determined from biological replicates using Student's $t$-tests. *ns* = not significant.
- D Representative live-cell fluorescent images of HeLa cells co-transfected with mCherry-TECPR1 and LAMP1-SMase-EGFP (top) or LAMP-SMase$^{DEAD}$-EGFP (bottom) before and after treatment with 1 mM LLOMe. Scale bar = 10 μM.
- E Quantification of the fold change in mCherry-TECPR1 lysosomal fluorescence intensity after a 15-min treatment with vehicle or 1 mM LLOMe (from D). Gray points represent individual cells from three independent experiments. Red points represent the means of individual experiments ($n$ > 25 cells per experiment). Bars represent the mean ± SD from the three experiments. Significance was determined from biological replicates using Student's $t$-tests. *** = 0.0002.

 © 2023 The Authors

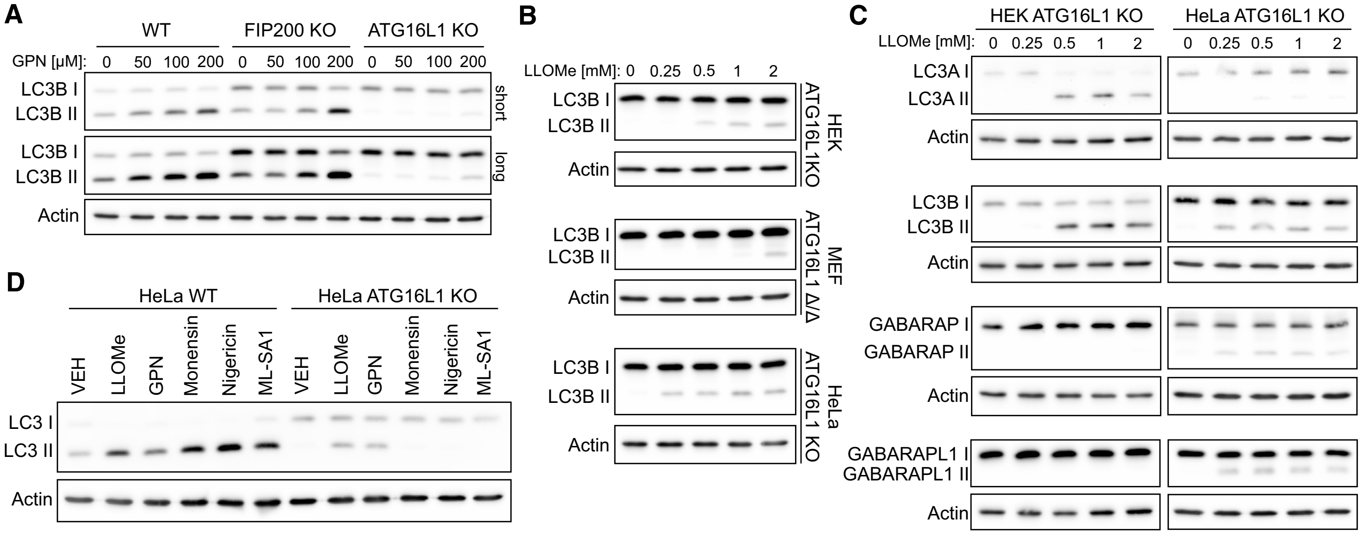


**Figure EV2. ATG8 lipidation in ATG16L1-deficient cell lines.**

A Western blot analysis of LC3 lipidation status in wild-type (WT), FIP200 KO, and ATG16L1 KO HeLa cells treated with the indicated concentrations of GPN for 30 min.
B Western blot analysis of LC3B lipidation status in HEK, MEF, and HeLa ATG16L1-deficient cell lines treated with the indicated concentration of LLOMe for 30 min.
C Western blot analysis of ATG8 lipidation status in HEK and HeLa ATG16L1-deficient cell lines treated with the indicated concentration of LLOMe for 30 min.
D Western blot analysis of LC3 lipidation status in HeLa WT and ATG16L1 KO cells treated as indicated.

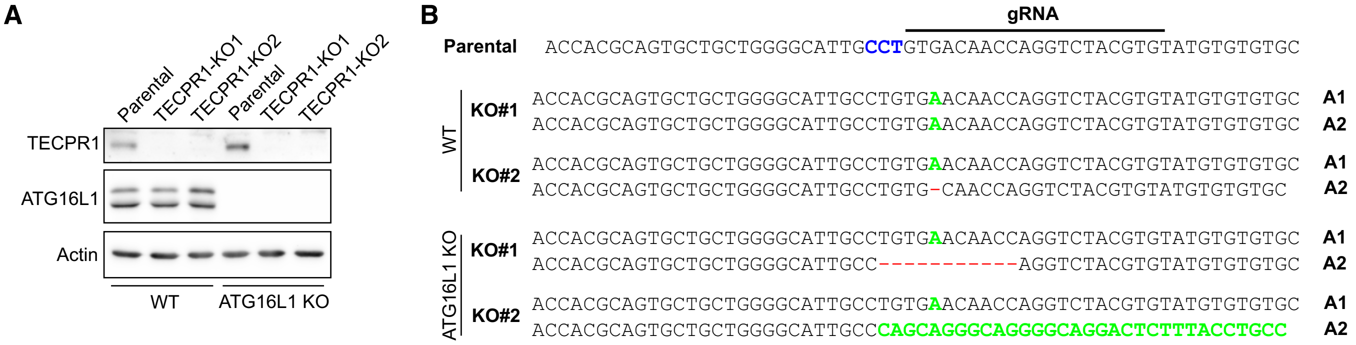


**Figure EV3. Validation of HEK TECPR1 KO cell lines.**

A Western blot analysis of TECPR1 and ATG16L1 protein levels in CRISPR-Cas9 clones.
B Sequencing results from genomic PCR of TECPR1 exon 3 from CRISPR-Cas9 clones. Green represents insertions and red dashes represent deletions.

© 2023 The Authors

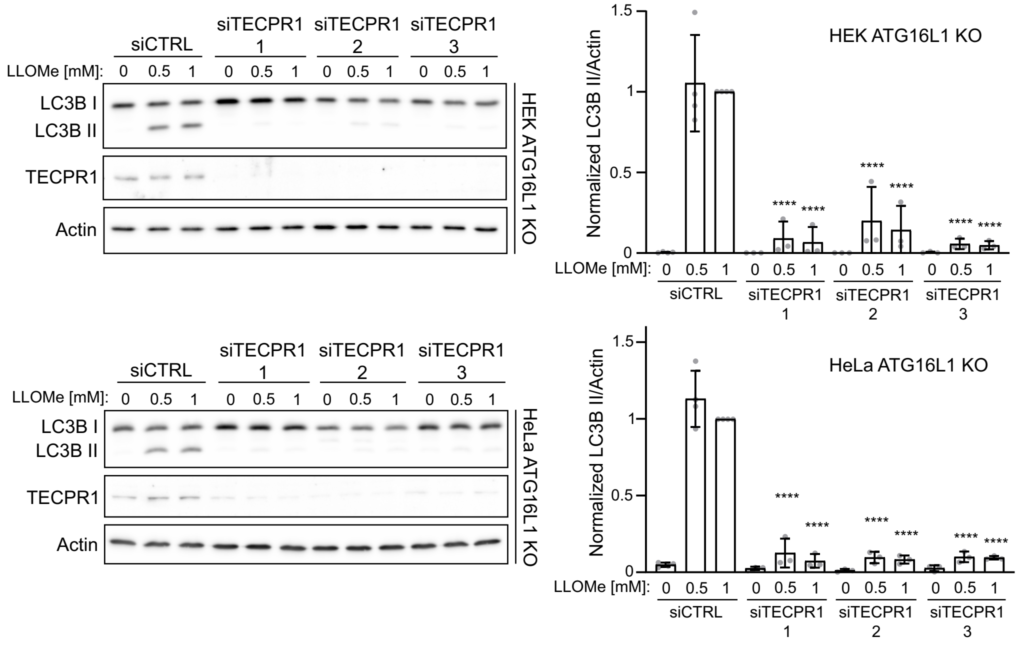


**Figure EV4. siRNA-mediated TECPR1 knockdown prevents ATG16L1-independent LC3 lipidation.**

Western blot analysis of LC3 lipidation status in HEK (top) and HeLa (bottom) ATG16 KO cells transfected with TECPR1 siRNAs for 72 h and treated with the indicated concentration of LLOMe for 30 min. To the right is the corresponding quantification of LC3-II protein levels. Bars show mean ± SD from three or four biologically independent experiments, which are represented as data points. Significance was determined from biological replicates using a one-way ANOVA with Tukey's multiple comparisons tests. ****$P < 0.0001$.

 © 2023 The Authors
