# Supplementary figures and images for: An ATG12‐ATG5‐TECPR1 E3‐like complex regulates unconventional LC3 lipidation at damaged lysosomes (part 1 of 4)
Source: EMBO Rep. 2023 Jun 29;24(9):e56841. doi: 10.15252/embr.202356841 (PMC10481663; doi:10.15252/embr.202356841)

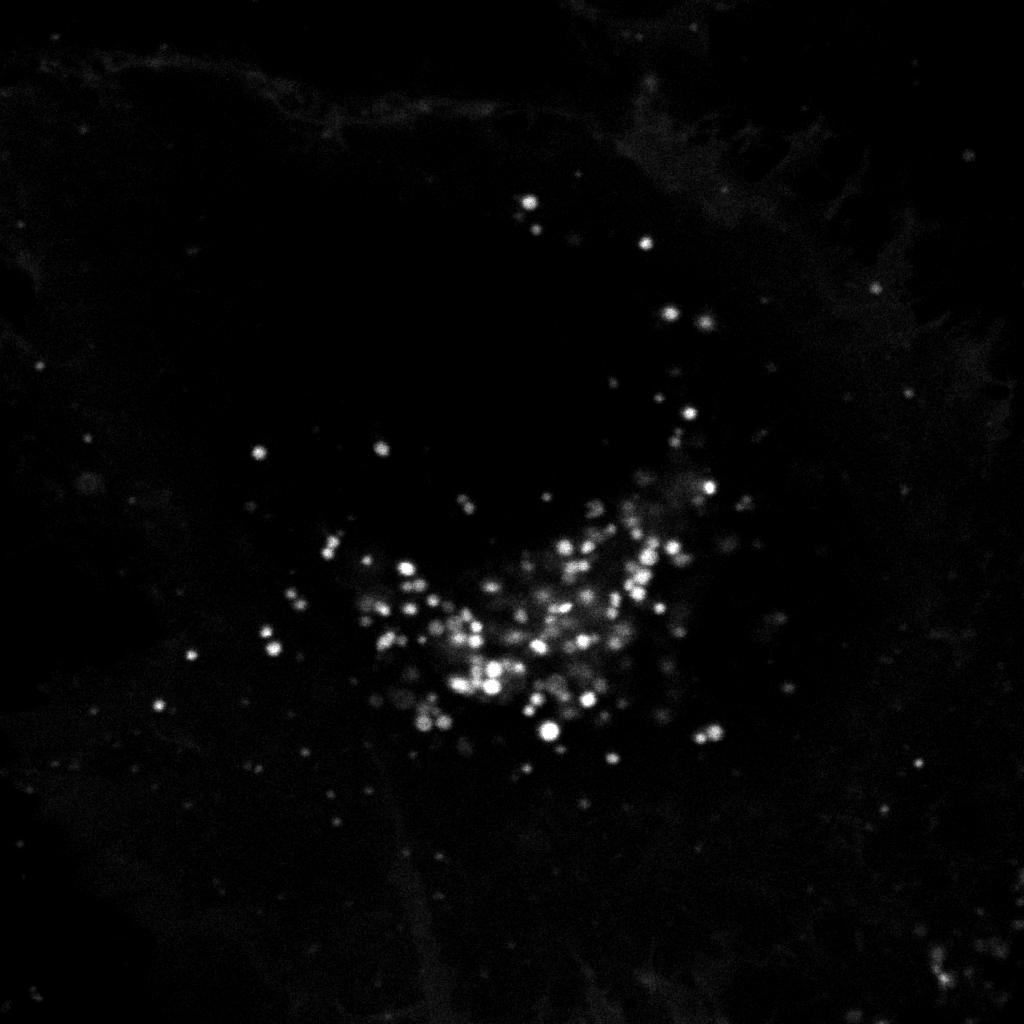

Supplement: Supplementary file 3 — Source Data for Figure 1 [file EMBR-24-e56841-s001.zip › Figure_1/1B/FULL_0min_LAMP.tif]

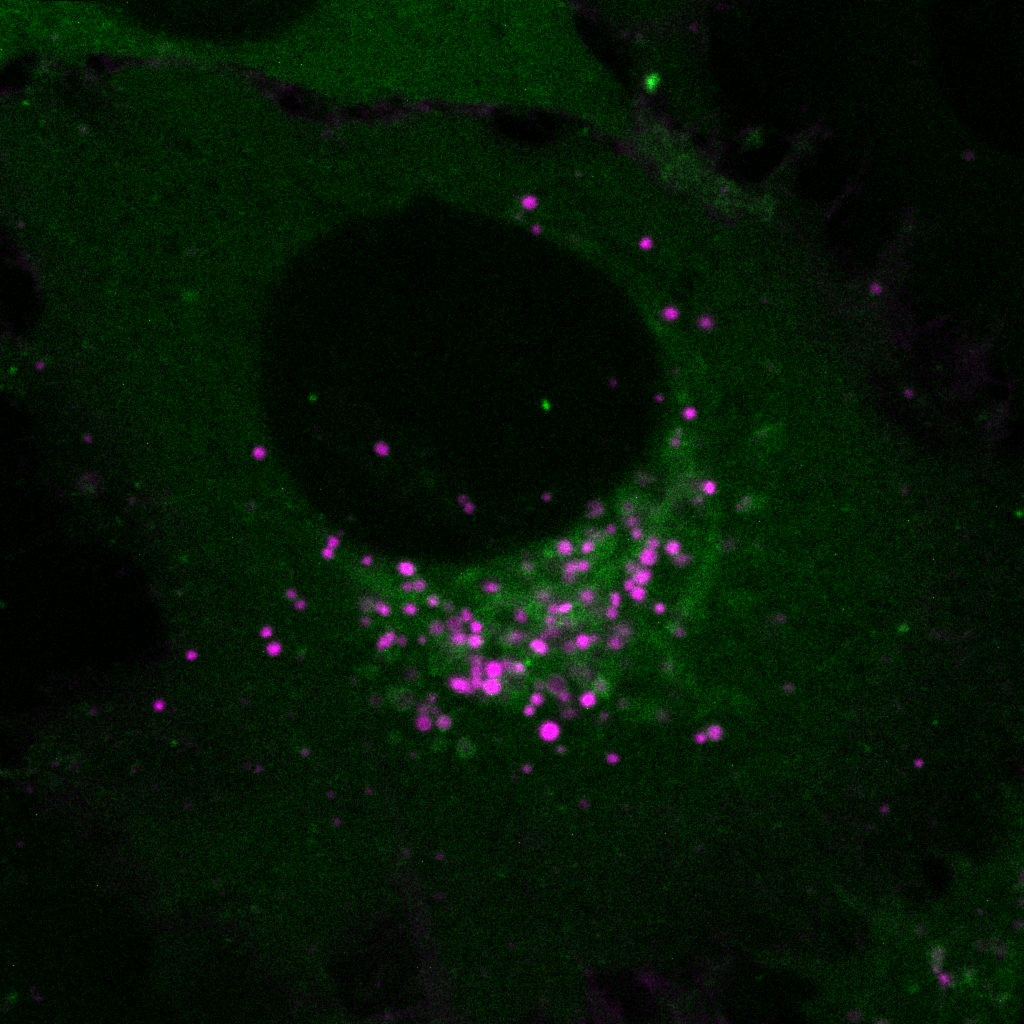

Supplement: Supplementary file 3 — Source Data for Figure 1 [file EMBR-24-e56841-s001.zip › Figure_1/1B/FULL_0min_LAMP_merge.tif]

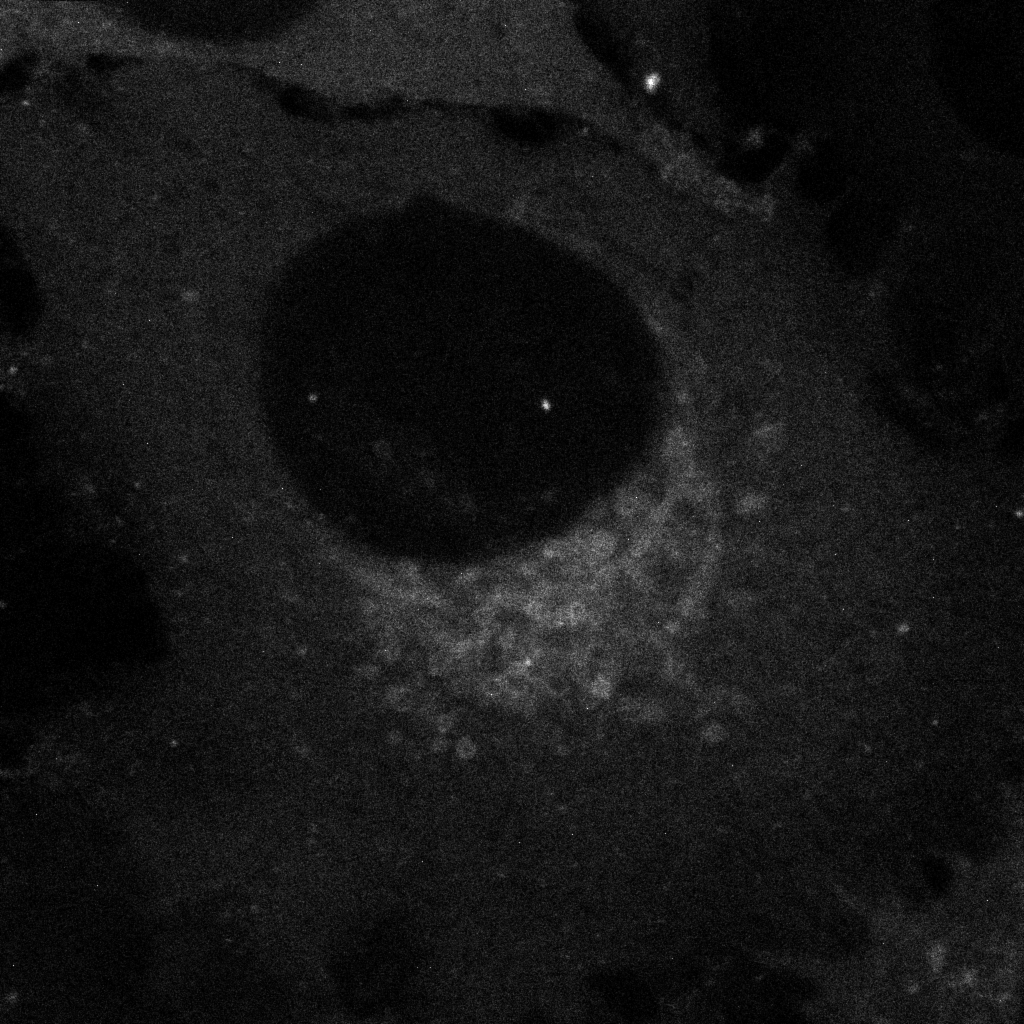

Supplement: Supplementary file 3 — Source Data for Figure 1 [file EMBR-24-e56841-s001.zip › Figure_1/1B/FULL_0min_TECPR1.tif]

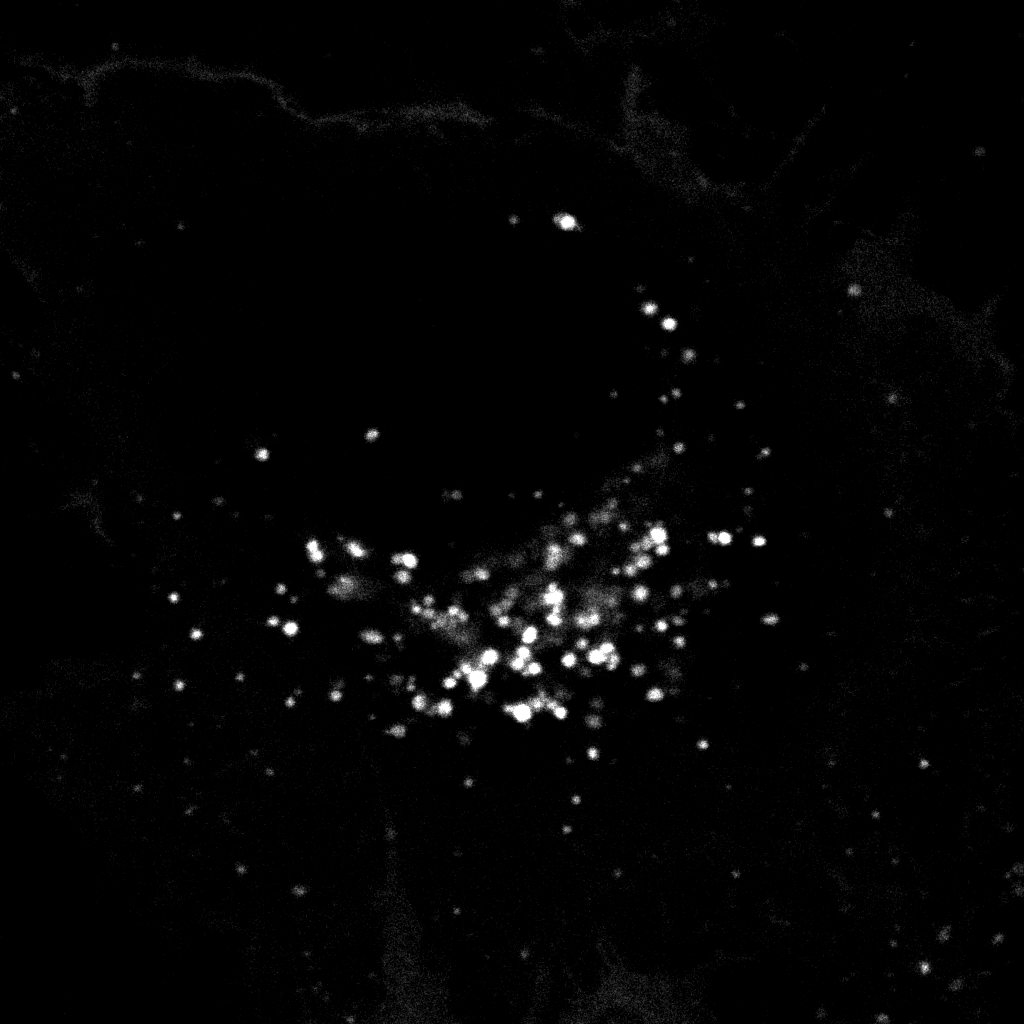

Supplement: Supplementary file 3 — Source Data for Figure 1 [file EMBR-24-e56841-s001.zip › Figure_1/1B/FULL_10min_LAMP.tif]

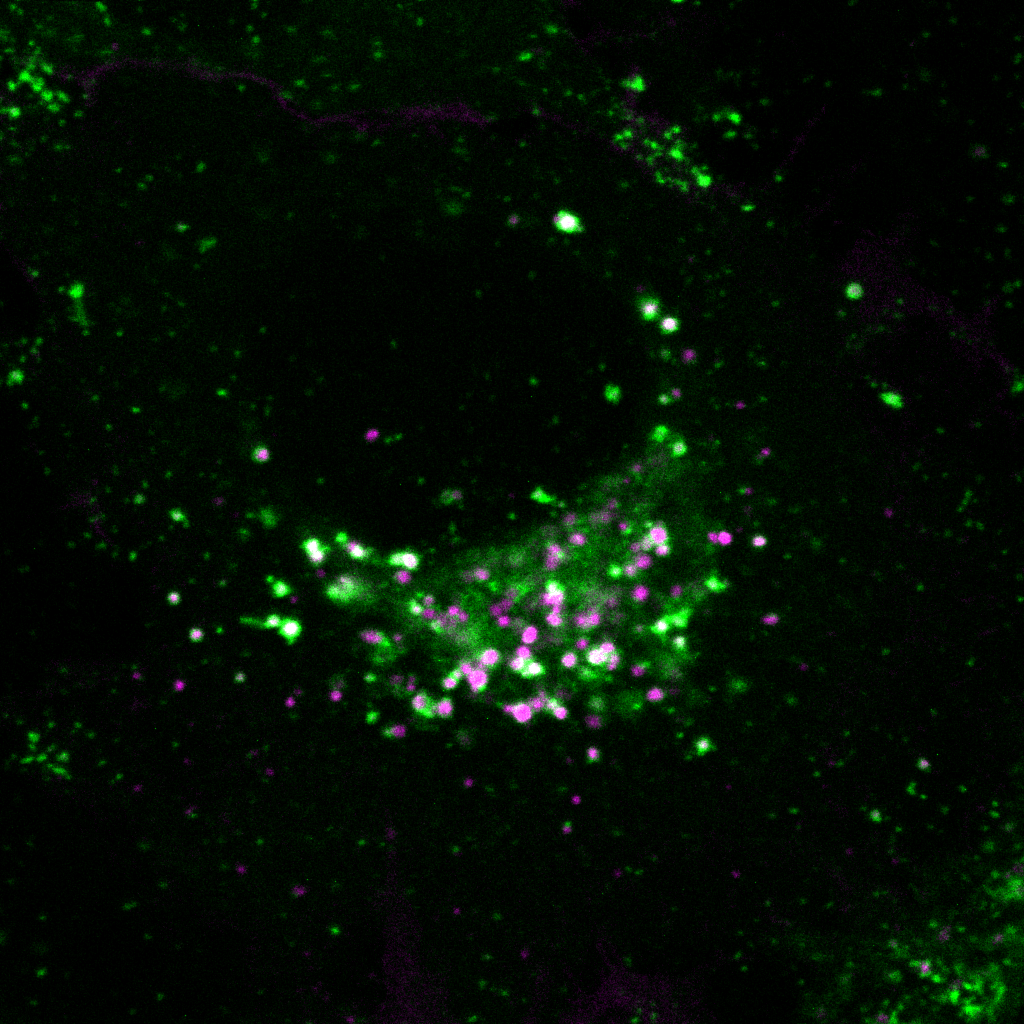

Supplement: Supplementary file 3 — Source Data for Figure 1 [file EMBR-24-e56841-s001.zip › Figure_1/1B/FULL_10min_LAMP_merge.tif]

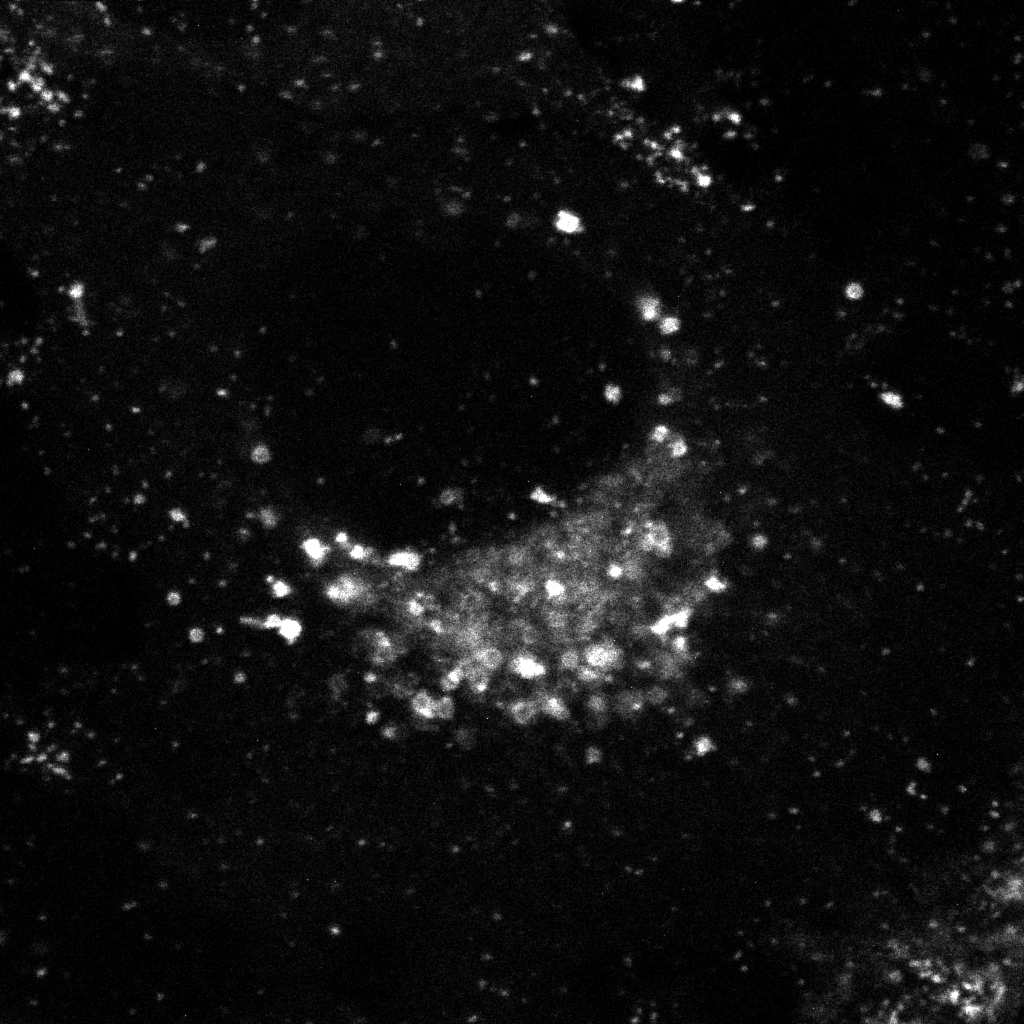

Supplement: Supplementary file 3 — Source Data for Figure 1 [file EMBR-24-e56841-s001.zip › Figure_1/1B/FULL_10min_TECPR1.tif]

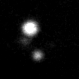

Supplement: Supplementary file 3 — Source Data for Figure 1 [file EMBR-24-e56841-s001.zip › Figure_1/1B/Time_series_zoom/0min_LAMP.tif]

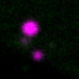

Supplement: Supplementary file 3 — Source Data for Figure 1 [file EMBR-24-e56841-s001.zip › Figure_1/1B/Time_series_zoom/0min_merge.tif]

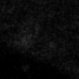

Supplement: Supplementary file 3 — Source Data for Figure 1 [file EMBR-24-e56841-s001.zip › Figure_1/1B/Time_series_zoom/0min_TECPR1.tif]

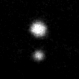

Supplement: Supplementary file 3 — Source Data for Figure 1 [file EMBR-24-e56841-s001.zip › Figure_1/1B/Time_series_zoom/220min_LAMP.tif]

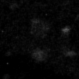

Supplement: Supplementary file 3 — Source Data for Figure 1 [file EMBR-24-e56841-s001.zip › Figure_1/1B/Time_series_zoom/220min_TECPR1.tif]

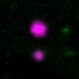

Supplement: Supplementary file 3 — Source Data for Figure 1 [file EMBR-24-e56841-s001.zip › Figure_1/1B/Time_series_zoom/220_merge.tif]

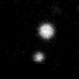

Supplement: Supplementary file 3 — Source Data for Figure 1 [file EMBR-24-e56841-s001.zip › Figure_1/1B/Time_series_zoom/230min_LAMP.tif]

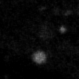

Supplement: Supplementary file 3 — Source Data for Figure 1 [file EMBR-24-e56841-s001.zip › Figure_1/1B/Time_series_zoom/230min_TECPR1.tif]

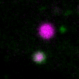

Supplement: Supplementary file 3 — Source Data for Figure 1 [file EMBR-24-e56841-s001.zip › Figure_1/1B/Time_series_zoom/230_merge.tif]

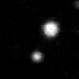

Supplement: Supplementary file 3 — Source Data for Figure 1 [file EMBR-24-e56841-s001.zip › Figure_1/1B/Time_series_zoom/240min_LAMP.tif]

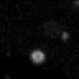

Supplement: Supplementary file 3 — Source Data for Figure 1 [file EMBR-24-e56841-s001.zip › Figure_1/1B/Time_series_zoom/240min_TECPR1.tif]

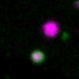

Supplement: Supplementary file 3 — Source Data for Figure 1 [file EMBR-24-e56841-s001.zip › Figure_1/1B/Time_series_zoom/240_merge.tif]

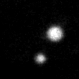

Supplement: Supplementary file 3 — Source Data for Figure 1 [file EMBR-24-e56841-s001.zip › Figure_1/1B/Time_series_zoom/250min_LAMP.tif]

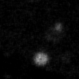

Supplement: Supplementary file 3 — Source Data for Figure 1 [file EMBR-24-e56841-s001.zip › Figure_1/1B/Time_series_zoom/250min_TECPR1.tif]

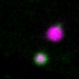

Supplement: Supplementary file 3 — Source Data for Figure 1 [file EMBR-24-e56841-s001.zip › Figure_1/1B/Time_series_zoom/250_merge.tif]

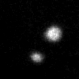

Supplement: Supplementary file 3 — Source Data for Figure 1 [file EMBR-24-e56841-s001.zip › Figure_1/1B/Time_series_zoom/260min_LAMP.tif]

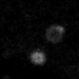

Supplement: Supplementary file 3 — Source Data for Figure 1 [file EMBR-24-e56841-s001.zip › Figure_1/1B/Time_series_zoom/260min_TECPR1.tif]

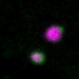

Supplement: Supplementary file 3 — Source Data for Figure 1 [file EMBR-24-e56841-s001.zip › Figure_1/1B/Time_series_zoom/260_merge.tif]

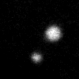

Supplement: Supplementary file 3 — Source Data for Figure 1 [file EMBR-24-e56841-s001.zip › Figure_1/1B/Time_series_zoom/270min_LAMP.tif]

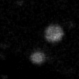

Supplement: Supplementary file 3 — Source Data for Figure 1 [file EMBR-24-e56841-s001.zip › Figure_1/1B/Time_series_zoom/270min_TECPR1.tif]

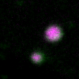

Supplement: Supplementary file 3 — Source Data for Figure 1 [file EMBR-24-e56841-s001.zip › Figure_1/1B/Time_series_zoom/270_merge.tif]

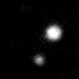

Supplement: Supplementary file 3 — Source Data for Figure 1 [file EMBR-24-e56841-s001.zip › Figure_1/1B/Time_series_zoom/280min_LAMP.tif]

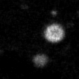

Supplement: Supplementary file 3 — Source Data for Figure 1 [file EMBR-24-e56841-s001.zip › Figure_1/1B/Time_series_zoom/280min_TECPR1.tif]

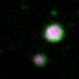

Supplement: Supplementary file 3 — Source Data for Figure 1 [file EMBR-24-e56841-s001.zip › Figure_1/1B/Time_series_zoom/280_merge.tif]

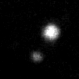

Supplement: Supplementary file 3 — Source Data for Figure 1 [file EMBR-24-e56841-s001.zip › Figure_1/1B/Time_series_zoom/290min_LAMP.tif]

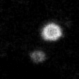

Supplement: Supplementary file 3 — Source Data for Figure 1 [file EMBR-24-e56841-s001.zip › Figure_1/1B/Time_series_zoom/290min_TECPR1.tif]

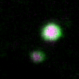

Supplement: Supplementary file 3 — Source Data for Figure 1 [file EMBR-24-e56841-s001.zip › Figure_1/1B/Time_series_zoom/290_merge.tif]

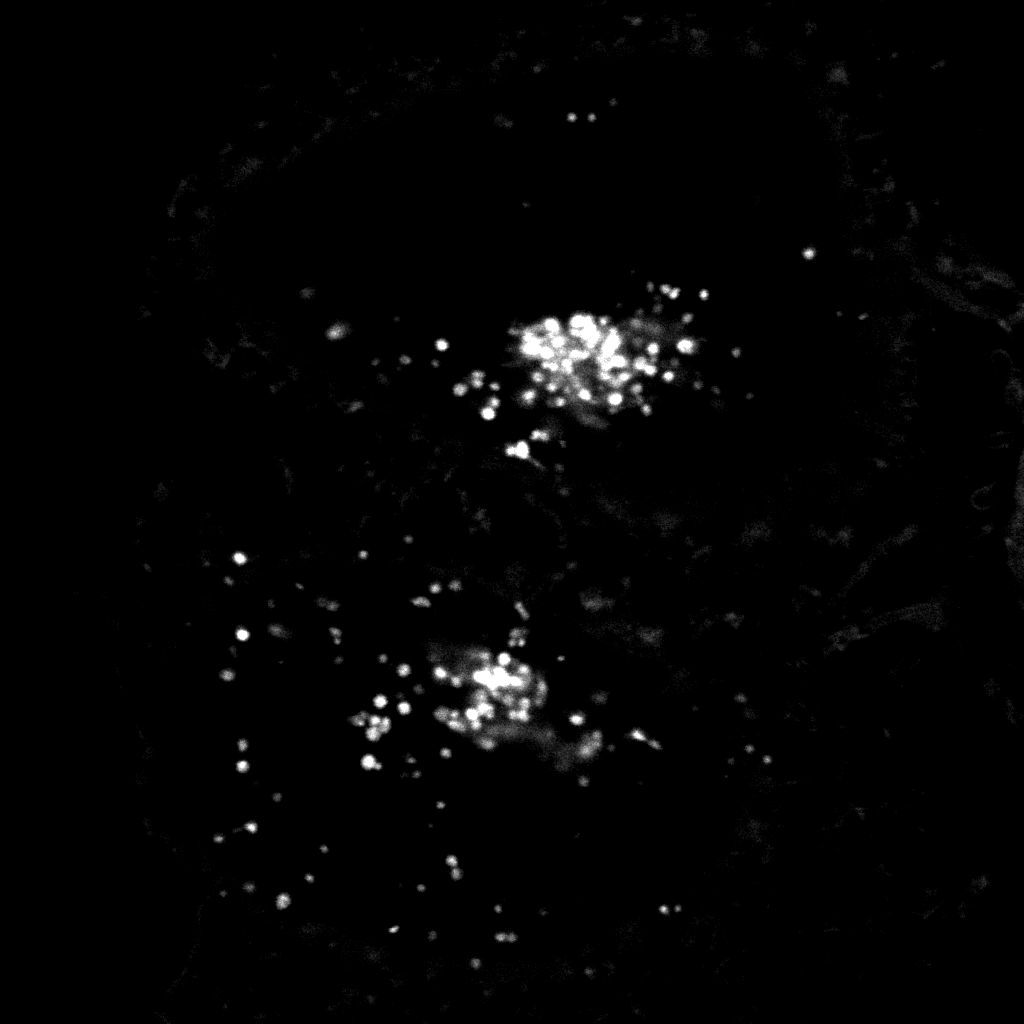

Supplement: Supplementary file 3 — Source Data for Figure 1 [file EMBR-24-e56841-s001.zip › Figure_1/1D/GPN_0min_LAMP.tif]

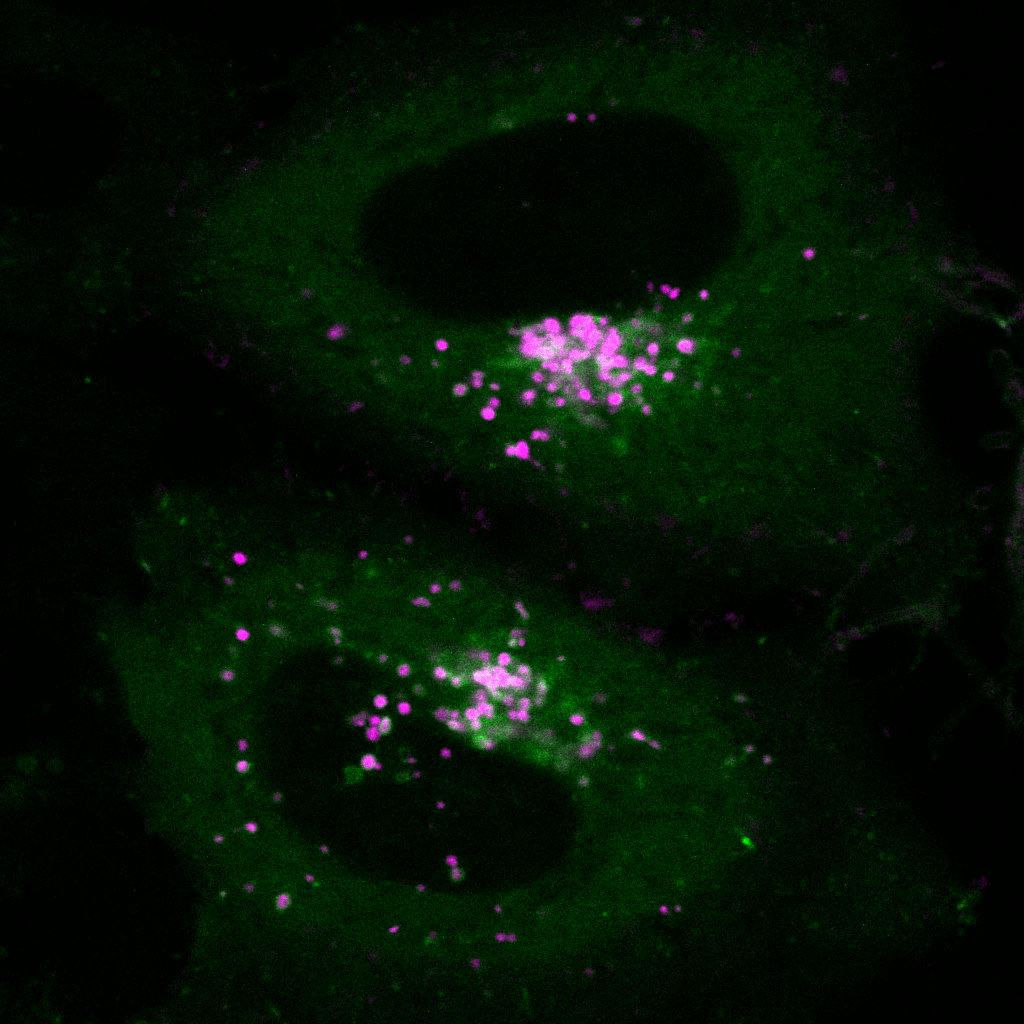

Supplement: Supplementary file 3 — Source Data for Figure 1 [file EMBR-24-e56841-s001.zip › Figure_1/1D/GPN_0min_merge.tif]

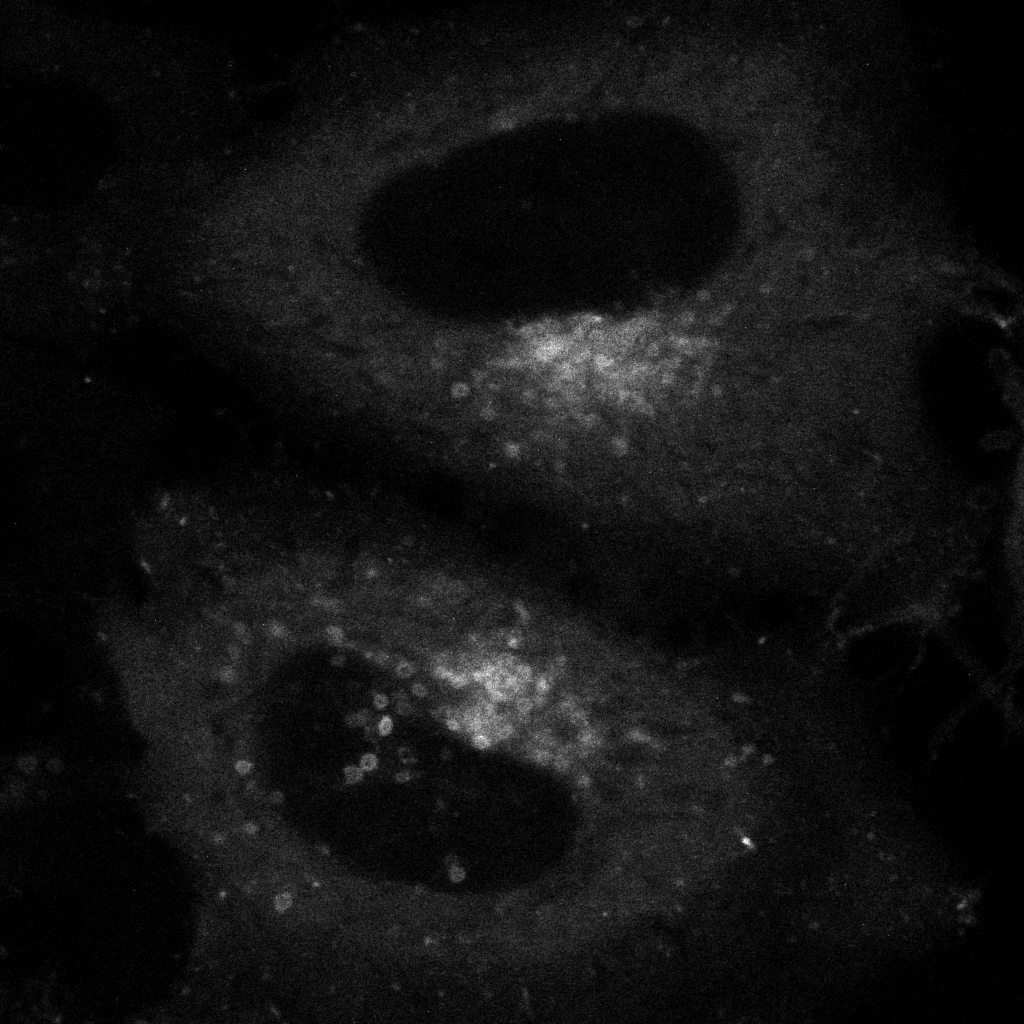

Supplement: Supplementary file 3 — Source Data for Figure 1 [file EMBR-24-e56841-s001.zip › Figure_1/1D/GPN_0min_TECPR1.tif]

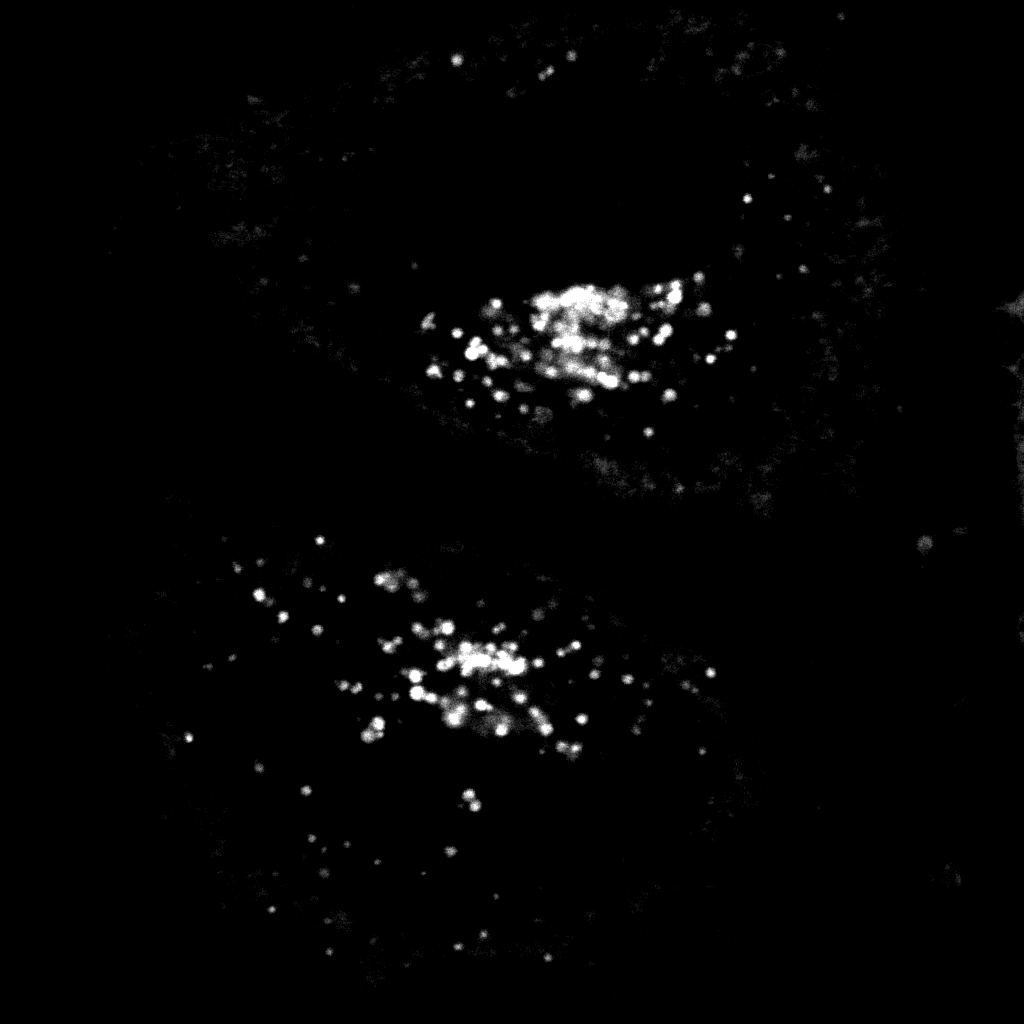

Supplement: Supplementary file 3 — Source Data for Figure 1 [file EMBR-24-e56841-s001.zip › Figure_1/1D/GPN_15min_LAMP.tif]

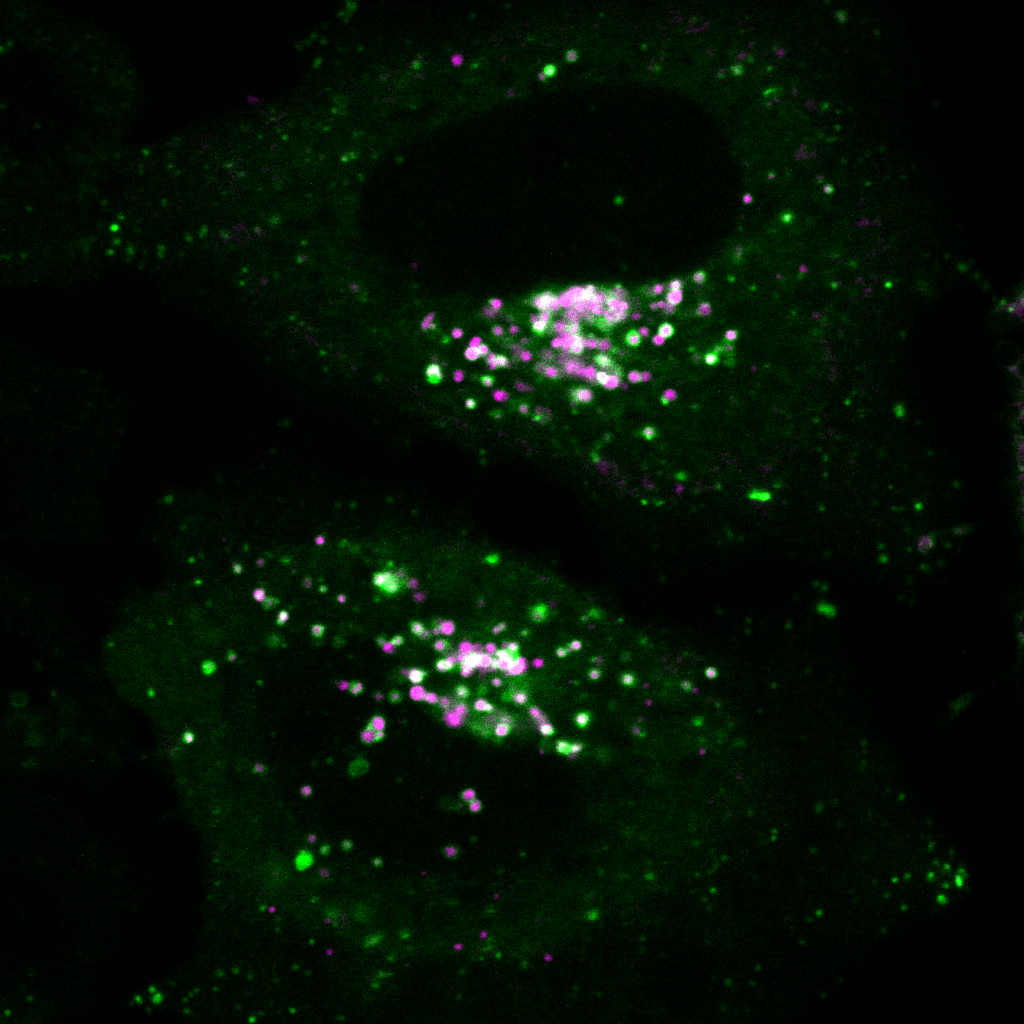

Supplement: Supplementary file 3 — Source Data for Figure 1 [file EMBR-24-e56841-s001.zip › Figure_1/1D/GPN_15min_merge.tif]

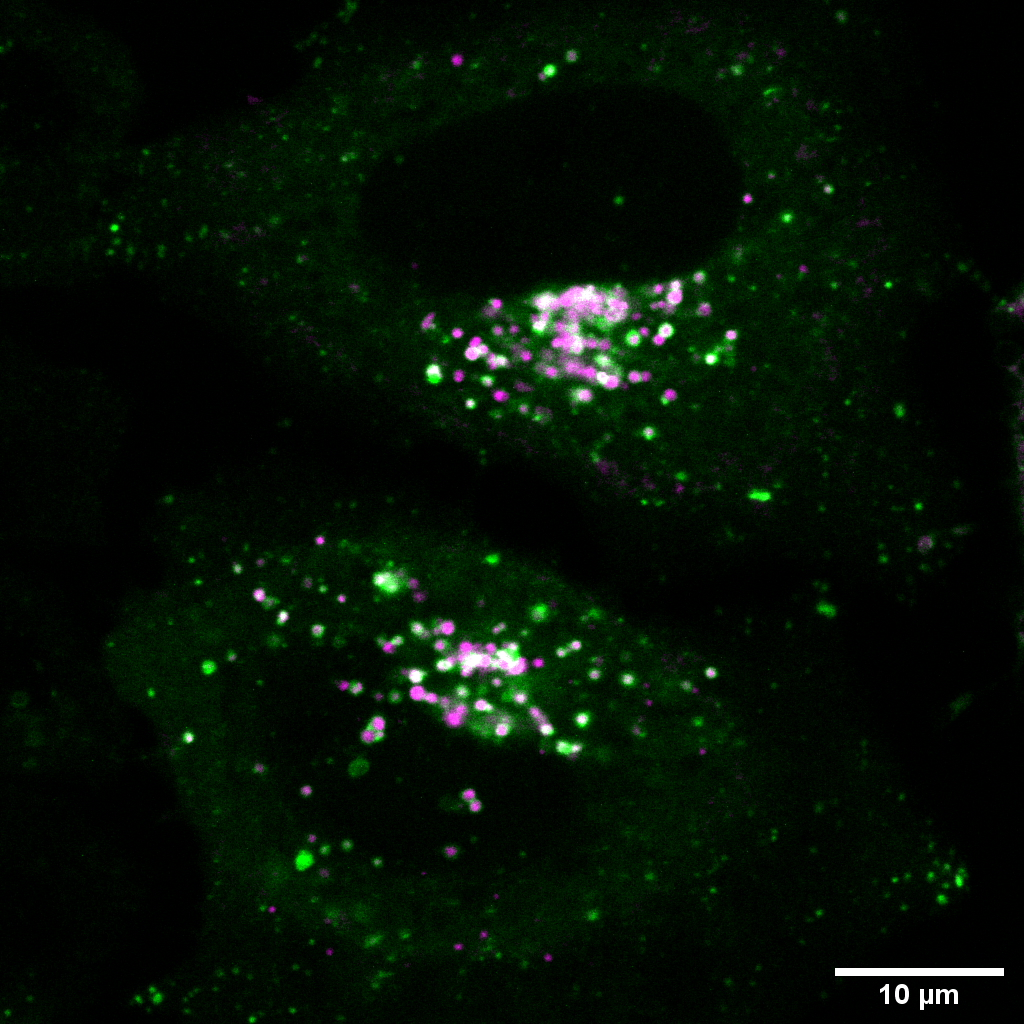

Supplement: Supplementary file 3 — Source Data for Figure 1 [file EMBR-24-e56841-s001.zip › Figure_1/1D/GPN_15min_scale.tif]

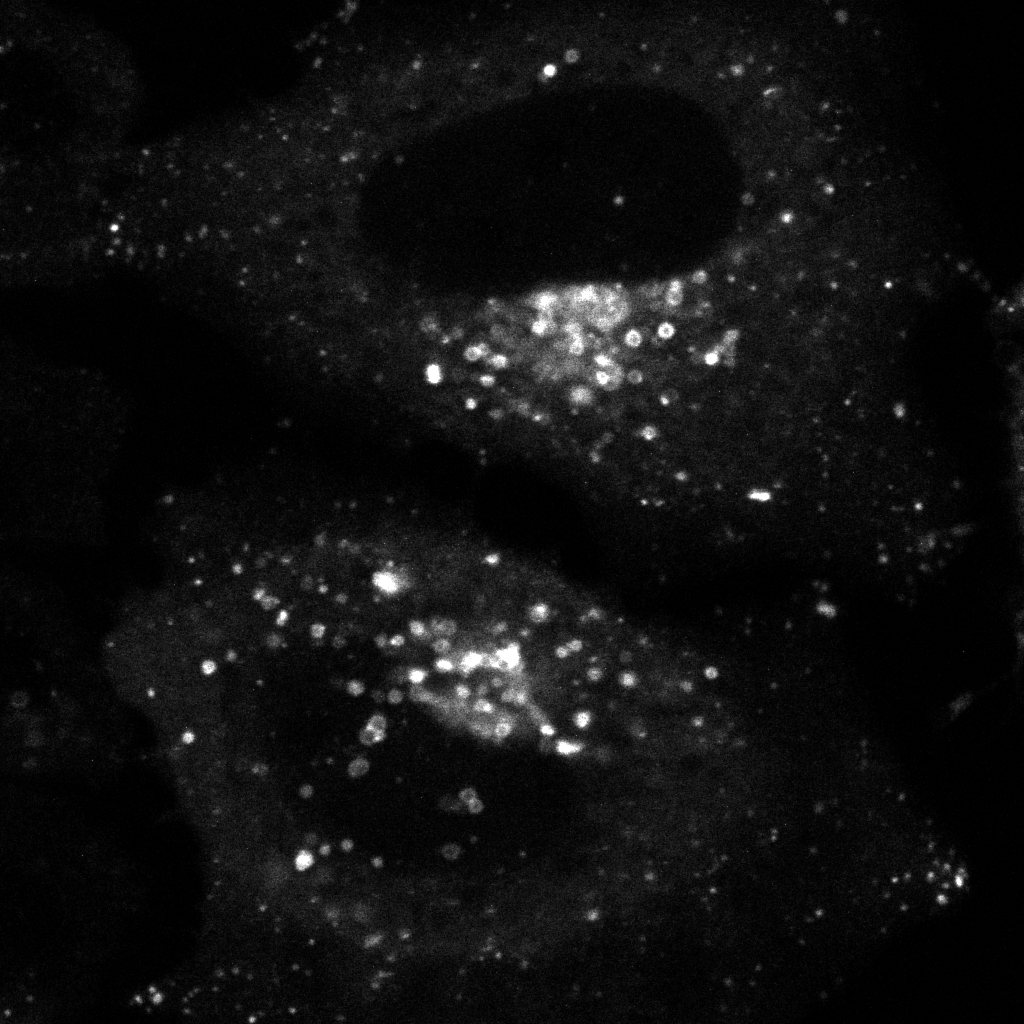

Supplement: Supplementary file 3 — Source Data for Figure 1 [file EMBR-24-e56841-s001.zip › Figure_1/1D/GPN_15min_TECPR1.tif]

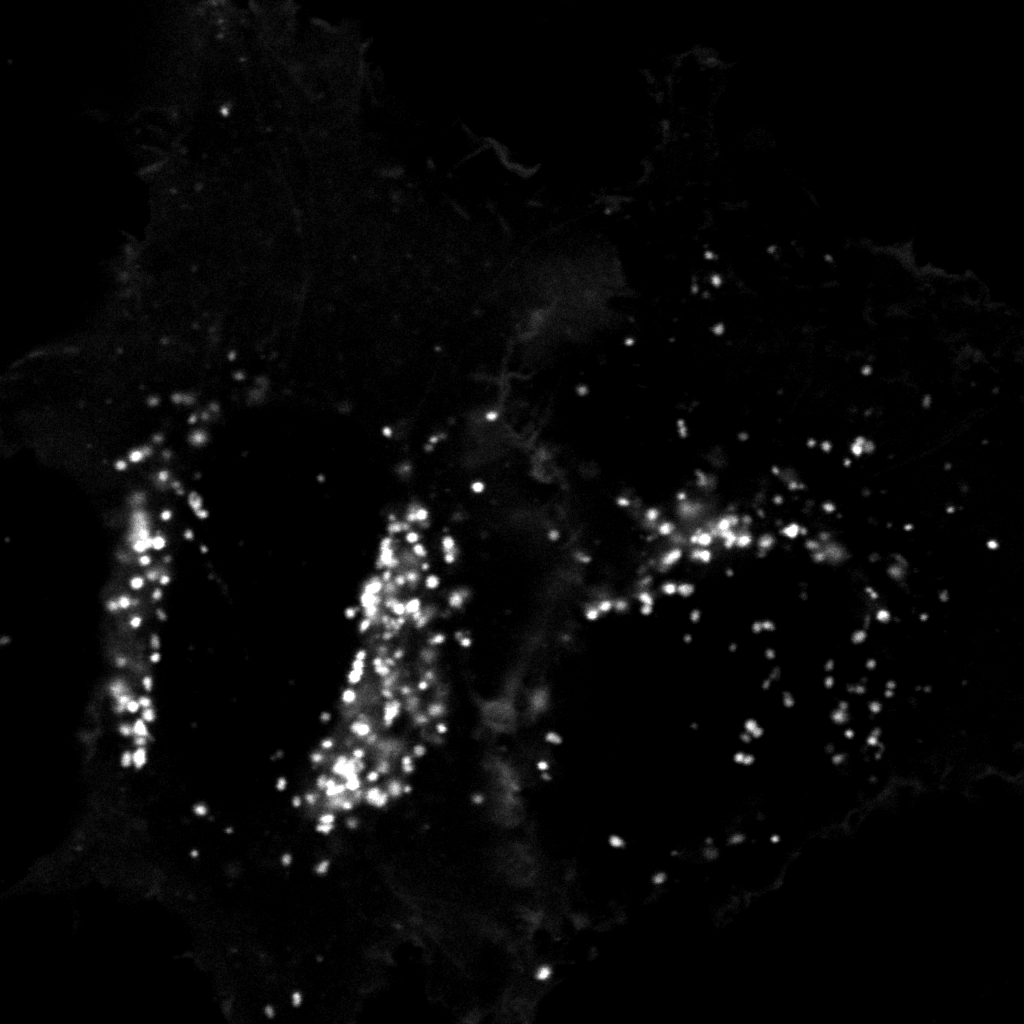

Supplement: Supplementary file 3 — Source Data for Figure 1 [file EMBR-24-e56841-s001.zip › Figure_1/1D/LLOMe_0min_LAMP.tif]

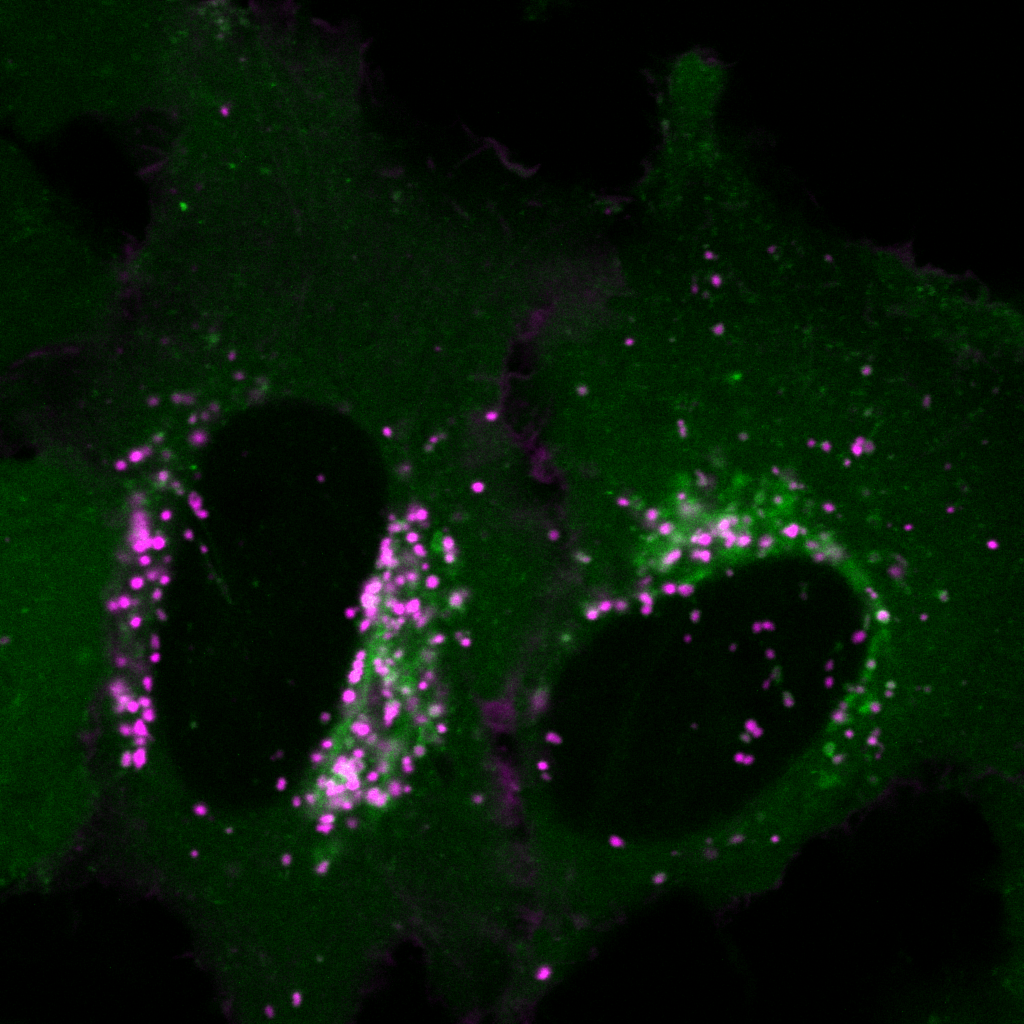

Supplement: Supplementary file 3 — Source Data for Figure 1 [file EMBR-24-e56841-s001.zip › Figure_1/1D/LLOMe_0min_merge.tif]

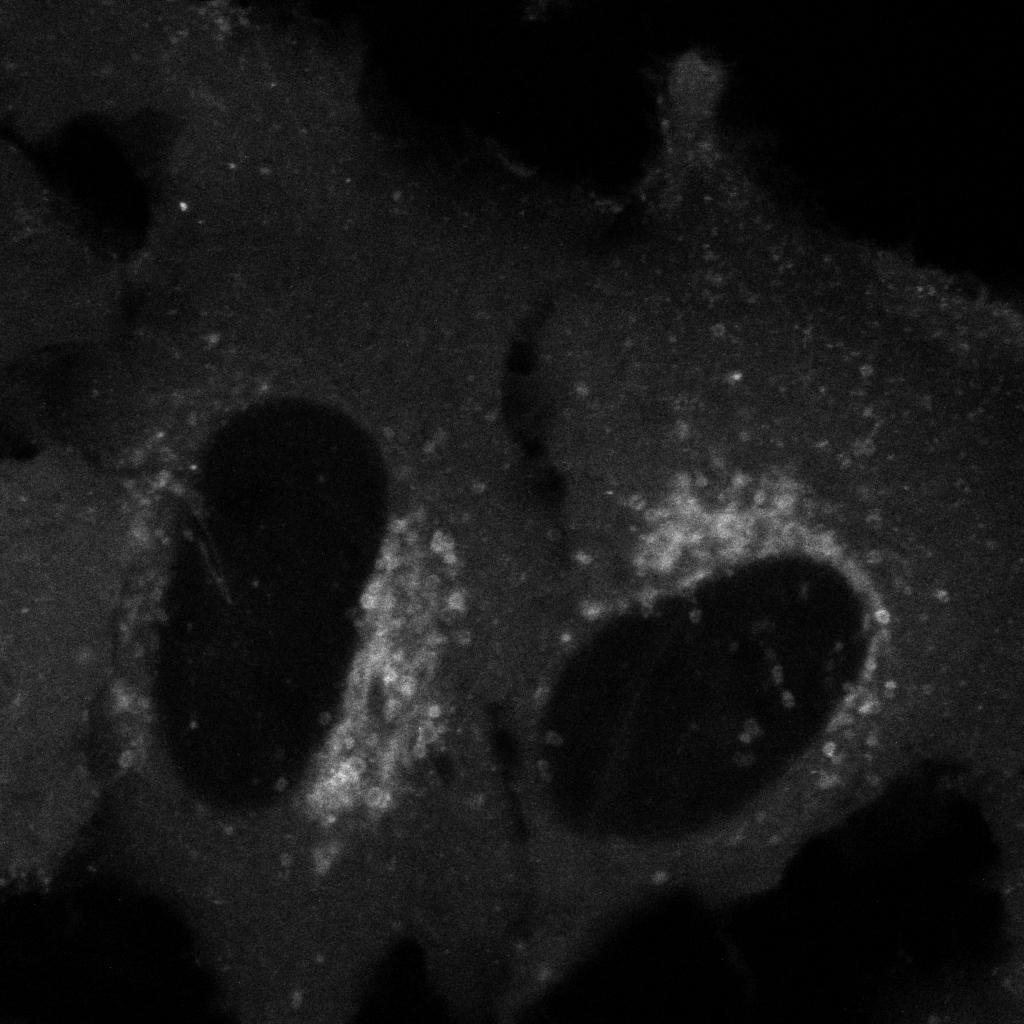

Supplement: Supplementary file 3 — Source Data for Figure 1 [file EMBR-24-e56841-s001.zip › Figure_1/1D/LLOMe_0min_TECPR1.tif]

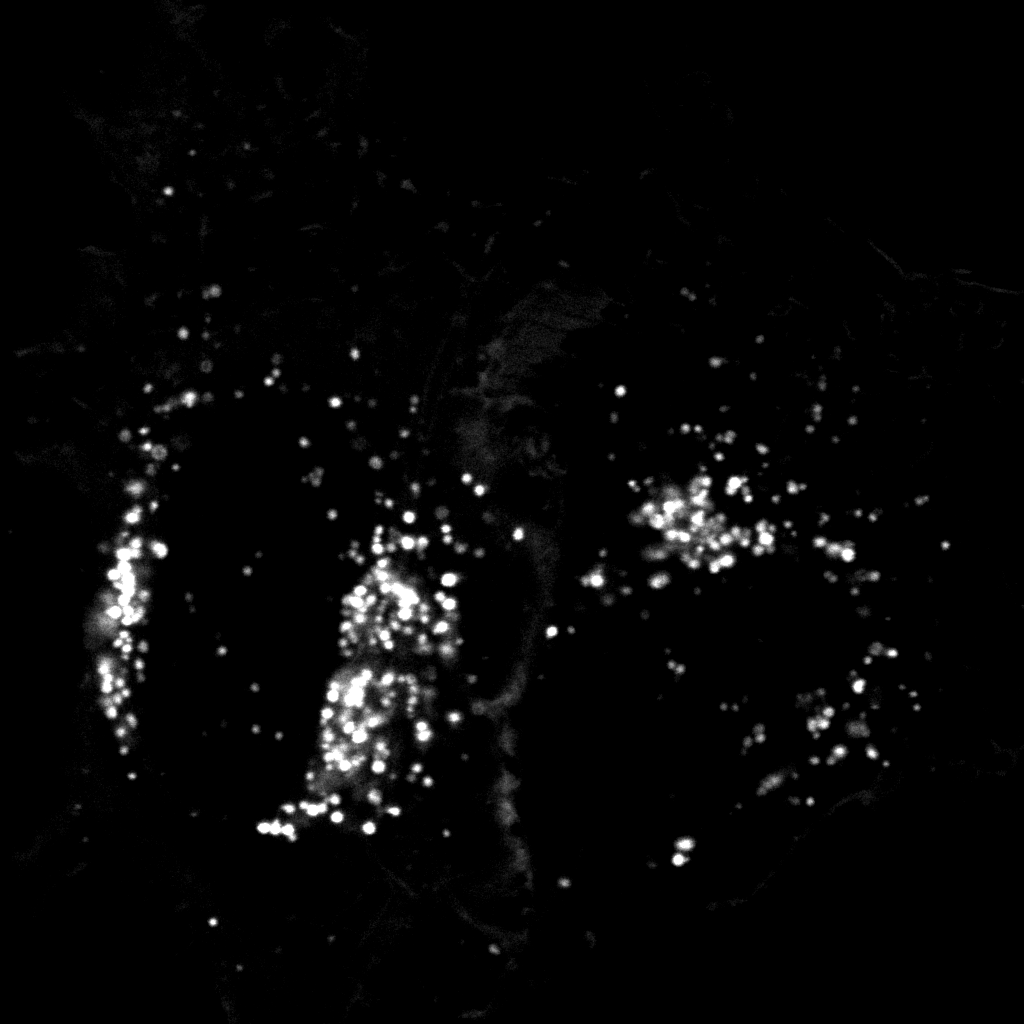

Supplement: Supplementary file 3 — Source Data for Figure 1 [file EMBR-24-e56841-s001.zip › Figure_1/1D/LLOMe_15min_LAMP.tif]

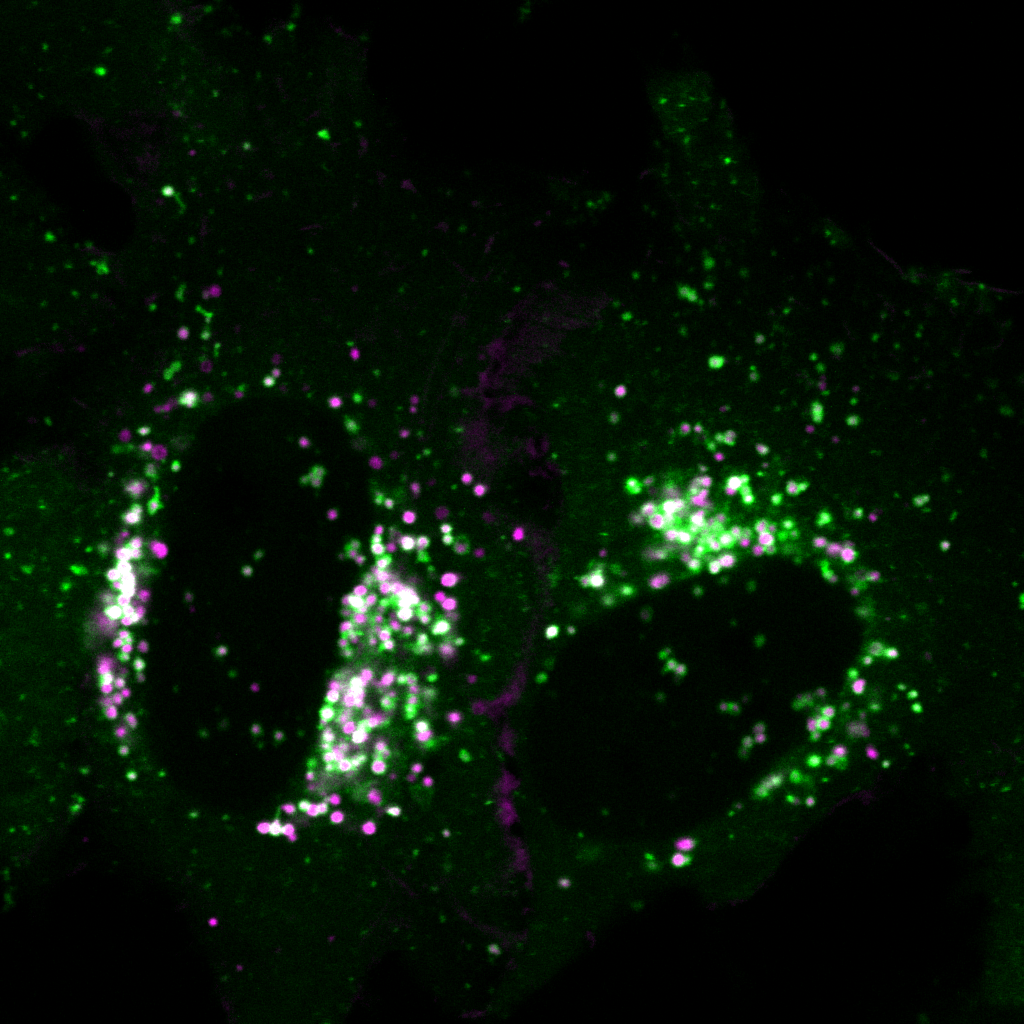

Supplement: Supplementary file 3 — Source Data for Figure 1 [file EMBR-24-e56841-s001.zip › Figure_1/1D/LLOMe_15min_merge.tif]

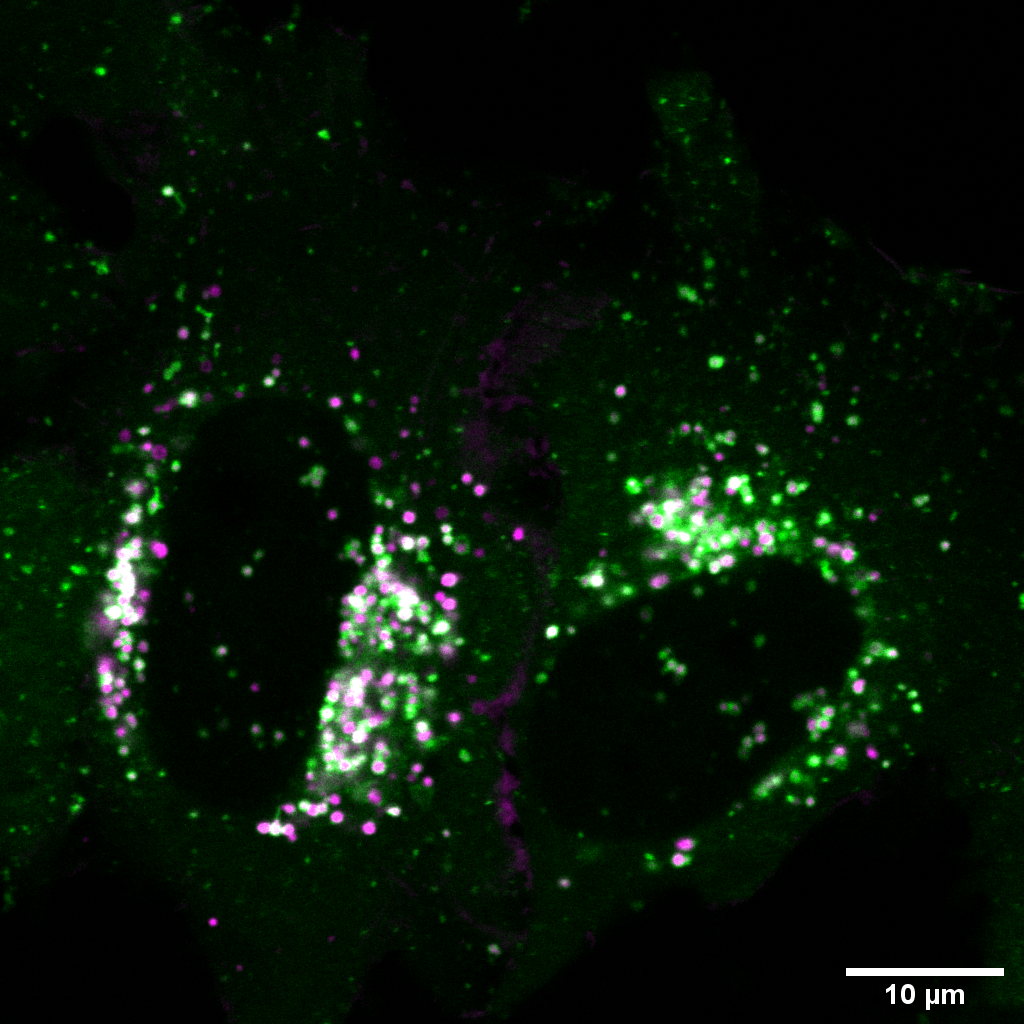

Supplement: Supplementary file 3 — Source Data for Figure 1 [file EMBR-24-e56841-s001.zip › Figure_1/1D/LLOMe_15min_scale.tif]

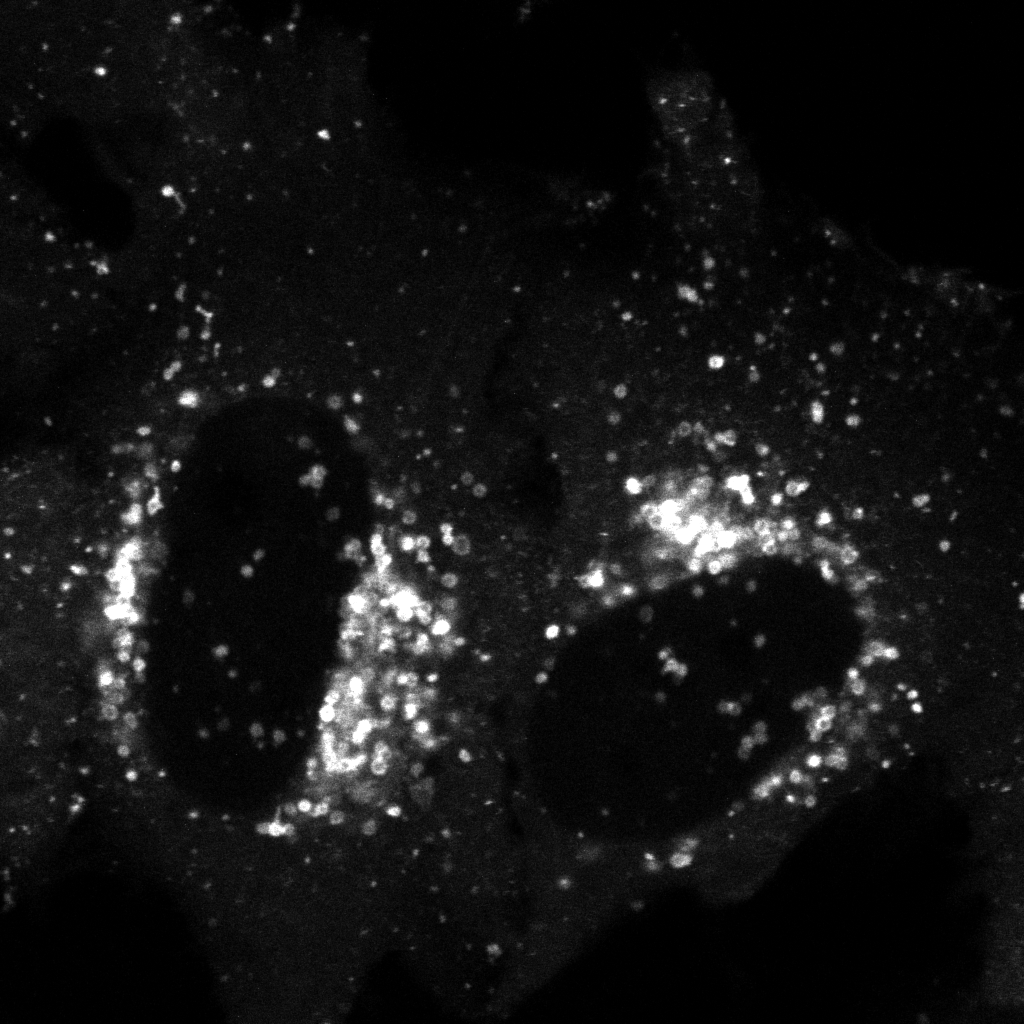

Supplement: Supplementary file 3 — Source Data for Figure 1 [file EMBR-24-e56841-s001.zip › Figure_1/1D/LLOMe_15min_TECPR1.tif]

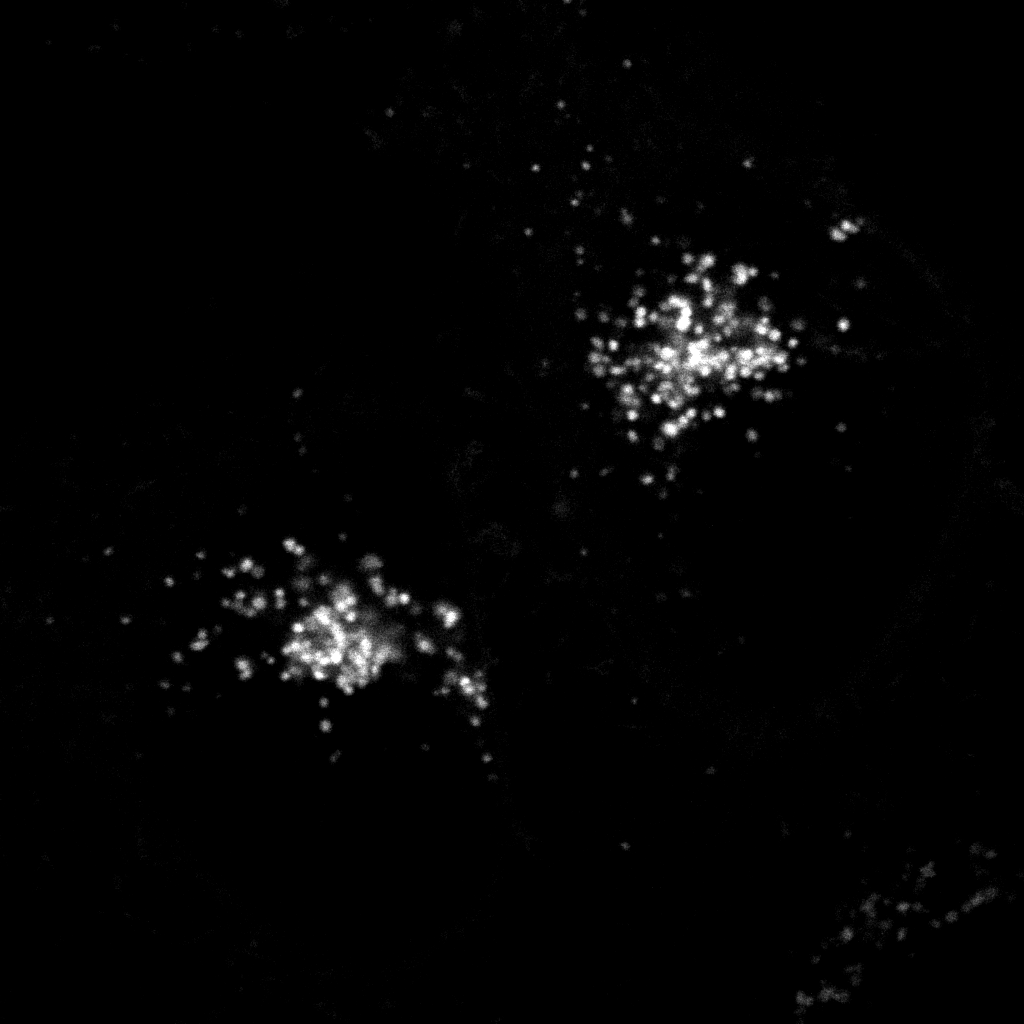

Supplement: Supplementary file 3 — Source Data for Figure 1 [file EMBR-24-e56841-s001.zip › Figure_1/1D/ML-SA1_0h_LAMP.tif]

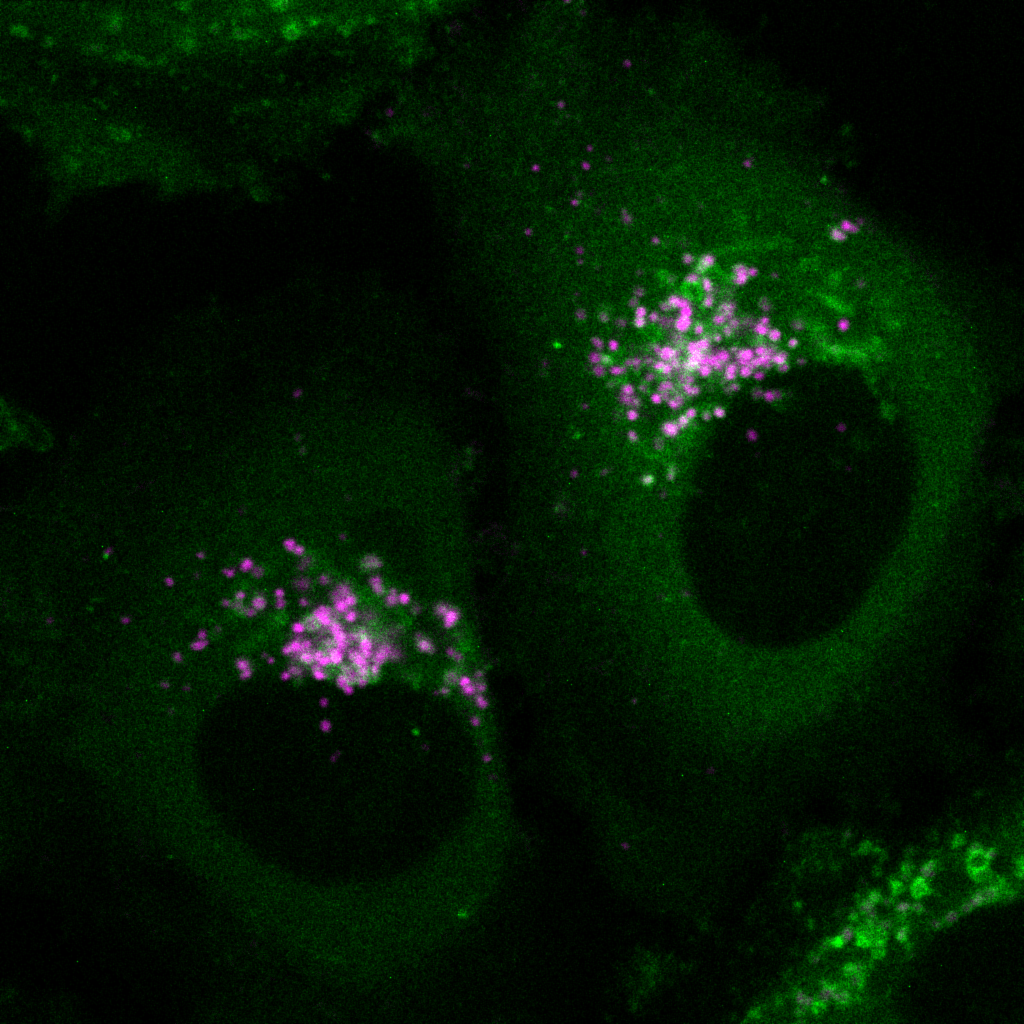

Supplement: Supplementary file 3 — Source Data for Figure 1 [file EMBR-24-e56841-s001.zip › Figure_1/1D/ML-SA1_0h_merge.tif]

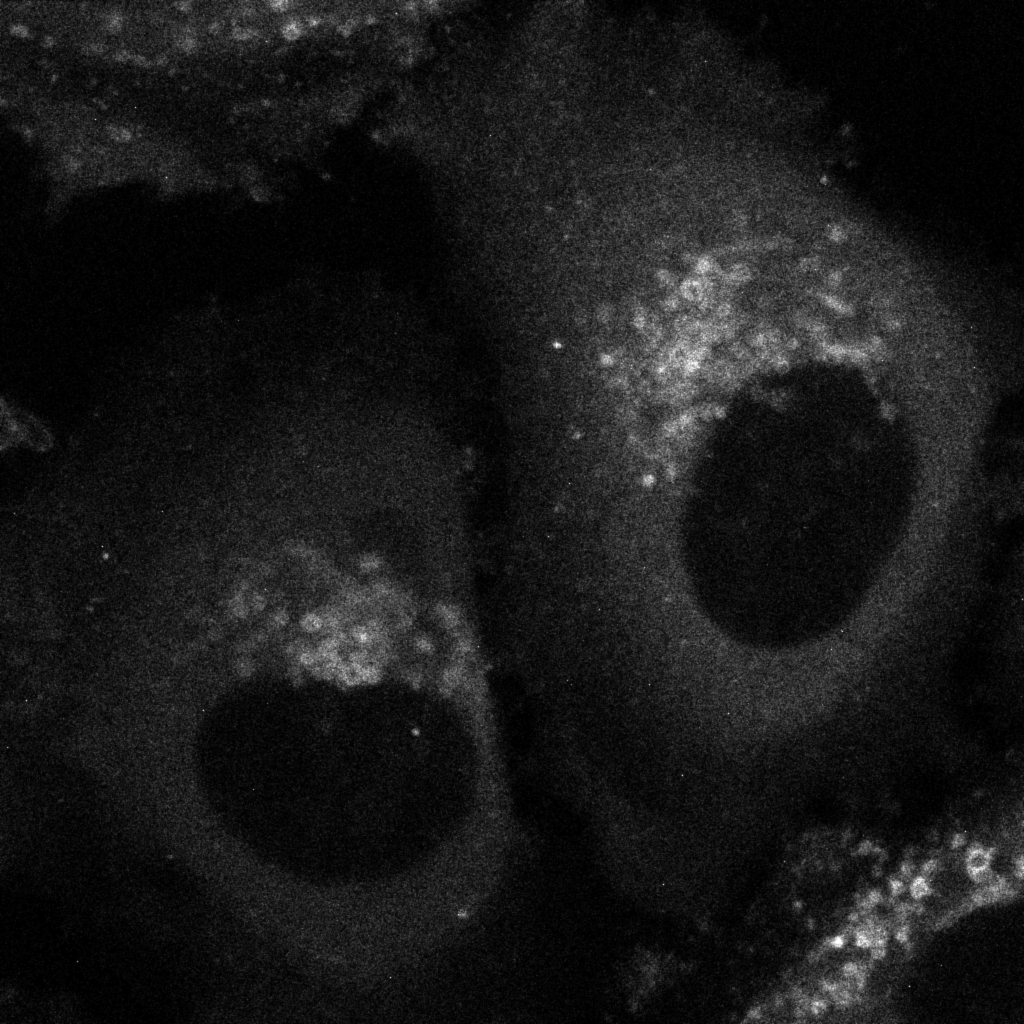

Supplement: Supplementary file 3 — Source Data for Figure 1 [file EMBR-24-e56841-s001.zip › Figure_1/1D/ML-SA1_0h_TECPR1.tif]

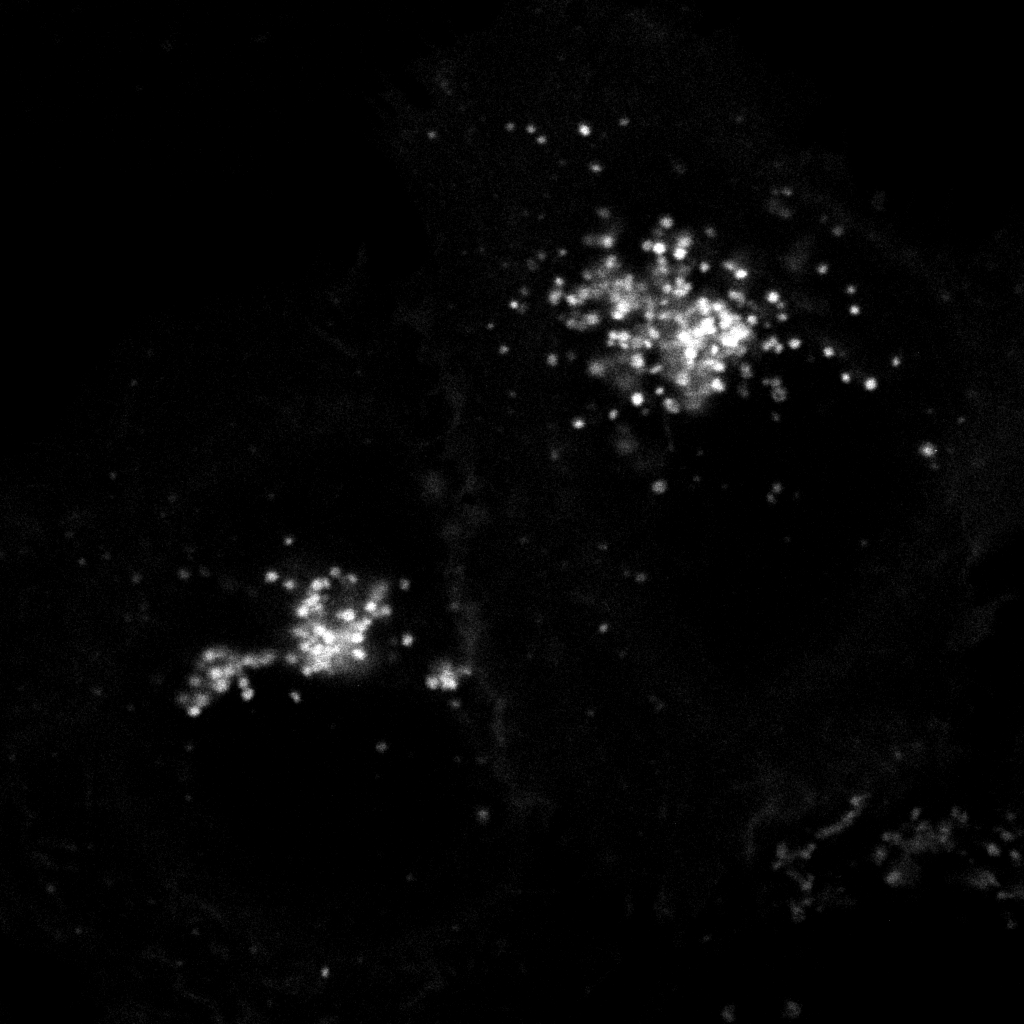

Supplement: Supplementary file 3 — Source Data for Figure 1 [file EMBR-24-e56841-s001.zip › Figure_1/1D/ML-SA1_1h_LAMP.tif]

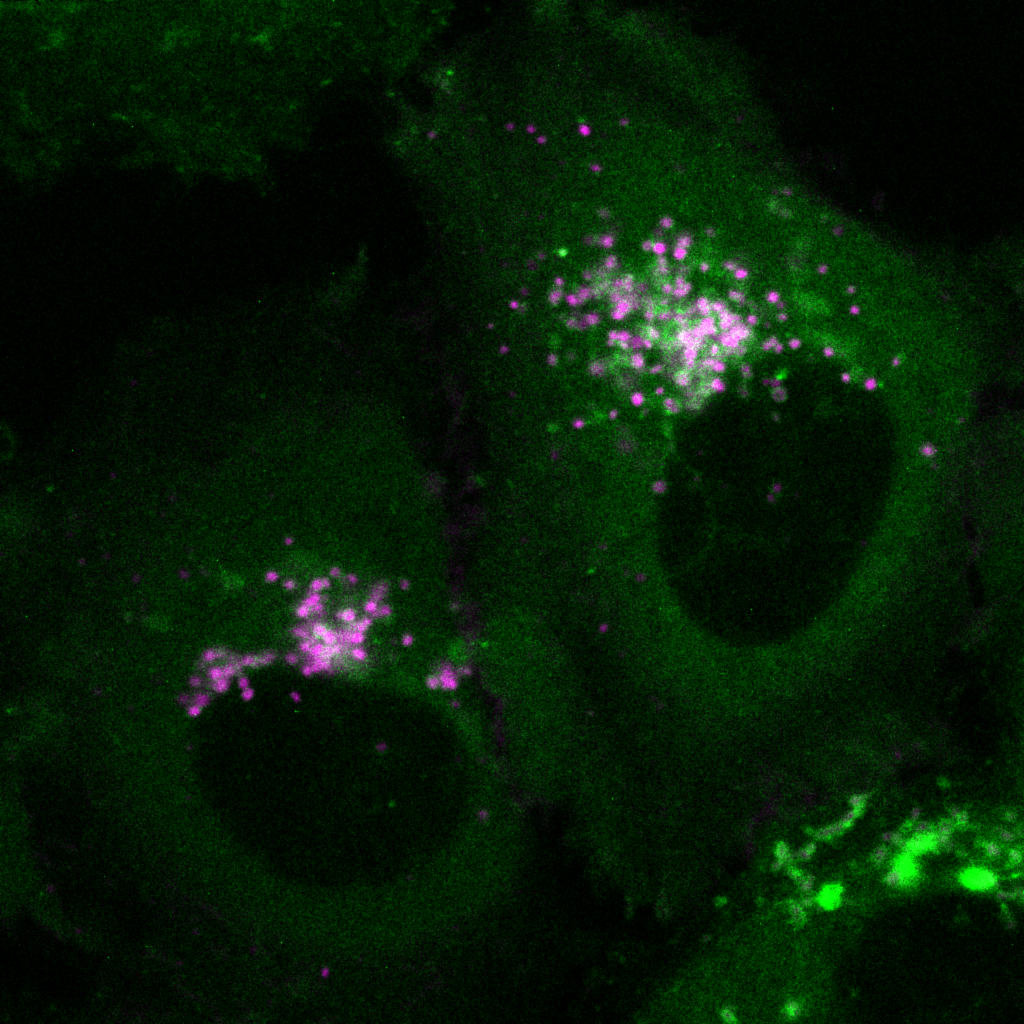

Supplement: Supplementary file 3 — Source Data for Figure 1 [file EMBR-24-e56841-s001.zip › Figure_1/1D/ML-SA1_1h_merge.tif]

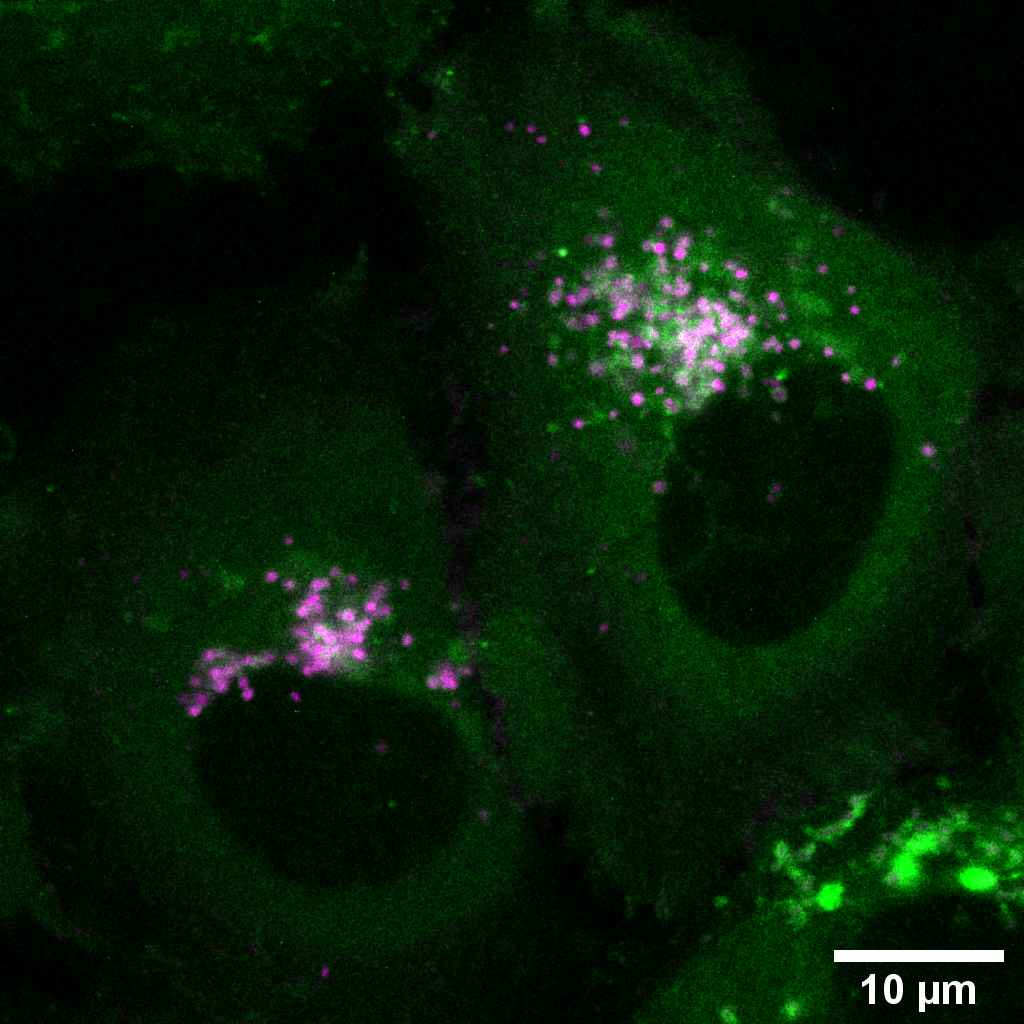

Supplement: Supplementary file 3 — Source Data for Figure 1 [file EMBR-24-e56841-s001.zip › Figure_1/1D/ML-SA1_1h_scale.tif]

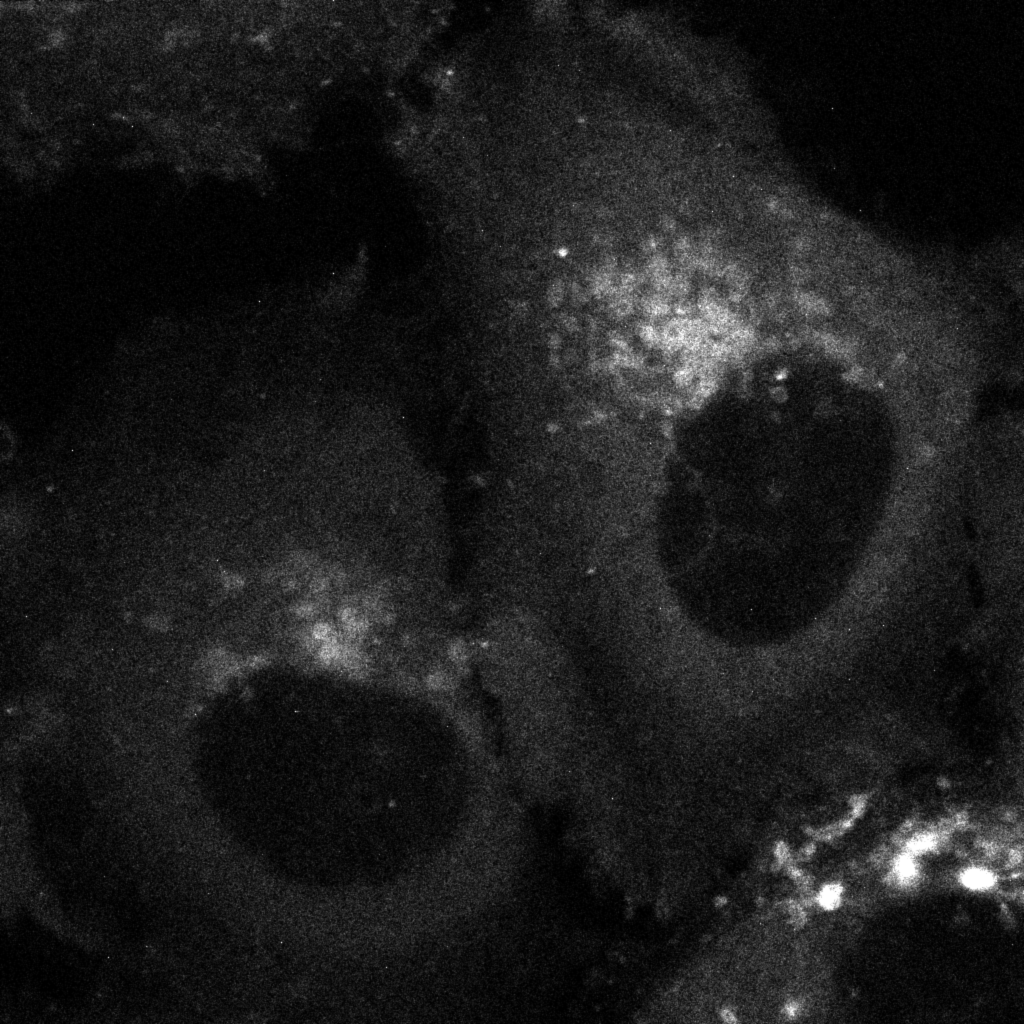

Supplement: Supplementary file 3 — Source Data for Figure 1 [file EMBR-24-e56841-s001.zip › Figure_1/1D/ML-SA1_1h_TECPR1.tif]

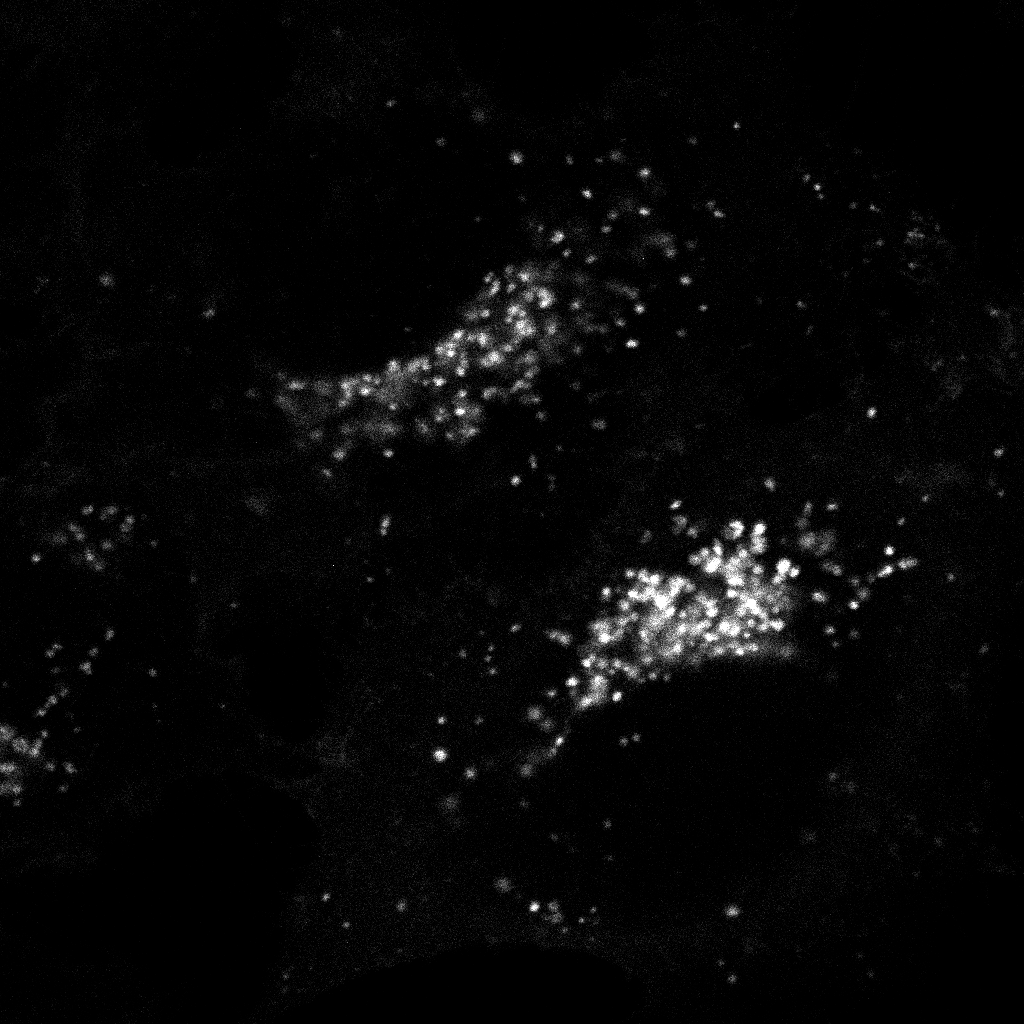

Supplement: Supplementary file 3 — Source Data for Figure 1 [file EMBR-24-e56841-s001.zip › Figure_1/1D/Monensin_0h_LAMP.tif]

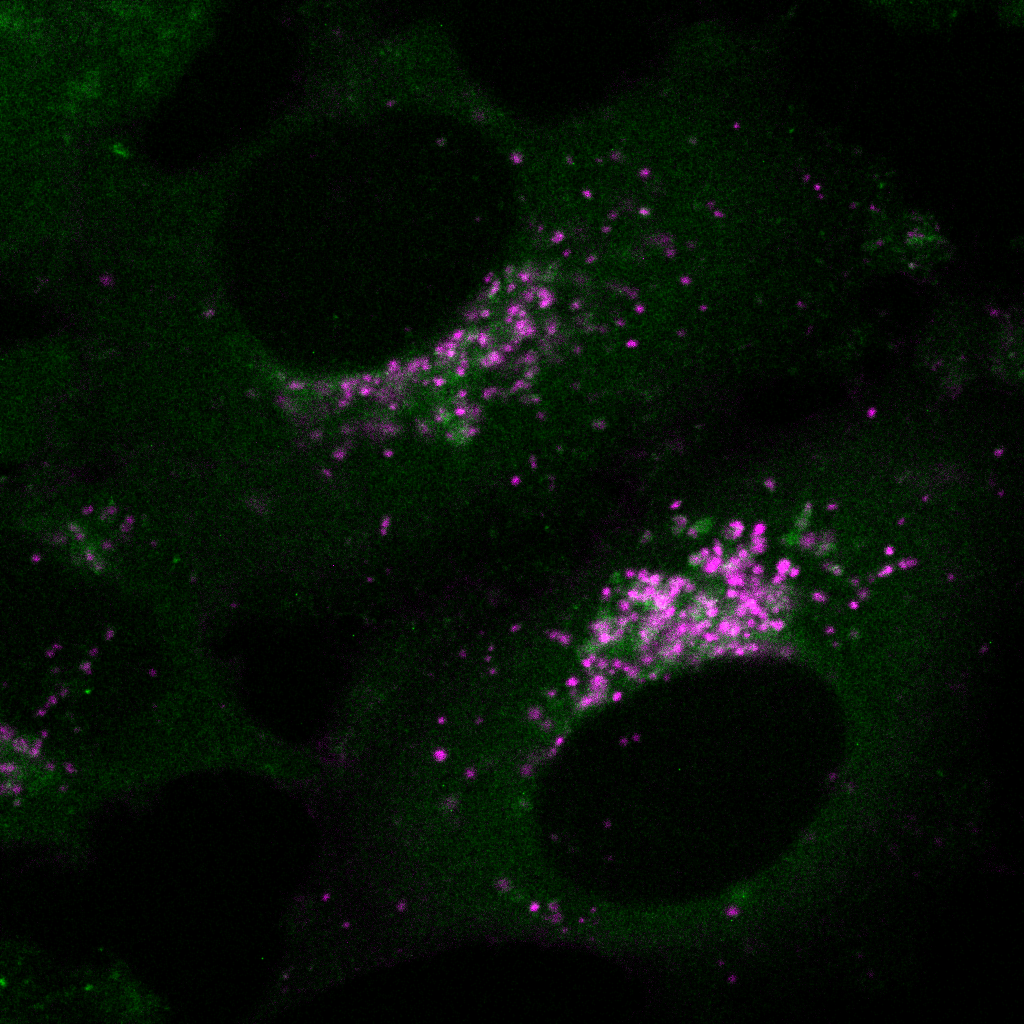

Supplement: Supplementary file 3 — Source Data for Figure 1 [file EMBR-24-e56841-s001.zip › Figure_1/1D/Monensin_0h_merge.tif]

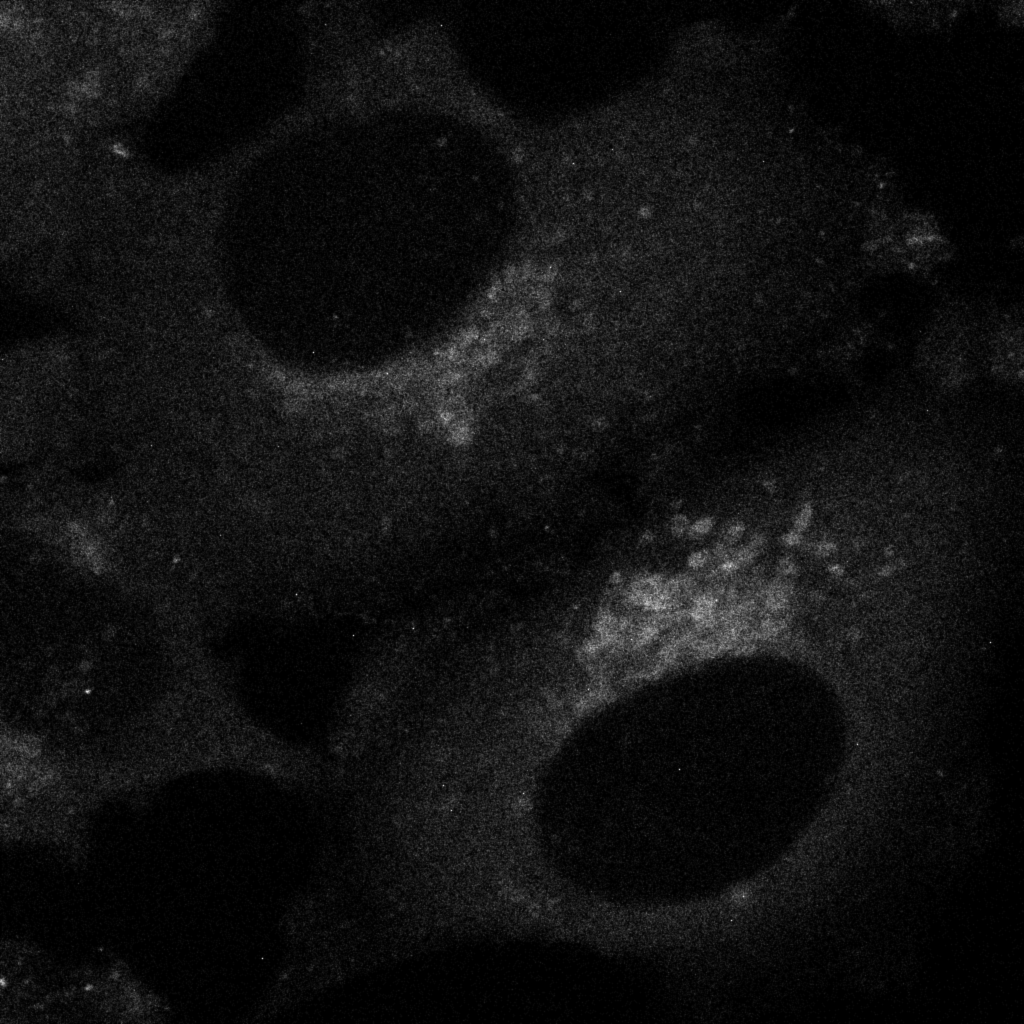

Supplement: Supplementary file 3 — Source Data for Figure 1 [file EMBR-24-e56841-s001.zip › Figure_1/1D/Monensin_0h_TECPR1.tif]

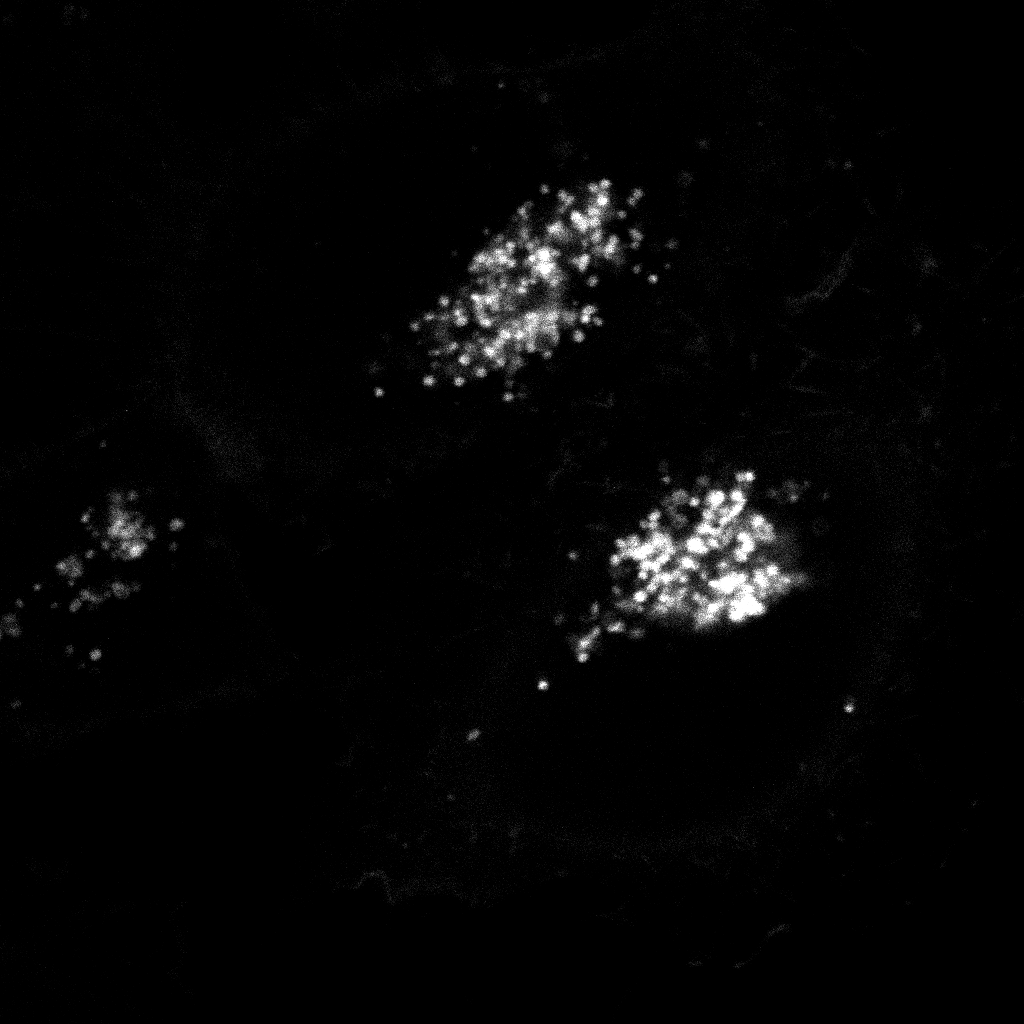

Supplement: Supplementary file 3 — Source Data for Figure 1 [file EMBR-24-e56841-s001.zip › Figure_1/1D/Monensin_1h_LAMP.tif]

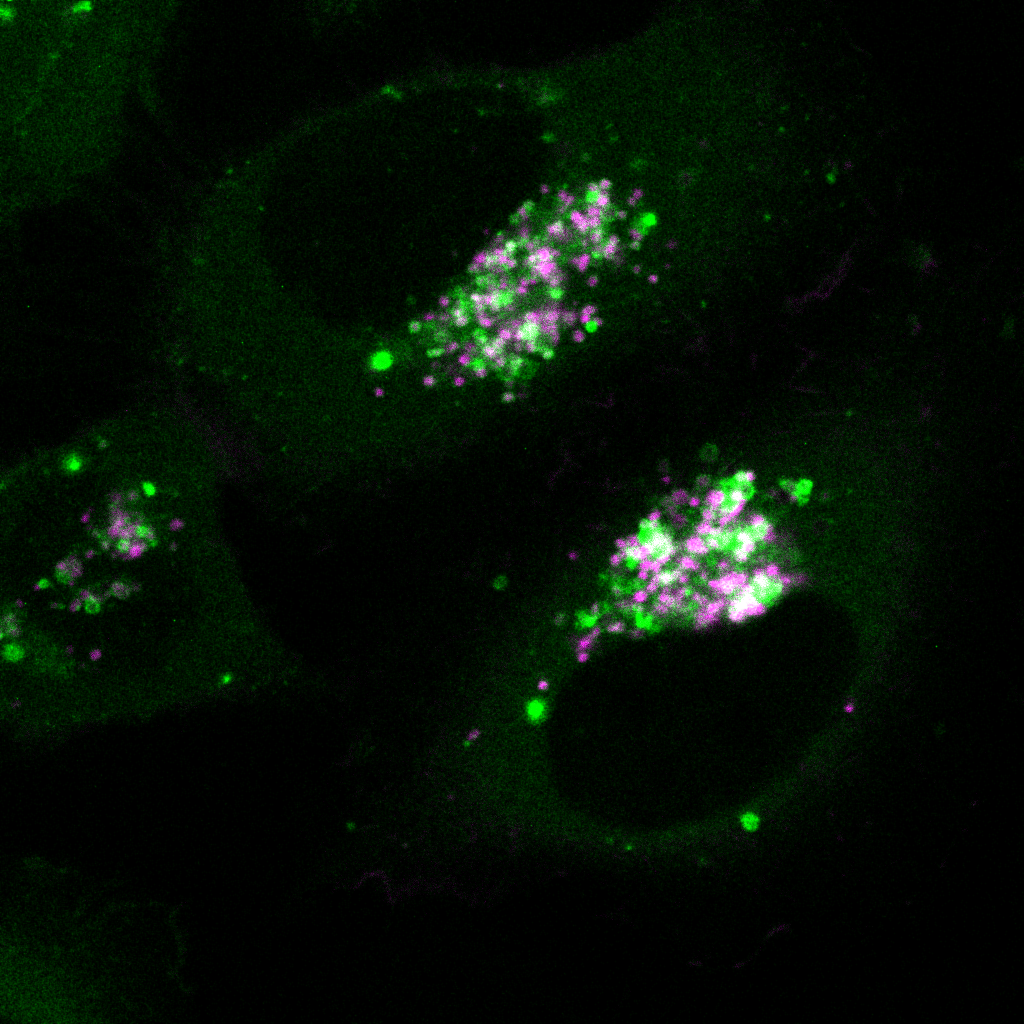

Supplement: Supplementary file 3 — Source Data for Figure 1 [file EMBR-24-e56841-s001.zip › Figure_1/1D/Monensin_1h_merge.tif]

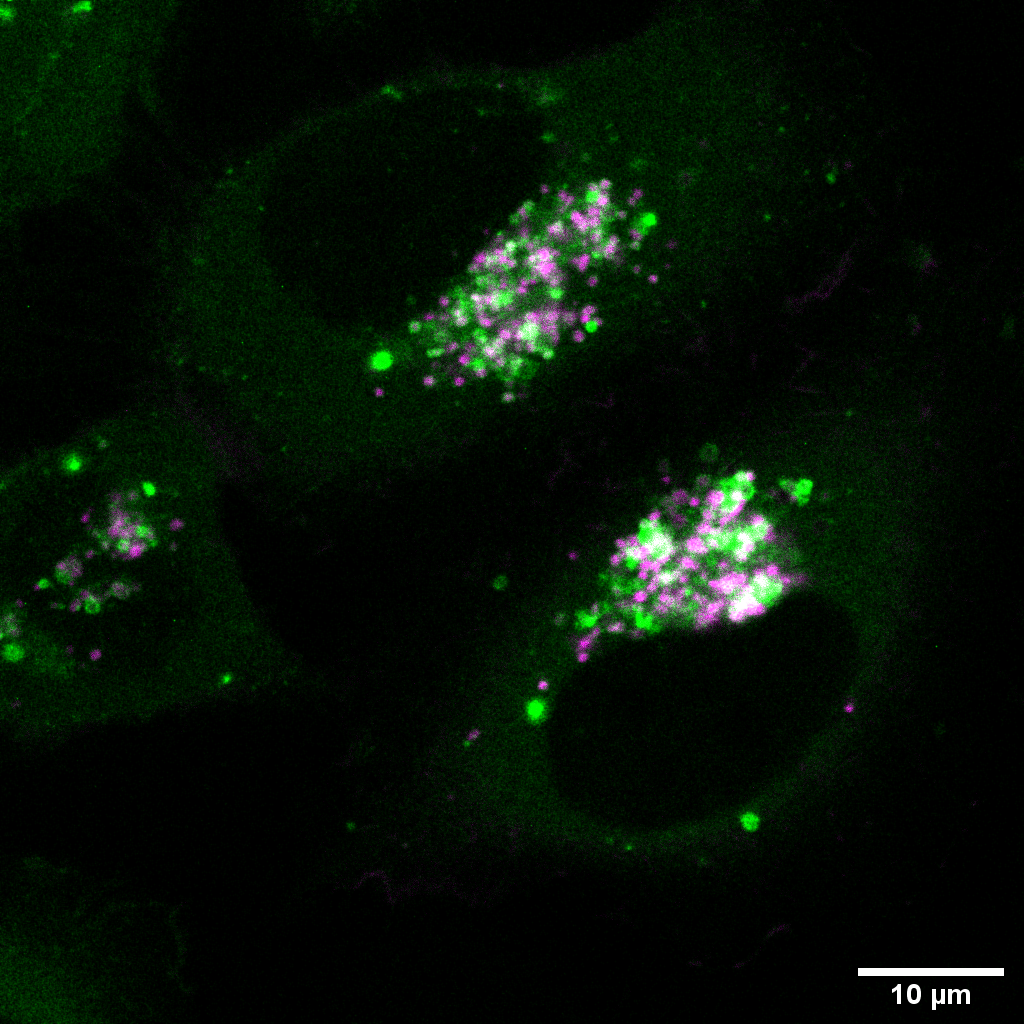

Supplement: Supplementary file 3 — Source Data for Figure 1 [file EMBR-24-e56841-s001.zip › Figure_1/1D/Monensin_1h_scale.tif]

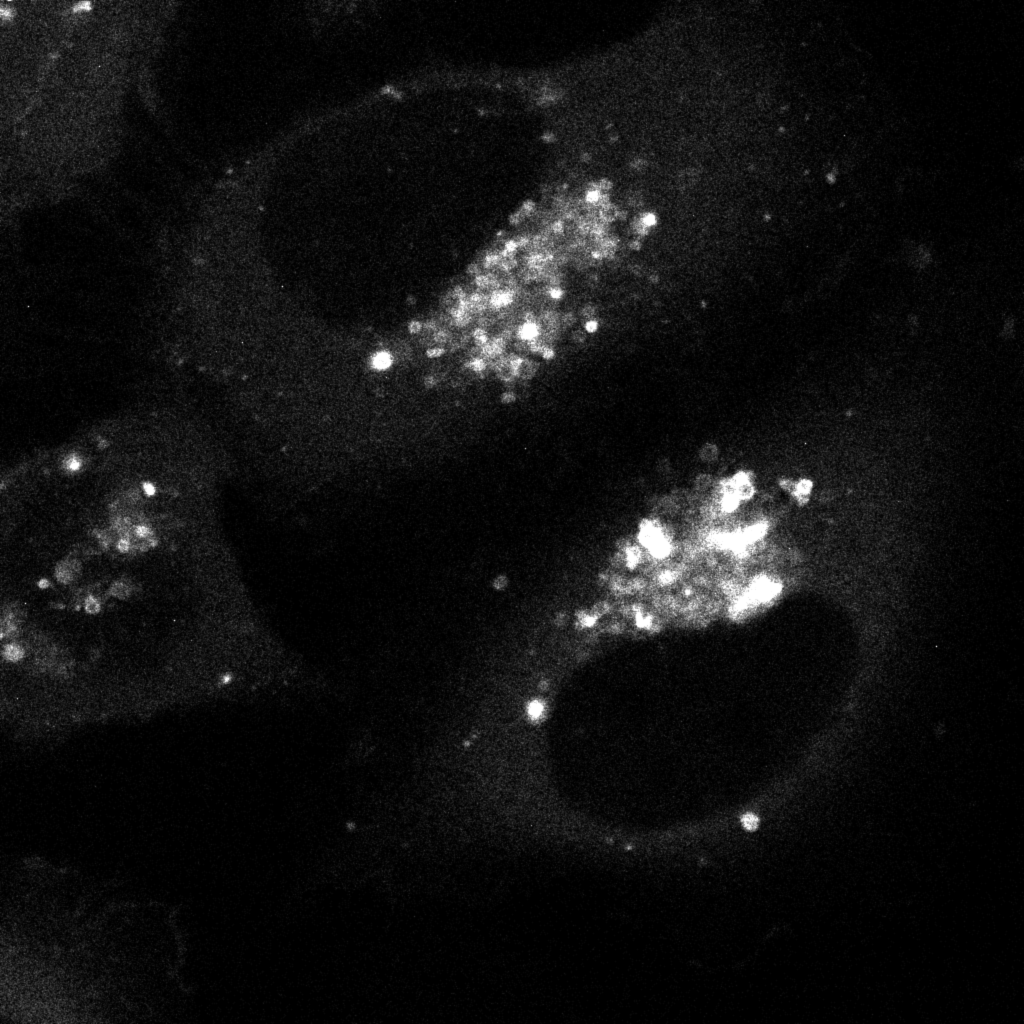

Supplement: Supplementary file 3 — Source Data for Figure 1 [file EMBR-24-e56841-s001.zip › Figure_1/1D/Monensin_1h_TECPR1.tif]

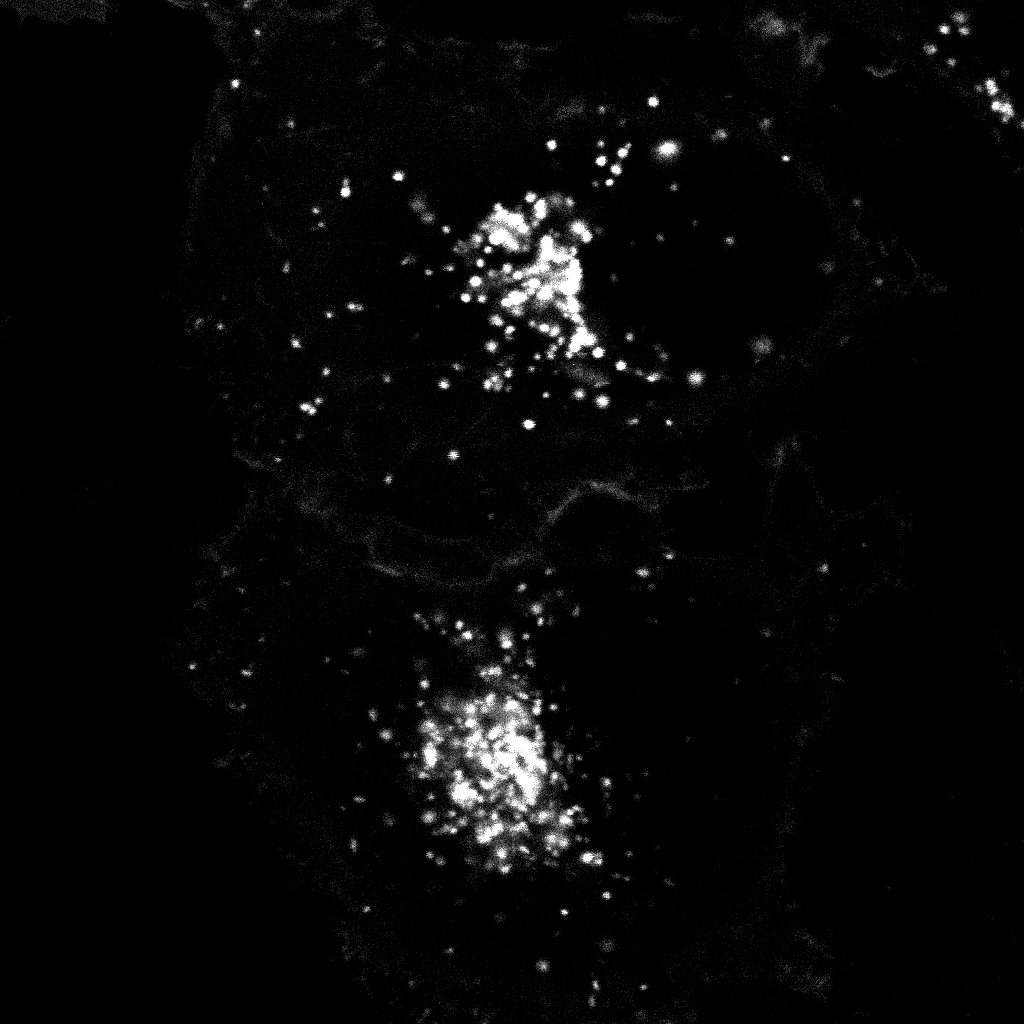

Supplement: Supplementary file 3 — Source Data for Figure 1 [file EMBR-24-e56841-s001.zip › Figure_1/1D/Nigericin_0h_LAMP.tif]

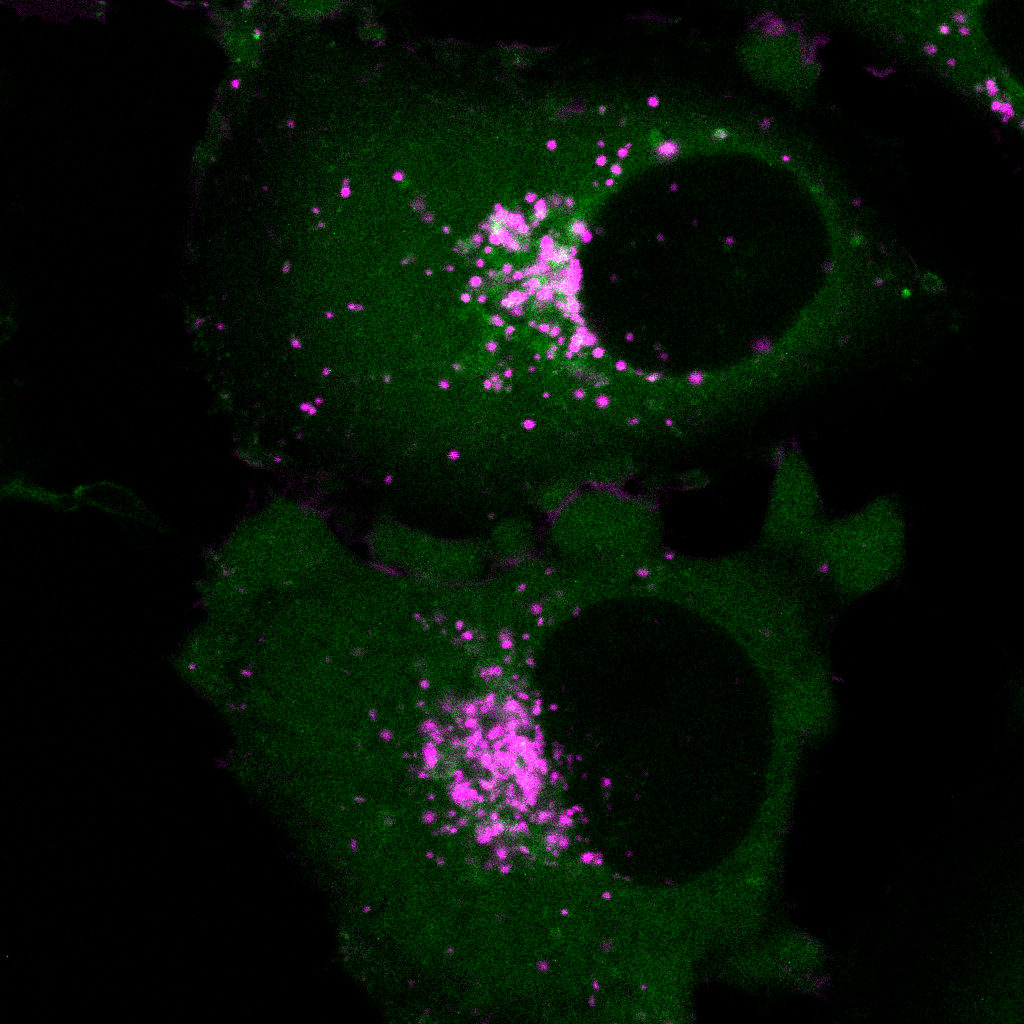

Supplement: Supplementary file 3 — Source Data for Figure 1 [file EMBR-24-e56841-s001.zip › Figure_1/1D/Nigericin_0h_merge.tif]

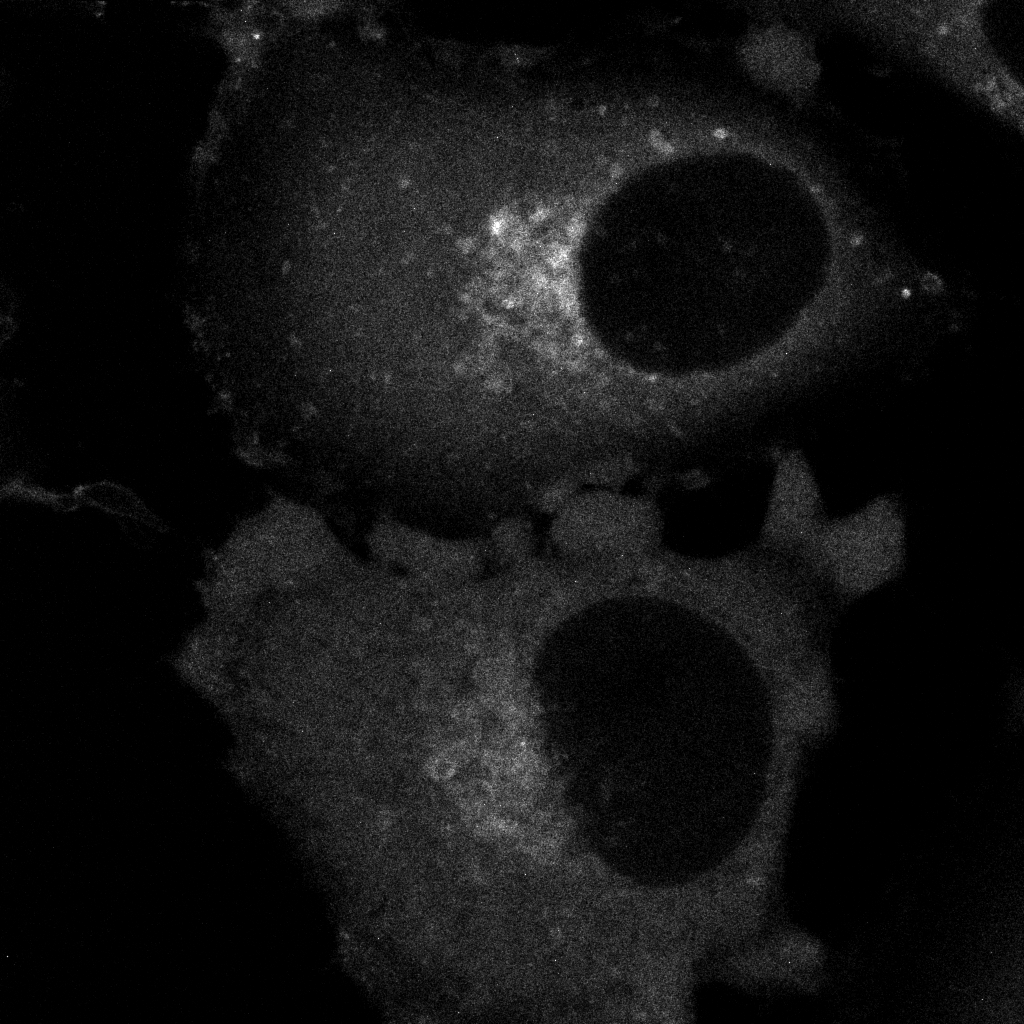

Supplement: Supplementary file 3 — Source Data for Figure 1 [file EMBR-24-e56841-s001.zip › Figure_1/1D/Nigericin_0h_TECPR1.tif]

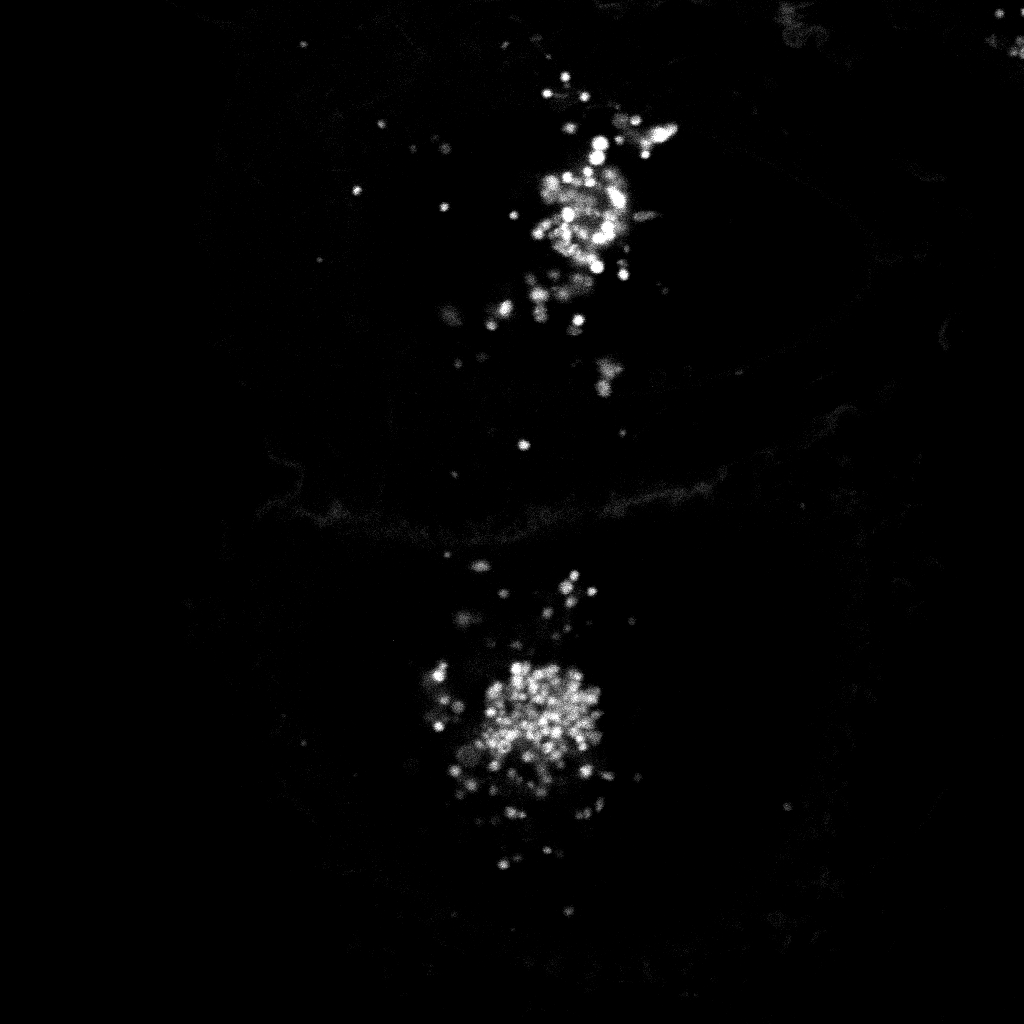

Supplement: Supplementary file 3 — Source Data for Figure 1 [file EMBR-24-e56841-s001.zip › Figure_1/1D/Nigericin_1h_LAMP.tif]

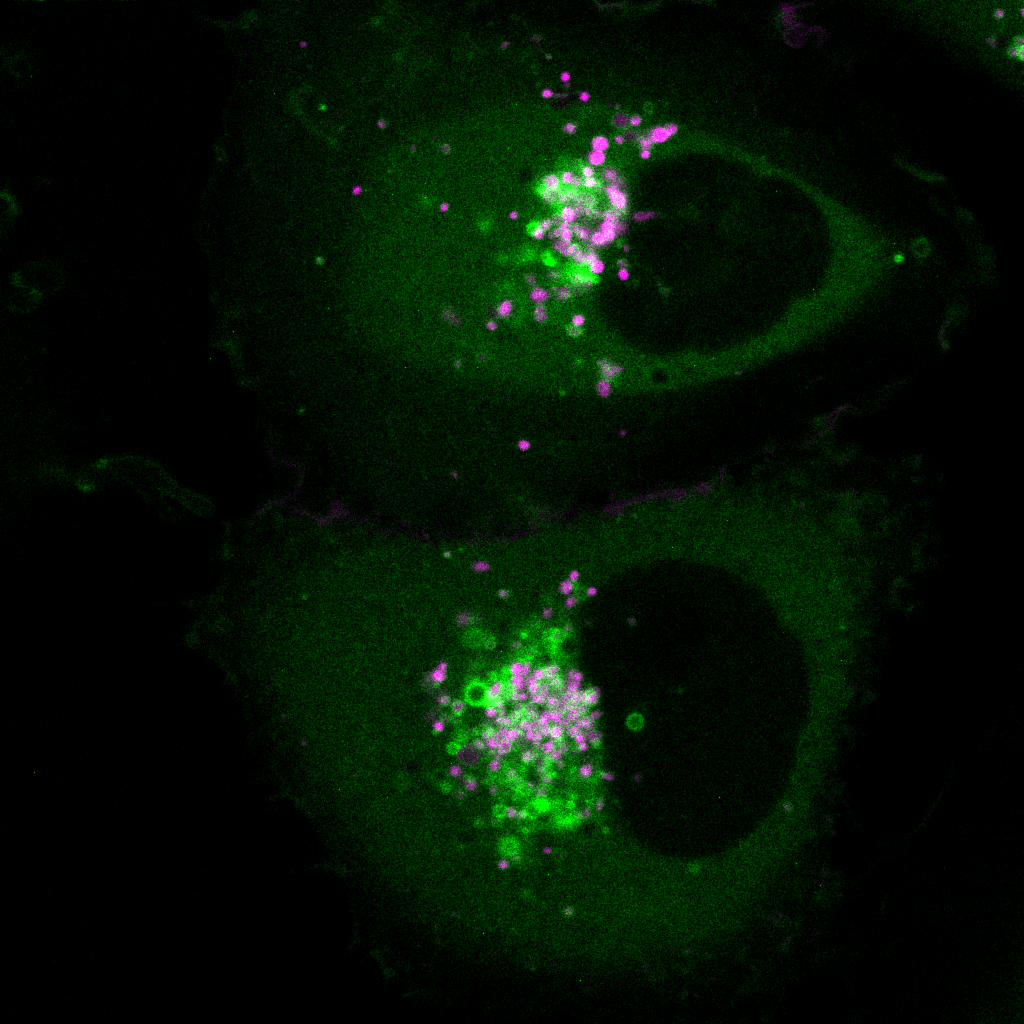

Supplement: Supplementary file 3 — Source Data for Figure 1 [file EMBR-24-e56841-s001.zip › Figure_1/1D/Nigericin_1h_merge.tif]

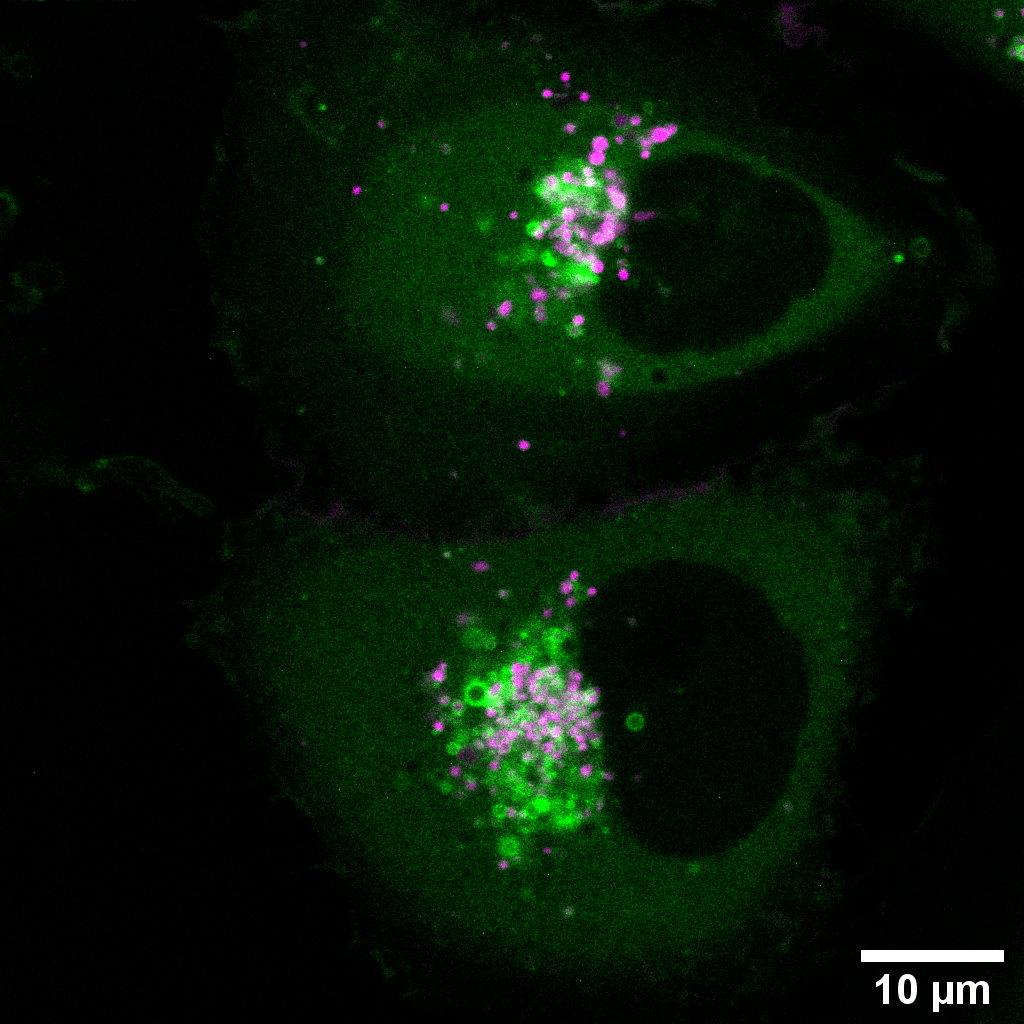

Supplement: Supplementary file 3 — Source Data for Figure 1 [file EMBR-24-e56841-s001.zip › Figure_1/1D/Nigericin_1h_scale.tif]

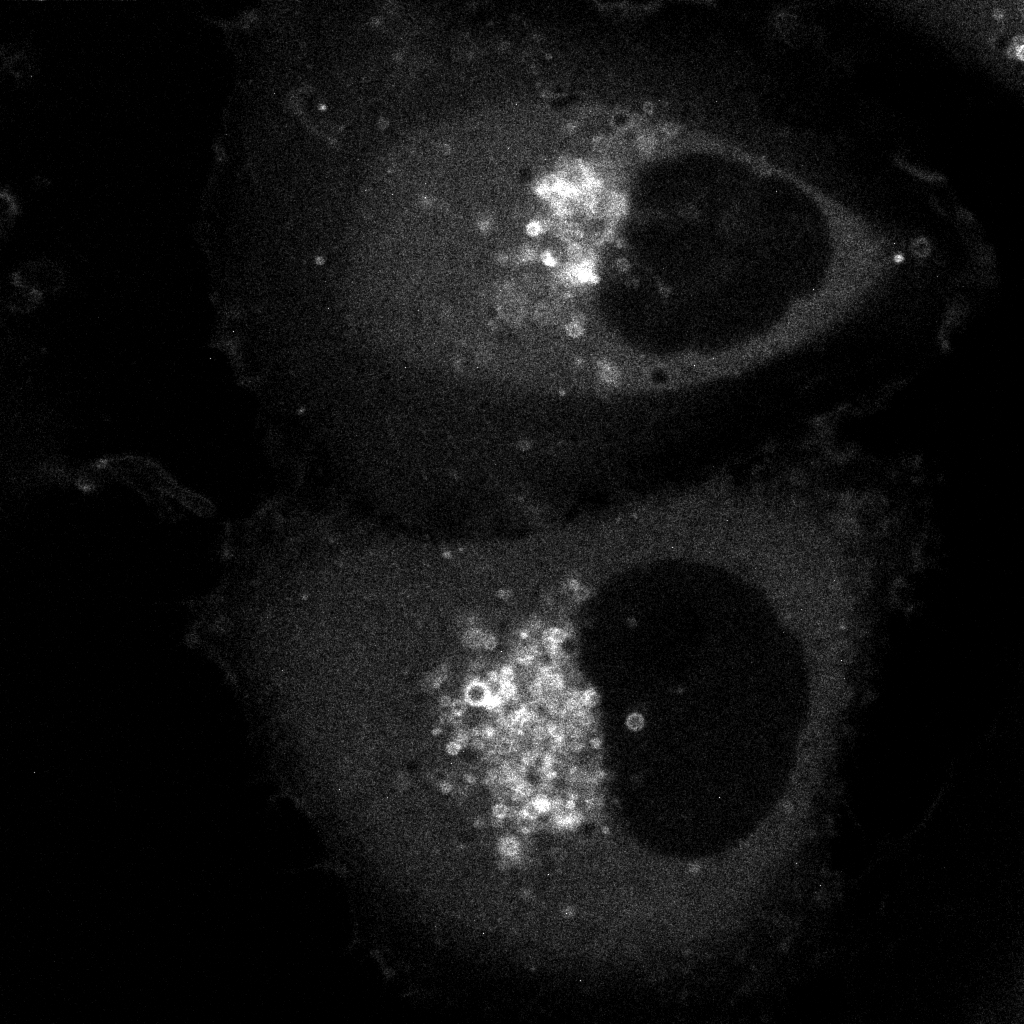

Supplement: Supplementary file 3 — Source Data for Figure 1 [file EMBR-24-e56841-s001.zip › Figure_1/1D/Nigericin_1h_TECPR1.tif]

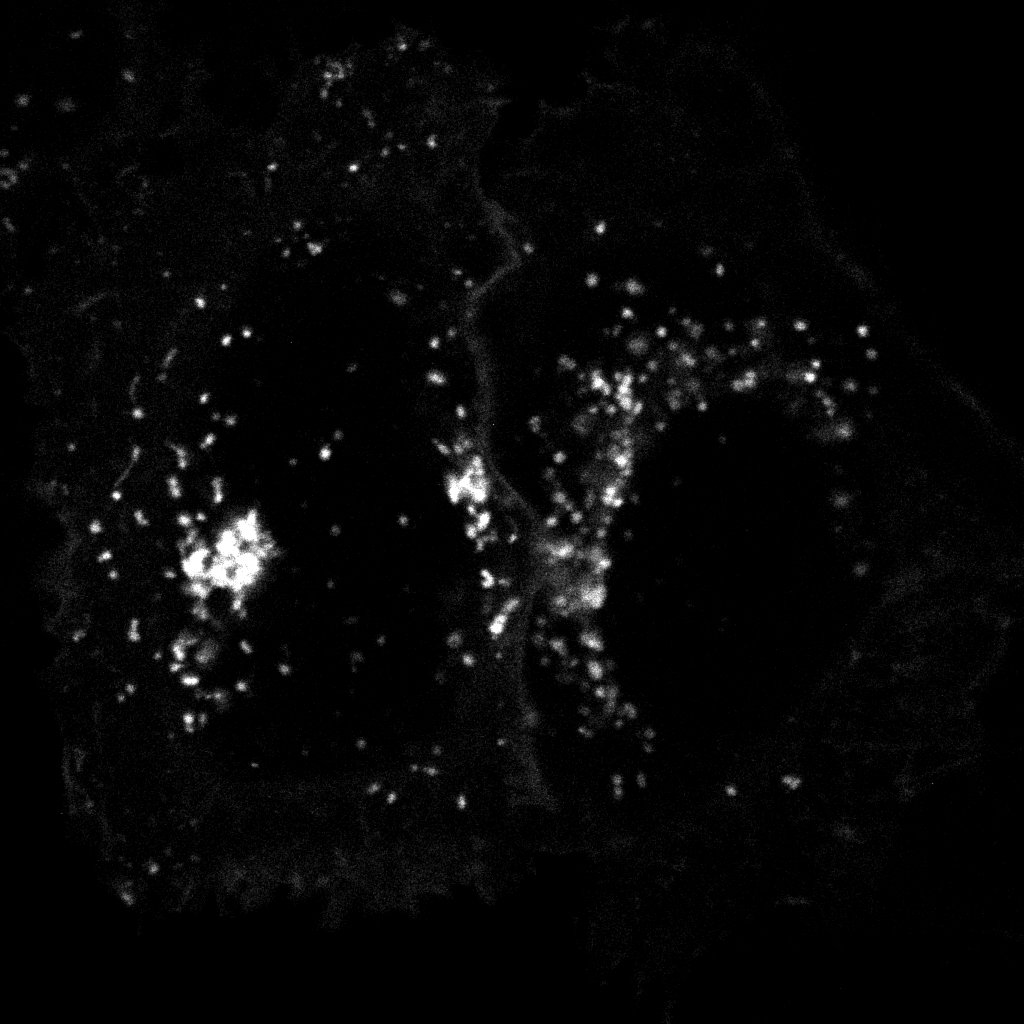

Supplement: Supplementary file 3 — Source Data for Figure 1 [file EMBR-24-e56841-s001.zip › Figure_1/1D/VEH_0h_LAMP.tif]

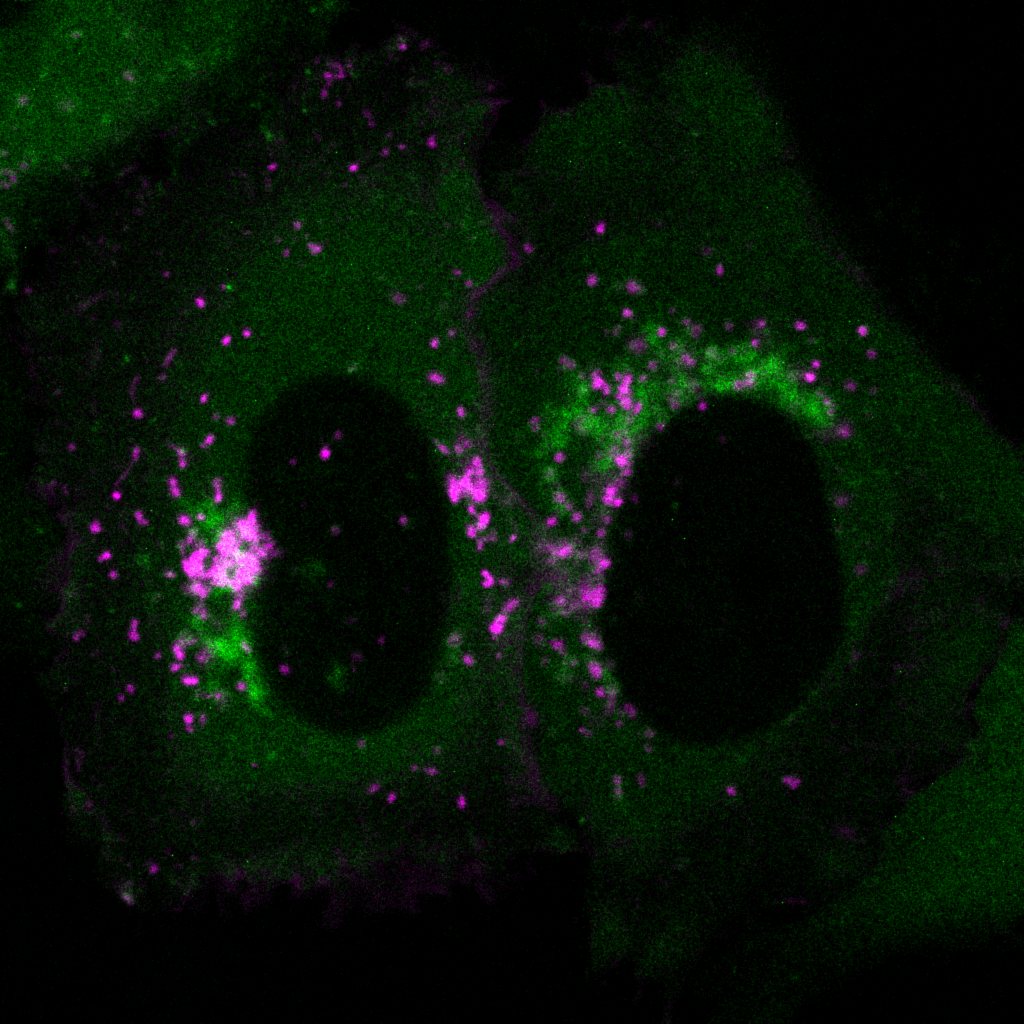

Supplement: Supplementary file 3 — Source Data for Figure 1 [file EMBR-24-e56841-s001.zip › Figure_1/1D/VEH_0h_merge.tif]

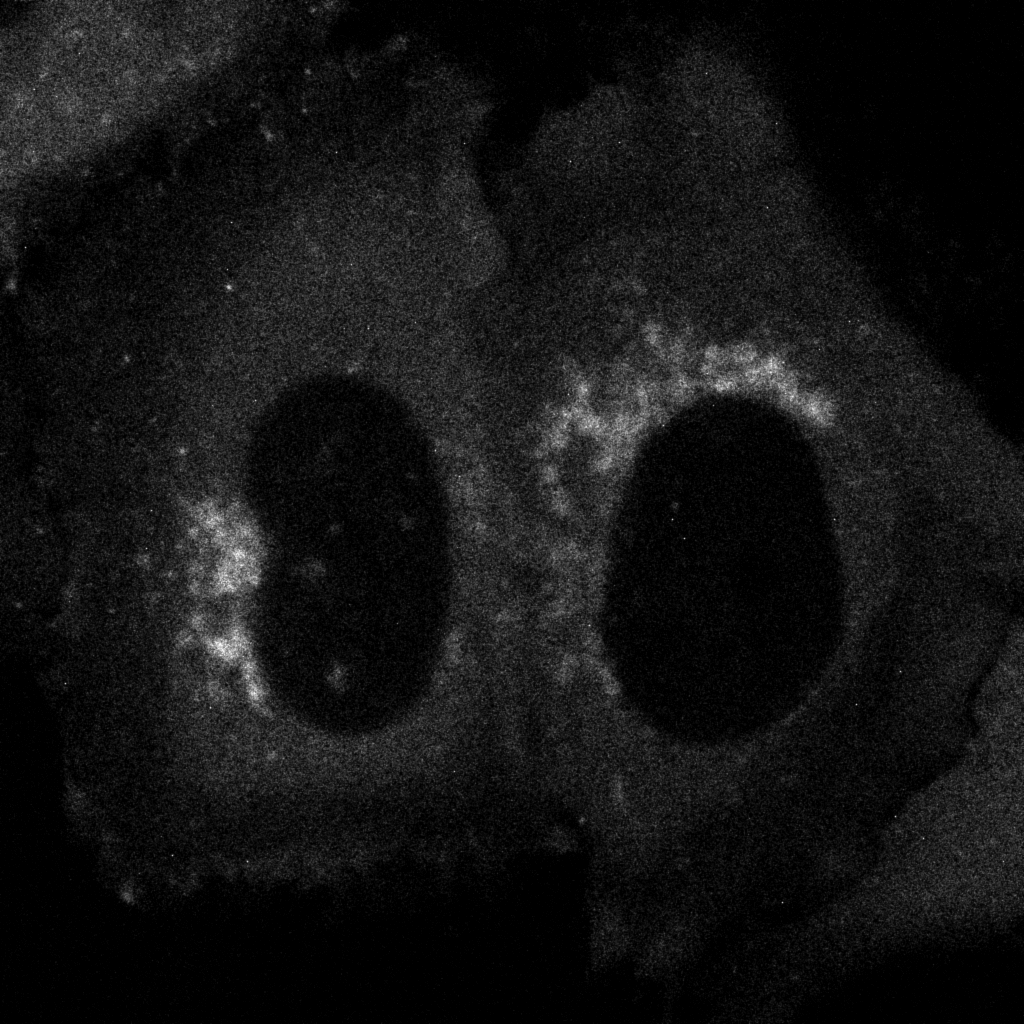

Supplement: Supplementary file 3 — Source Data for Figure 1 [file EMBR-24-e56841-s001.zip › Figure_1/1D/VEH_0h_TECPR1.tif]

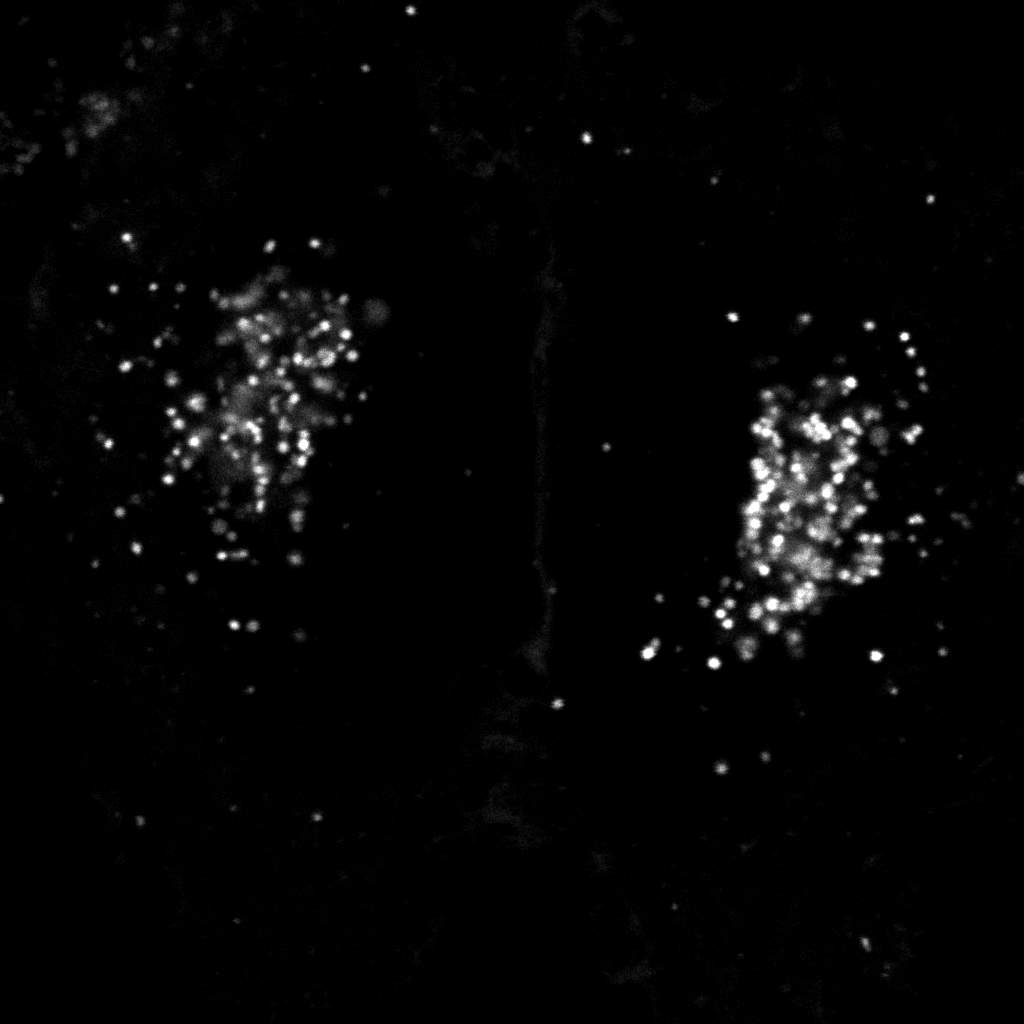

Supplement: Supplementary file 3 — Source Data for Figure 1 [file EMBR-24-e56841-s001.zip › Figure_1/1D/VEH_0min_LAMP.tif]

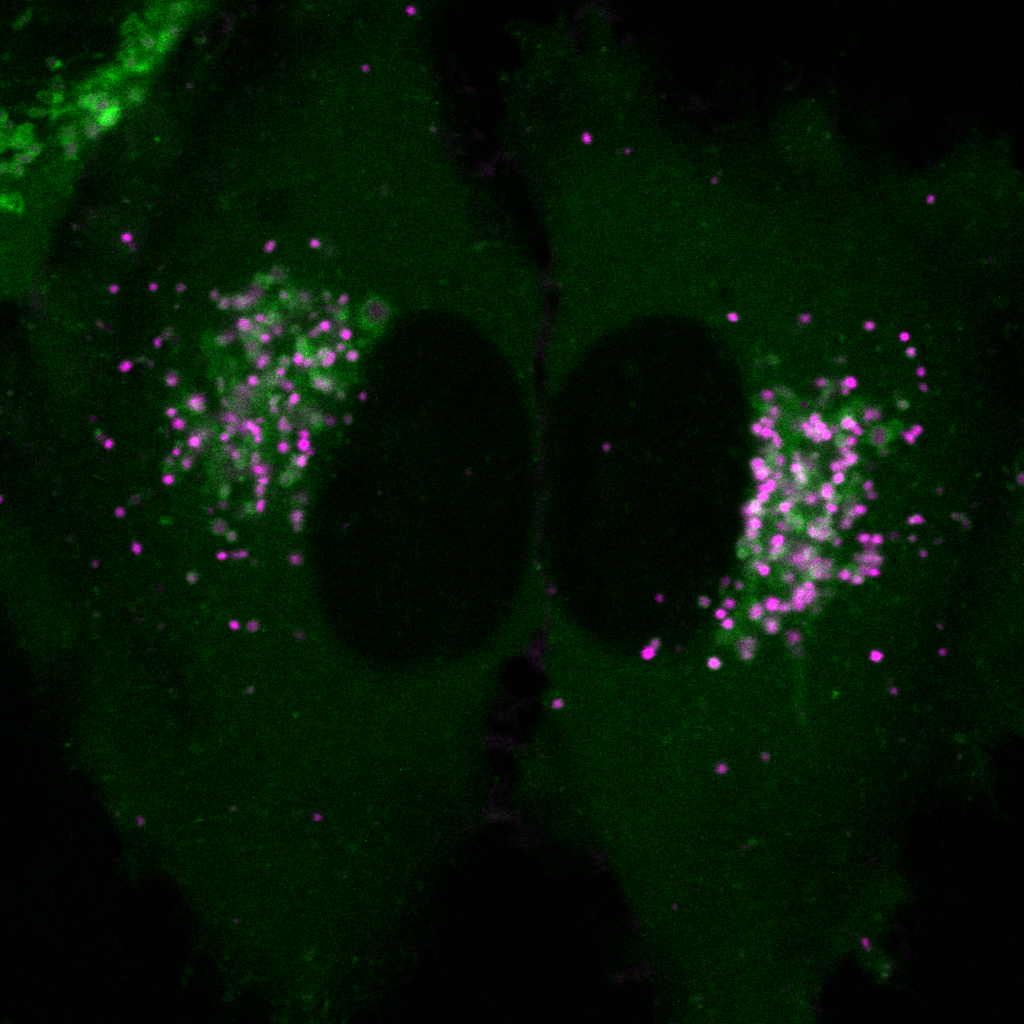

Supplement: Supplementary file 3 — Source Data for Figure 1 [file EMBR-24-e56841-s001.zip › Figure_1/1D/VEH_0min_Merge.tif]

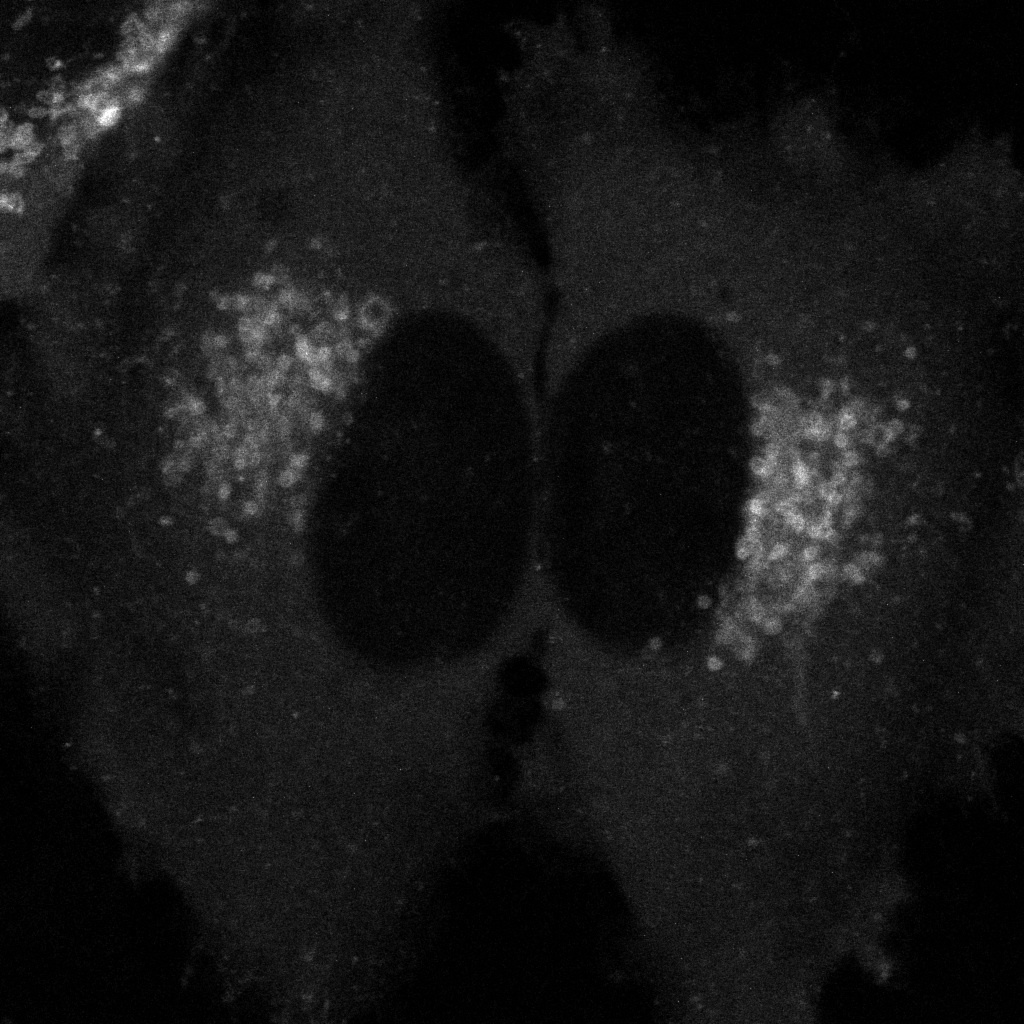

Supplement: Supplementary file 3 — Source Data for Figure 1 [file EMBR-24-e56841-s001.zip › Figure_1/1D/VEH_0min_TECPR1.tif]

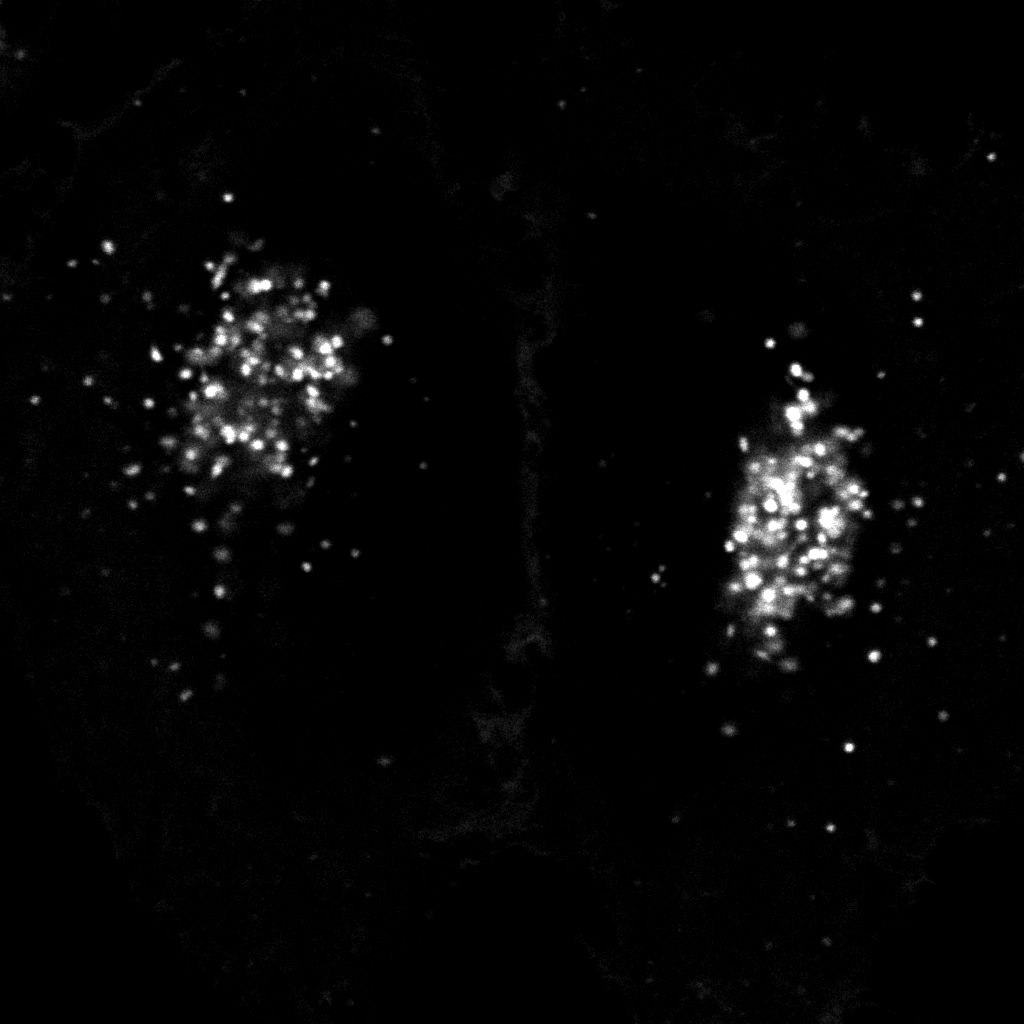

Supplement: Supplementary file 3 — Source Data for Figure 1 [file EMBR-24-e56841-s001.zip › Figure_1/1D/VEH_15min_LAMP.tif]

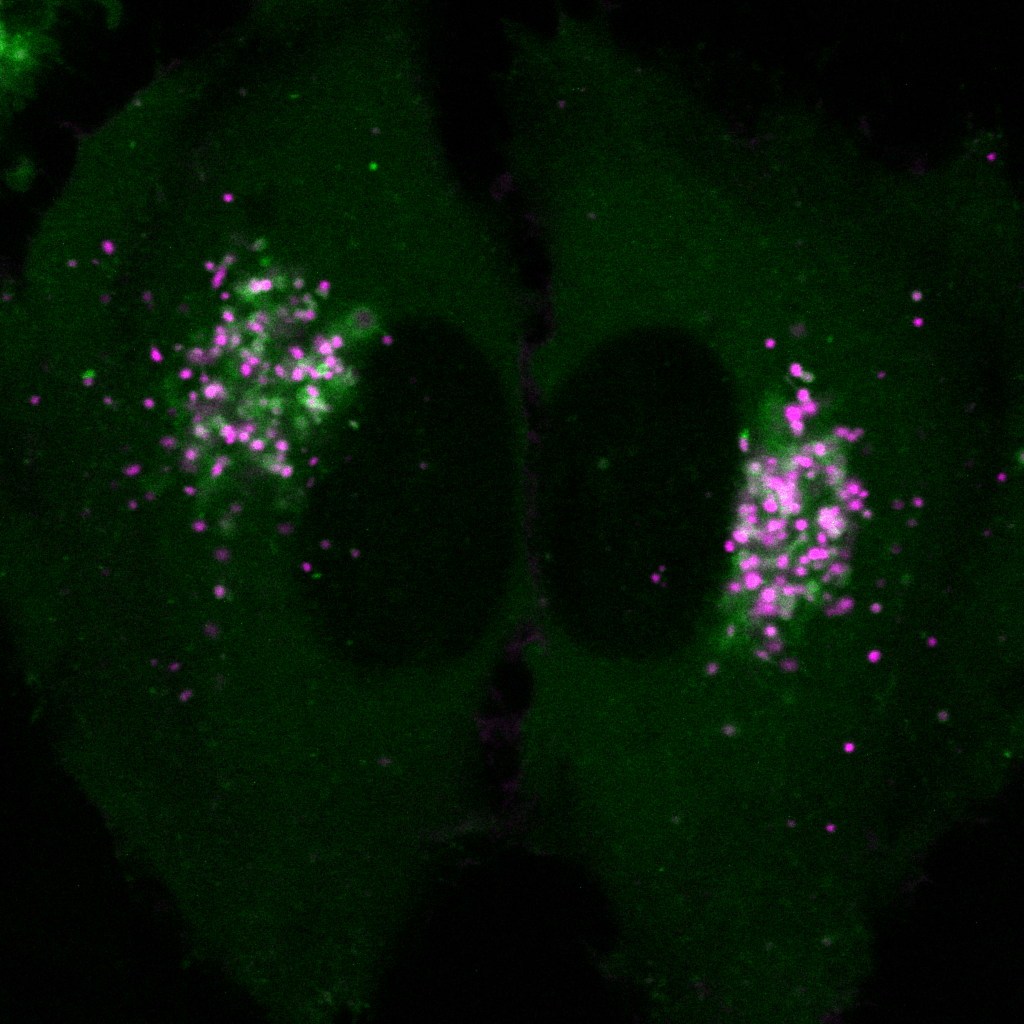

Supplement: Supplementary file 3 — Source Data for Figure 1 [file EMBR-24-e56841-s001.zip › Figure_1/1D/VEH_15min_Merge.tif]

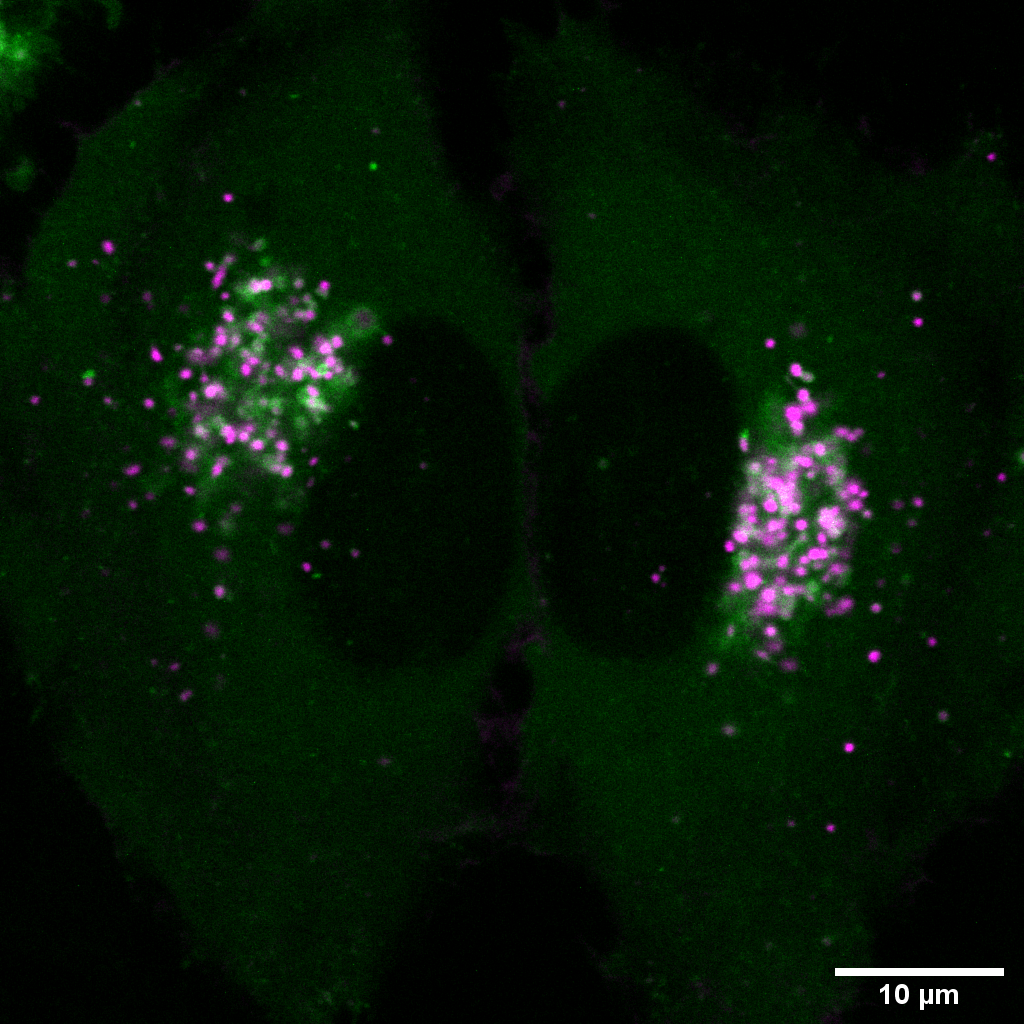

Supplement: Supplementary file 3 — Source Data for Figure 1 [file EMBR-24-e56841-s001.zip › Figure_1/1D/VEH_15min_scale.tif]

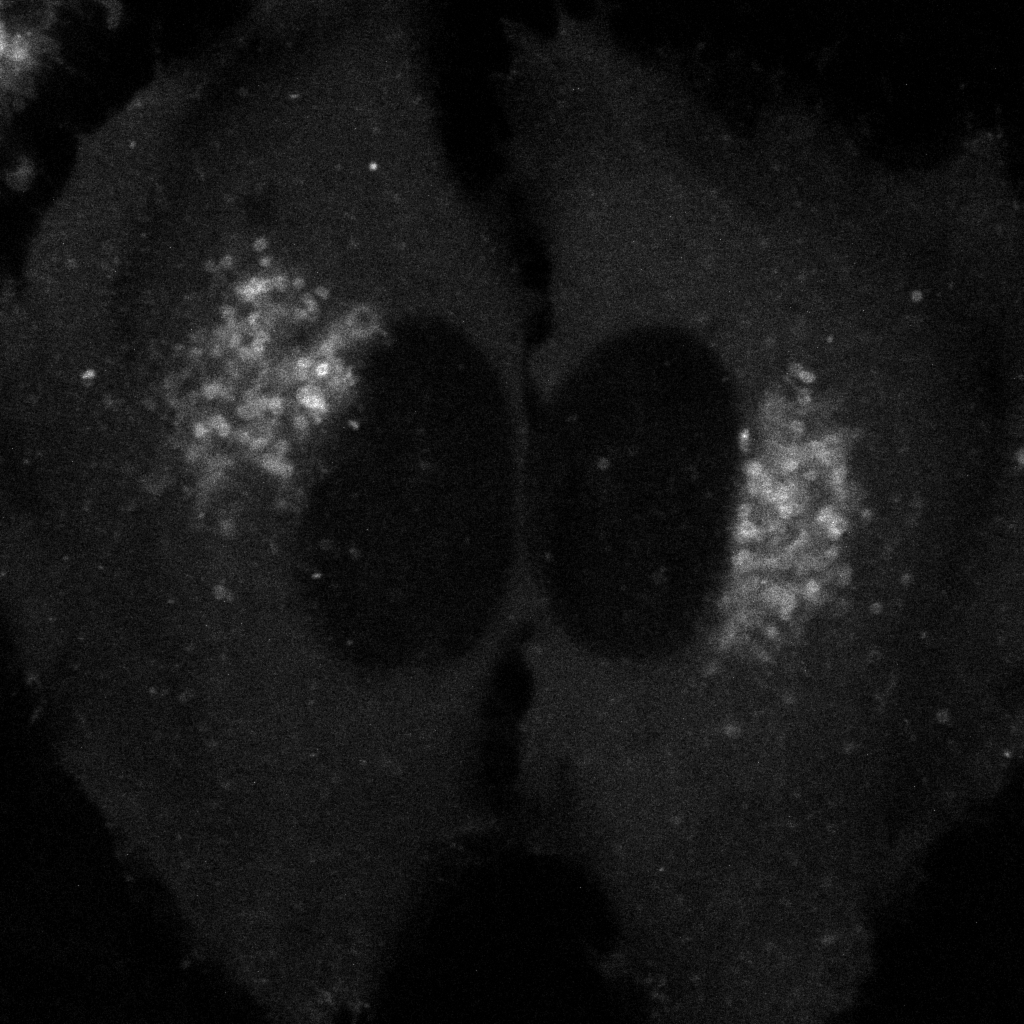

Supplement: Supplementary file 3 — Source Data for Figure 1 [file EMBR-24-e56841-s001.zip › Figure_1/1D/VEH_15min_TECPR1.tif]

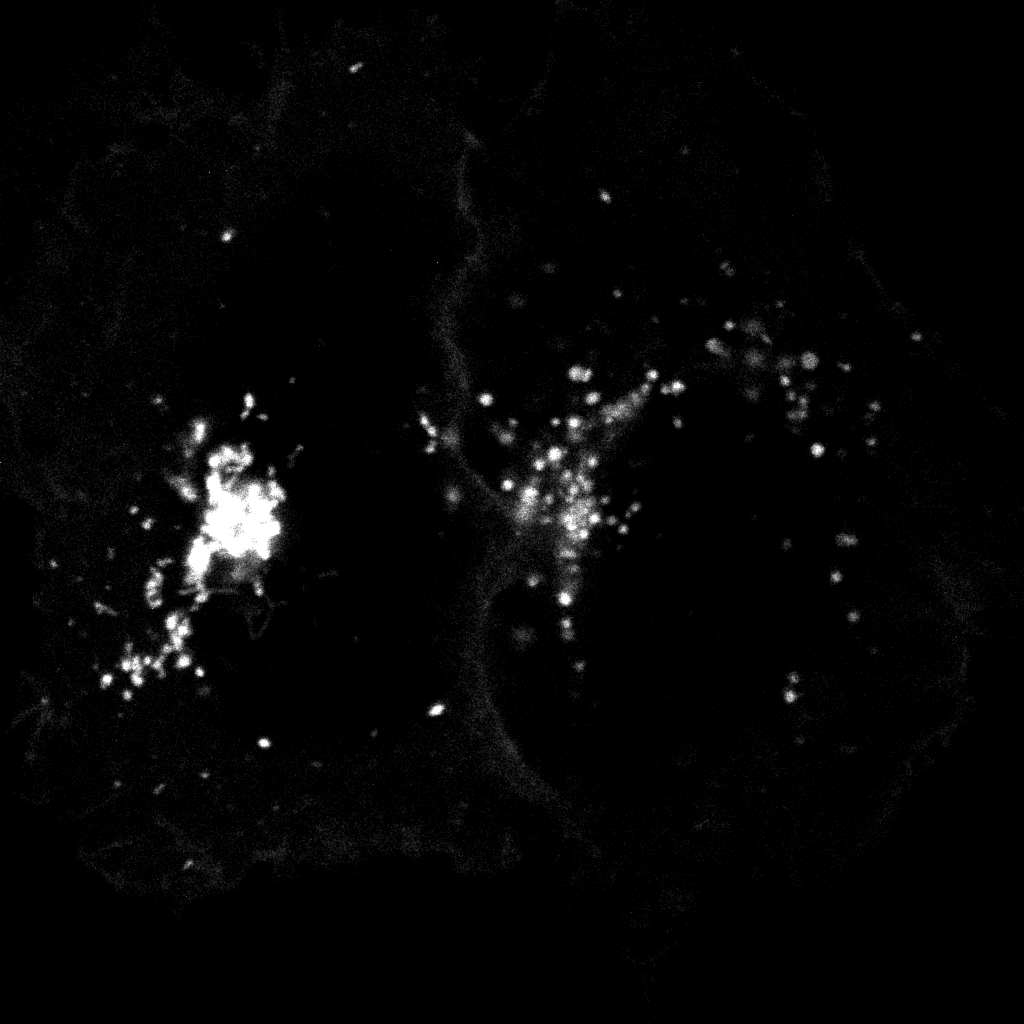

Supplement: Supplementary file 3 — Source Data for Figure 1 [file EMBR-24-e56841-s001.zip › Figure_1/1D/VEH_1h_LAMP.tif]

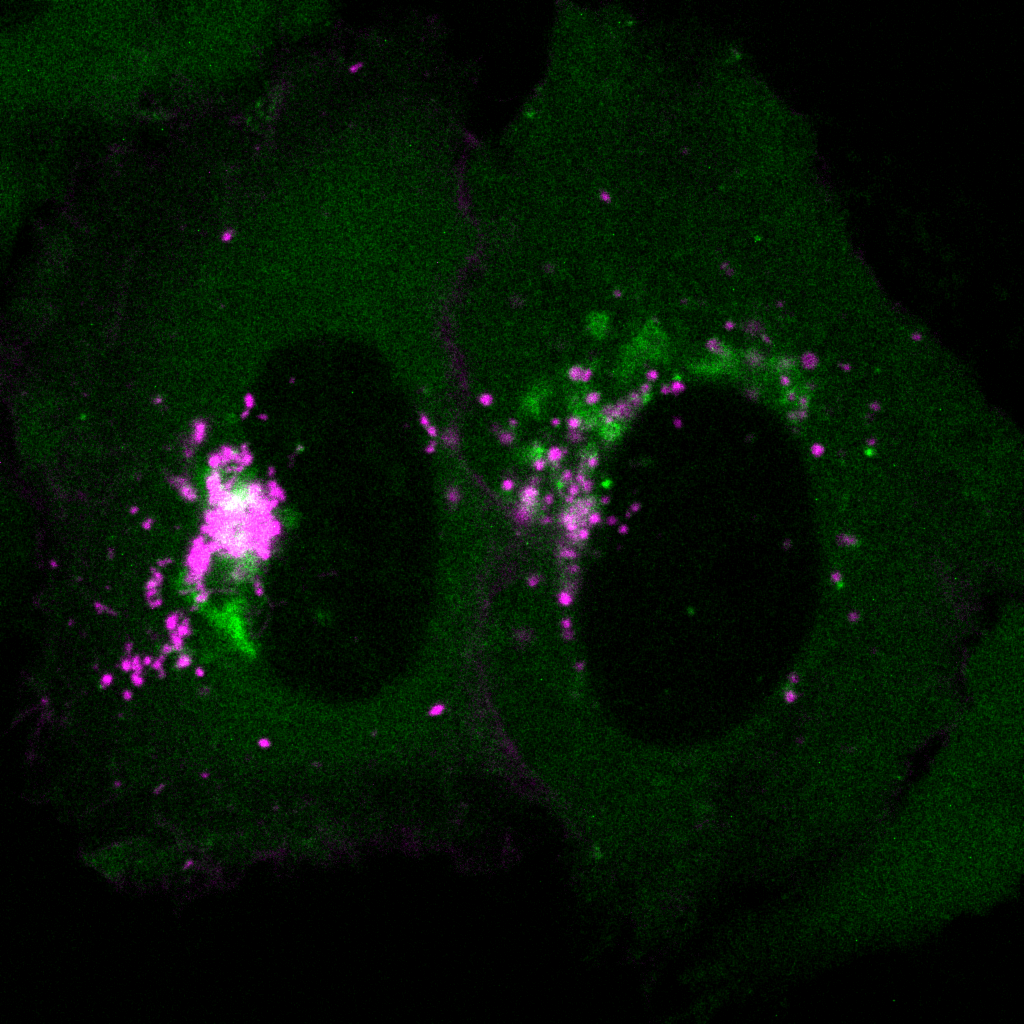

Supplement: Supplementary file 3 — Source Data for Figure 1 [file EMBR-24-e56841-s001.zip › Figure_1/1D/VEH_1h_merge.tif]

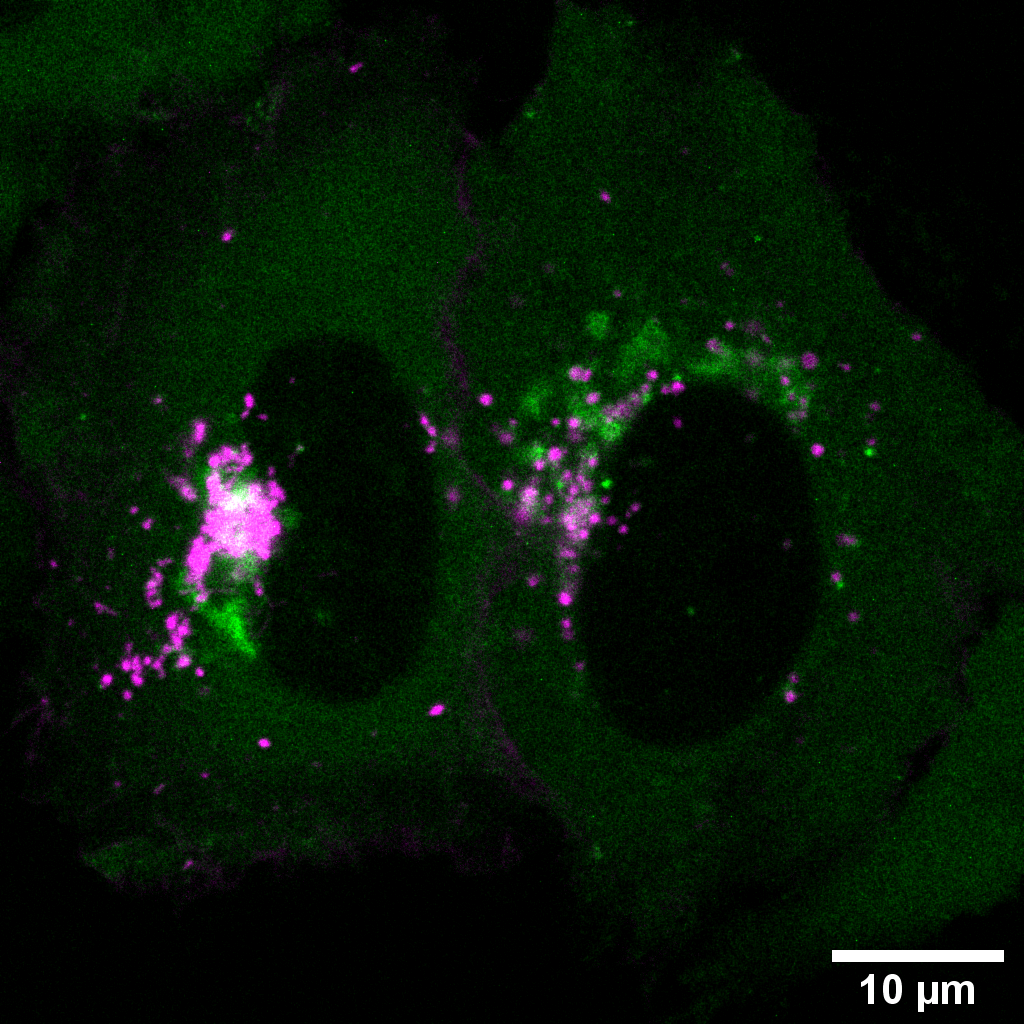

Supplement: Supplementary file 3 — Source Data for Figure 1 [file EMBR-24-e56841-s001.zip › Figure_1/1D/VEH_1h_scale.tif]

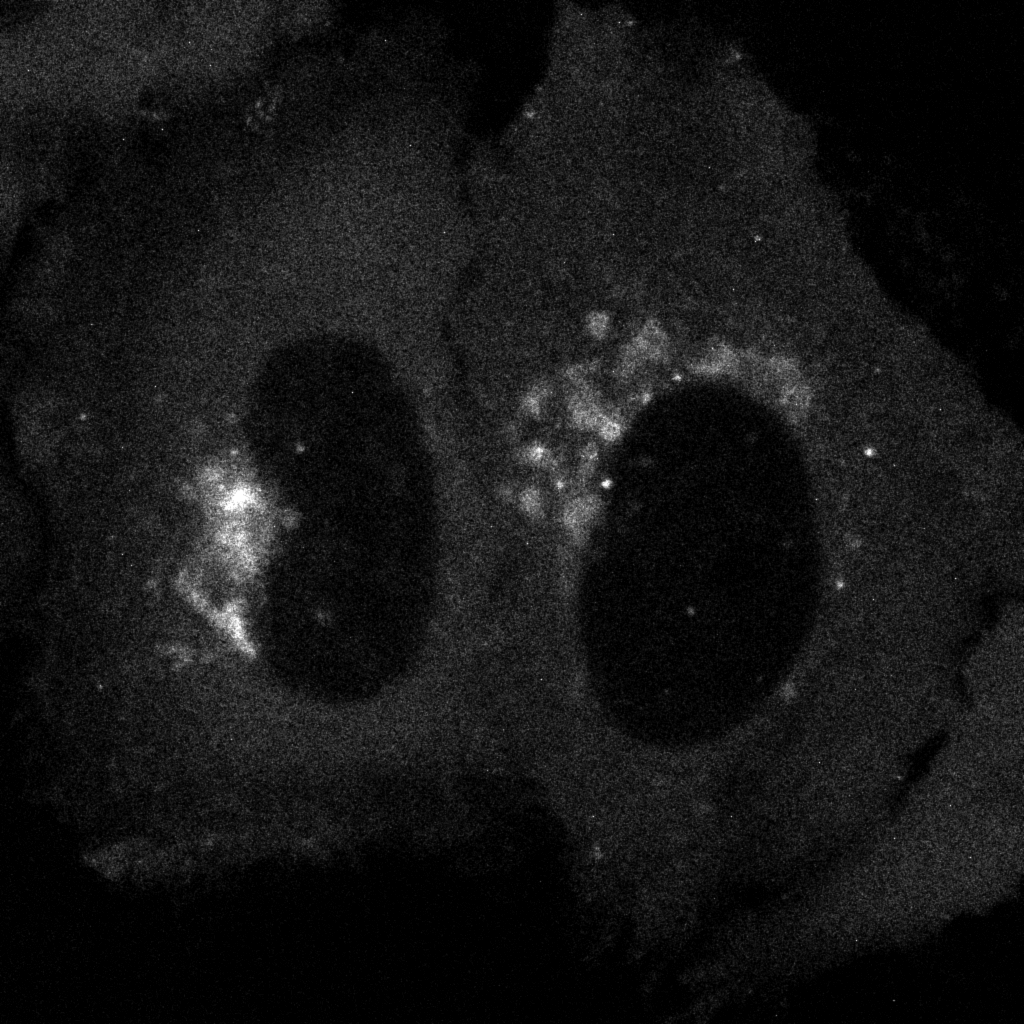

Supplement: Supplementary file 3 — Source Data for Figure 1 [file EMBR-24-e56841-s001.zip › Figure_1/1D/VEH_1h_TECPR1.tif]

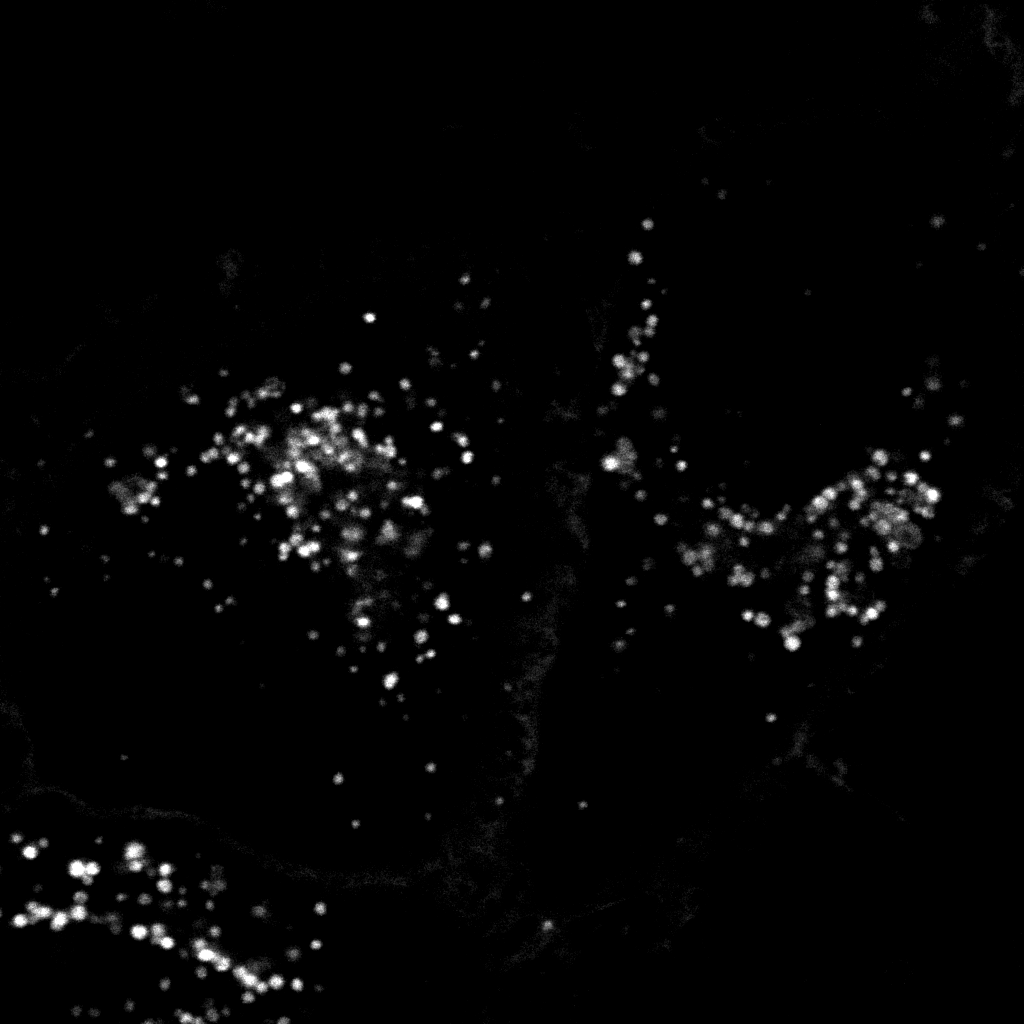

Supplement: Supplementary file 4 — Source Data for Figure 2 [file EMBR-24-e56841-s005.zip › Figure_2/2B/Image_Data/d1-170_0min_LAMP.tif]

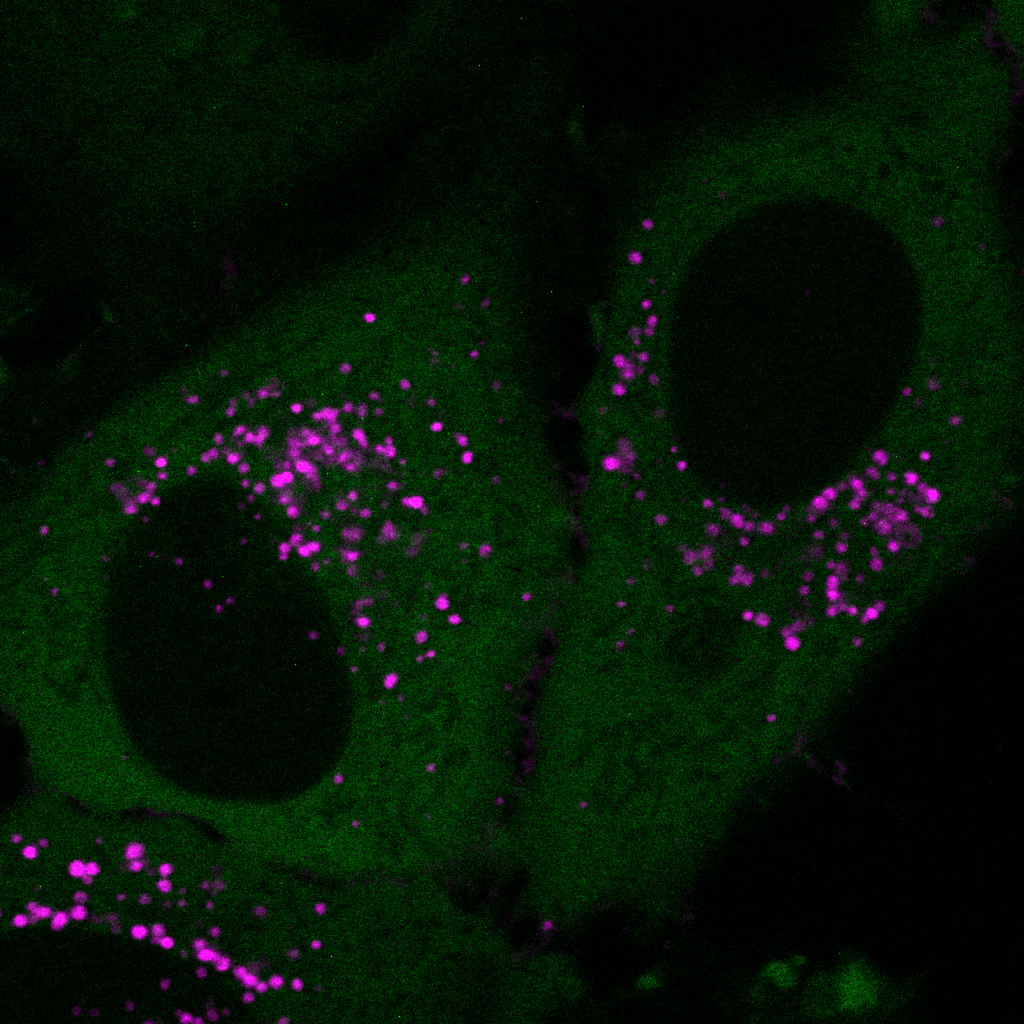

Supplement: Supplementary file 4 — Source Data for Figure 2 [file EMBR-24-e56841-s005.zip › Figure_2/2B/Image_Data/d1-170_0min_merge.tif]

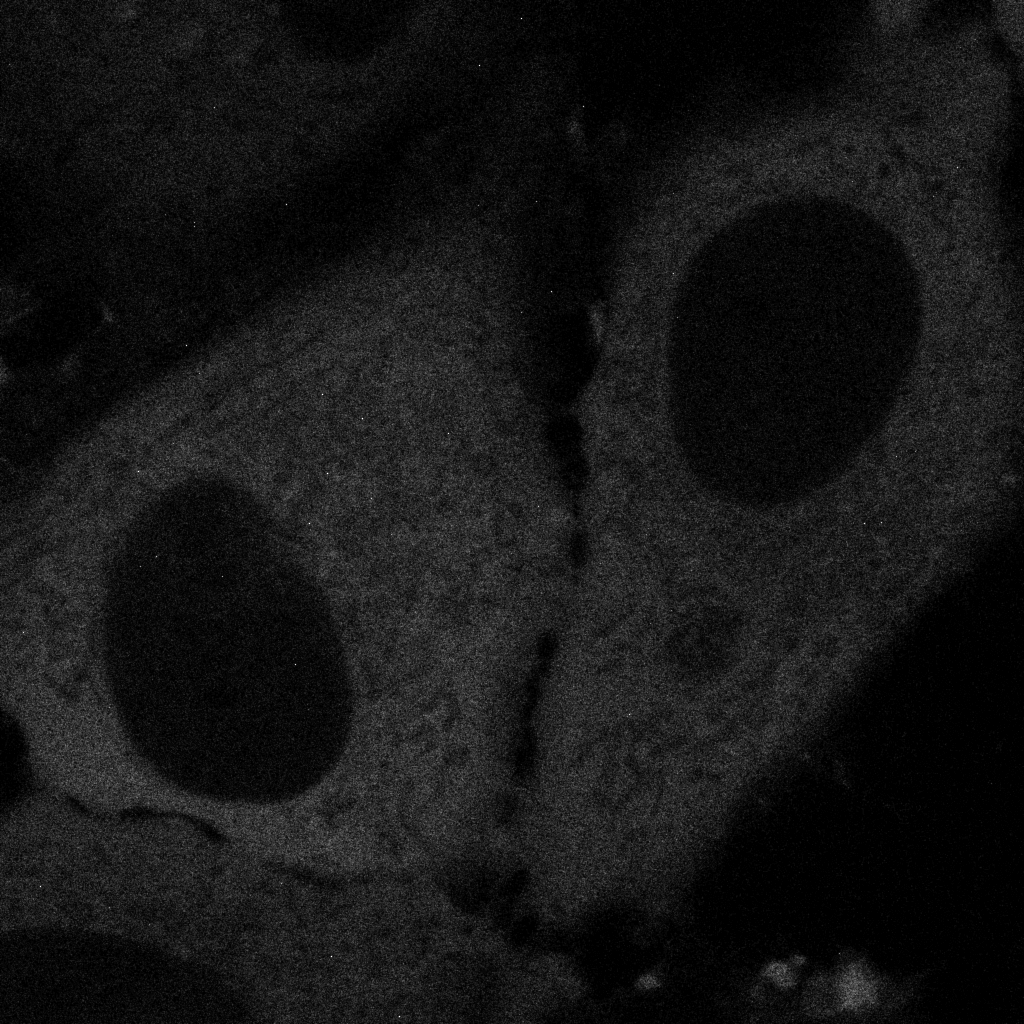

Supplement: Supplementary file 4 — Source Data for Figure 2 [file EMBR-24-e56841-s005.zip › Figure_2/2B/Image_Data/d1-170_0min_TECPR1.tif]

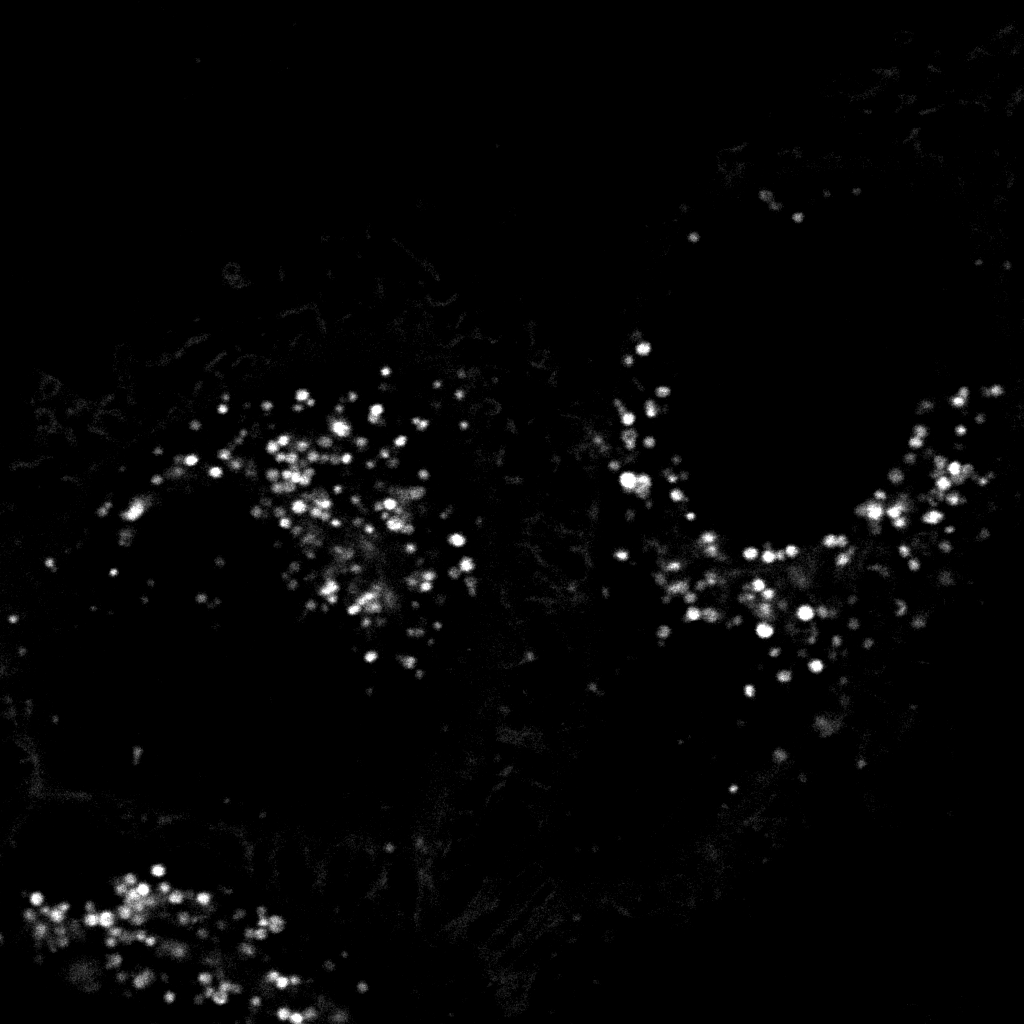

Supplement: Supplementary file 4 — Source Data for Figure 2 [file EMBR-24-e56841-s005.zip › Figure_2/2B/Image_Data/d1-170_15min_LAMP.tif]

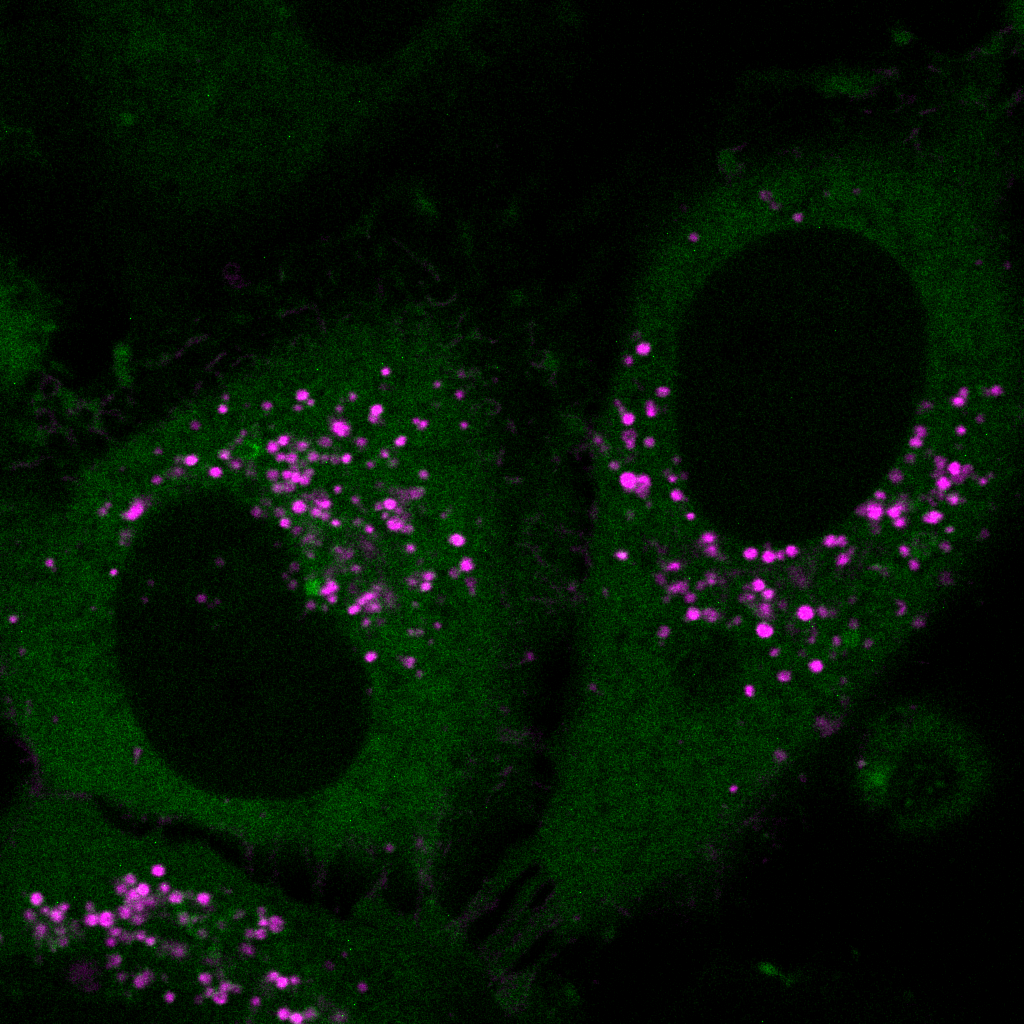

Supplement: Supplementary file 4 — Source Data for Figure 2 [file EMBR-24-e56841-s005.zip › Figure_2/2B/Image_Data/d1-170_15min_merge.tif]

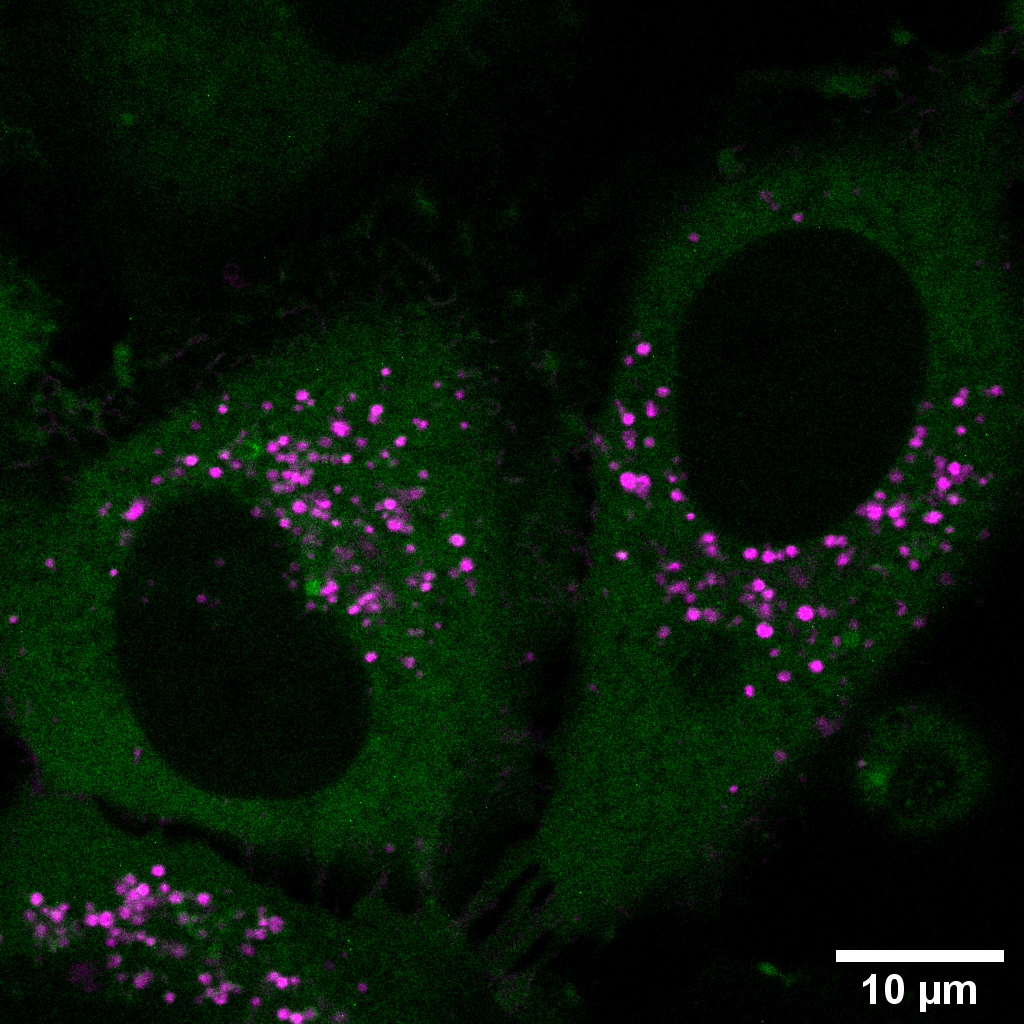

Supplement: Supplementary file 4 — Source Data for Figure 2 [file EMBR-24-e56841-s005.zip › Figure_2/2B/Image_Data/d1-170_15min_scale.tif]

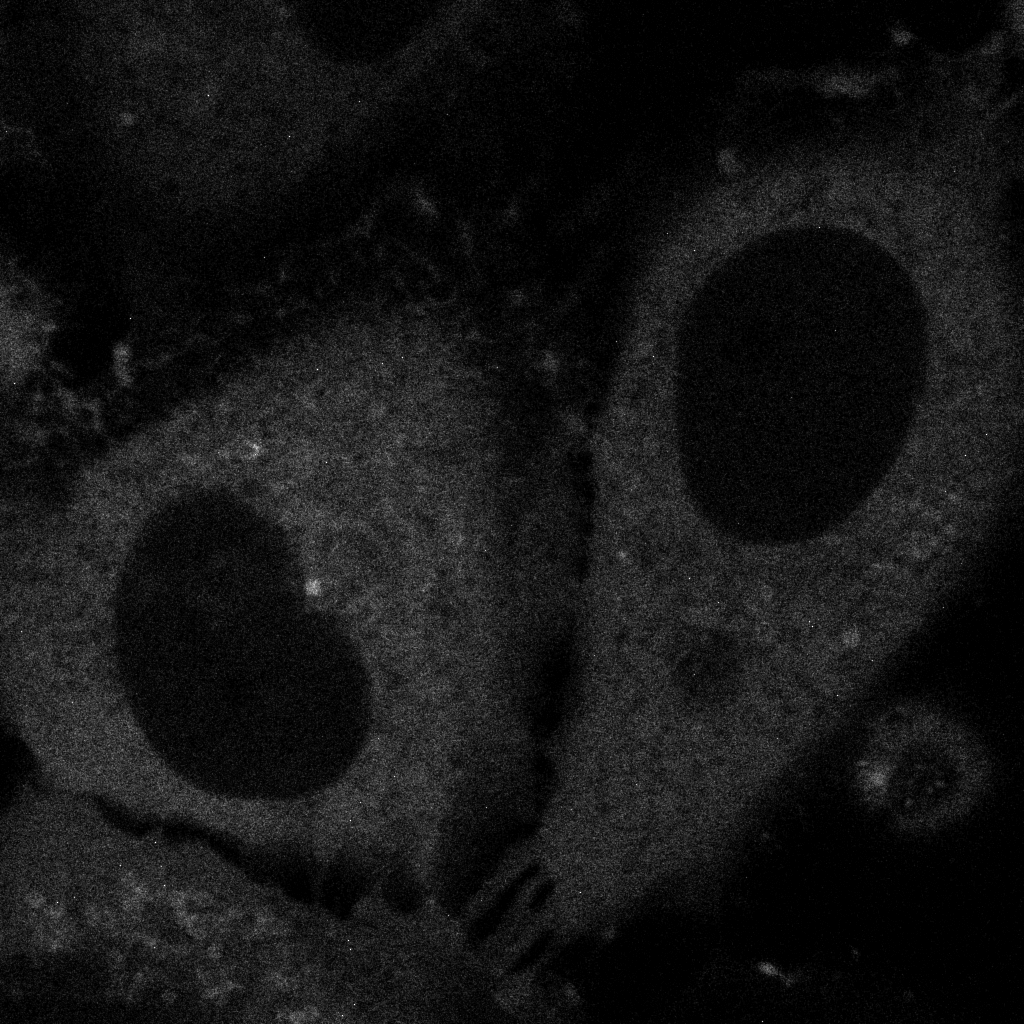

Supplement: Supplementary file 4 — Source Data for Figure 2 [file EMBR-24-e56841-s005.zip › Figure_2/2B/Image_Data/d1-170_15min_TECPR1.tif]

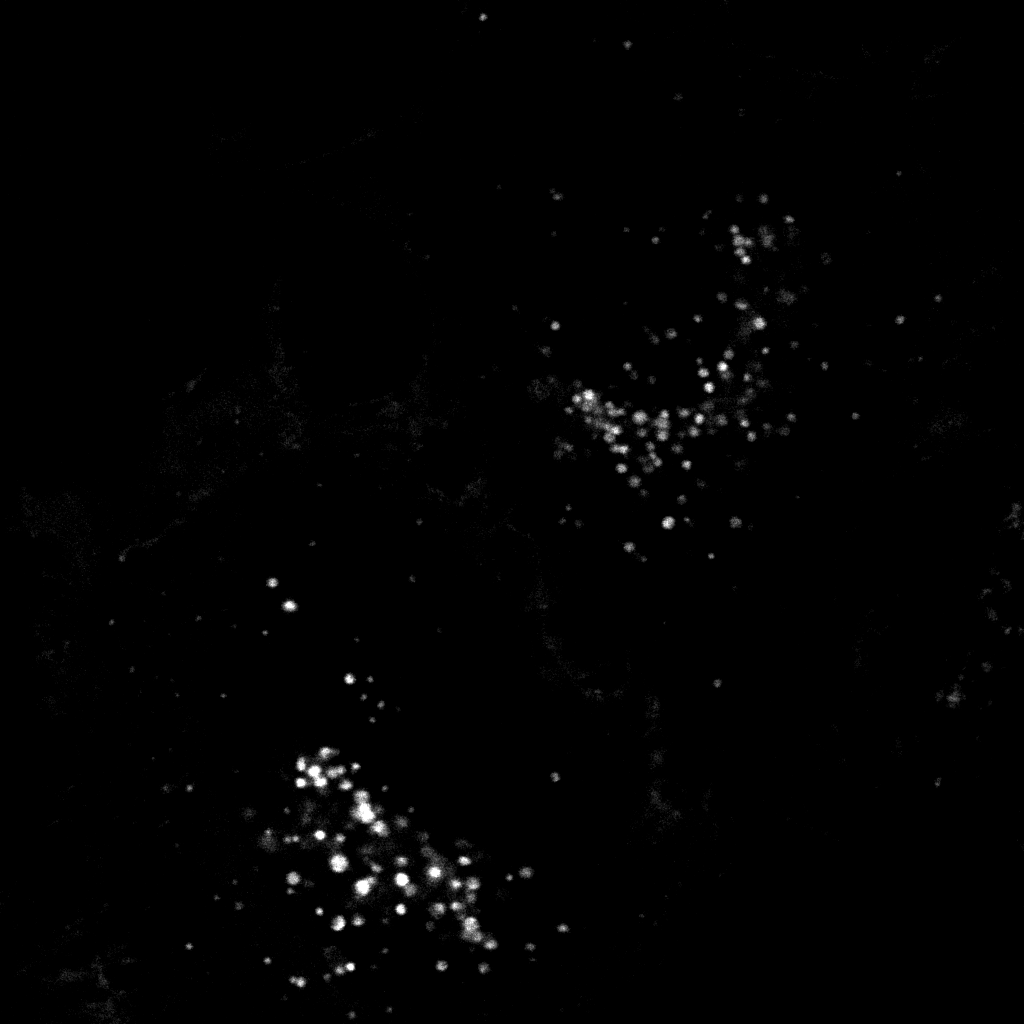

Supplement: Supplementary file 4 — Source Data for Figure 2 [file EMBR-24-e56841-s005.zip › Figure_2/2B/Image_Data/d1-377_0min_LAMP.tif]

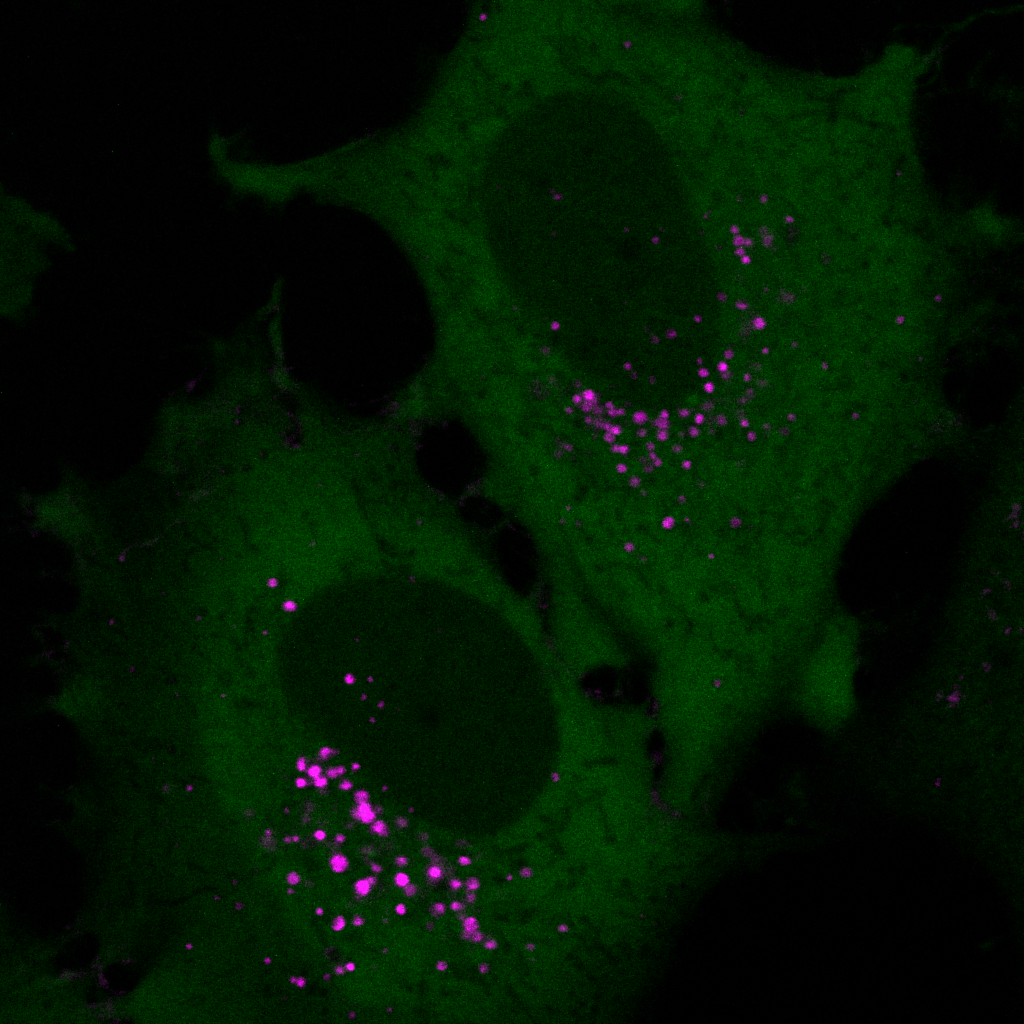

Supplement: Supplementary file 4 — Source Data for Figure 2 [file EMBR-24-e56841-s005.zip › Figure_2/2B/Image_Data/d1-377_0min_merge.tif]

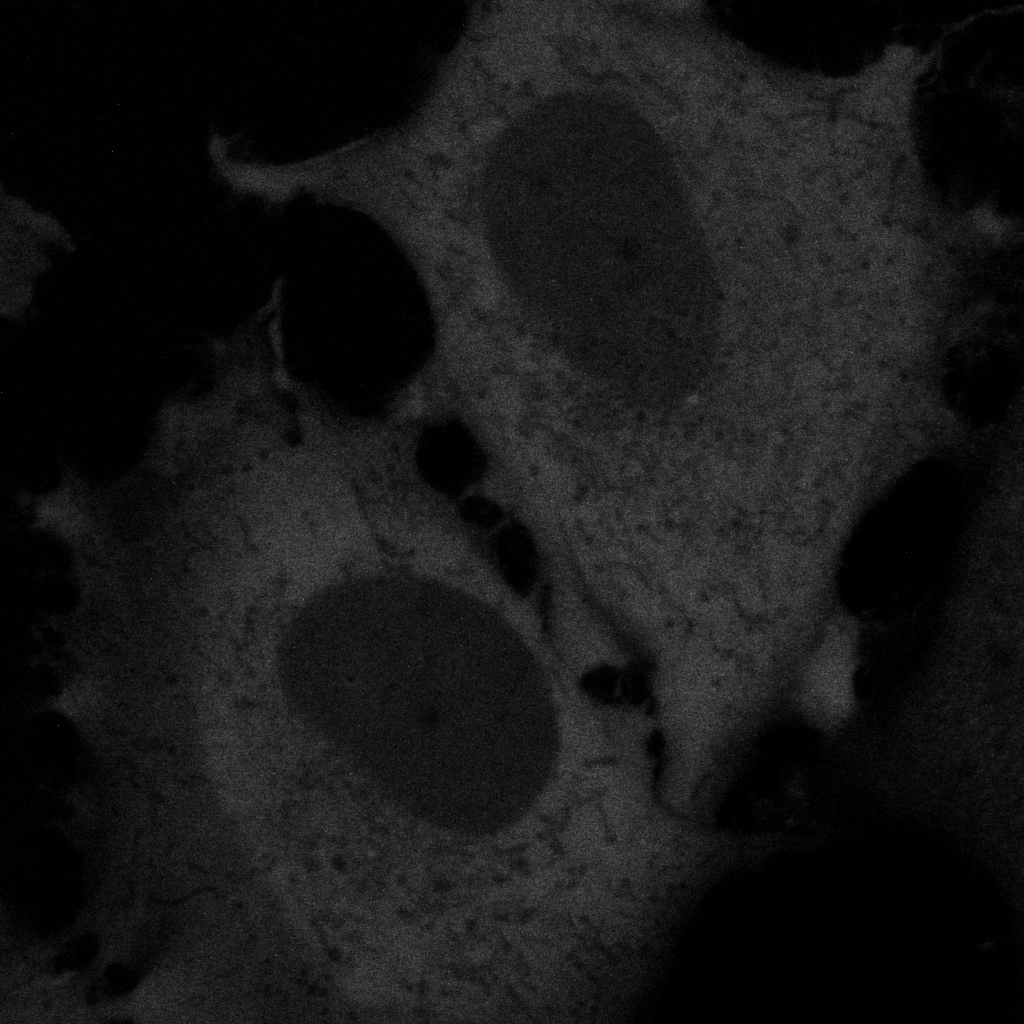

Supplement: Supplementary file 4 — Source Data for Figure 2 [file EMBR-24-e56841-s005.zip › Figure_2/2B/Image_Data/d1-377_0min_TECPR1.tif]

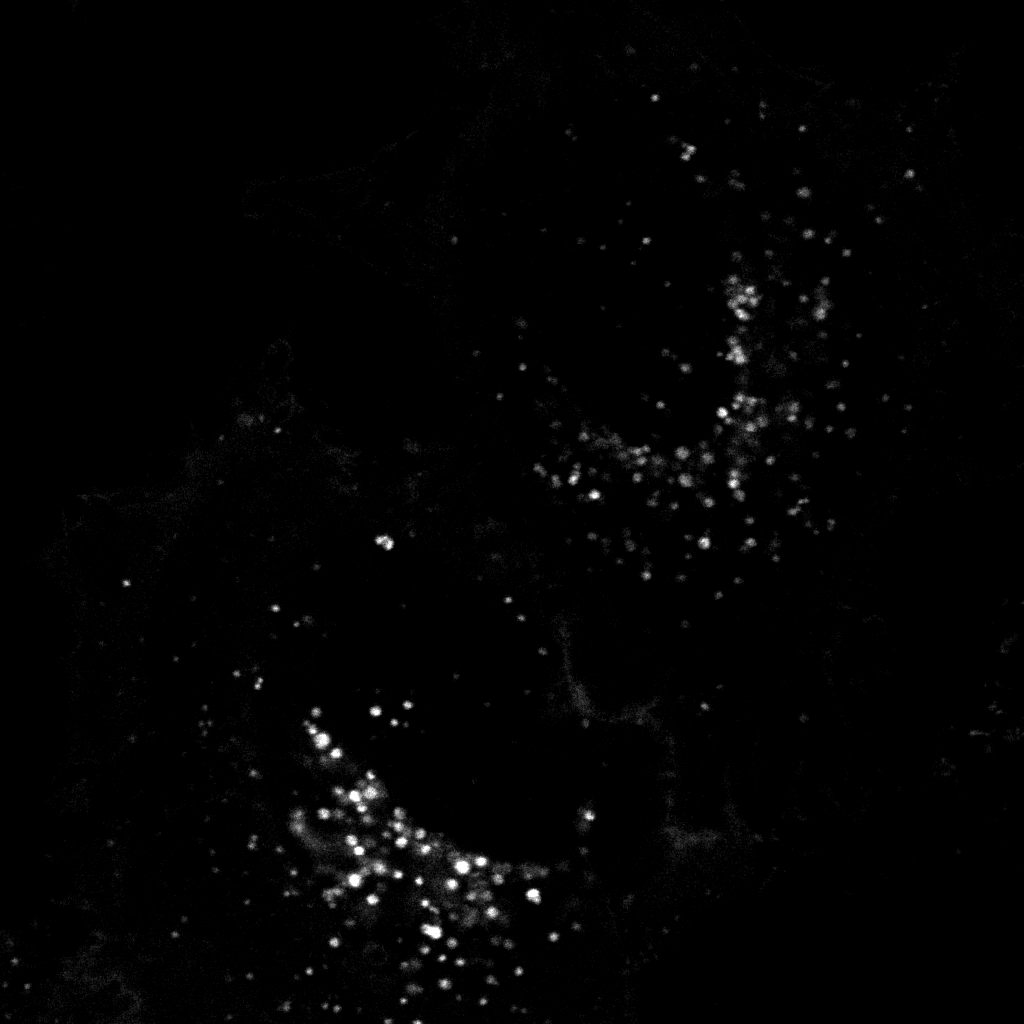

Supplement: Supplementary file 4 — Source Data for Figure 2 [file EMBR-24-e56841-s005.zip › Figure_2/2B/Image_Data/d1-377_15min_LAMP.tif]

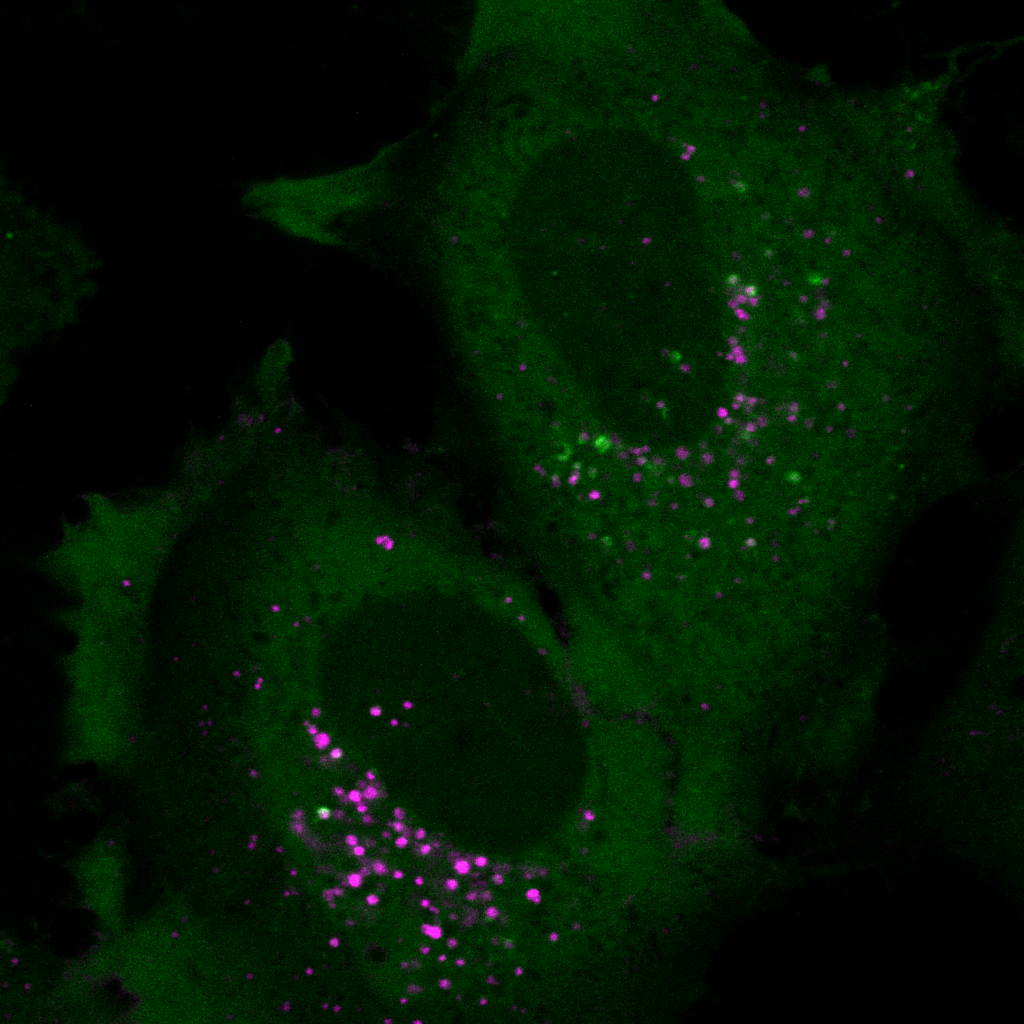

Supplement: Supplementary file 4 — Source Data for Figure 2 [file EMBR-24-e56841-s005.zip › Figure_2/2B/Image_Data/d1-377_15min_merge.tif]

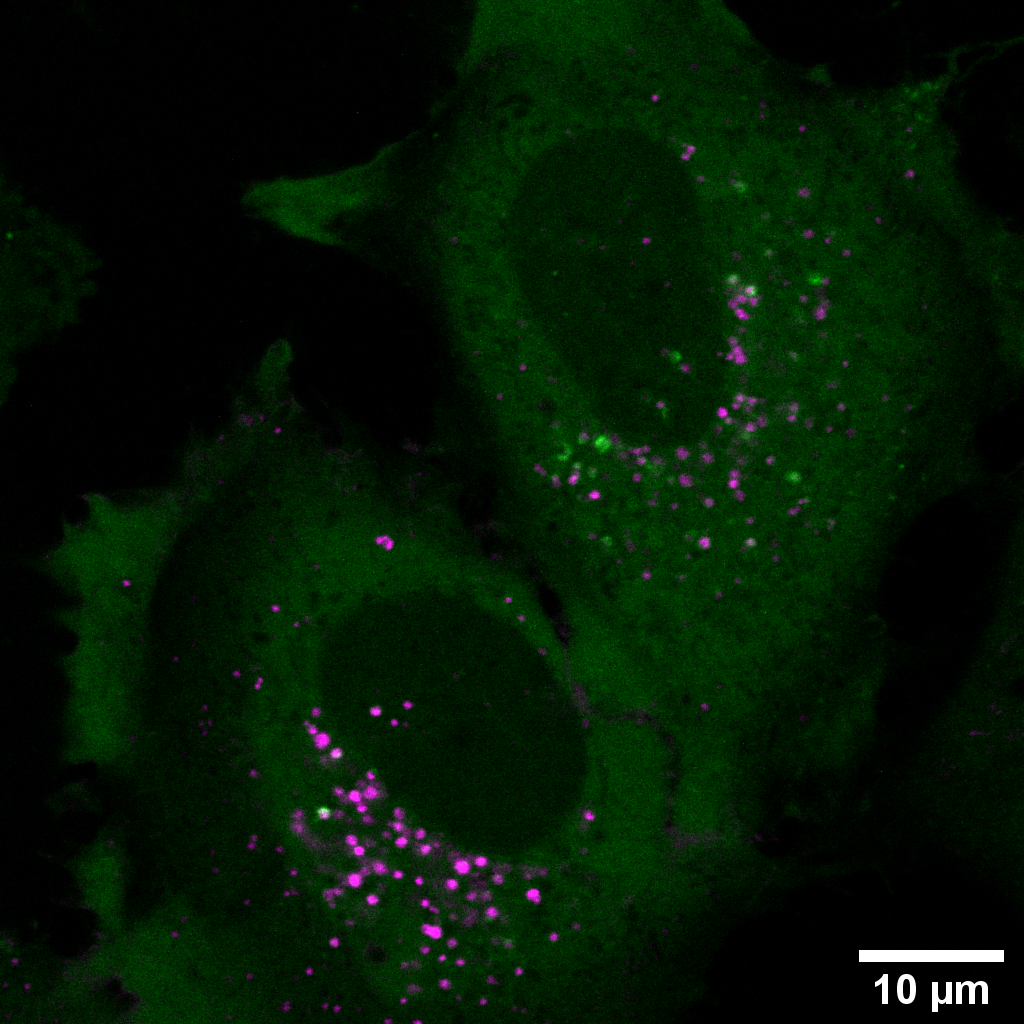

Supplement: Supplementary file 4 — Source Data for Figure 2 [file EMBR-24-e56841-s005.zip › Figure_2/2B/Image_Data/d1-377_15min_scale.tif]

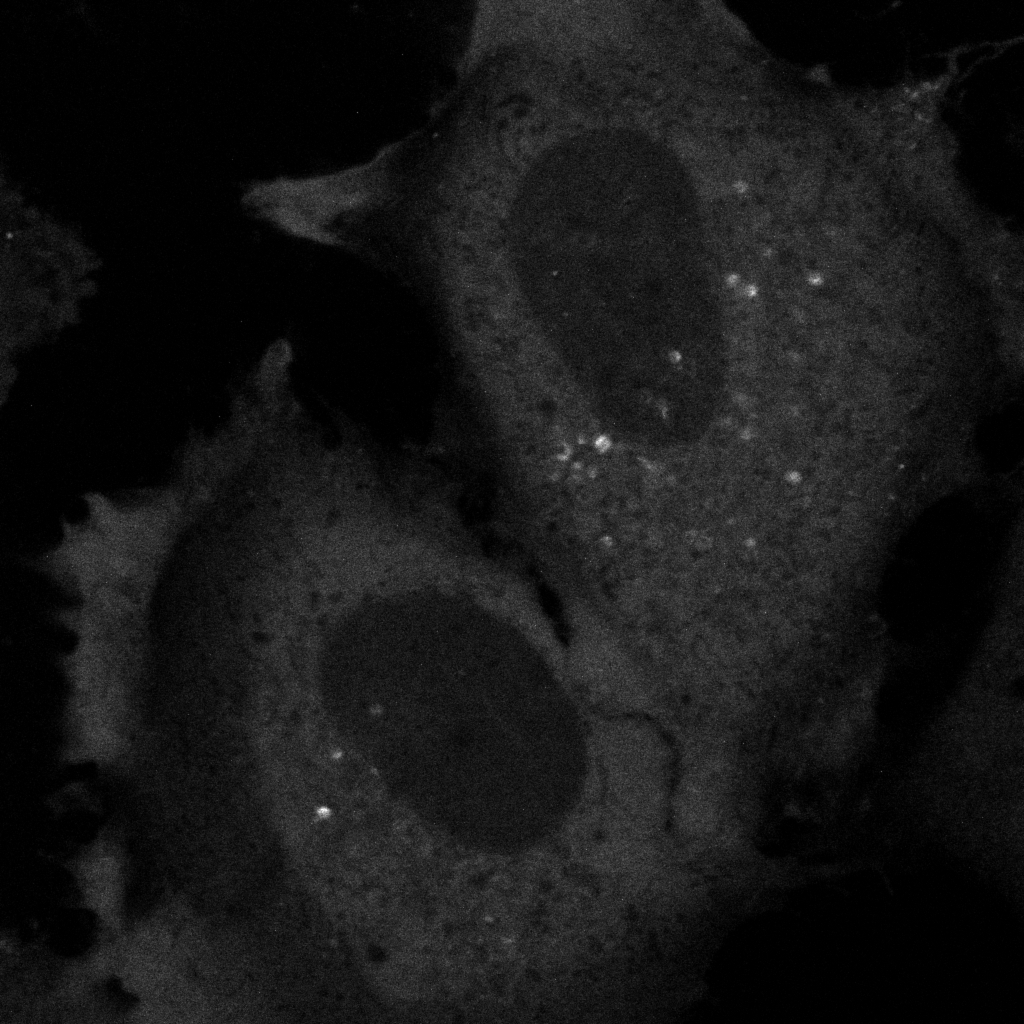

Supplement: Supplementary file 4 — Source Data for Figure 2 [file EMBR-24-e56841-s005.zip › Figure_2/2B/Image_Data/d1-377_15min_TECPR1.tif]

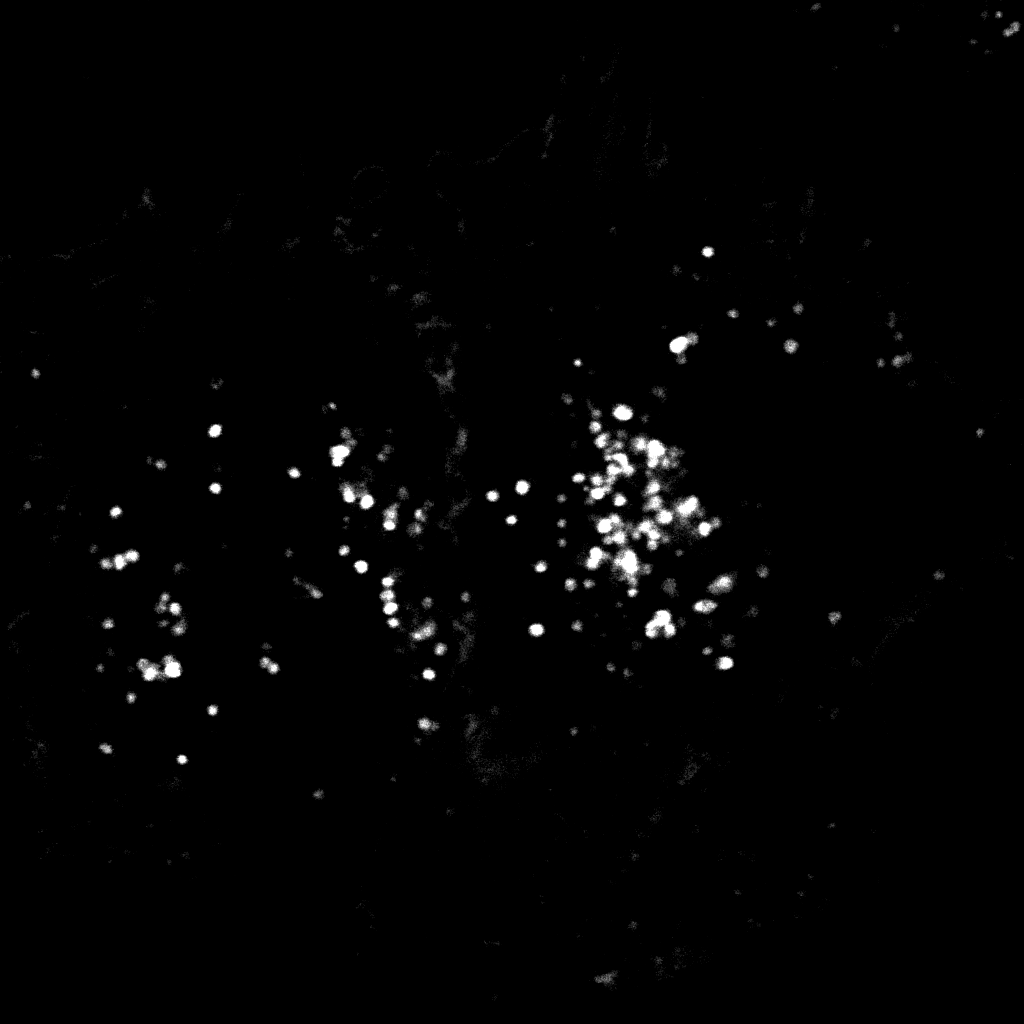

Supplement: Supplementary file 4 — Source Data for Figure 2 [file EMBR-24-e56841-s005.zip › Figure_2/2B/Image_Data/d209-376_0min_LAMP.tif]

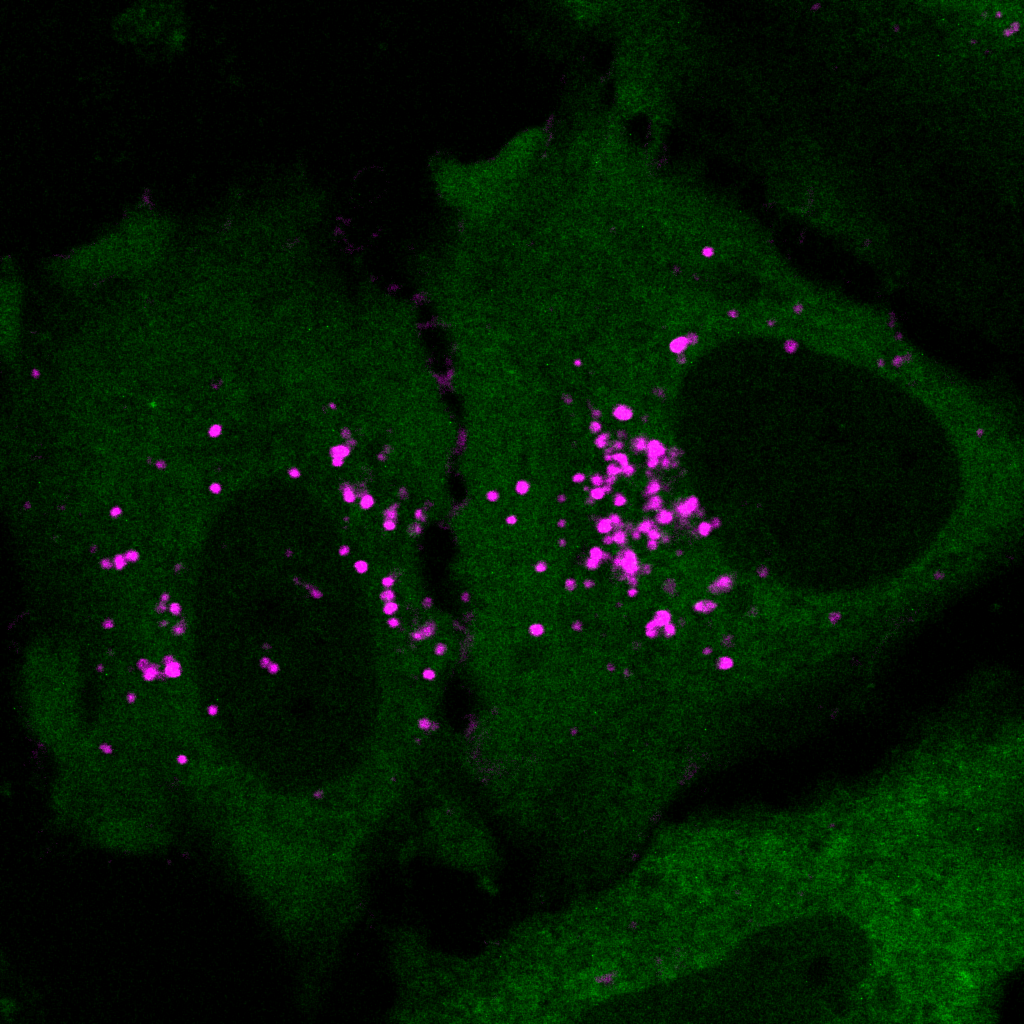

Supplement: Supplementary file 4 — Source Data for Figure 2 [file EMBR-24-e56841-s005.zip › Figure_2/2B/Image_Data/d209-376_0min_merge.tif]

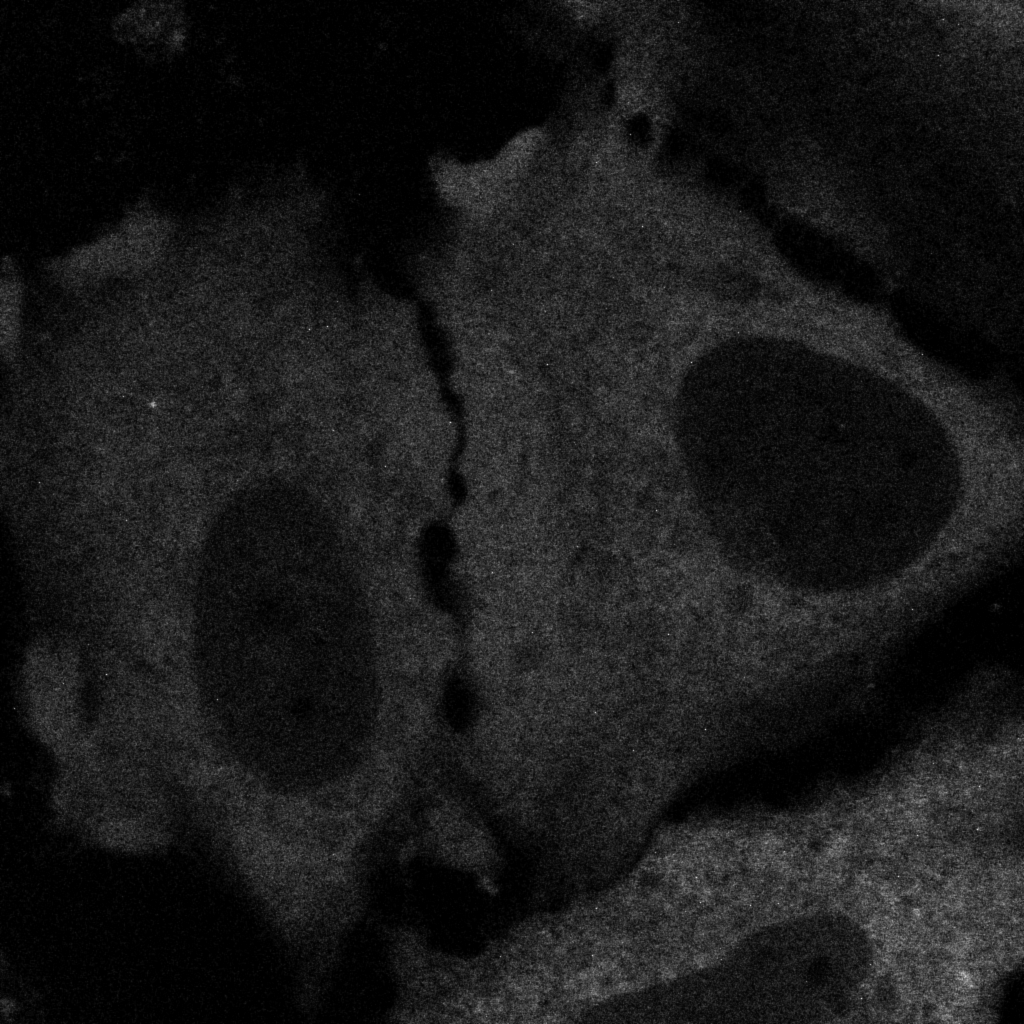

Supplement: Supplementary file 4 — Source Data for Figure 2 [file EMBR-24-e56841-s005.zip › Figure_2/2B/Image_Data/d209-376_0min_TECPR1.tif]

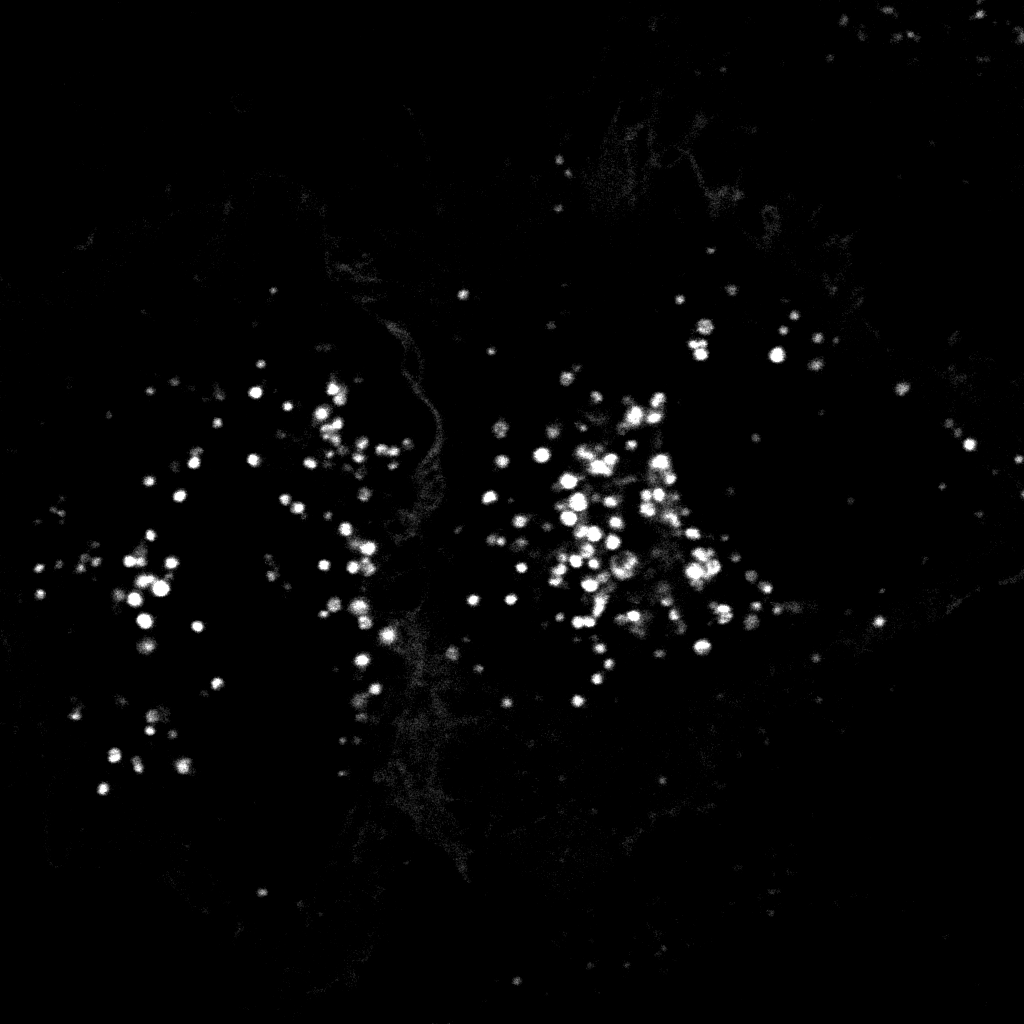

Supplement: Supplementary file 4 — Source Data for Figure 2 [file EMBR-24-e56841-s005.zip › Figure_2/2B/Image_Data/d209-376_15min_LAMP.tif]
