# Supplementary figures and images for: An ATG12‐ATG5‐TECPR1 E3‐like complex regulates unconventional LC3 lipidation at damaged lysosomes (part 2 of 4)
Source: EMBO Rep. 2023 Jun 29;24(9):e56841. doi: 10.15252/embr.202356841 (PMC10481663; doi:10.15252/embr.202356841)

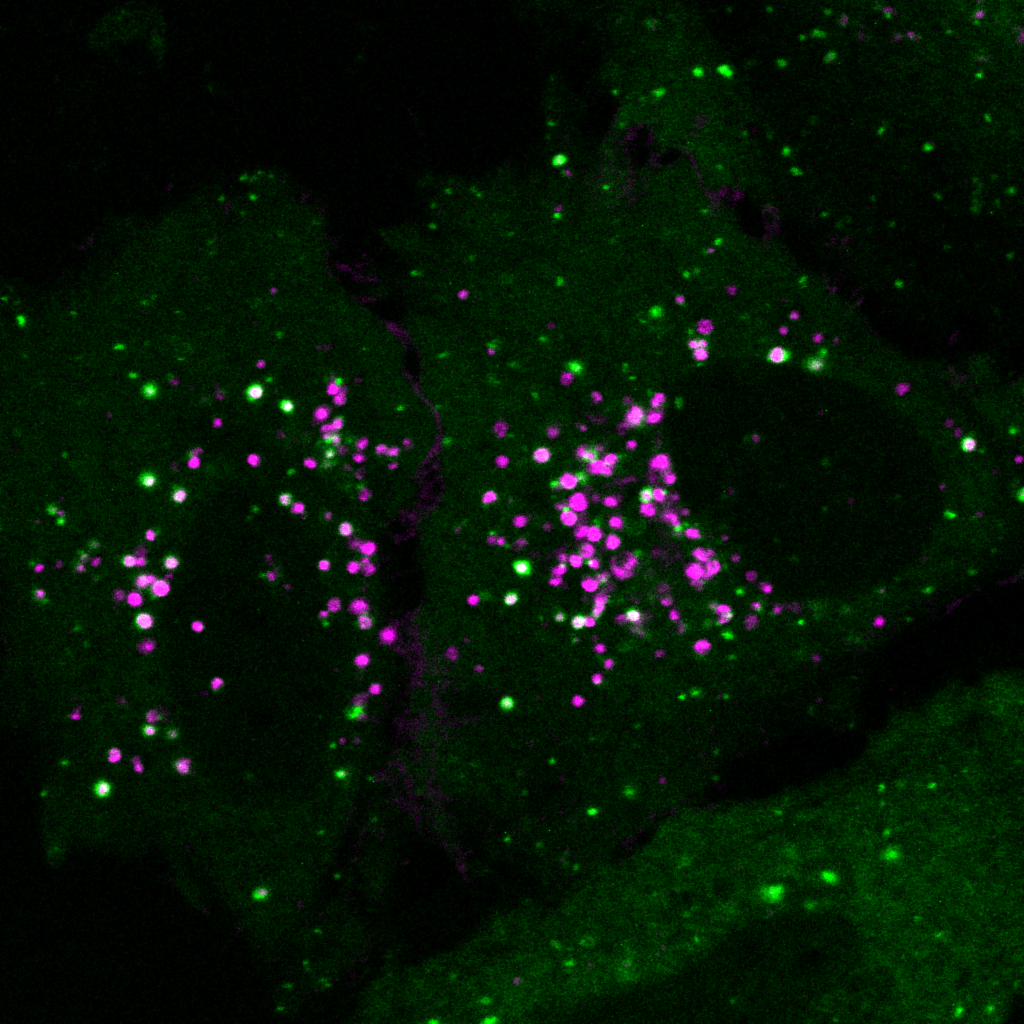

Supplement: Supplementary file 4 — Source Data for Figure 2 [file EMBR-24-e56841-s005.zip › Figure_2/2B/Image_Data/d209-376_15min_merge.tif]

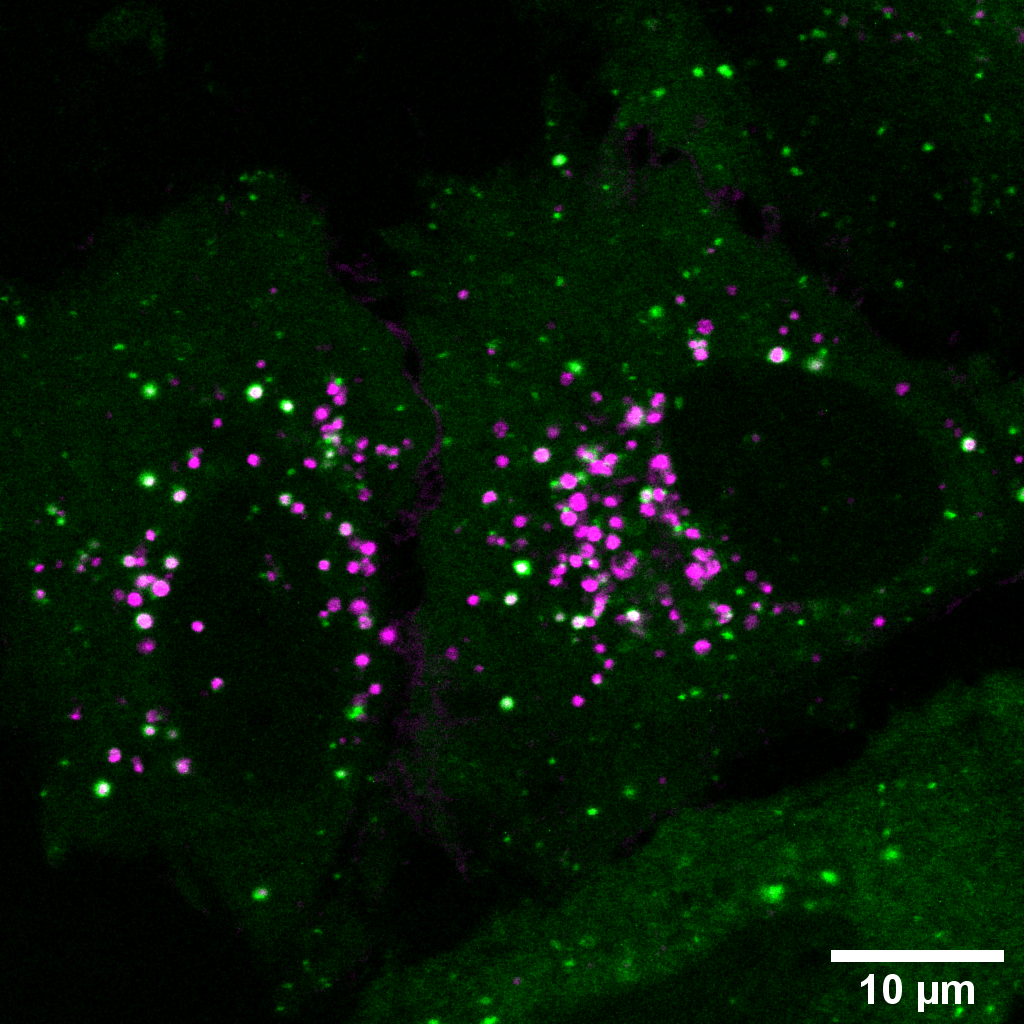

Supplement: Supplementary file 4 — Source Data for Figure 2 [file EMBR-24-e56841-s005.zip › Figure_2/2B/Image_Data/d209-376_15min_scale.tif]

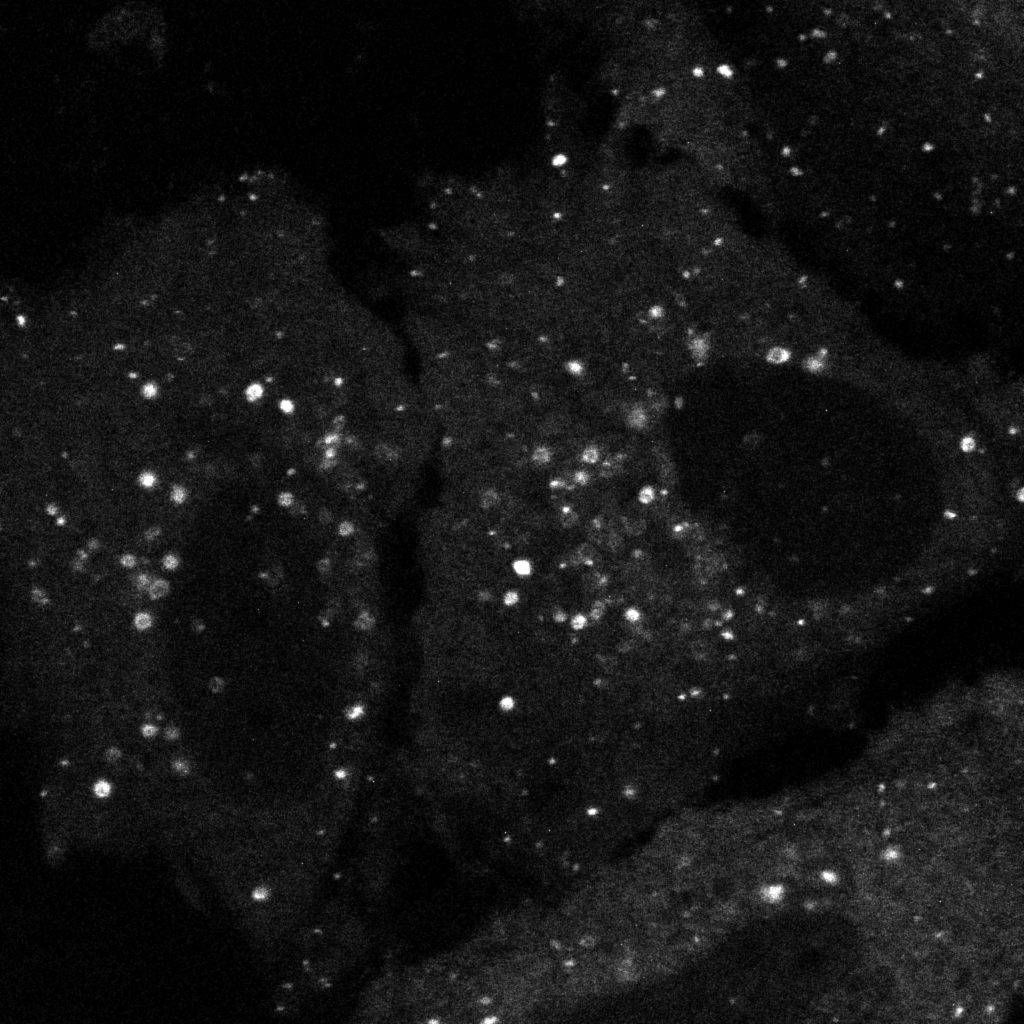

Supplement: Supplementary file 4 — Source Data for Figure 2 [file EMBR-24-e56841-s005.zip › Figure_2/2B/Image_Data/d209-376_15min_TECPR1.tif]

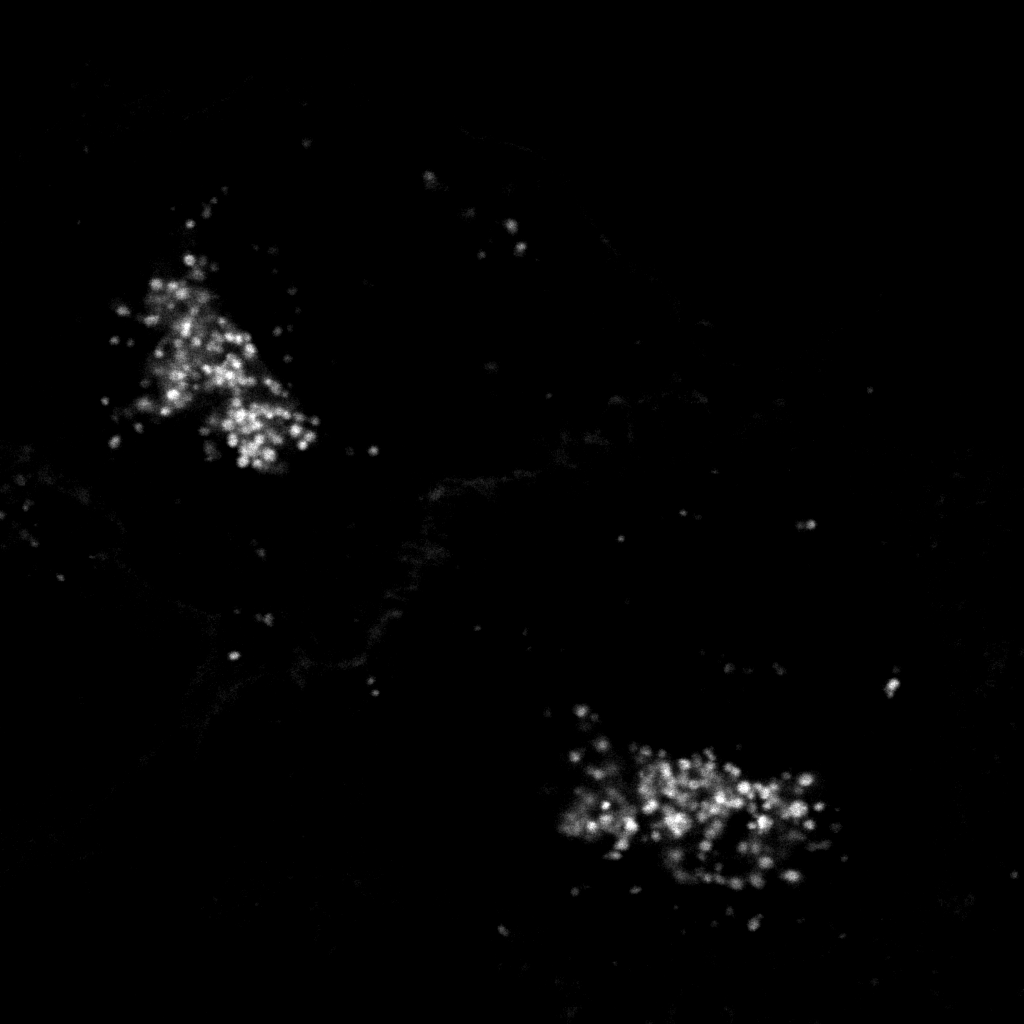

Supplement: Supplementary file 4 — Source Data for Figure 2 [file EMBR-24-e56841-s005.zip › Figure_2/2B/Image_Data/d722-1156_0min_LAMP.tif]

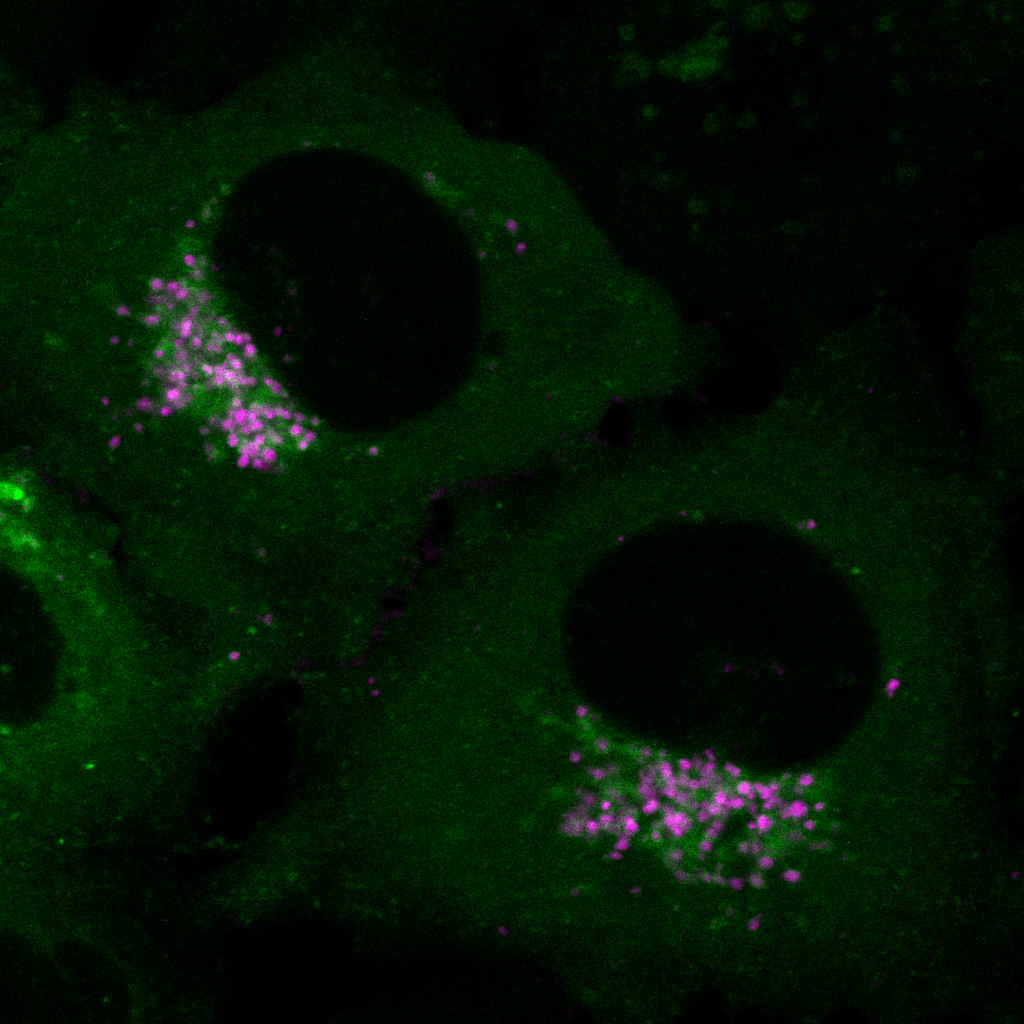

Supplement: Supplementary file 4 — Source Data for Figure 2 [file EMBR-24-e56841-s005.zip › Figure_2/2B/Image_Data/d722-1156_0min_merge.tif]

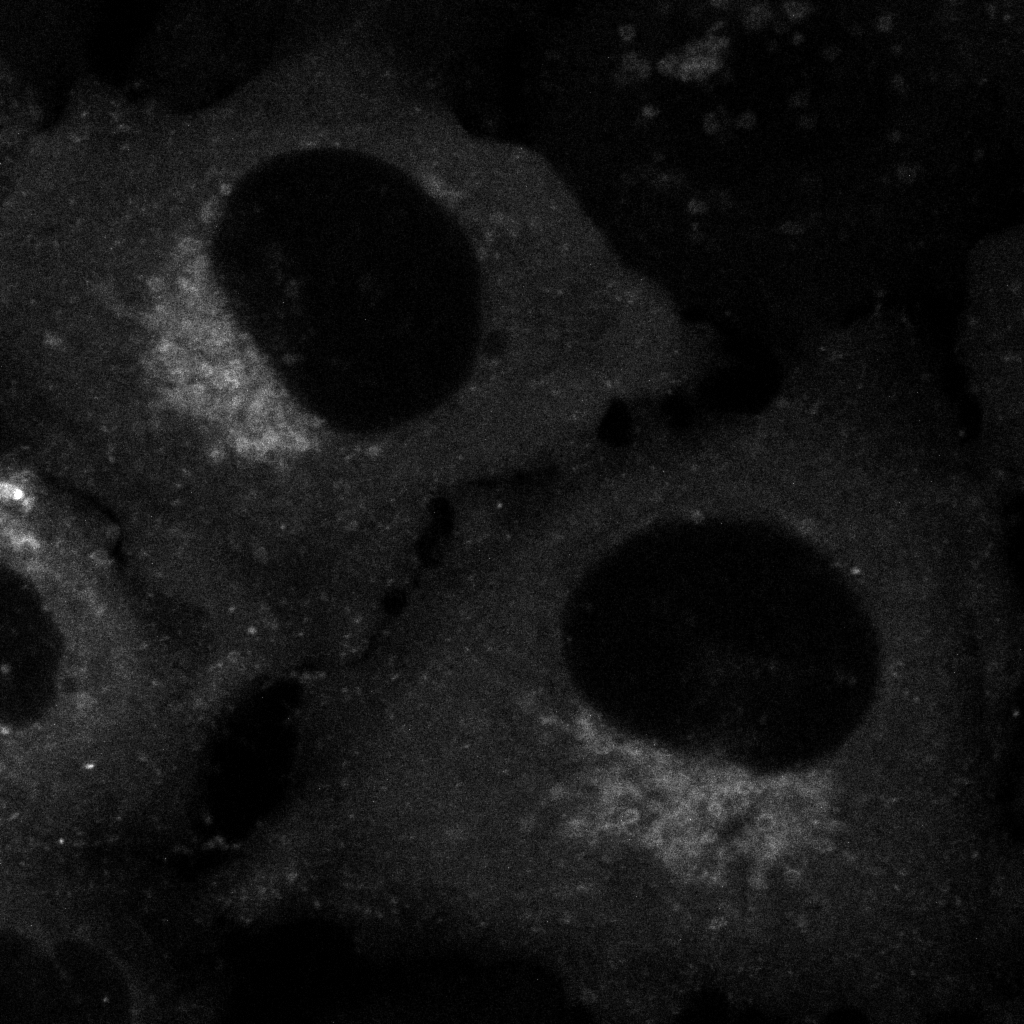

Supplement: Supplementary file 4 — Source Data for Figure 2 [file EMBR-24-e56841-s005.zip › Figure_2/2B/Image_Data/d722-1156_0min_TECPR1.tif]

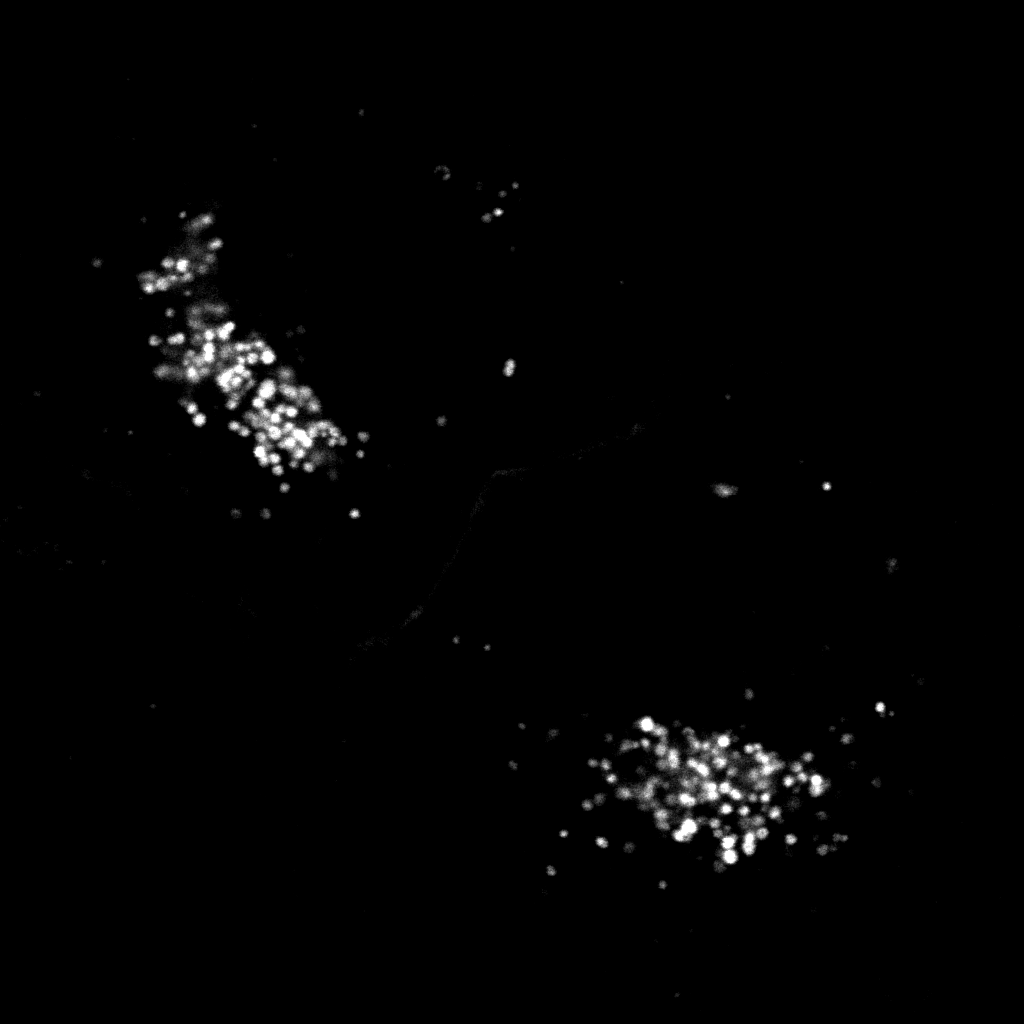

Supplement: Supplementary file 4 — Source Data for Figure 2 [file EMBR-24-e56841-s005.zip › Figure_2/2B/Image_Data/d722-1156_15min_LAMP.tif]

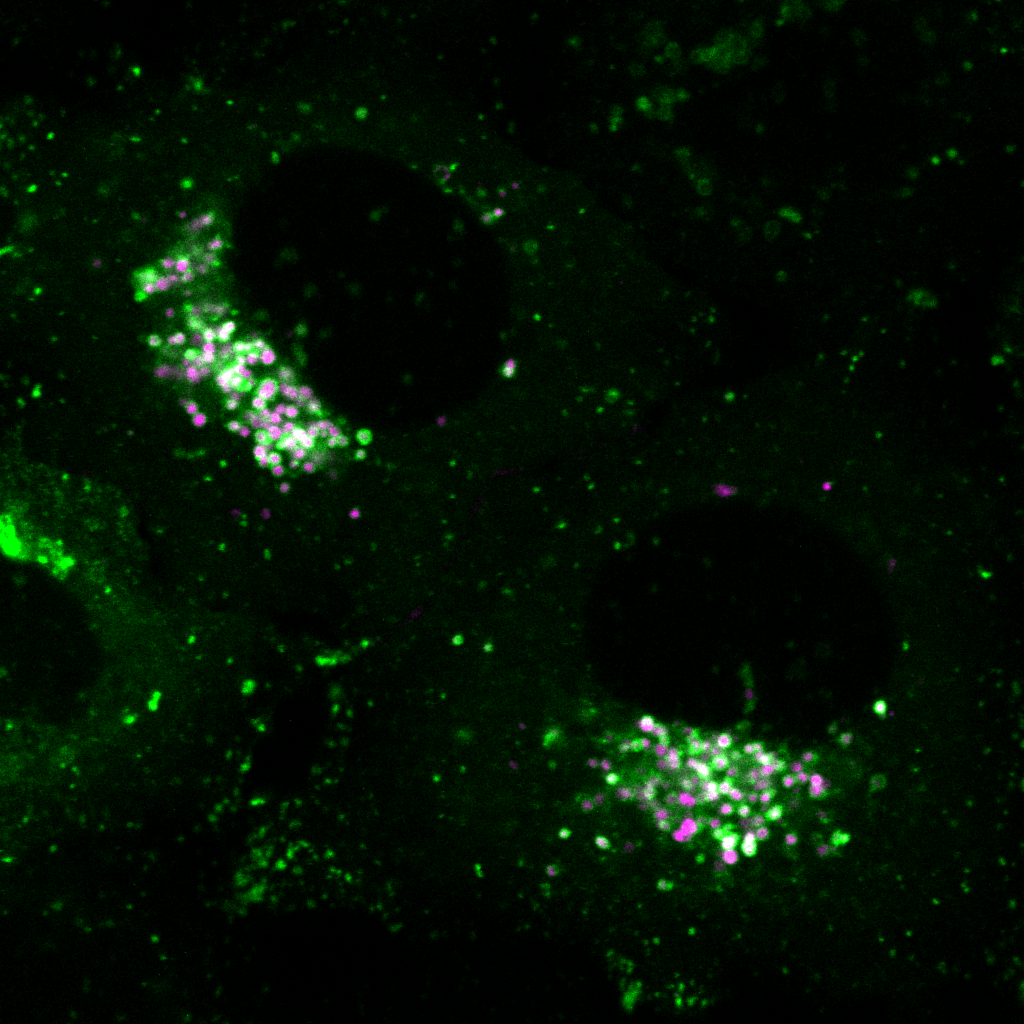

Supplement: Supplementary file 4 — Source Data for Figure 2 [file EMBR-24-e56841-s005.zip › Figure_2/2B/Image_Data/d722-1156_15min_merge.tif]

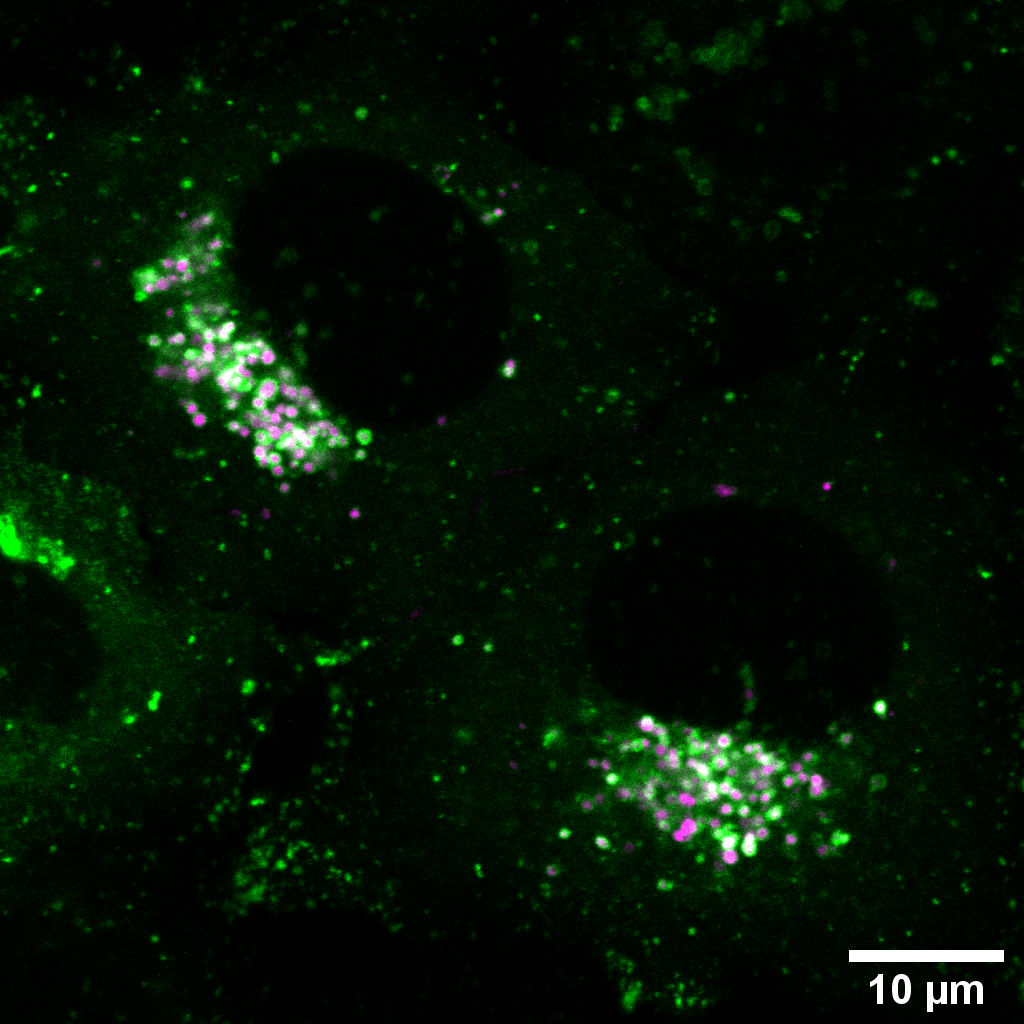

Supplement: Supplementary file 4 — Source Data for Figure 2 [file EMBR-24-e56841-s005.zip › Figure_2/2B/Image_Data/d722-1156_15min_scale.tif]

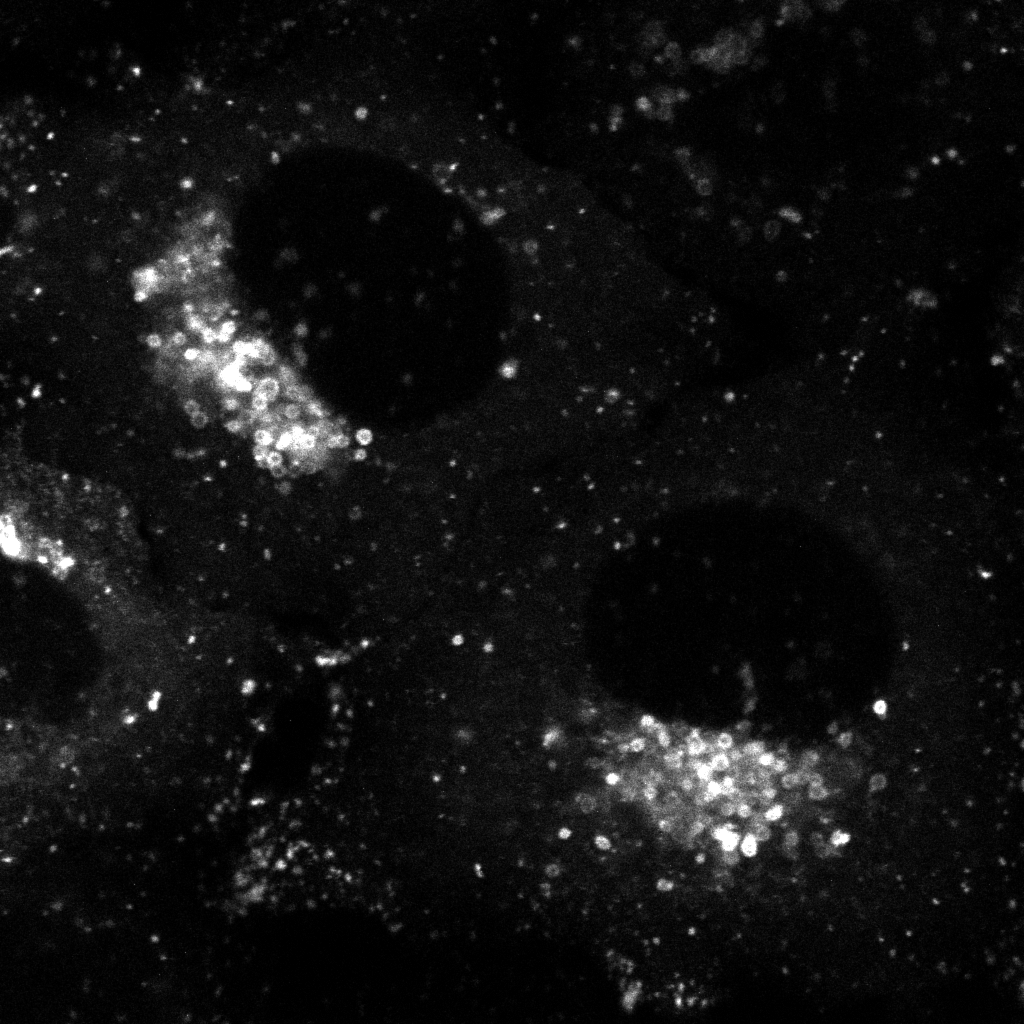

Supplement: Supplementary file 4 — Source Data for Figure 2 [file EMBR-24-e56841-s005.zip › Figure_2/2B/Image_Data/d722-1156_15min_TECPR1.tif]

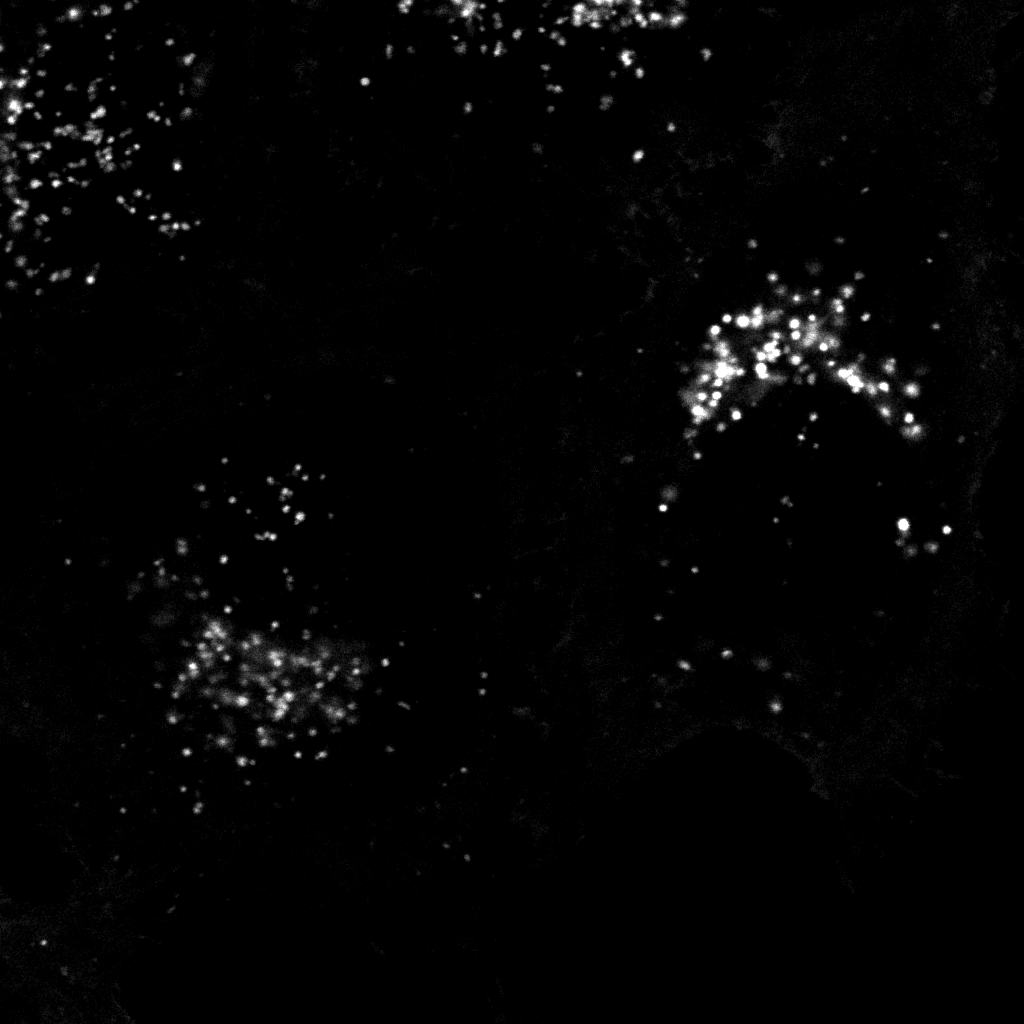

Supplement: Supplementary file 4 — Source Data for Figure 2 [file EMBR-24-e56841-s005.zip › Figure_2/2B/Image_Data/dPH_0min_LAMP.tif]

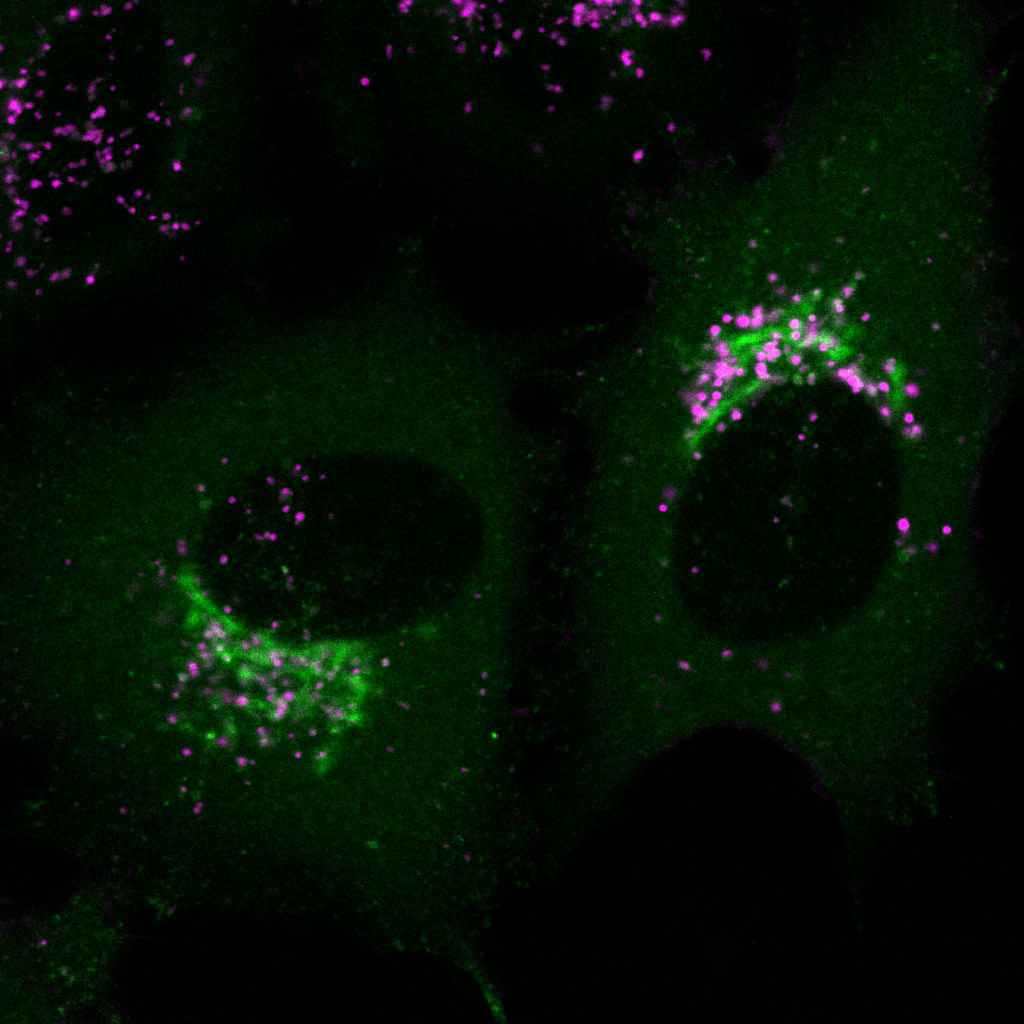

Supplement: Supplementary file 4 — Source Data for Figure 2 [file EMBR-24-e56841-s005.zip › Figure_2/2B/Image_Data/dPH_0min_merge.tif]

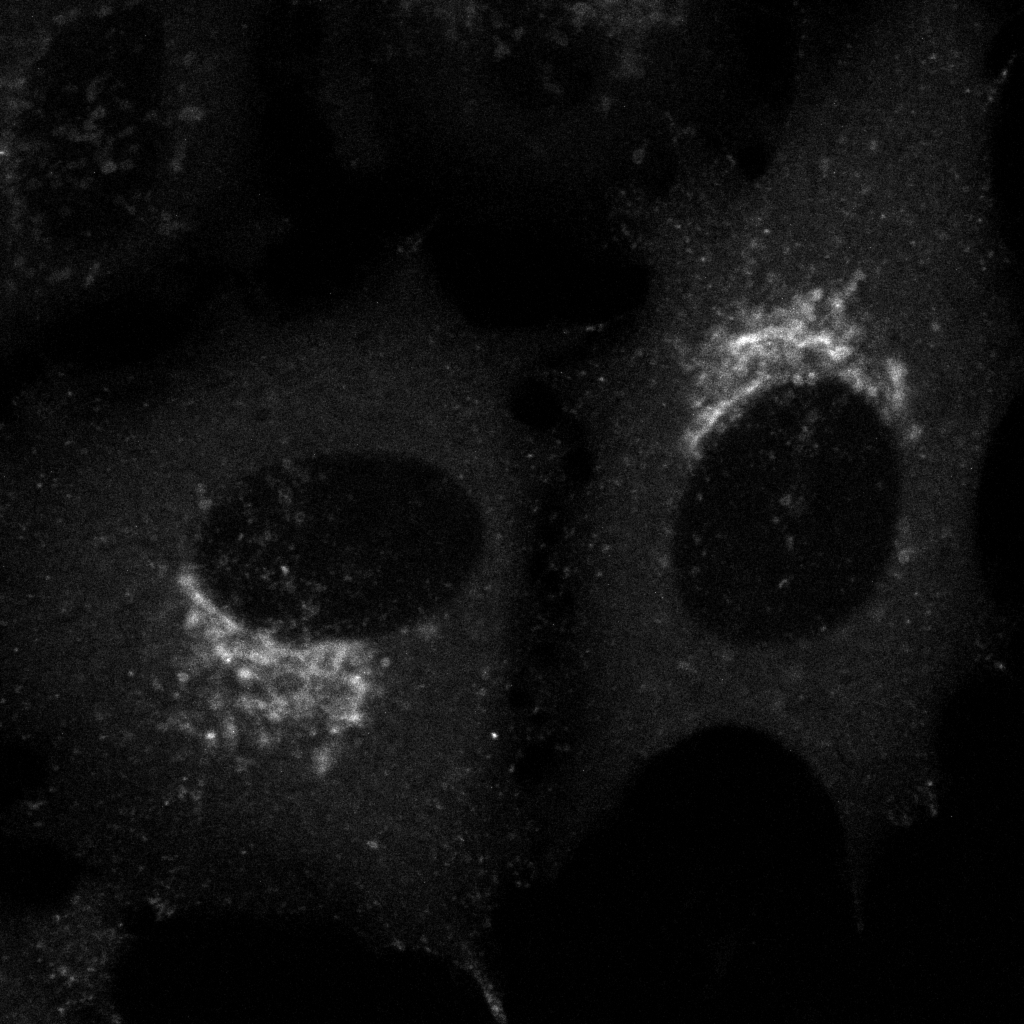

Supplement: Supplementary file 4 — Source Data for Figure 2 [file EMBR-24-e56841-s005.zip › Figure_2/2B/Image_Data/dPH_0min_TECPR1.tif]

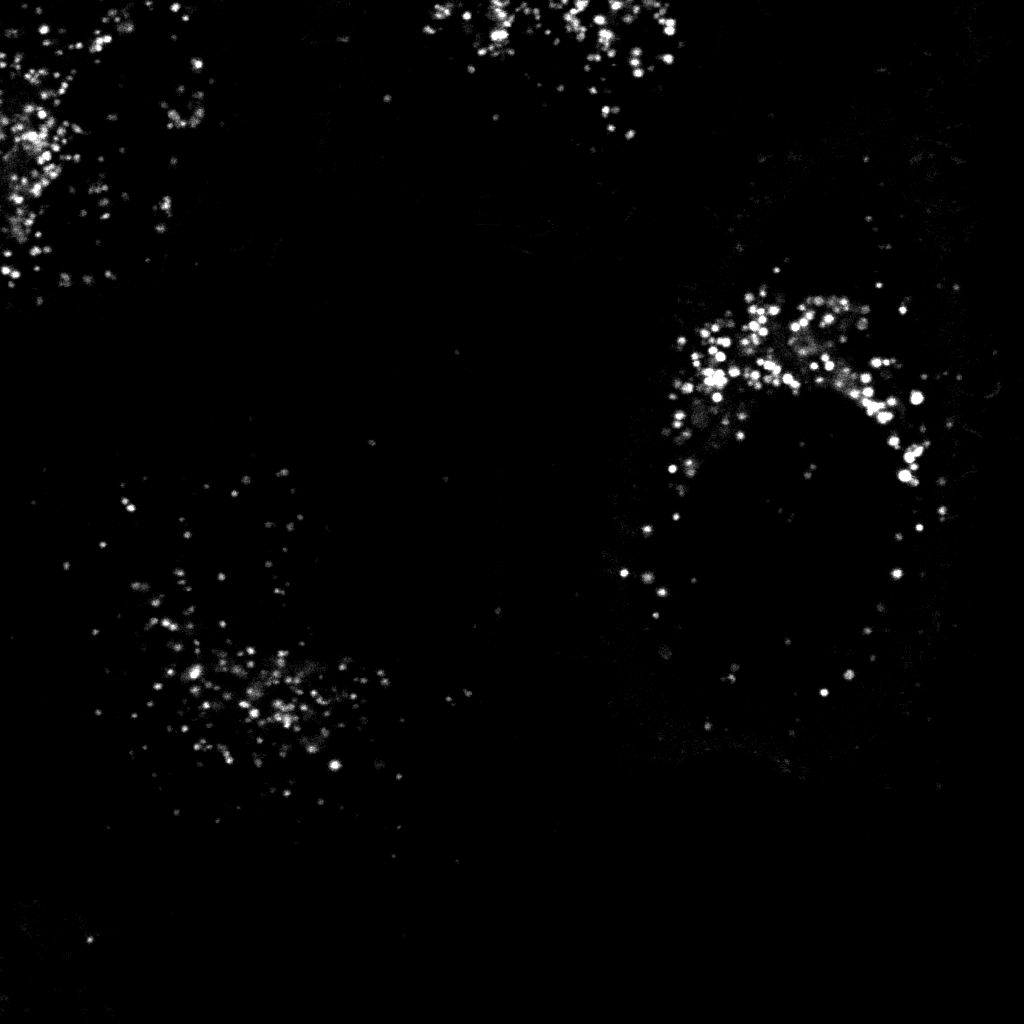

Supplement: Supplementary file 4 — Source Data for Figure 2 [file EMBR-24-e56841-s005.zip › Figure_2/2B/Image_Data/dPH_15min_LAMP.tif]

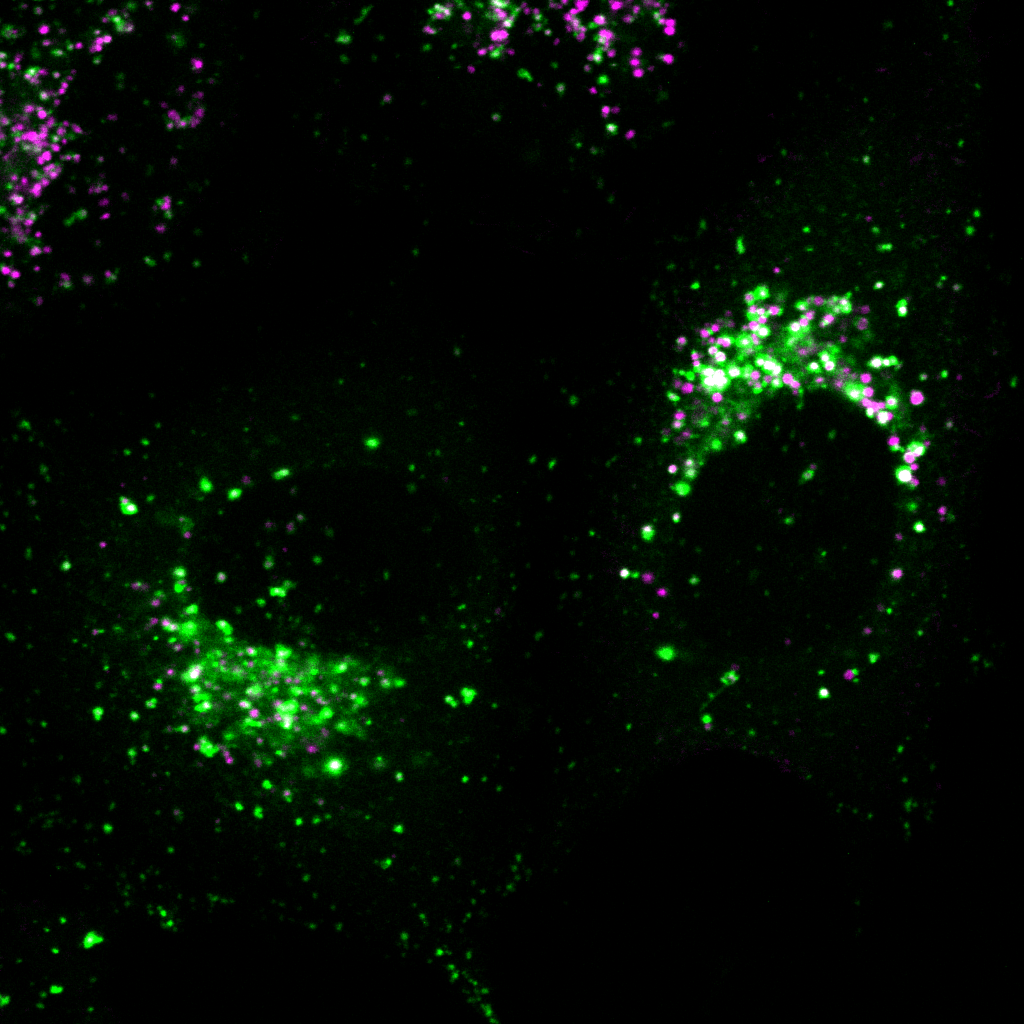

Supplement: Supplementary file 4 — Source Data for Figure 2 [file EMBR-24-e56841-s005.zip › Figure_2/2B/Image_Data/dPH_15min_merge.tif]

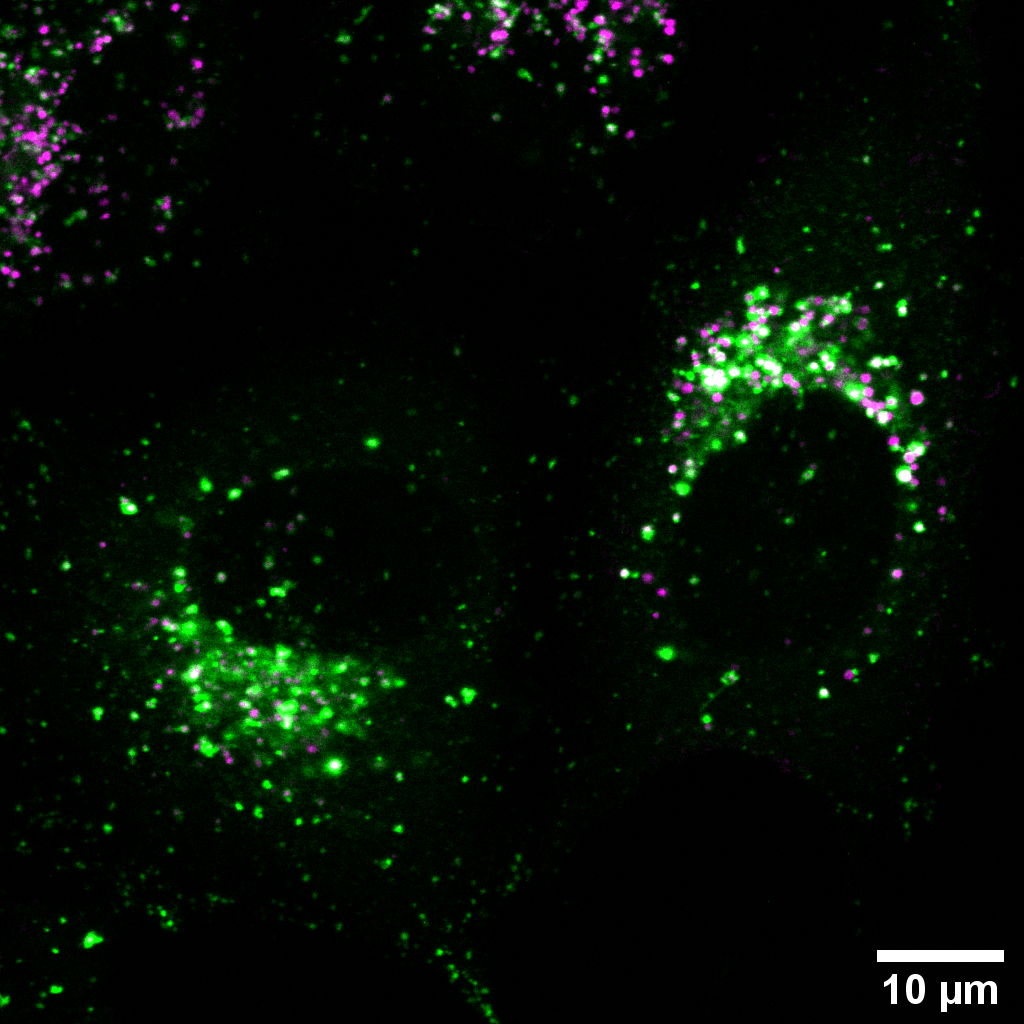

Supplement: Supplementary file 4 — Source Data for Figure 2 [file EMBR-24-e56841-s005.zip › Figure_2/2B/Image_Data/dPH_15min_scale.tif]

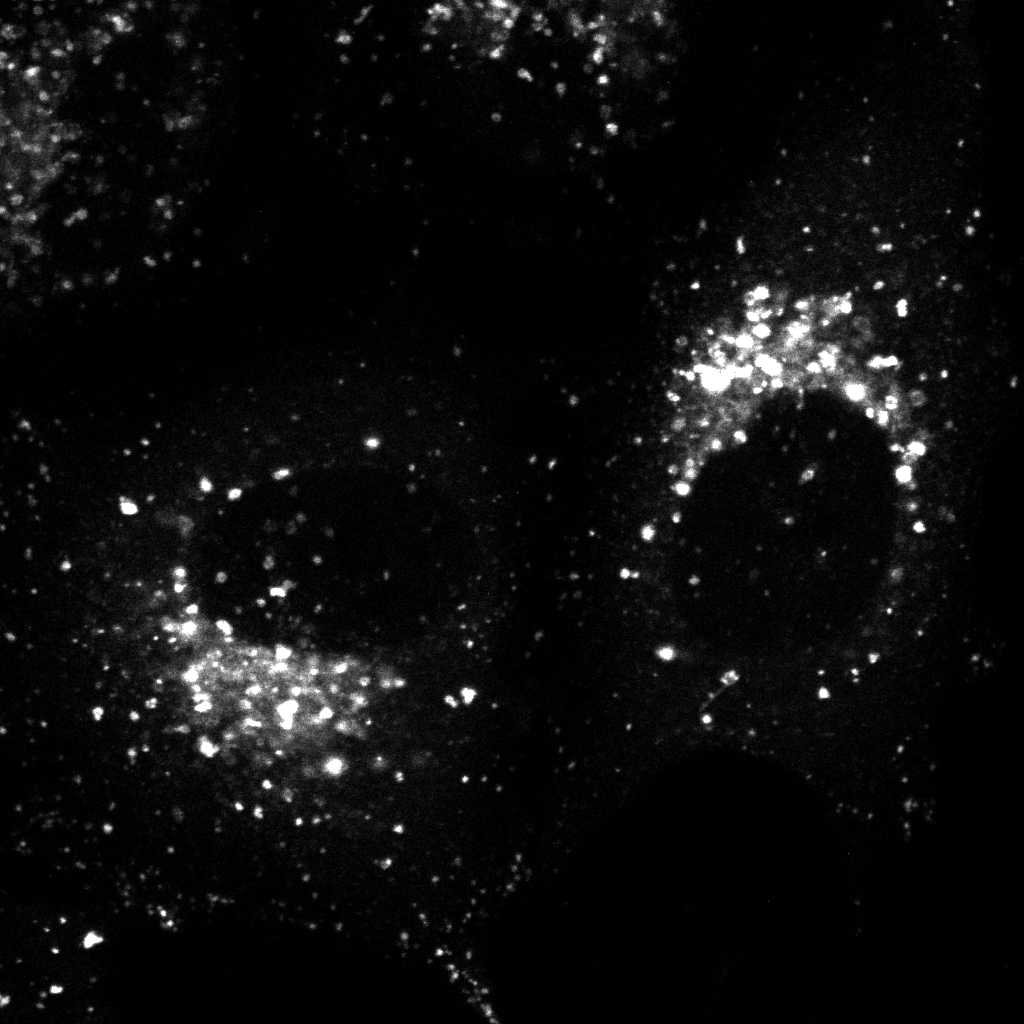

Supplement: Supplementary file 4 — Source Data for Figure 2 [file EMBR-24-e56841-s005.zip › Figure_2/2B/Image_Data/dPH_15min_TECPR1.tif]

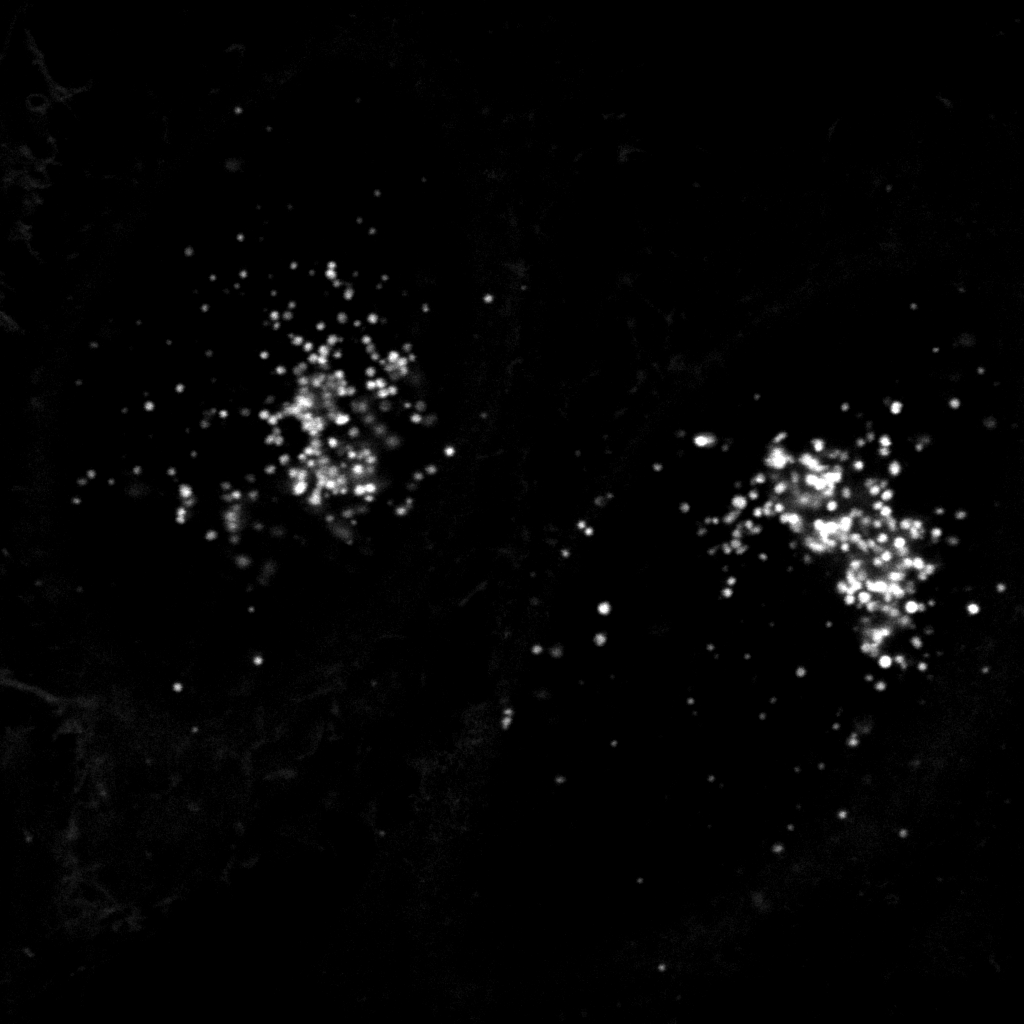

Supplement: Supplementary file 4 — Source Data for Figure 2 [file EMBR-24-e56841-s005.zip › Figure_2/2B/Image_Data/WT_0min_LAMP1.tif]

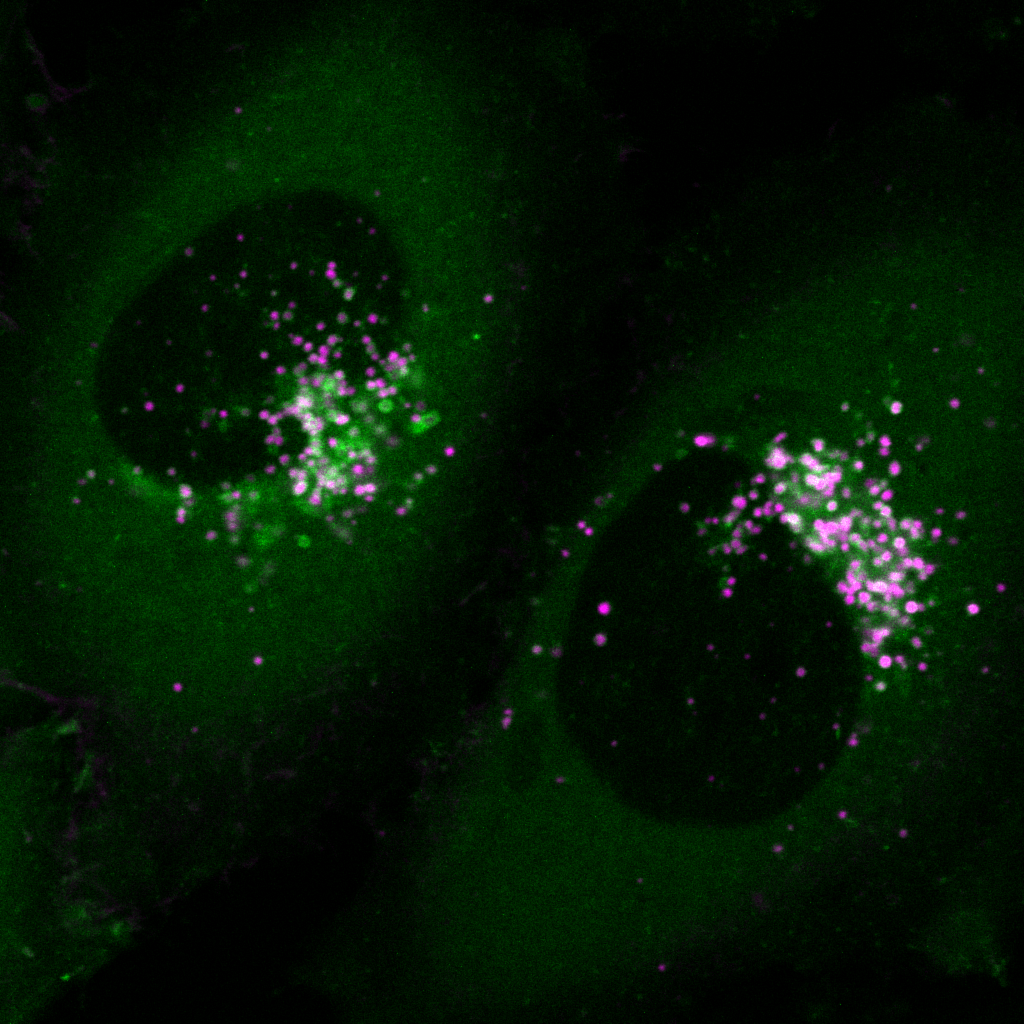

Supplement: Supplementary file 4 — Source Data for Figure 2 [file EMBR-24-e56841-s005.zip › Figure_2/2B/Image_Data/WT_0min_merge.tif]

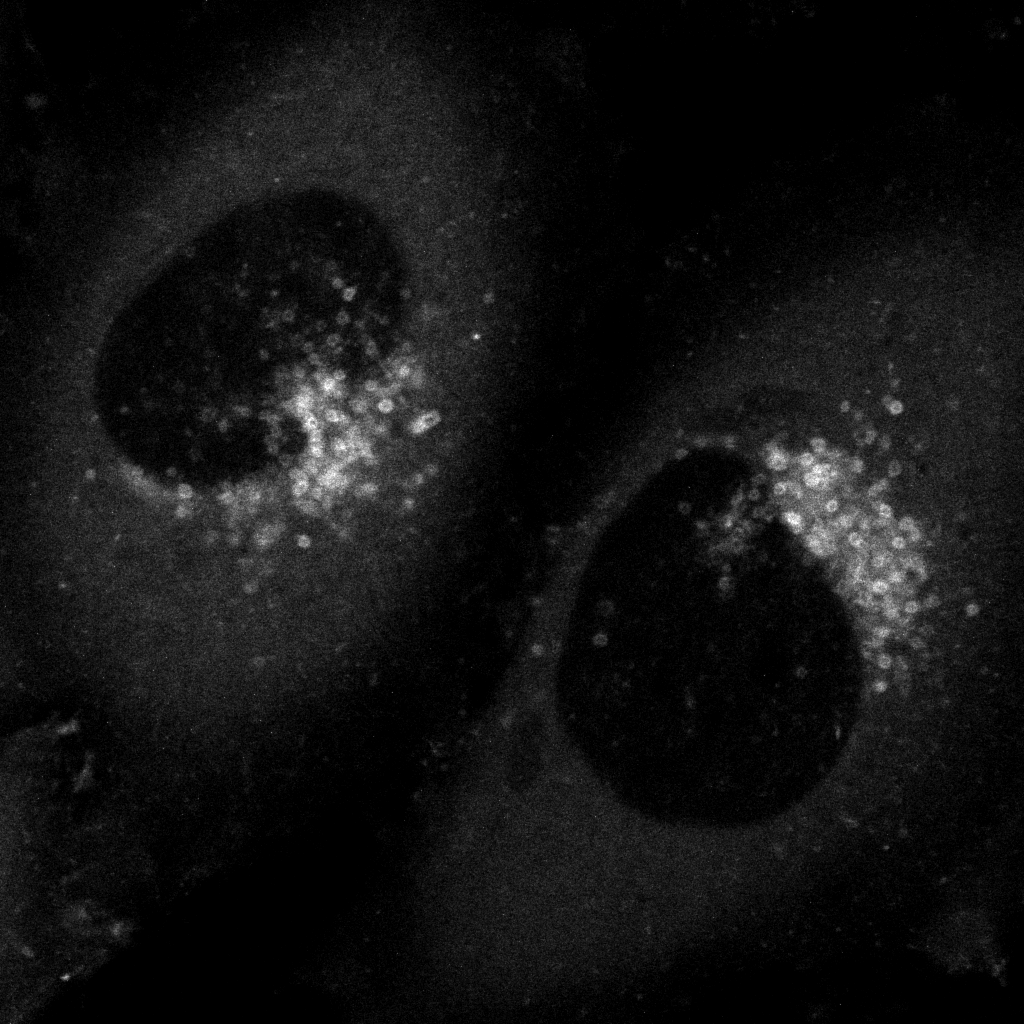

Supplement: Supplementary file 4 — Source Data for Figure 2 [file EMBR-24-e56841-s005.zip › Figure_2/2B/Image_Data/WT_0min_TECPR1.tif]

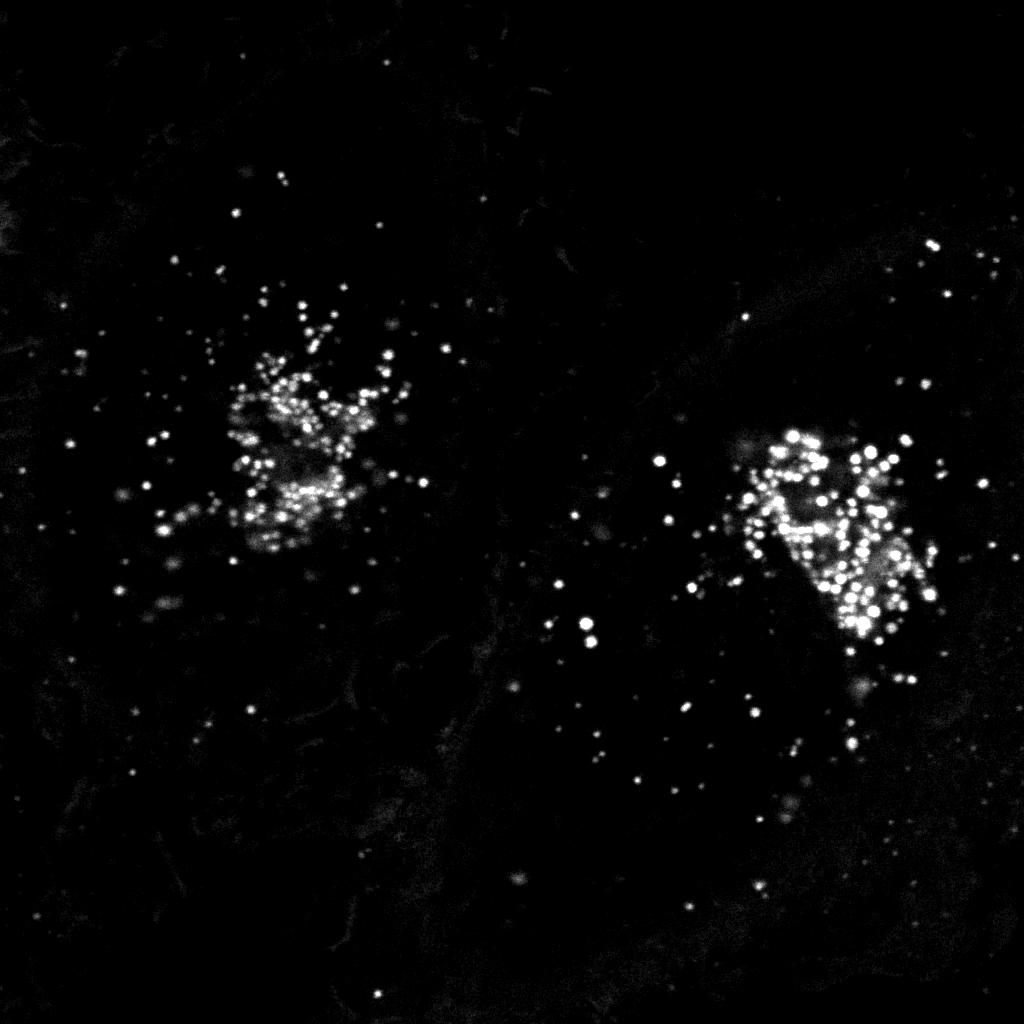

Supplement: Supplementary file 4 — Source Data for Figure 2 [file EMBR-24-e56841-s005.zip › Figure_2/2B/Image_Data/WT_15min_LAMP1.tif]

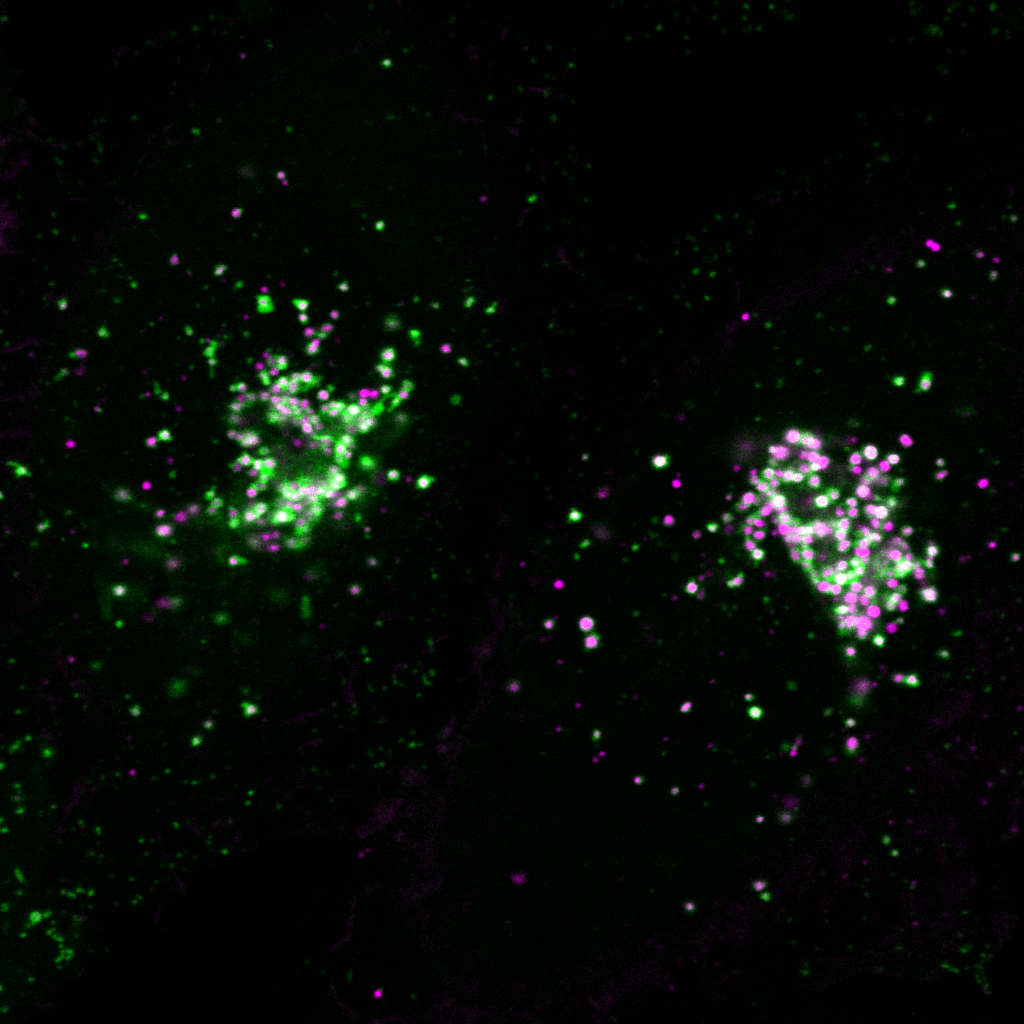

Supplement: Supplementary file 4 — Source Data for Figure 2 [file EMBR-24-e56841-s005.zip › Figure_2/2B/Image_Data/WT_15min_merge.tif]

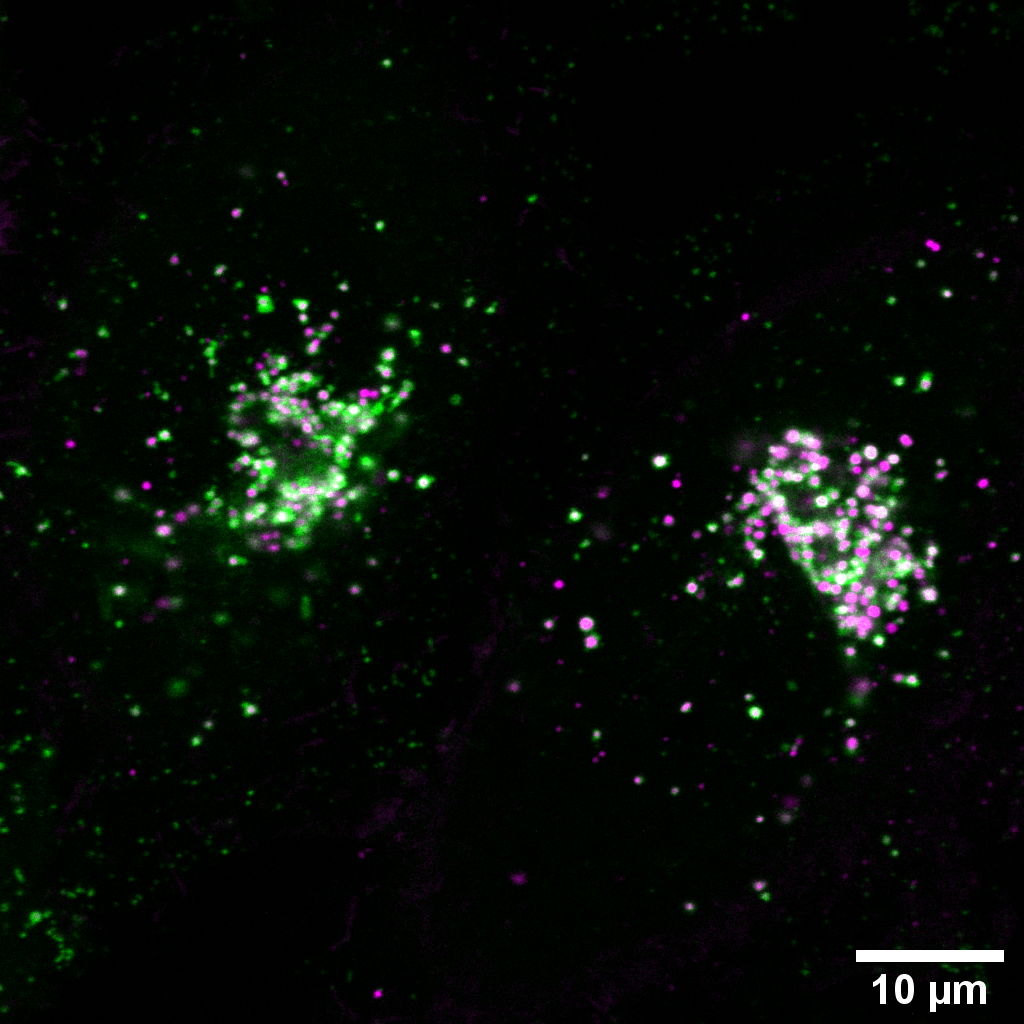

Supplement: Supplementary file 4 — Source Data for Figure 2 [file EMBR-24-e56841-s005.zip › Figure_2/2B/Image_Data/WT_15min_scale.tif]

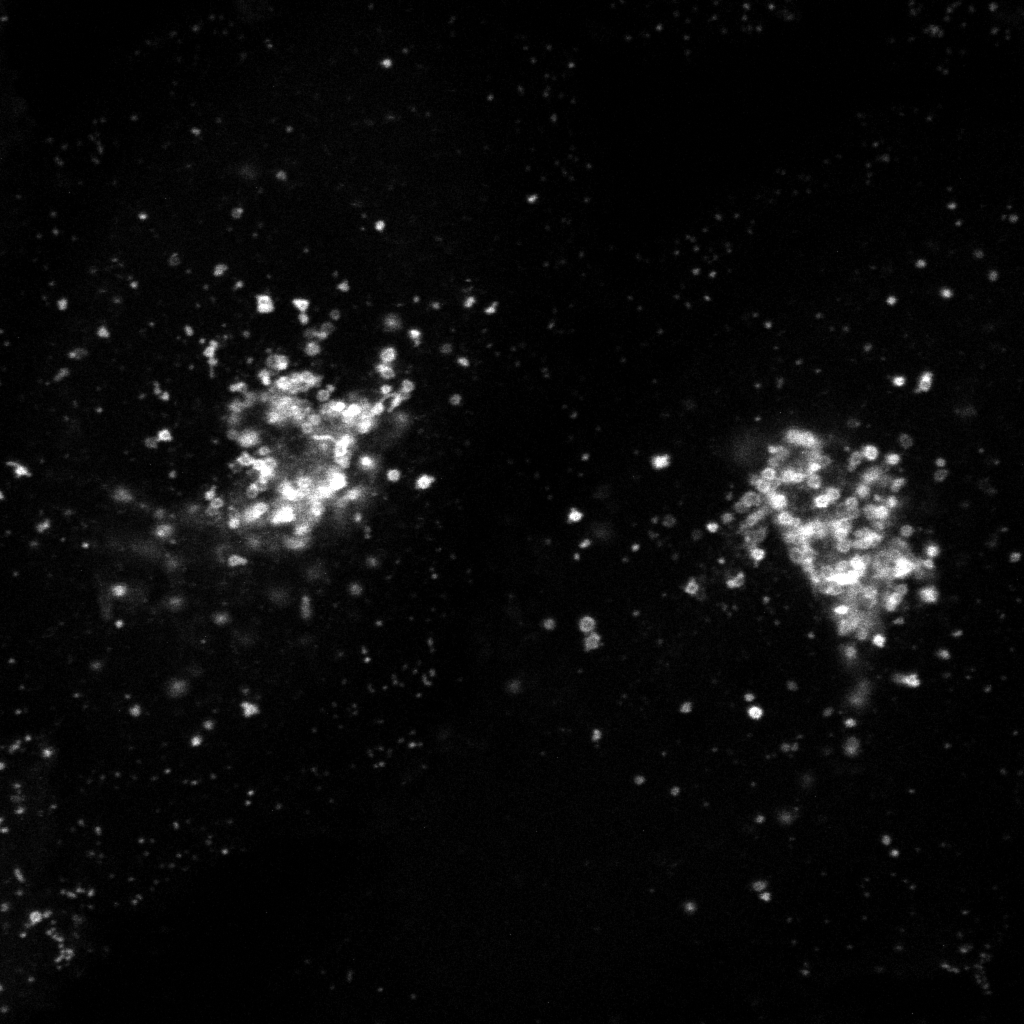

Supplement: Supplementary file 4 — Source Data for Figure 2 [file EMBR-24-e56841-s005.zip › Figure_2/2B/Image_Data/WT_15min_TECPR1.tif]

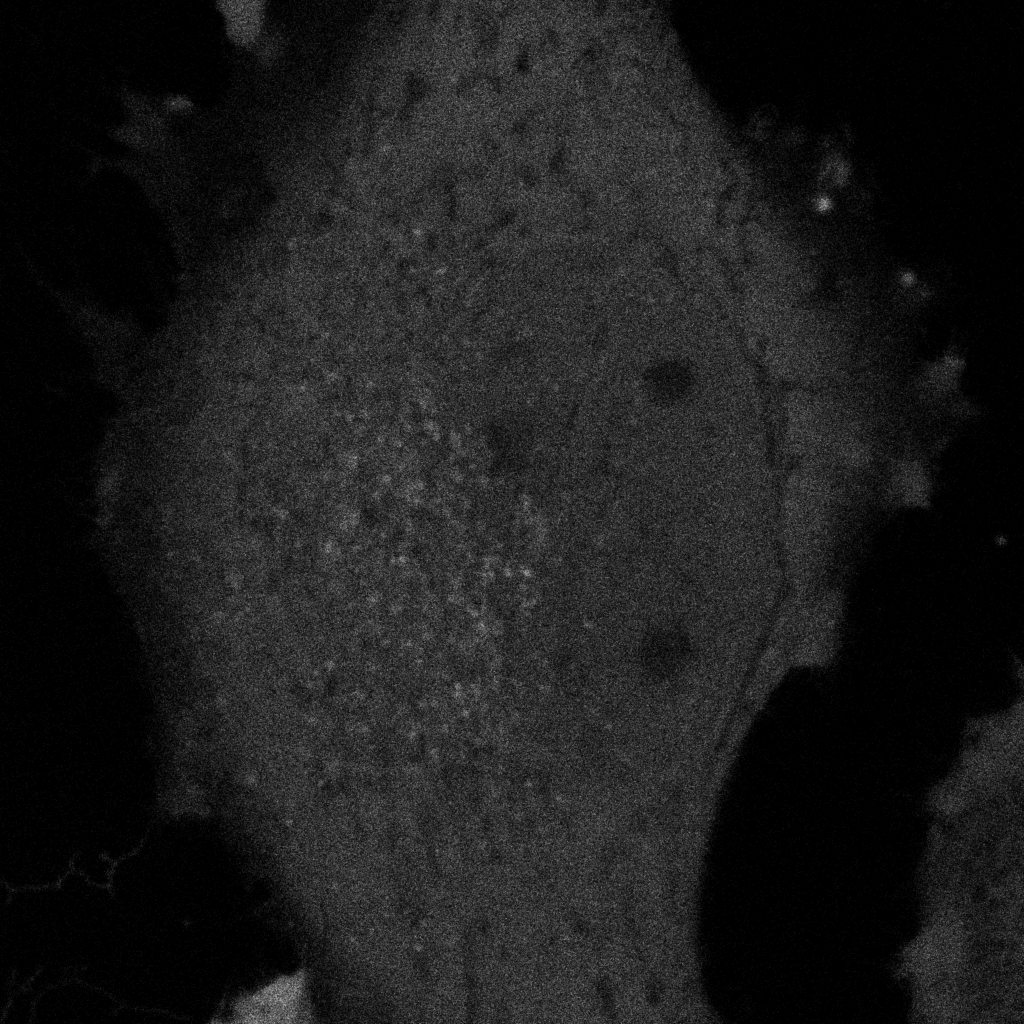

Supplement: Supplementary file 5 — Source Data for Figure 3 [file EMBR-24-e56841-s004.zip › Figure_3/3A/0_min_Gal3.tif]

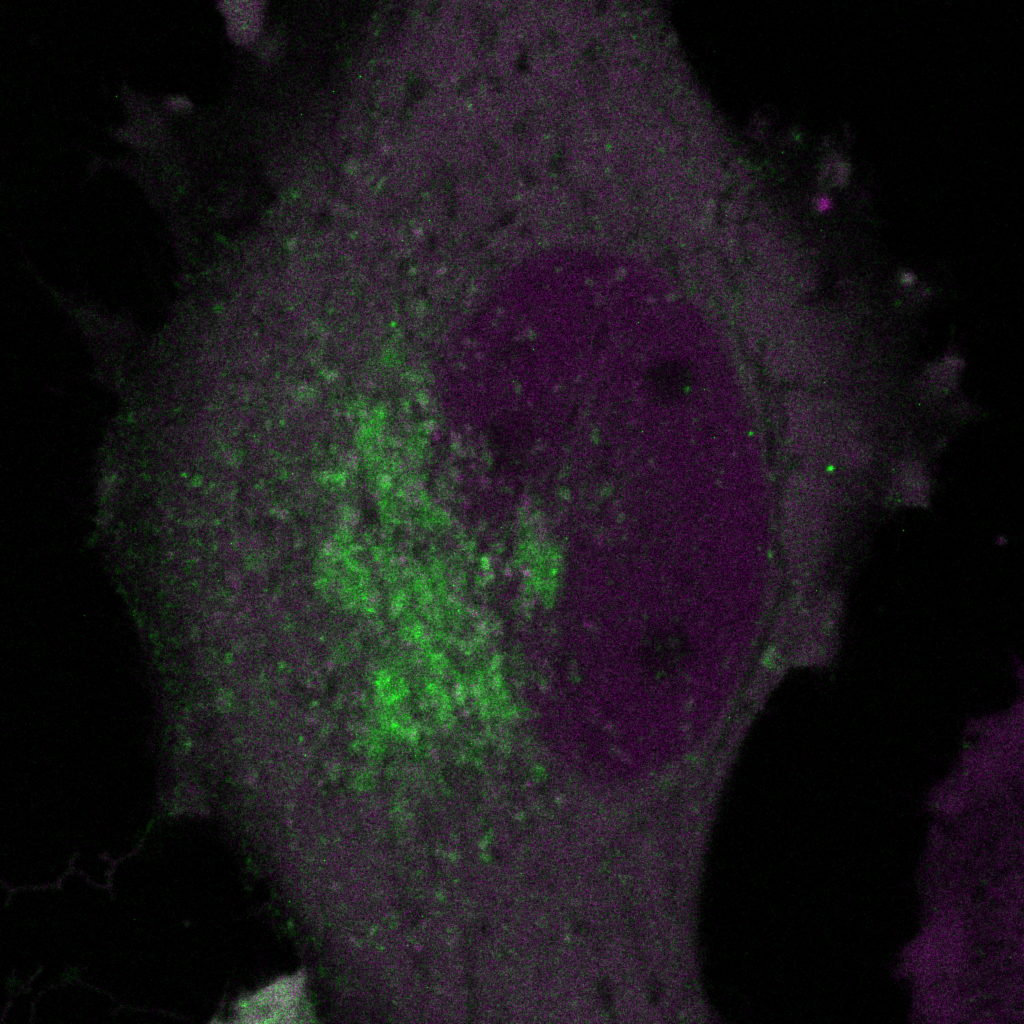

Supplement: Supplementary file 5 — Source Data for Figure 3 [file EMBR-24-e56841-s004.zip › Figure_3/3A/0_min_merge.tif]

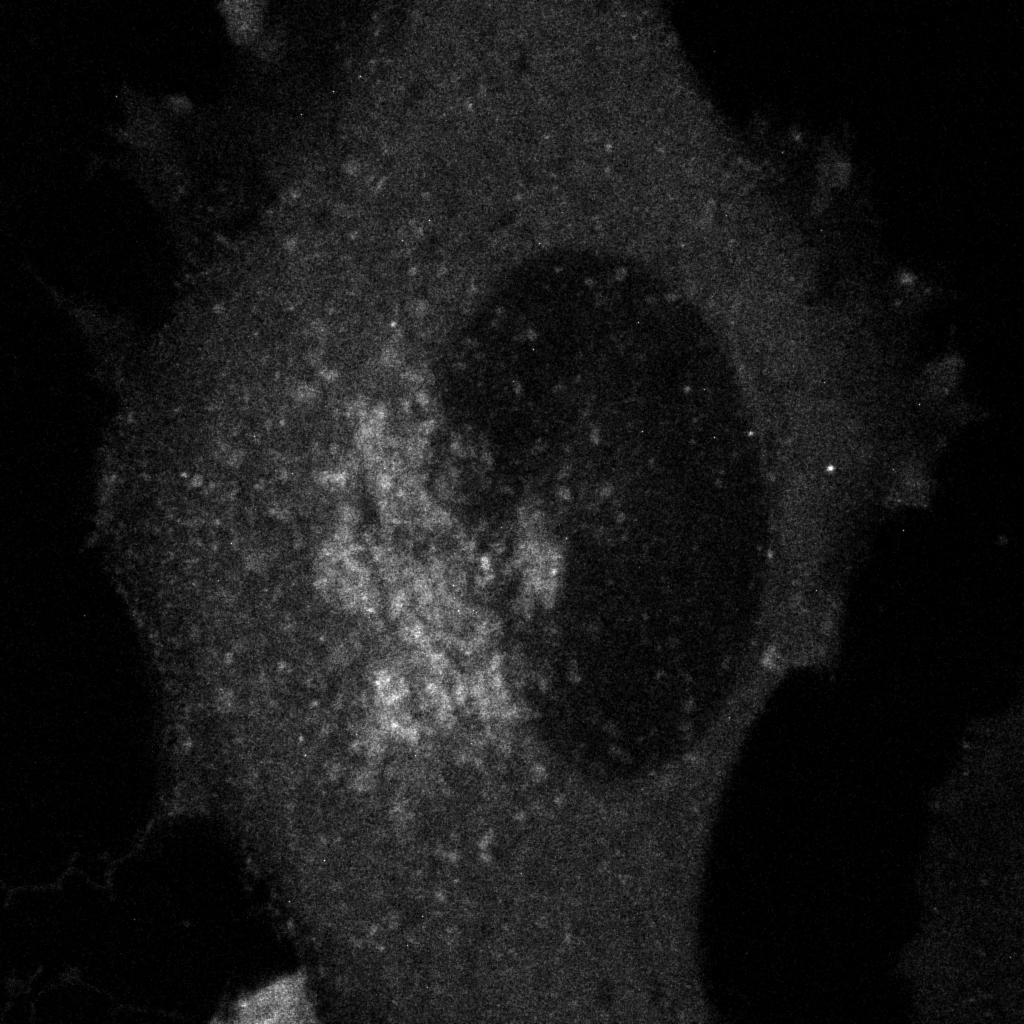

Supplement: Supplementary file 5 — Source Data for Figure 3 [file EMBR-24-e56841-s004.zip › Figure_3/3A/0_min_TECPR1.tif]

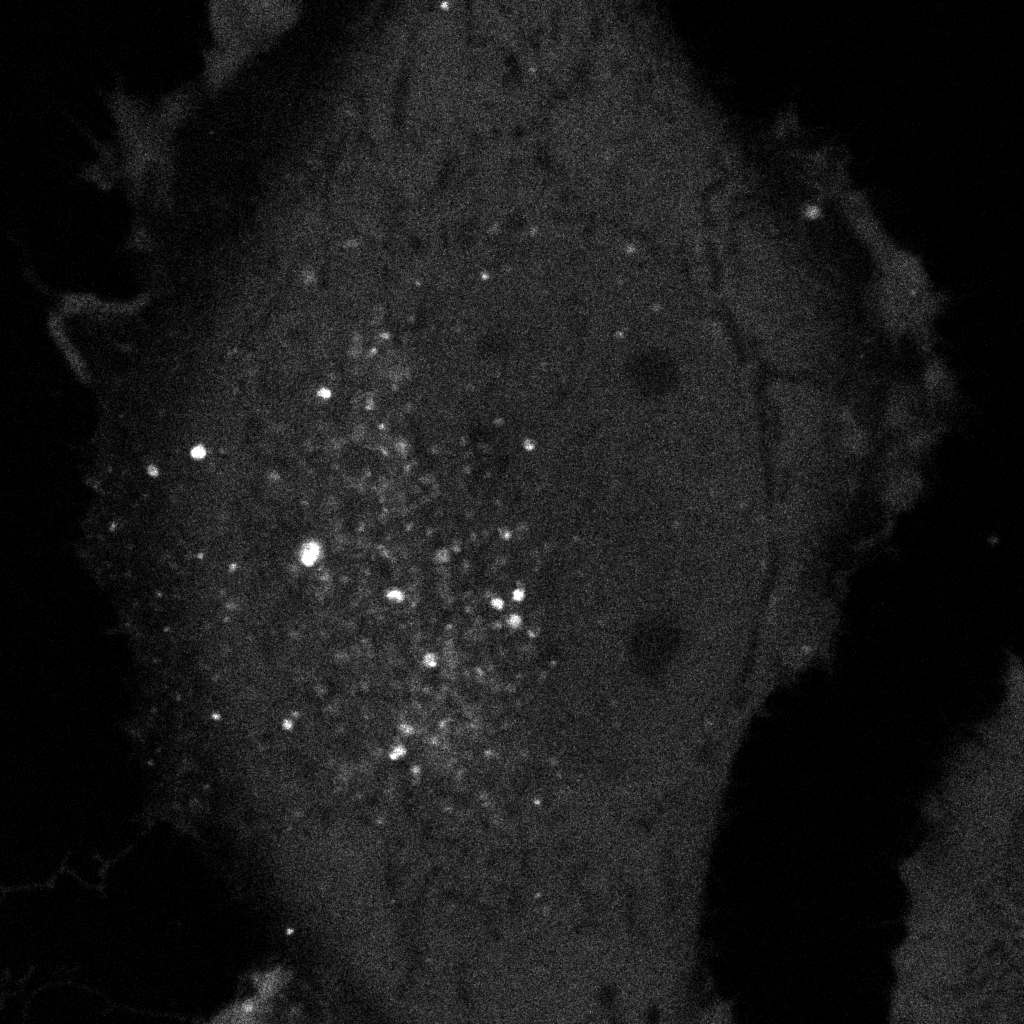

Supplement: Supplementary file 5 — Source Data for Figure 3 [file EMBR-24-e56841-s004.zip › Figure_3/3A/10-15_Gal3.tif]

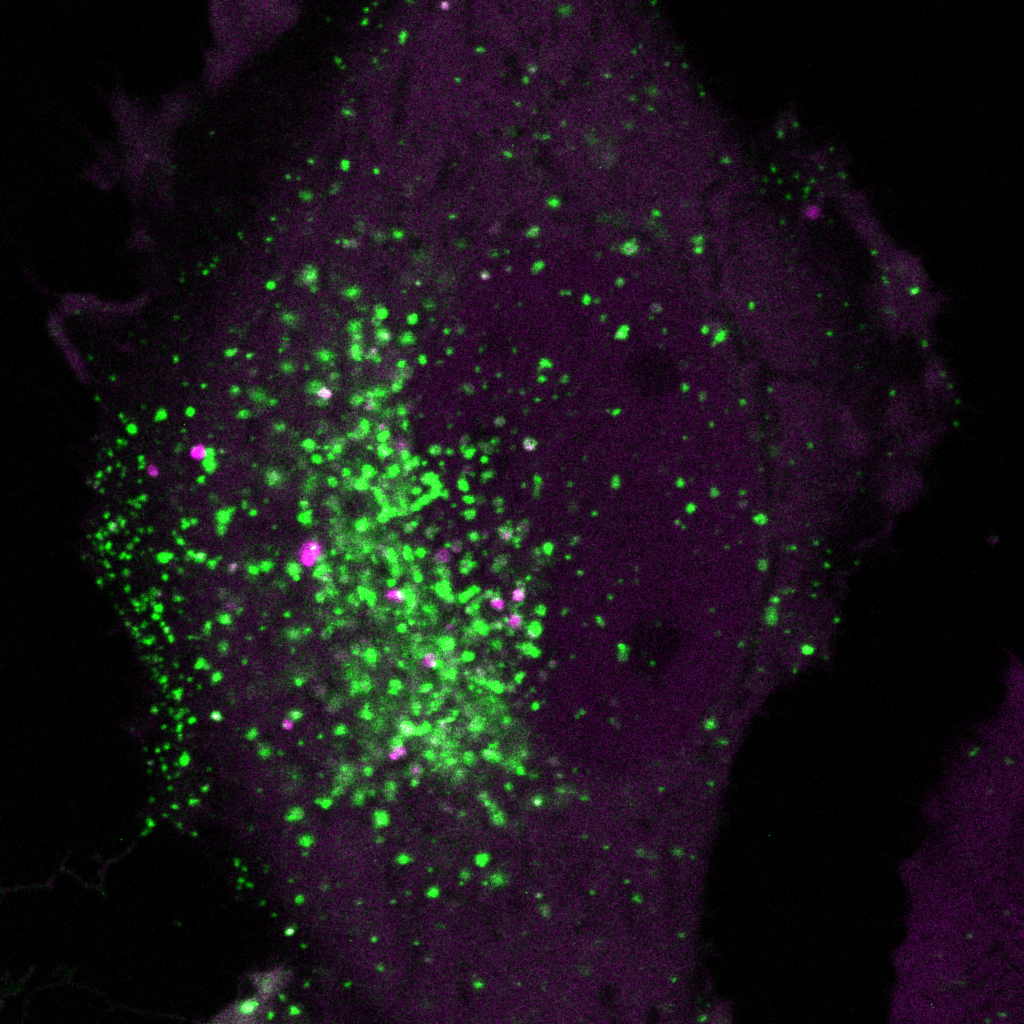

Supplement: Supplementary file 5 — Source Data for Figure 3 [file EMBR-24-e56841-s004.zip › Figure_3/3A/10-15_merge.tif]

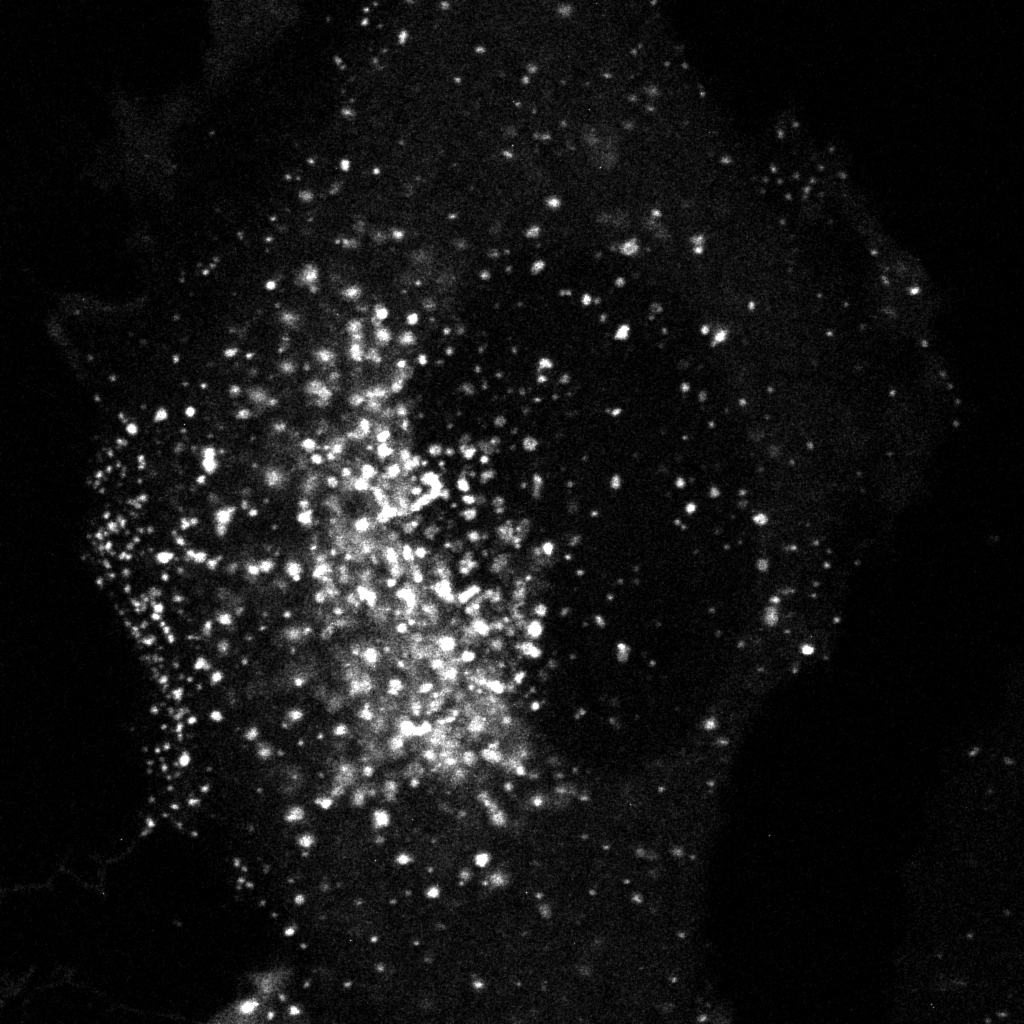

Supplement: Supplementary file 5 — Source Data for Figure 3 [file EMBR-24-e56841-s004.zip › Figure_3/3A/10-15_TECPR1.tif]

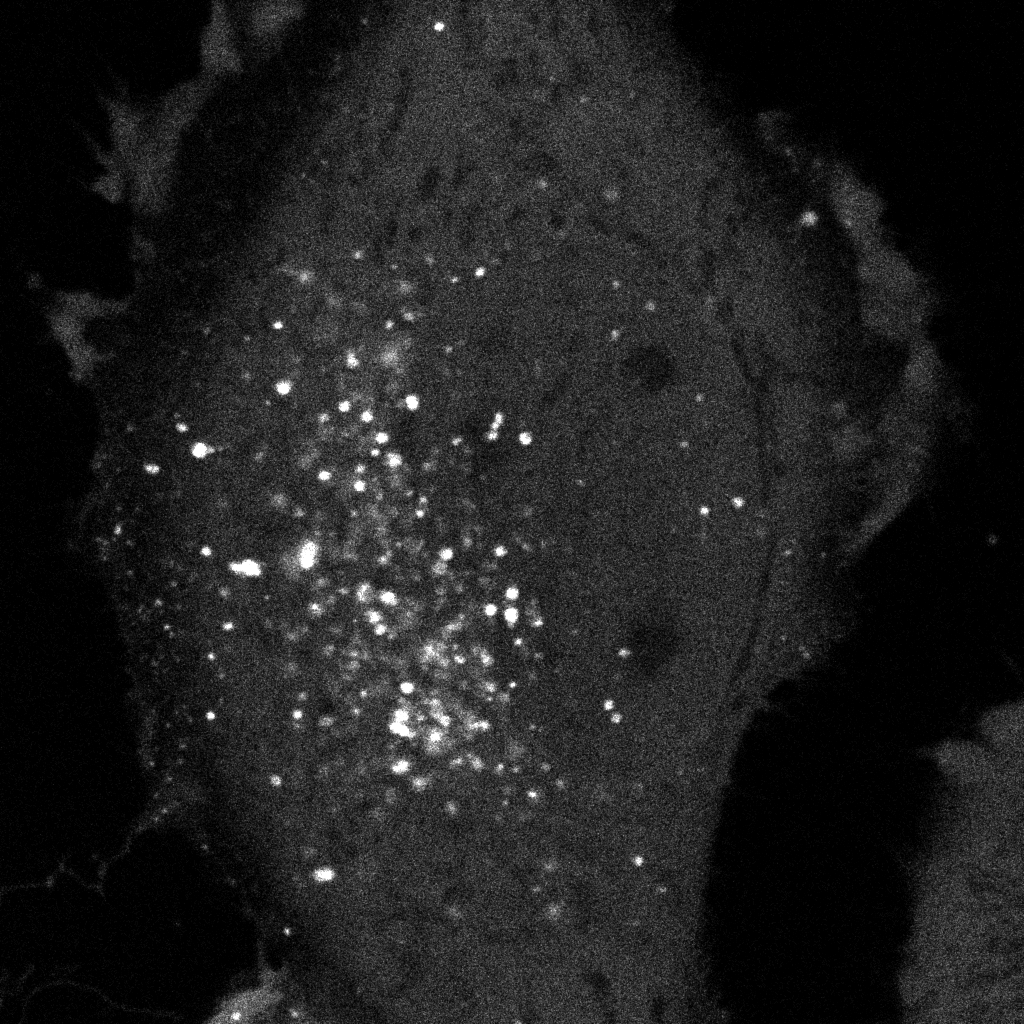

Supplement: Supplementary file 5 — Source Data for Figure 3 [file EMBR-24-e56841-s004.zip › Figure_3/3A/15-15_Gal3.tif]

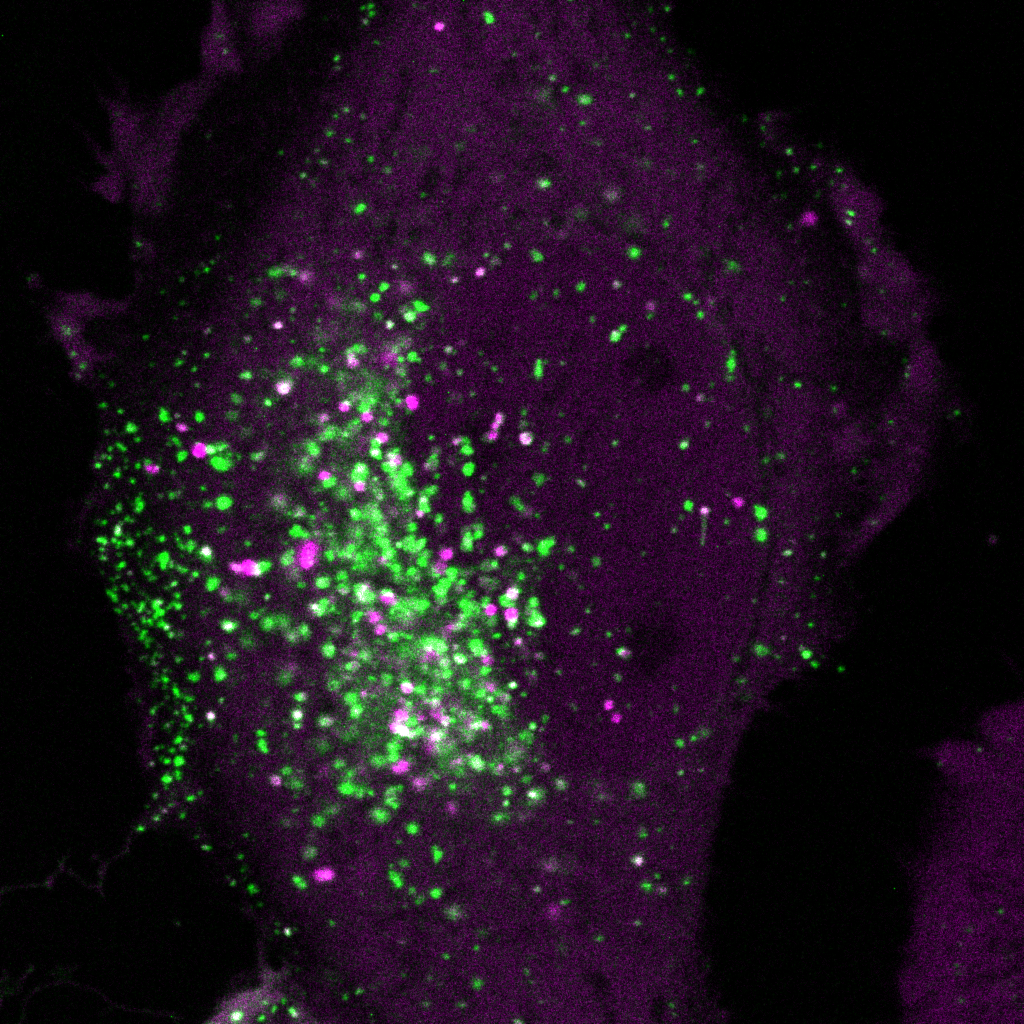

Supplement: Supplementary file 5 — Source Data for Figure 3 [file EMBR-24-e56841-s004.zip › Figure_3/3A/15-15_merge.tif]

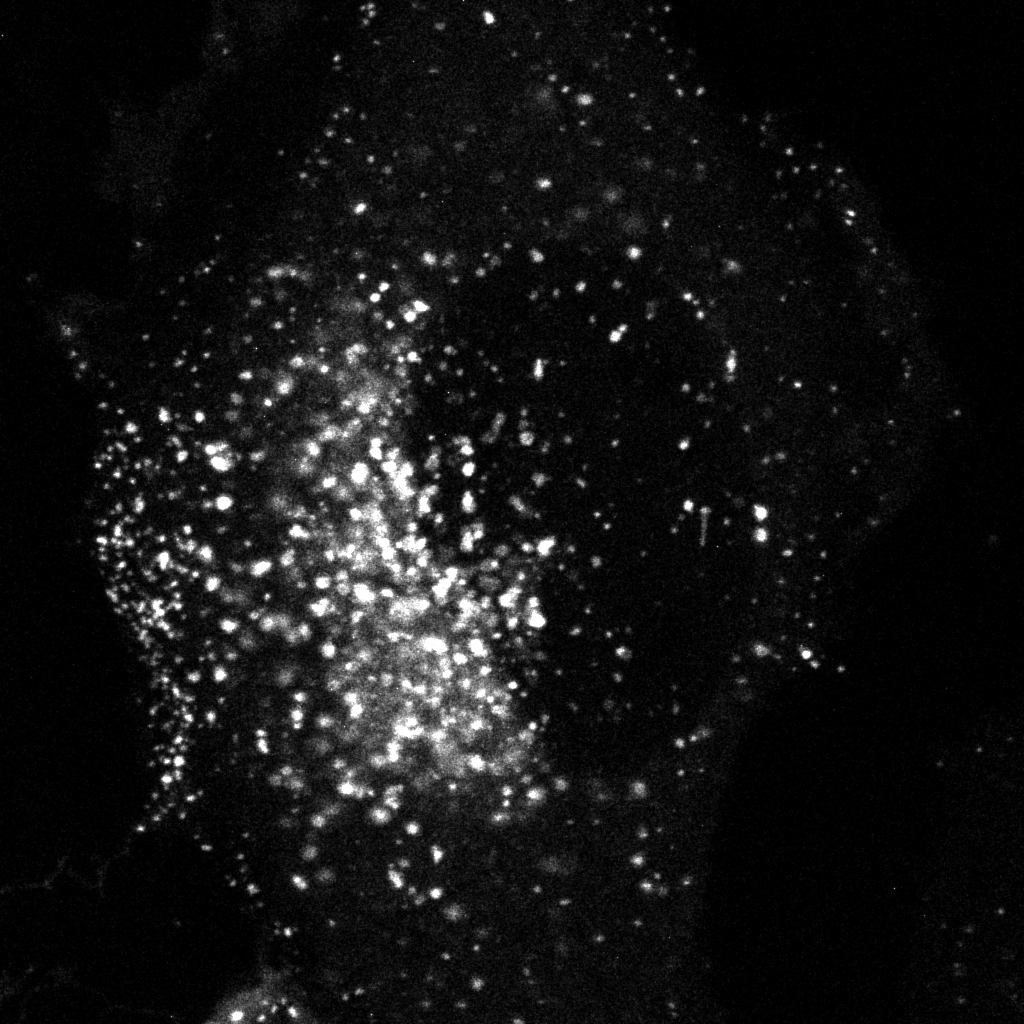

Supplement: Supplementary file 5 — Source Data for Figure 3 [file EMBR-24-e56841-s004.zip › Figure_3/3A/15-15_TECPR1.tif]

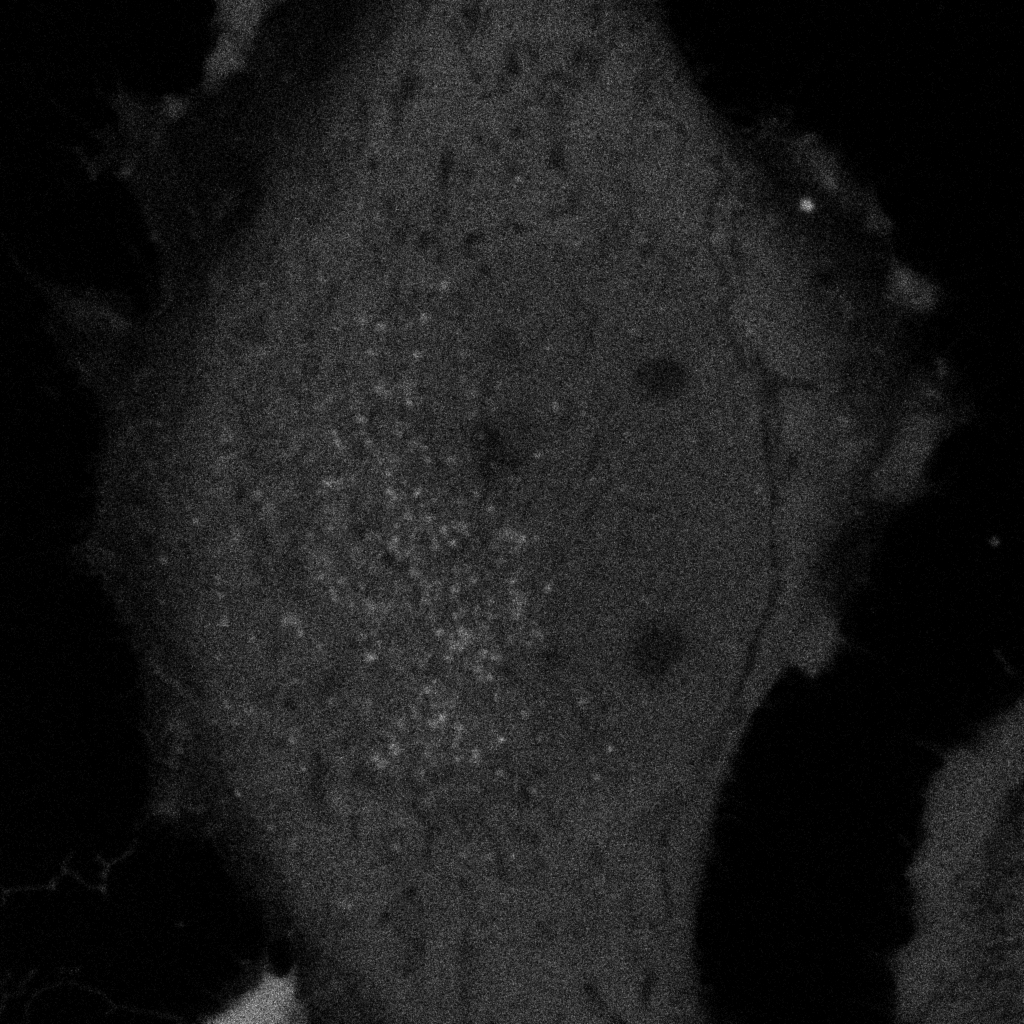

Supplement: Supplementary file 5 — Source Data for Figure 3 [file EMBR-24-e56841-s004.zip › Figure_3/3A/2-45_Gal3.tif]

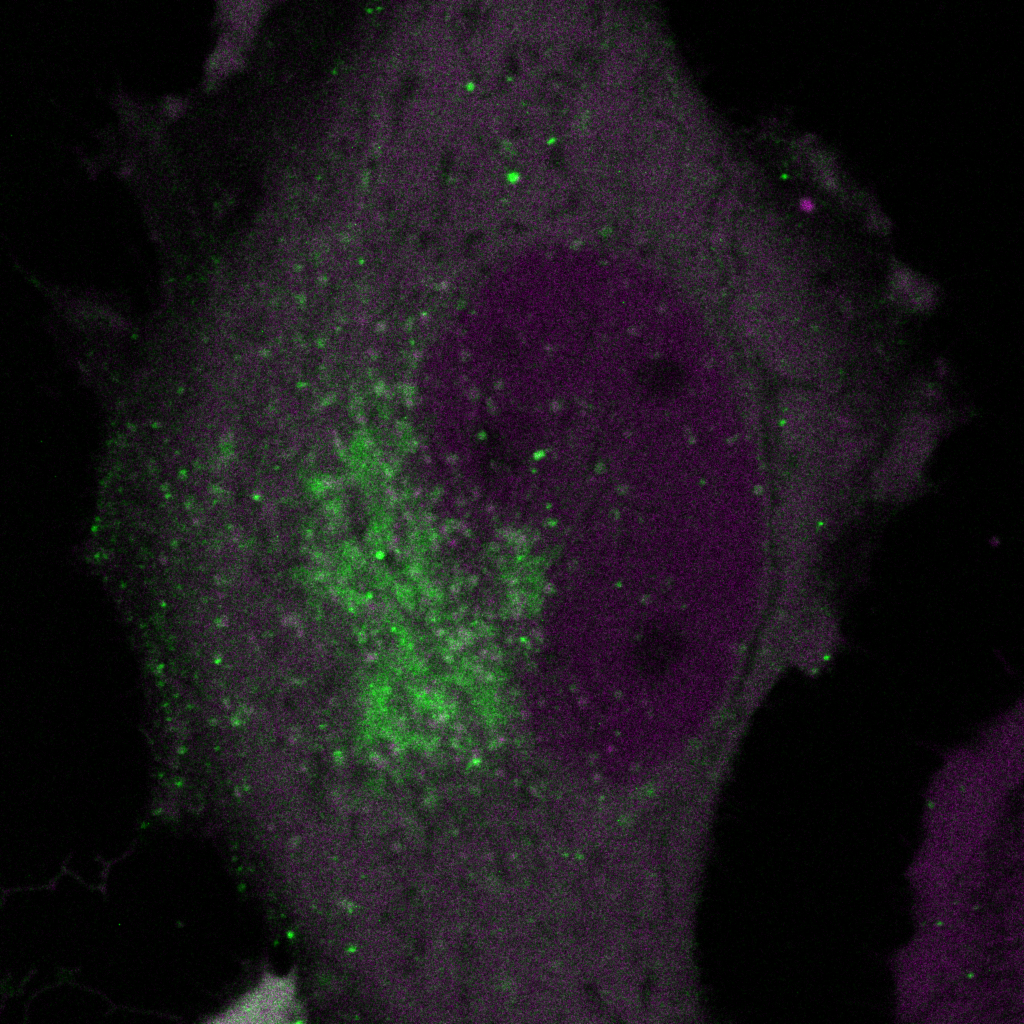

Supplement: Supplementary file 5 — Source Data for Figure 3 [file EMBR-24-e56841-s004.zip › Figure_3/3A/2-45_merge.tif]

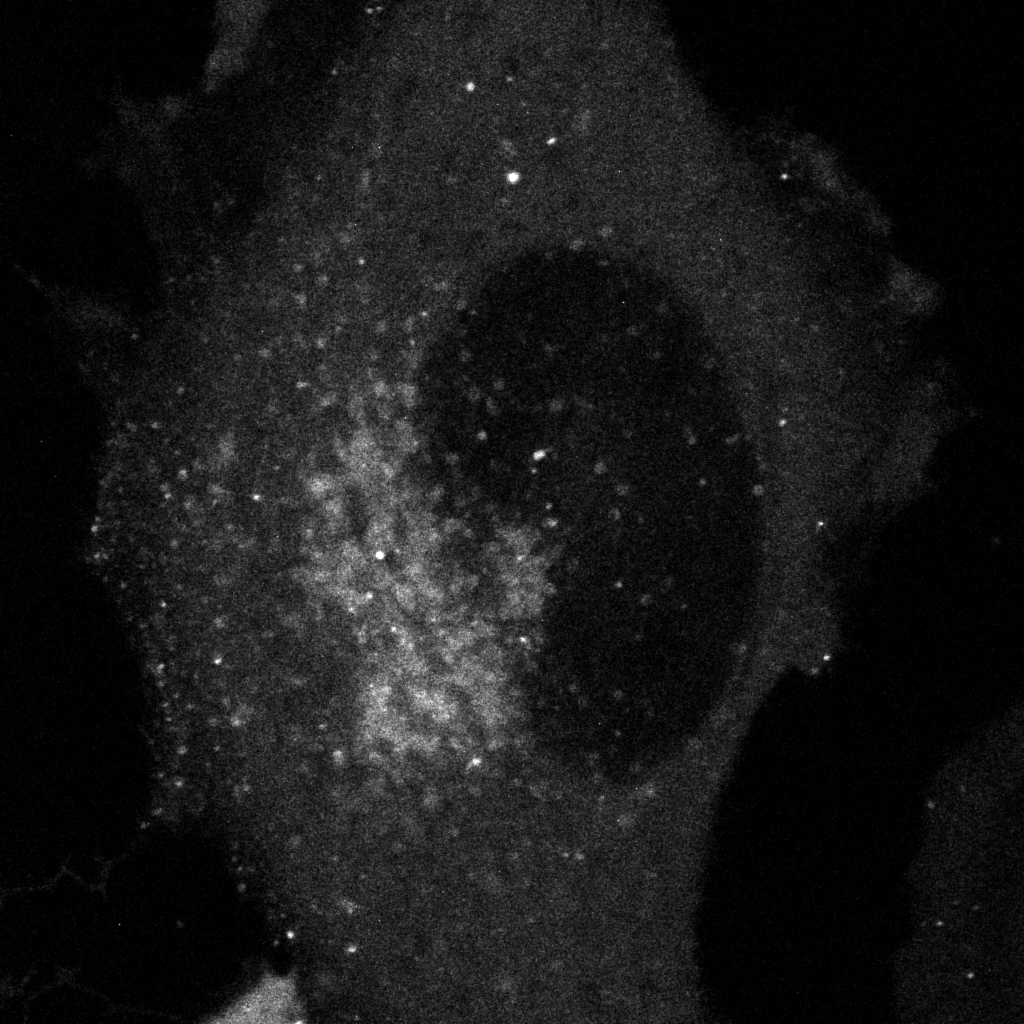

Supplement: Supplementary file 5 — Source Data for Figure 3 [file EMBR-24-e56841-s004.zip › Figure_3/3A/2-45_TECPR1.tif]

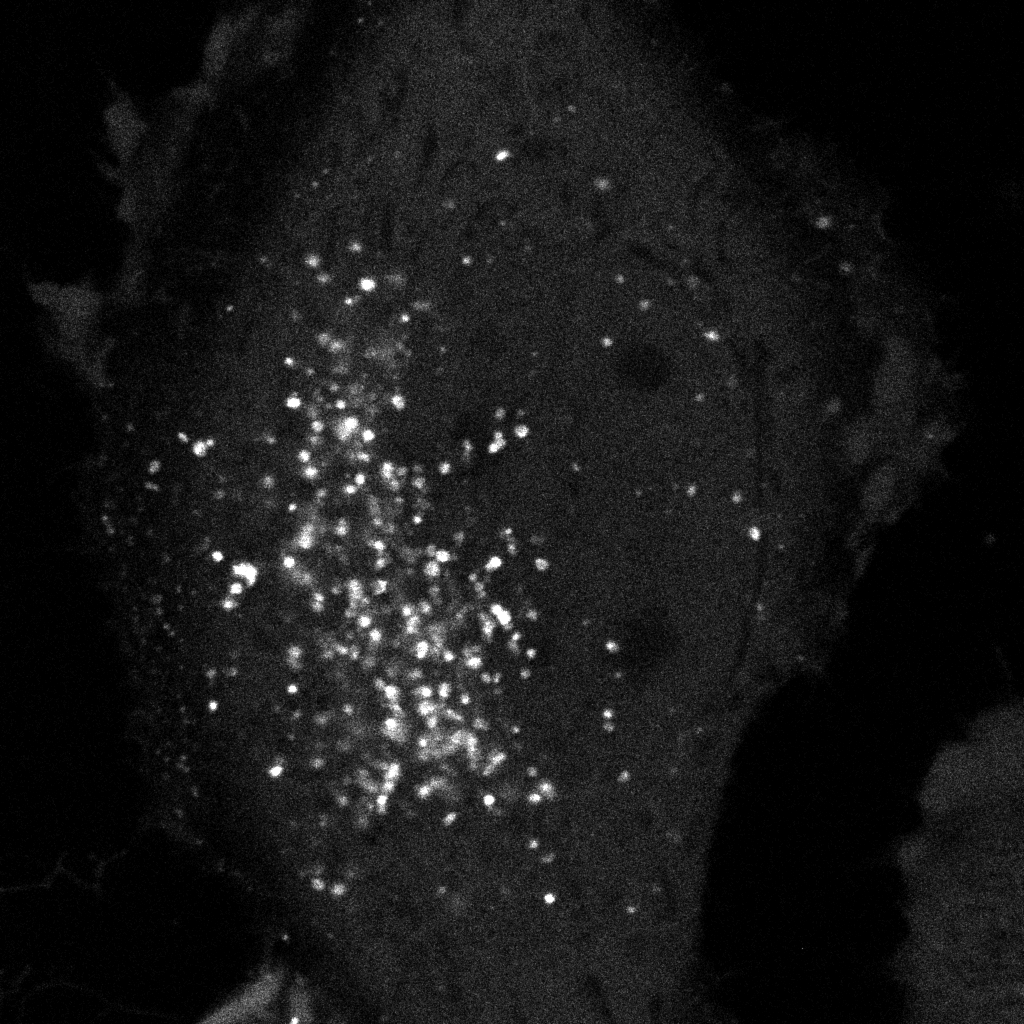

Supplement: Supplementary file 5 — Source Data for Figure 3 [file EMBR-24-e56841-s004.zip › Figure_3/3A/22-45_Gal3.tif]

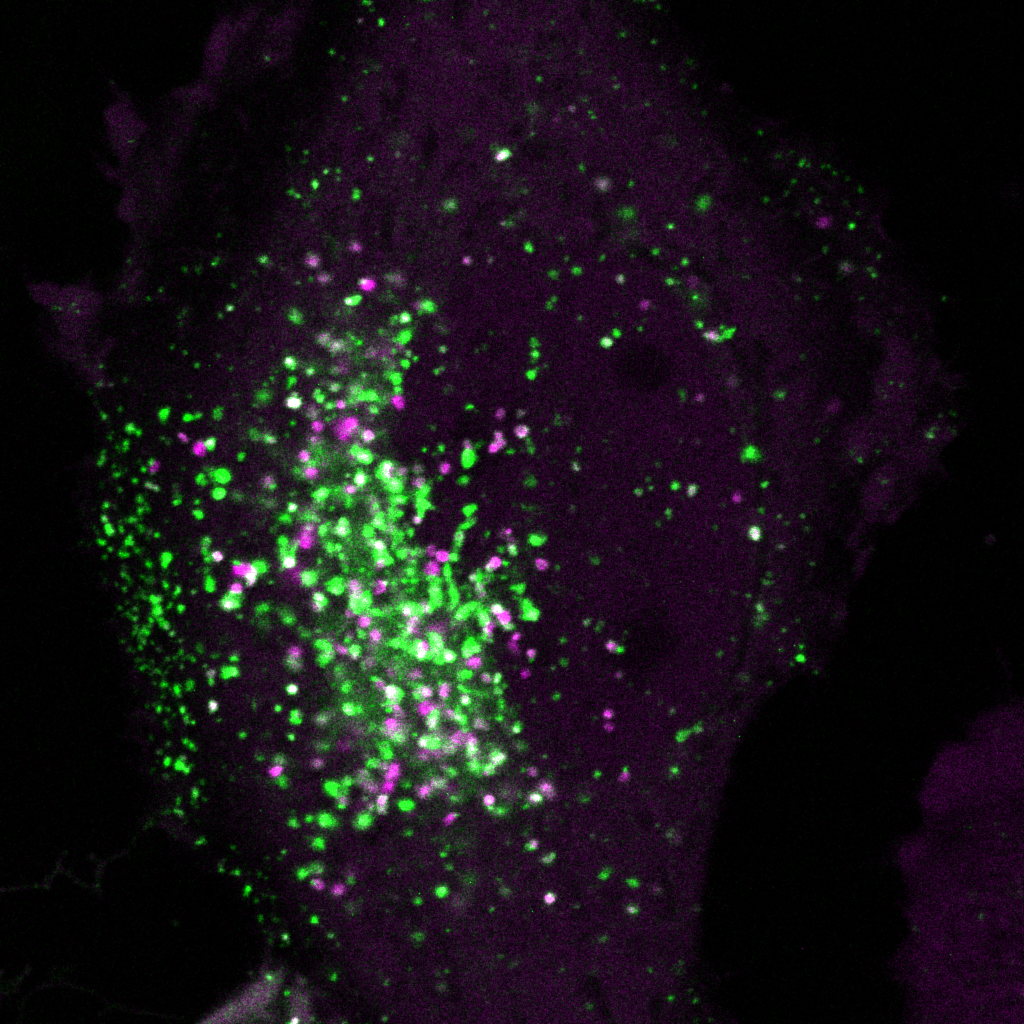

Supplement: Supplementary file 5 — Source Data for Figure 3 [file EMBR-24-e56841-s004.zip › Figure_3/3A/22-45_merge.tif]

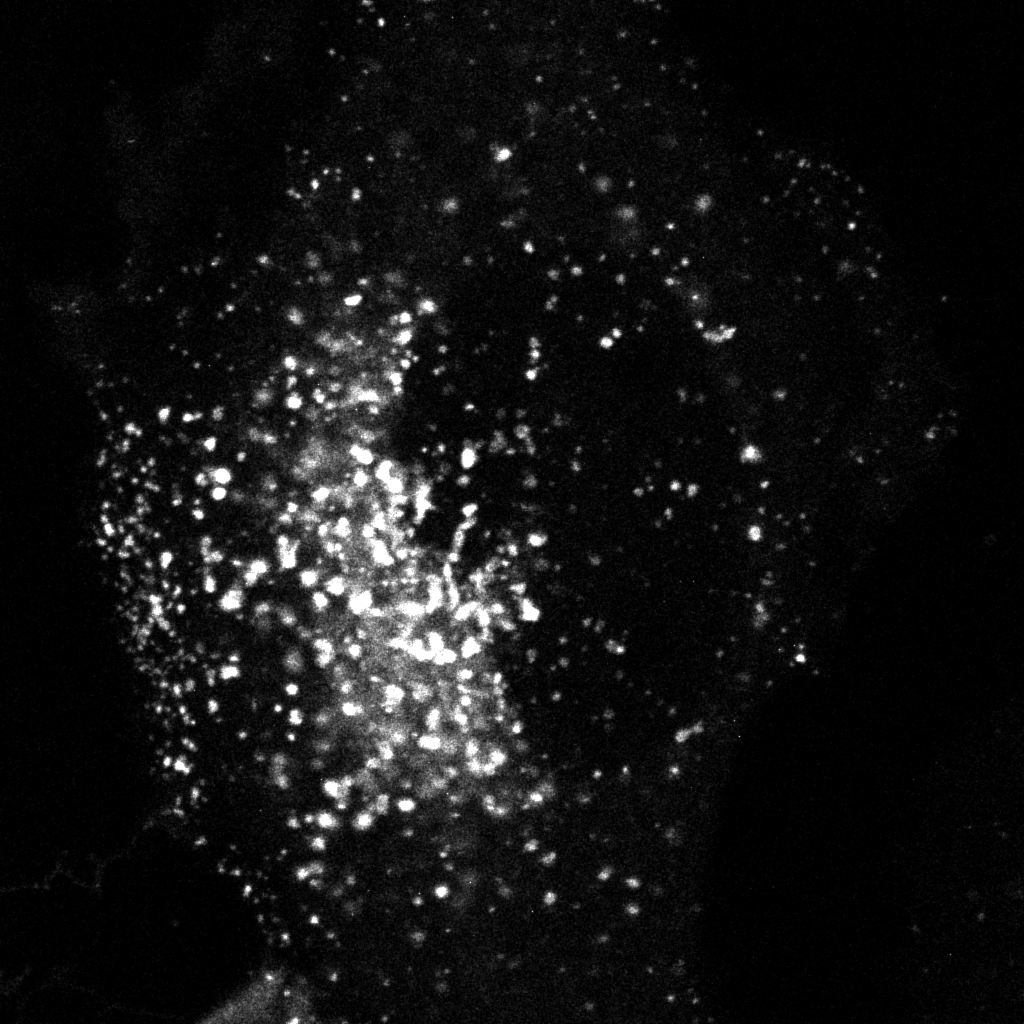

Supplement: Supplementary file 5 — Source Data for Figure 3 [file EMBR-24-e56841-s004.zip › Figure_3/3A/22-45_TECPR1.tif]

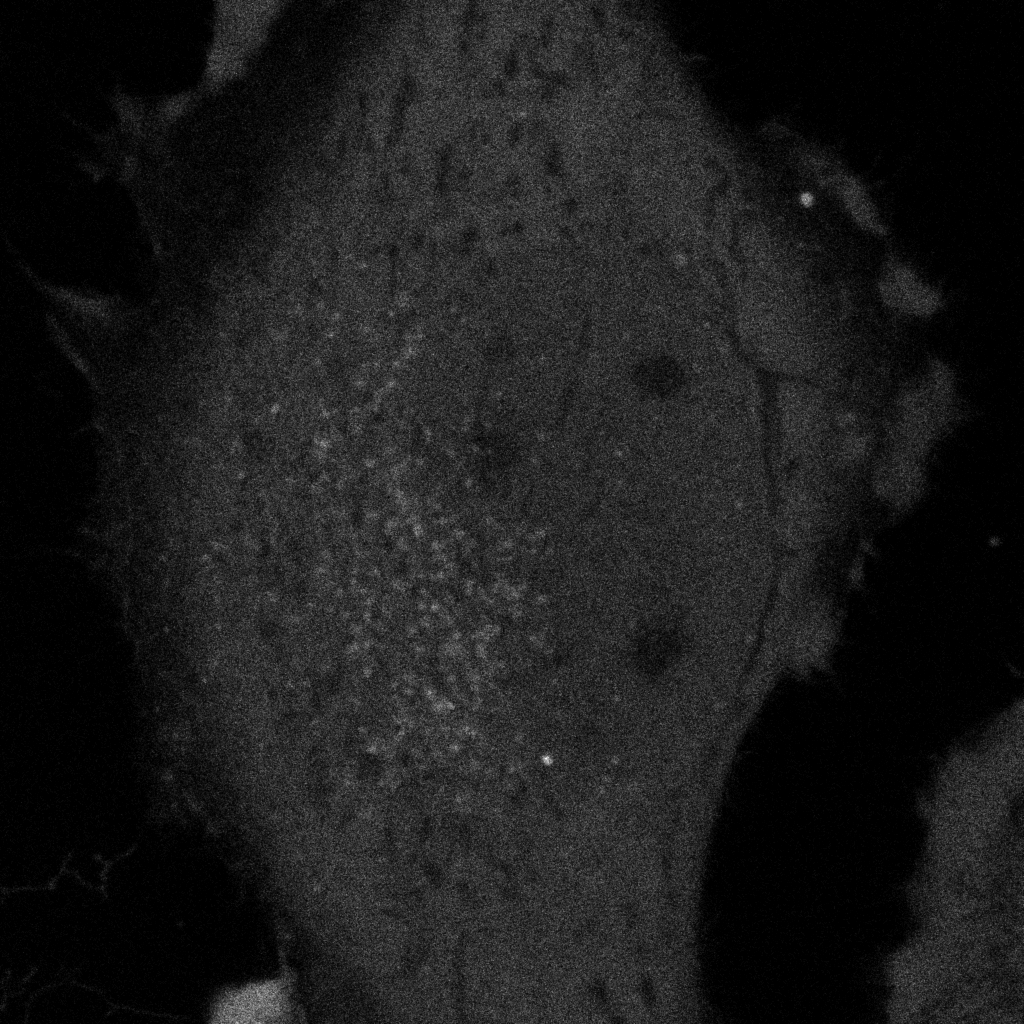

Supplement: Supplementary file 5 — Source Data for Figure 3 [file EMBR-24-e56841-s004.zip › Figure_3/3A/5-15_Gal3.tif]

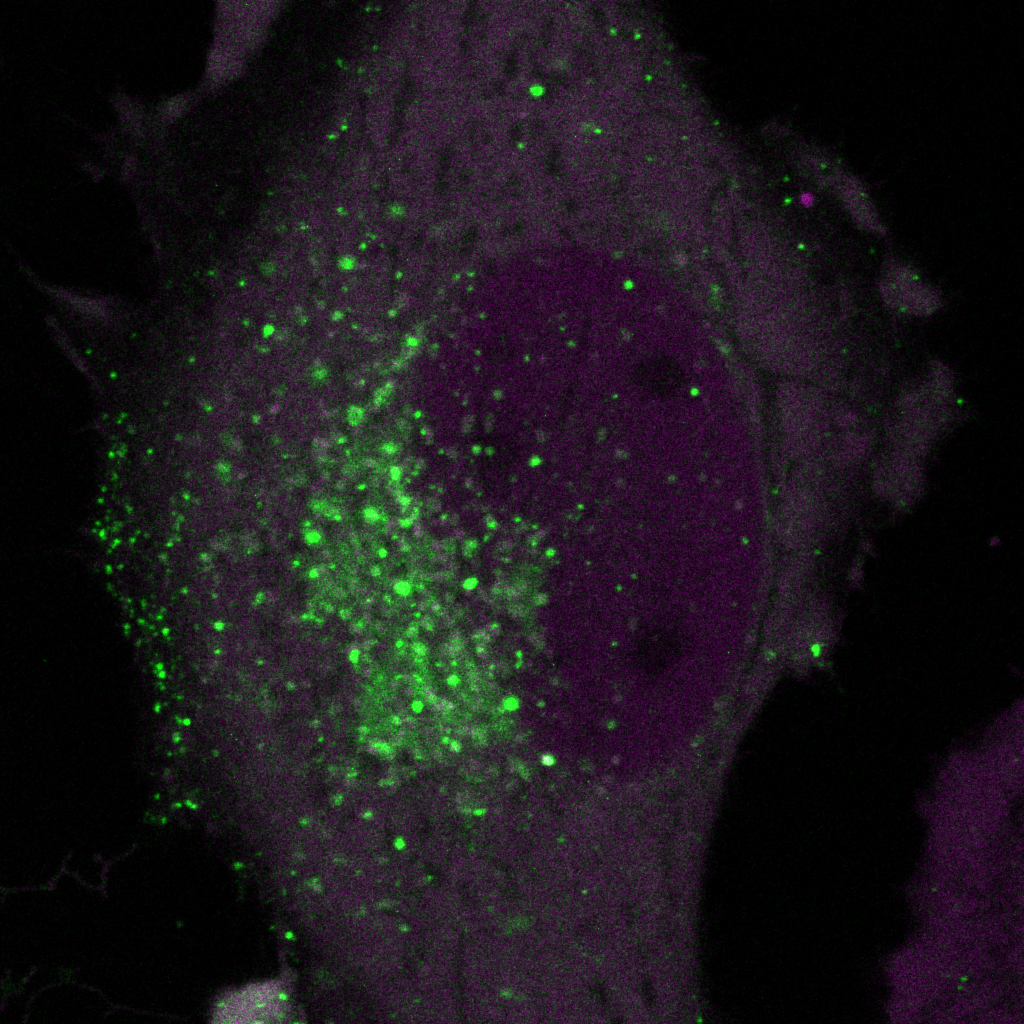

Supplement: Supplementary file 5 — Source Data for Figure 3 [file EMBR-24-e56841-s004.zip › Figure_3/3A/5-15_merge.tif]

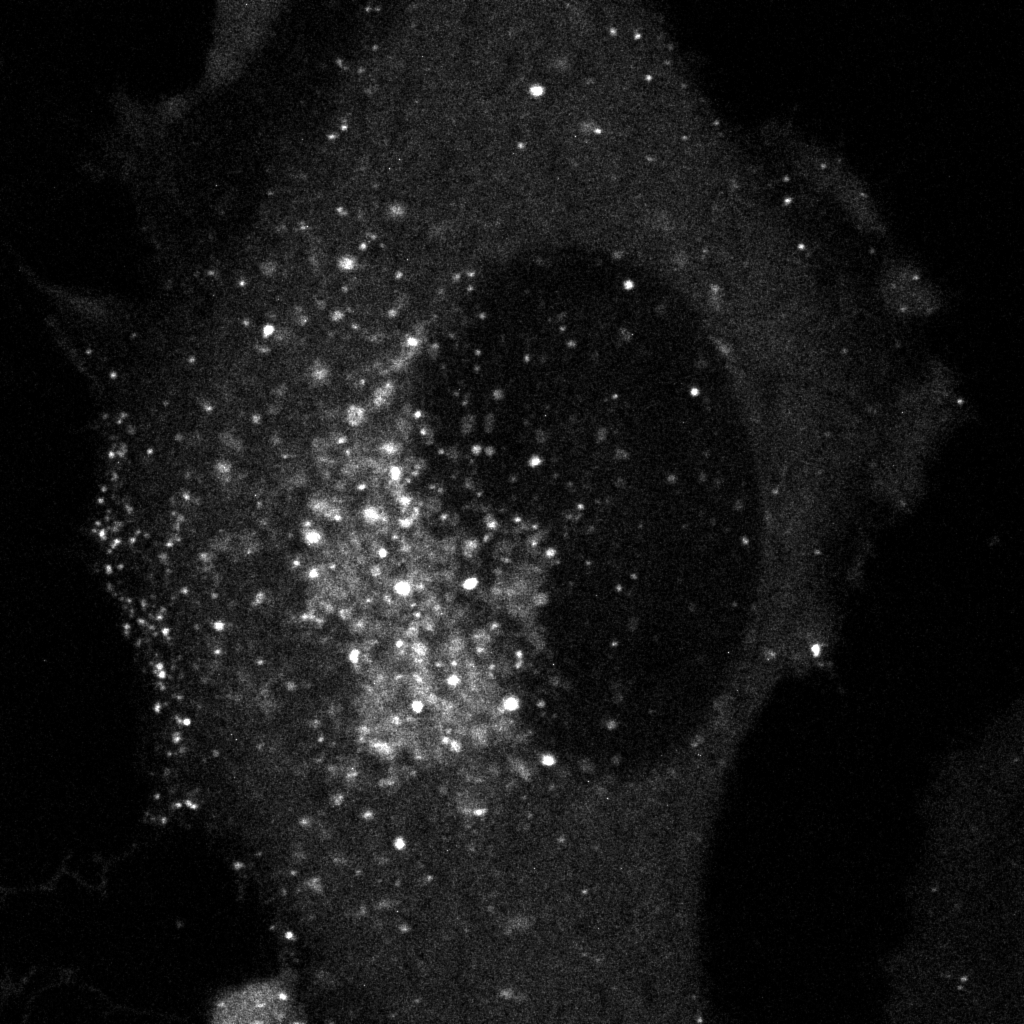

Supplement: Supplementary file 5 — Source Data for Figure 3 [file EMBR-24-e56841-s004.zip › Figure_3/3A/5-15_TECPR1.tif]

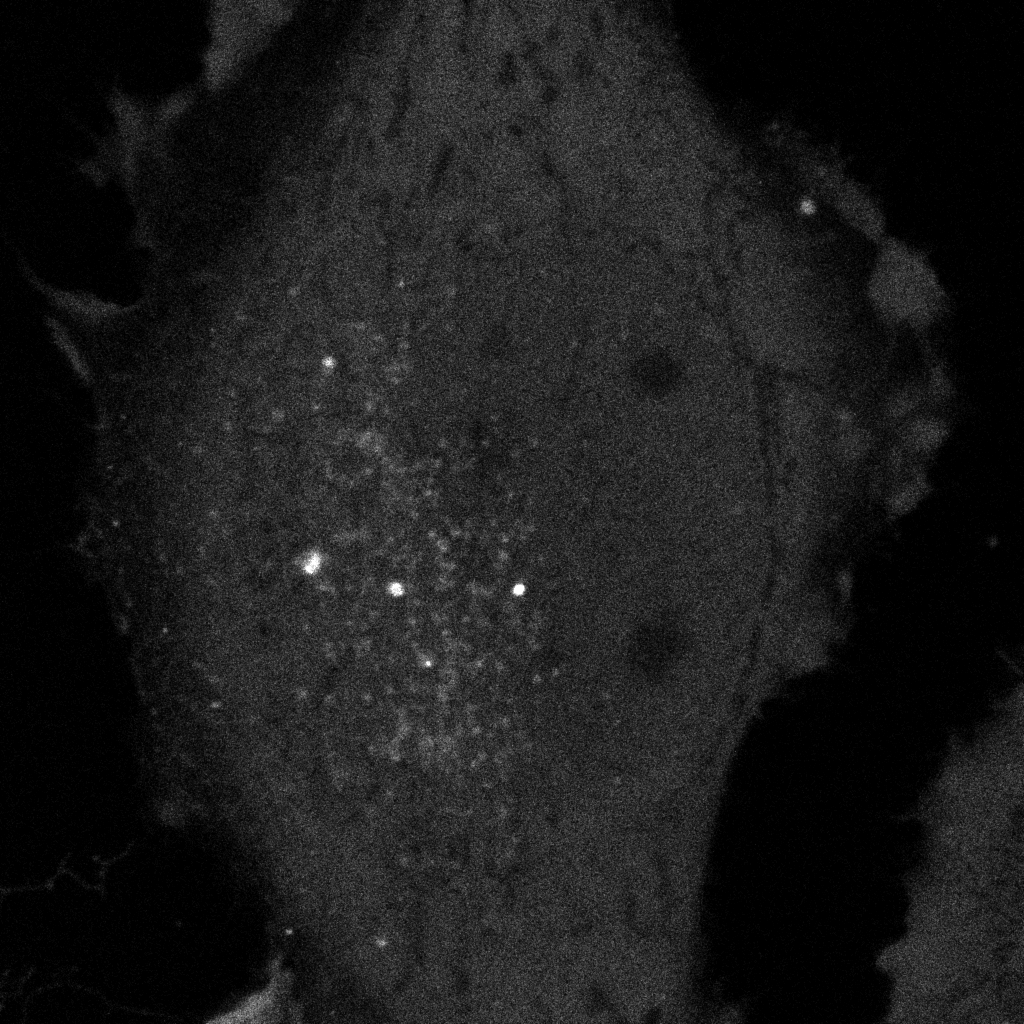

Supplement: Supplementary file 5 — Source Data for Figure 3 [file EMBR-24-e56841-s004.zip › Figure_3/3A/7-30_Gal3.tif]

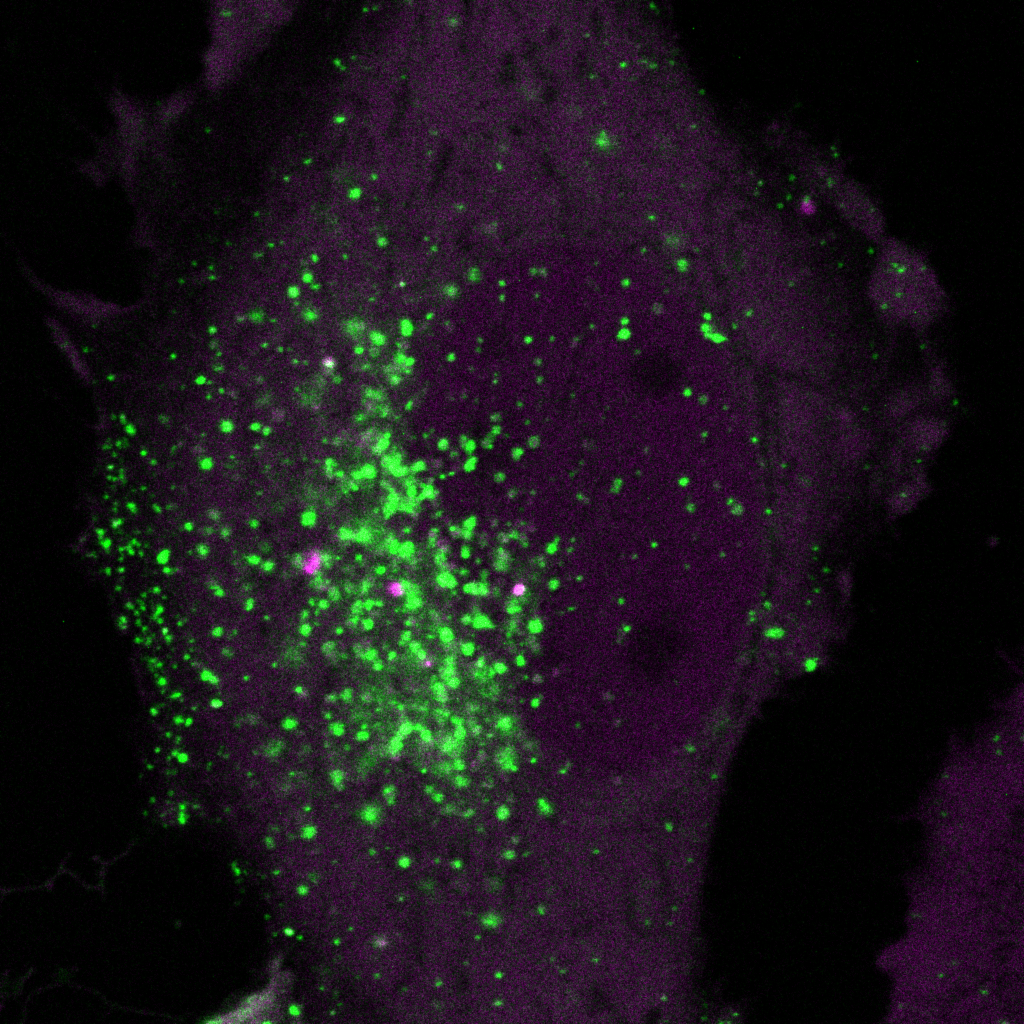

Supplement: Supplementary file 5 — Source Data for Figure 3 [file EMBR-24-e56841-s004.zip › Figure_3/3A/7-30_merge.tif]

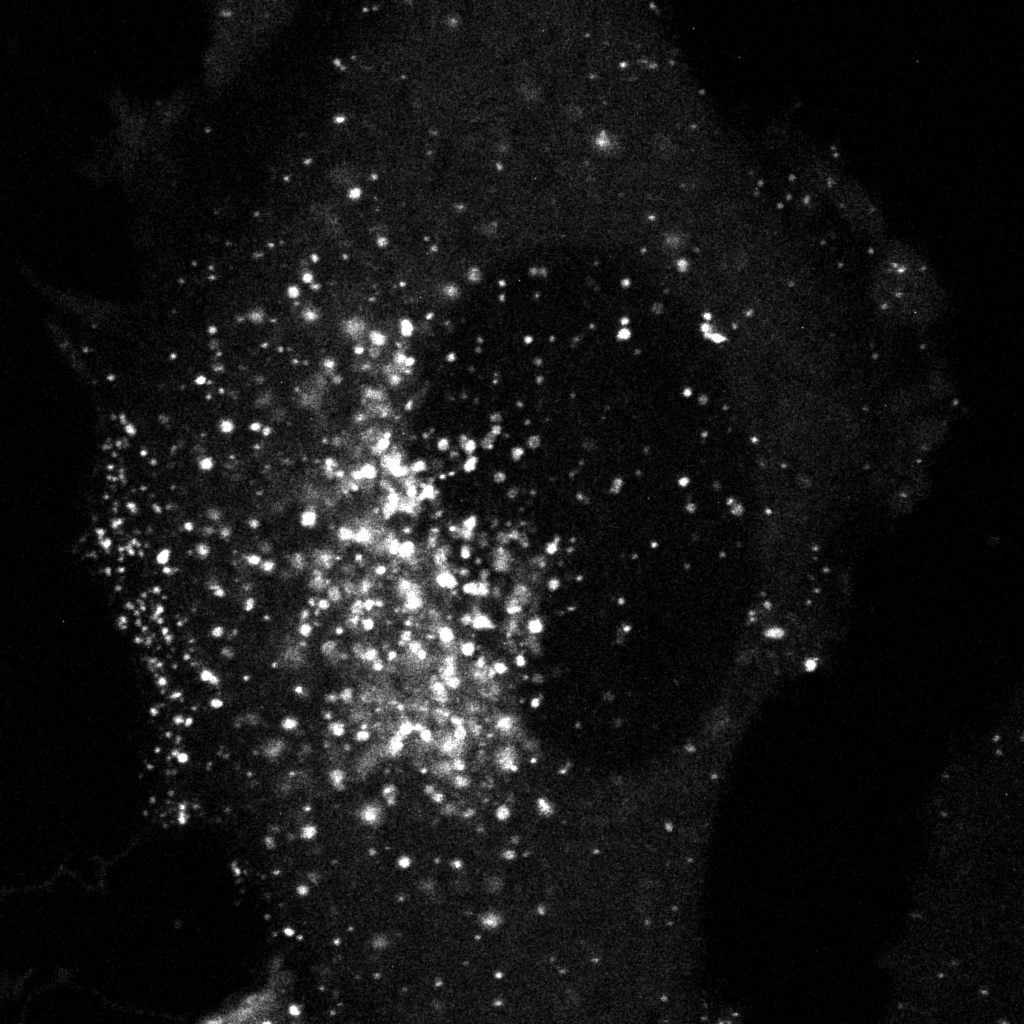

Supplement: Supplementary file 5 — Source Data for Figure 3 [file EMBR-24-e56841-s004.zip › Figure_3/3A/7-30_TECPR1.tif]

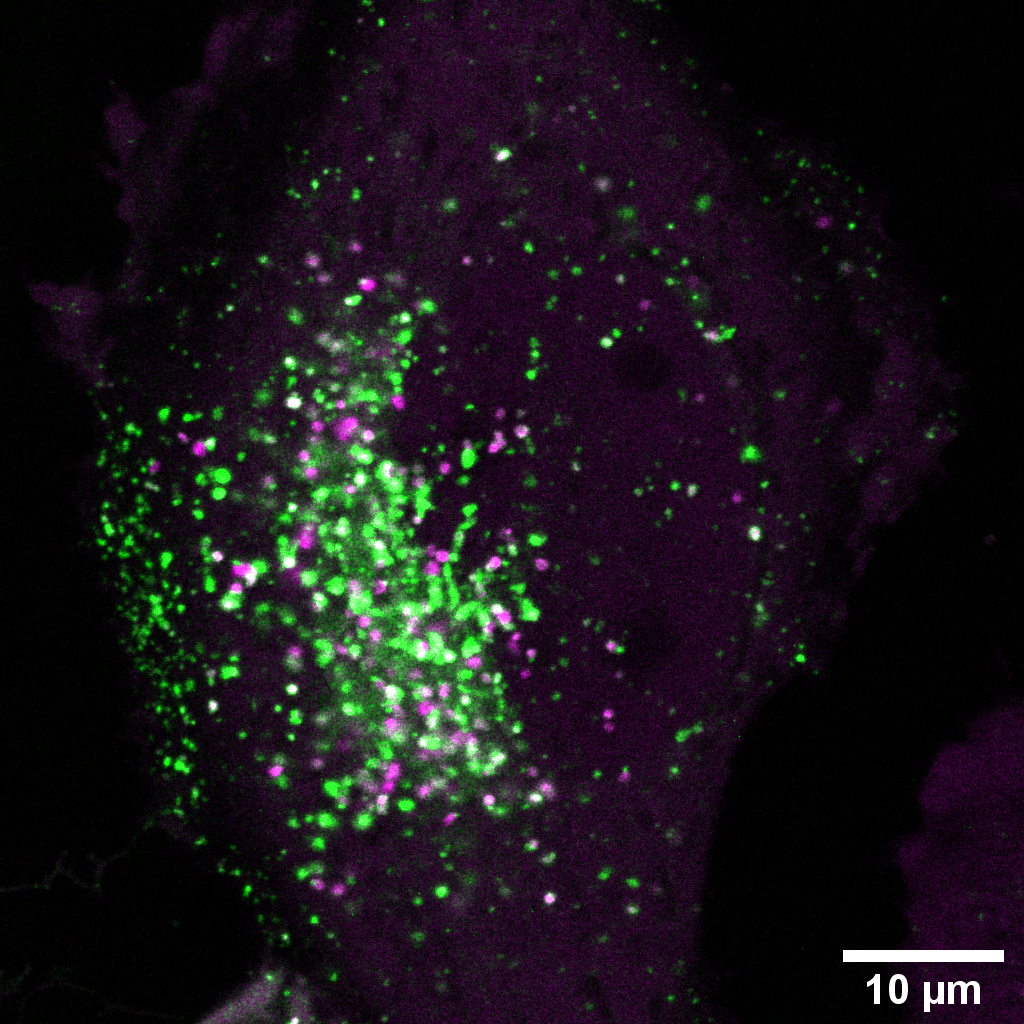

Supplement: Supplementary file 5 — Source Data for Figure 3 [file EMBR-24-e56841-s004.zip › Figure_3/3A/scale.tif]

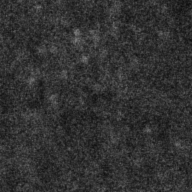

Supplement: Supplementary file 5 — Source Data for Figure 3 [file EMBR-24-e56841-s004.zip › Figure_3/3A/zoom/0min_Gal3.tif]

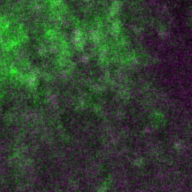

Supplement: Supplementary file 5 — Source Data for Figure 3 [file EMBR-24-e56841-s004.zip › Figure_3/3A/zoom/0min_merge.tif]

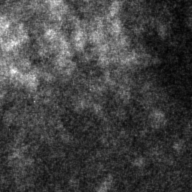

Supplement: Supplementary file 5 — Source Data for Figure 3 [file EMBR-24-e56841-s004.zip › Figure_3/3A/zoom/0min_TECPR1.tif]

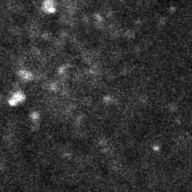

Supplement: Supplementary file 5 — Source Data for Figure 3 [file EMBR-24-e56841-s004.zip › Figure_3/3A/zoom/10_15_Gal3.tif]

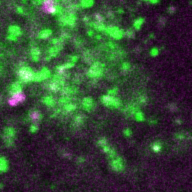

Supplement: Supplementary file 5 — Source Data for Figure 3 [file EMBR-24-e56841-s004.zip › Figure_3/3A/zoom/10_15_merge.tif]

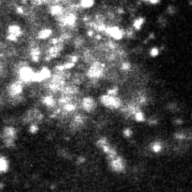

Supplement: Supplementary file 5 — Source Data for Figure 3 [file EMBR-24-e56841-s004.zip › Figure_3/3A/zoom/10_15_TECPR1.tif]

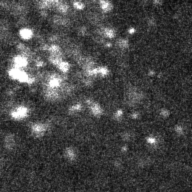

Supplement: Supplementary file 5 — Source Data for Figure 3 [file EMBR-24-e56841-s004.zip › Figure_3/3A/zoom/15_15_Gal3.tif]

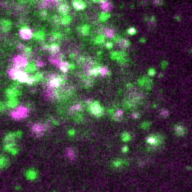

Supplement: Supplementary file 5 — Source Data for Figure 3 [file EMBR-24-e56841-s004.zip › Figure_3/3A/zoom/15_15_merge.tif]

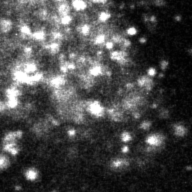

Supplement: Supplementary file 5 — Source Data for Figure 3 [file EMBR-24-e56841-s004.zip › Figure_3/3A/zoom/15_15_TECPR1.tif]

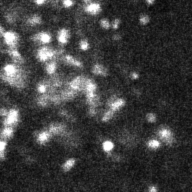

Supplement: Supplementary file 5 — Source Data for Figure 3 [file EMBR-24-e56841-s004.zip › Figure_3/3A/zoom/20_45_Gal3.tif]

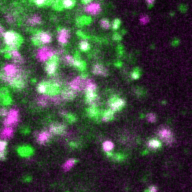

Supplement: Supplementary file 5 — Source Data for Figure 3 [file EMBR-24-e56841-s004.zip › Figure_3/3A/zoom/20_45_merge.tif]

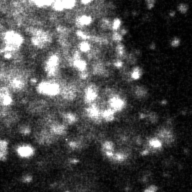

Supplement: Supplementary file 5 — Source Data for Figure 3 [file EMBR-24-e56841-s004.zip › Figure_3/3A/zoom/20_45_TECPR1.tif]

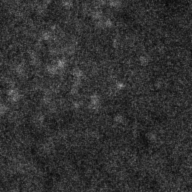

Supplement: Supplementary file 5 — Source Data for Figure 3 [file EMBR-24-e56841-s004.zip › Figure_3/3A/zoom/2_45_Gal3.tif]

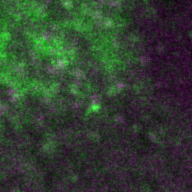

Supplement: Supplementary file 5 — Source Data for Figure 3 [file EMBR-24-e56841-s004.zip › Figure_3/3A/zoom/2_45_merge.tif]

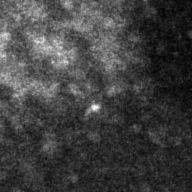

Supplement: Supplementary file 5 — Source Data for Figure 3 [file EMBR-24-e56841-s004.zip › Figure_3/3A/zoom/2_45_TECPR1.tif]

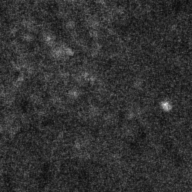

Supplement: Supplementary file 5 — Source Data for Figure 3 [file EMBR-24-e56841-s004.zip › Figure_3/3A/zoom/5_15_Gal3.tif]

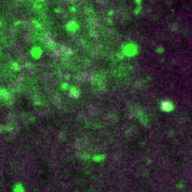

Supplement: Supplementary file 5 — Source Data for Figure 3 [file EMBR-24-e56841-s004.zip › Figure_3/3A/zoom/5_15_merge.tif]

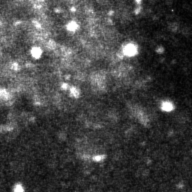

Supplement: Supplementary file 5 — Source Data for Figure 3 [file EMBR-24-e56841-s004.zip › Figure_3/3A/zoom/5_15_TECPR1.tif]

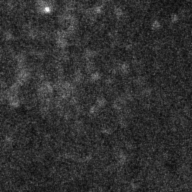

Supplement: Supplementary file 5 — Source Data for Figure 3 [file EMBR-24-e56841-s004.zip › Figure_3/3A/zoom/7_30_Gal3.tif]

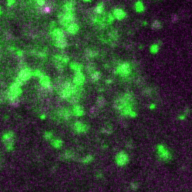

Supplement: Supplementary file 5 — Source Data for Figure 3 [file EMBR-24-e56841-s004.zip › Figure_3/3A/zoom/7_30_Merge.tif]

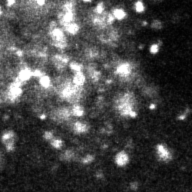

Supplement: Supplementary file 5 — Source Data for Figure 3 [file EMBR-24-e56841-s004.zip › Figure_3/3A/zoom/7_30_TECPR1.tif]

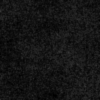

Supplement: Supplementary file 5 — Source Data for Figure 3 [file EMBR-24-e56841-s004.zip › Figure_3/3B/0min_Gal3.tif]

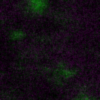

Supplement: Supplementary file 5 — Source Data for Figure 3 [file EMBR-24-e56841-s004.zip › Figure_3/3B/0min_merge.tif]

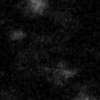

Supplement: Supplementary file 5 — Source Data for Figure 3 [file EMBR-24-e56841-s004.zip › Figure_3/3B/0min_TECPR1.tif]

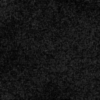

Supplement: Supplementary file 5 — Source Data for Figure 3 [file EMBR-24-e56841-s004.zip › Figure_3/3B/5min15sec_Gal3.tif]

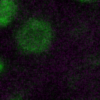

Supplement: Supplementary file 5 — Source Data for Figure 3 [file EMBR-24-e56841-s004.zip › Figure_3/3B/5min15sec_merge.tif]

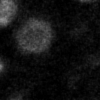

Supplement: Supplementary file 5 — Source Data for Figure 3 [file EMBR-24-e56841-s004.zip › Figure_3/3B/5min15sec_TECPR1.tif]

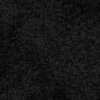

Supplement: Supplementary file 5 — Source Data for Figure 3 [file EMBR-24-e56841-s004.zip › Figure_3/3B/5min30sec_Gal3.tif]

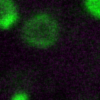

Supplement: Supplementary file 5 — Source Data for Figure 3 [file EMBR-24-e56841-s004.zip › Figure_3/3B/5min30sec_merge.tif]

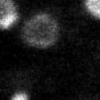

Supplement: Supplementary file 5 — Source Data for Figure 3 [file EMBR-24-e56841-s004.zip › Figure_3/3B/5min30sec_TECPR1.tif]

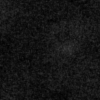

Supplement: Supplementary file 5 — Source Data for Figure 3 [file EMBR-24-e56841-s004.zip › Figure_3/3B/5min45sec_Gal3.tif]

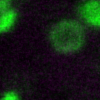

Supplement: Supplementary file 5 — Source Data for Figure 3 [file EMBR-24-e56841-s004.zip › Figure_3/3B/5min45sec_merge.tif]

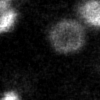

Supplement: Supplementary file 5 — Source Data for Figure 3 [file EMBR-24-e56841-s004.zip › Figure_3/3B/5min45sec_TECPR1.tif]

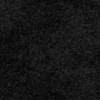

Supplement: Supplementary file 5 — Source Data for Figure 3 [file EMBR-24-e56841-s004.zip › Figure_3/3B/5min_Gal3.tif]

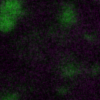

Supplement: Supplementary file 5 — Source Data for Figure 3 [file EMBR-24-e56841-s004.zip › Figure_3/3B/5min_merge.tif]

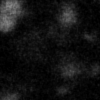

Supplement: Supplementary file 5 — Source Data for Figure 3 [file EMBR-24-e56841-s004.zip › Figure_3/3B/5min_TECPR1.tif]

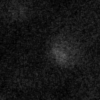

Supplement: Supplementary file 5 — Source Data for Figure 3 [file EMBR-24-e56841-s004.zip › Figure_3/3B/6min15sec_Gal3.tif]

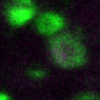

Supplement: Supplementary file 5 — Source Data for Figure 3 [file EMBR-24-e56841-s004.zip › Figure_3/3B/6min15sec_merge.tif]

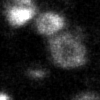

Supplement: Supplementary file 5 — Source Data for Figure 3 [file EMBR-24-e56841-s004.zip › Figure_3/3B/6min15sec_TECPR1.tif]

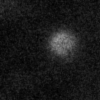

Supplement: Supplementary file 5 — Source Data for Figure 3 [file EMBR-24-e56841-s004.zip › Figure_3/3B/6min30sec_Gal3.tif]

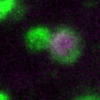

Supplement: Supplementary file 5 — Source Data for Figure 3 [file EMBR-24-e56841-s004.zip › Figure_3/3B/6min30sec_merge.tif]

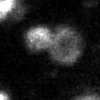

Supplement: Supplementary file 5 — Source Data for Figure 3 [file EMBR-24-e56841-s004.zip › Figure_3/3B/6min30sec_TECPR1.tif]

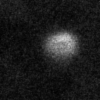

Supplement: Supplementary file 5 — Source Data for Figure 3 [file EMBR-24-e56841-s004.zip › Figure_3/3B/6min45sec_Gal3.tif]

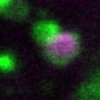

Supplement: Supplementary file 5 — Source Data for Figure 3 [file EMBR-24-e56841-s004.zip › Figure_3/3B/6min45sec_merge.tif]

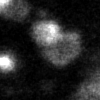

Supplement: Supplementary file 5 — Source Data for Figure 3 [file EMBR-24-e56841-s004.zip › Figure_3/3B/6min45sec_TECPR1.tif]

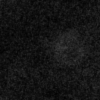

Supplement: Supplementary file 5 — Source Data for Figure 3 [file EMBR-24-e56841-s004.zip › Figure_3/3B/6min_Gal3.tif]

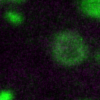

Supplement: Supplementary file 5 — Source Data for Figure 3 [file EMBR-24-e56841-s004.zip › Figure_3/3B/6min_merge.tif]

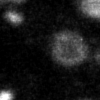

Supplement: Supplementary file 5 — Source Data for Figure 3 [file EMBR-24-e56841-s004.zip › Figure_3/3B/6min_TECPR1.tif]

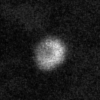

Supplement: Supplementary file 5 — Source Data for Figure 3 [file EMBR-24-e56841-s004.zip › Figure_3/3B/7min15sec_Gal3.tif]

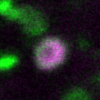

Supplement: Supplementary file 5 — Source Data for Figure 3 [file EMBR-24-e56841-s004.zip › Figure_3/3B/7min15sec_merge.tif]

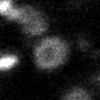

Supplement: Supplementary file 5 — Source Data for Figure 3 [file EMBR-24-e56841-s004.zip › Figure_3/3B/7min15sec_TECPR1.tif]

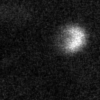

Supplement: Supplementary file 5 — Source Data for Figure 3 [file EMBR-24-e56841-s004.zip › Figure_3/3B/7min_Gal3.tif]

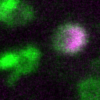

Supplement: Supplementary file 5 — Source Data for Figure 3 [file EMBR-24-e56841-s004.zip › Figure_3/3B/7min_merge.tif]

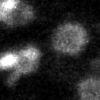

Supplement: Supplementary file 5 — Source Data for Figure 3 [file EMBR-24-e56841-s004.zip › Figure_3/3B/7min_TECPR1.tif]
