# Supplementary figures and images for: An ATG12‐ATG5‐TECPR1 E3‐like complex regulates unconventional LC3 lipidation at damaged lysosomes (part 3 of 4)
Source: EMBO Rep. 2023 Jun 29;24(9):e56841. doi: 10.15252/embr.202356841 (PMC10481663; doi:10.15252/embr.202356841)

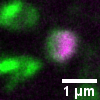

Supplement: Supplementary file 5 — Source Data for Figure 3 [file EMBR-24-e56841-s004.zip › Figure_3/3B/scale.tif]

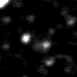

Supplement: Supplementary file 5 — Source Data for Figure 3 [file EMBR-24-e56841-s004.zip › Figure_3/3D/Image_Data/CHMP2A_zoom_1.tif]

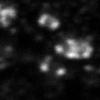

Supplement: Supplementary file 5 — Source Data for Figure 3 [file EMBR-24-e56841-s004.zip › Figure_3/3D/Image_Data/CHMP2A_zoom_2.tif]

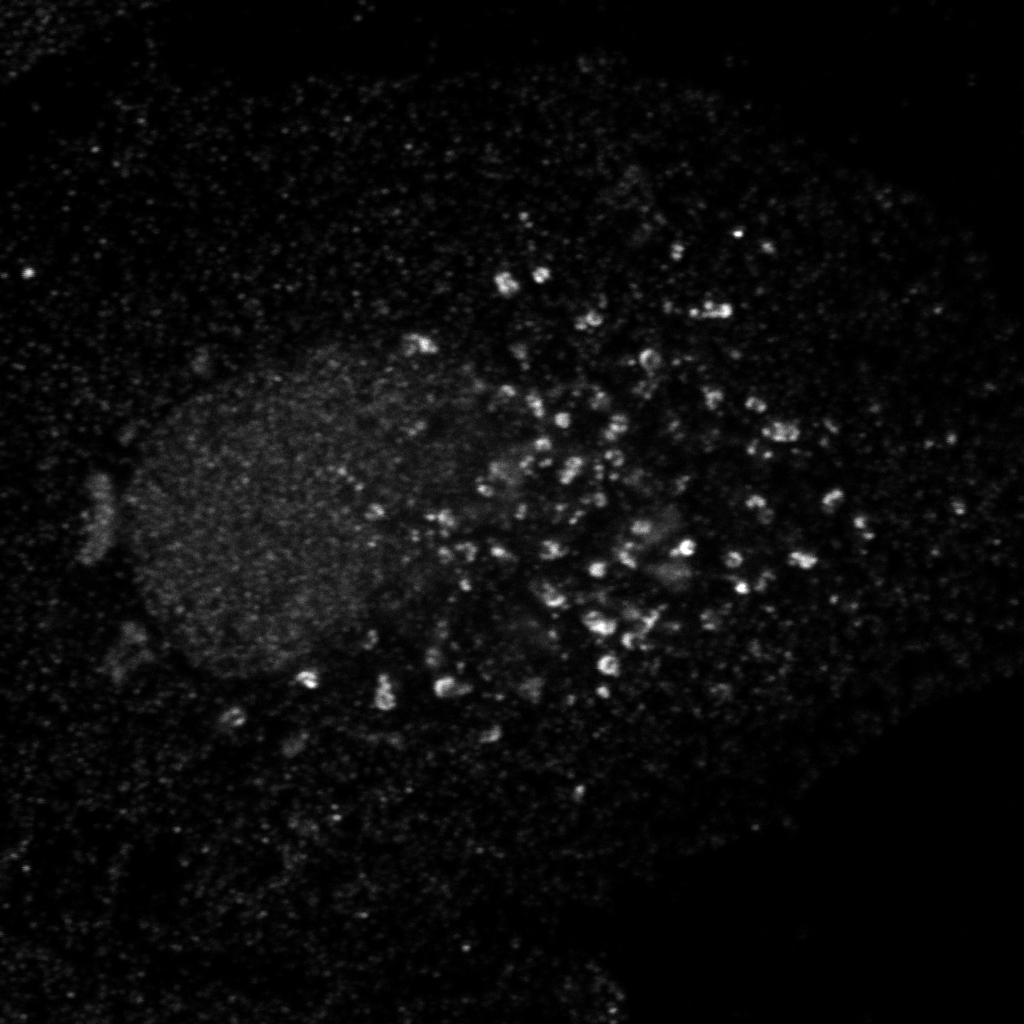

Supplement: Supplementary file 5 — Source Data for Figure 3 [file EMBR-24-e56841-s004.zip › Figure_3/3D/Image_Data/CHMP4A.tif]

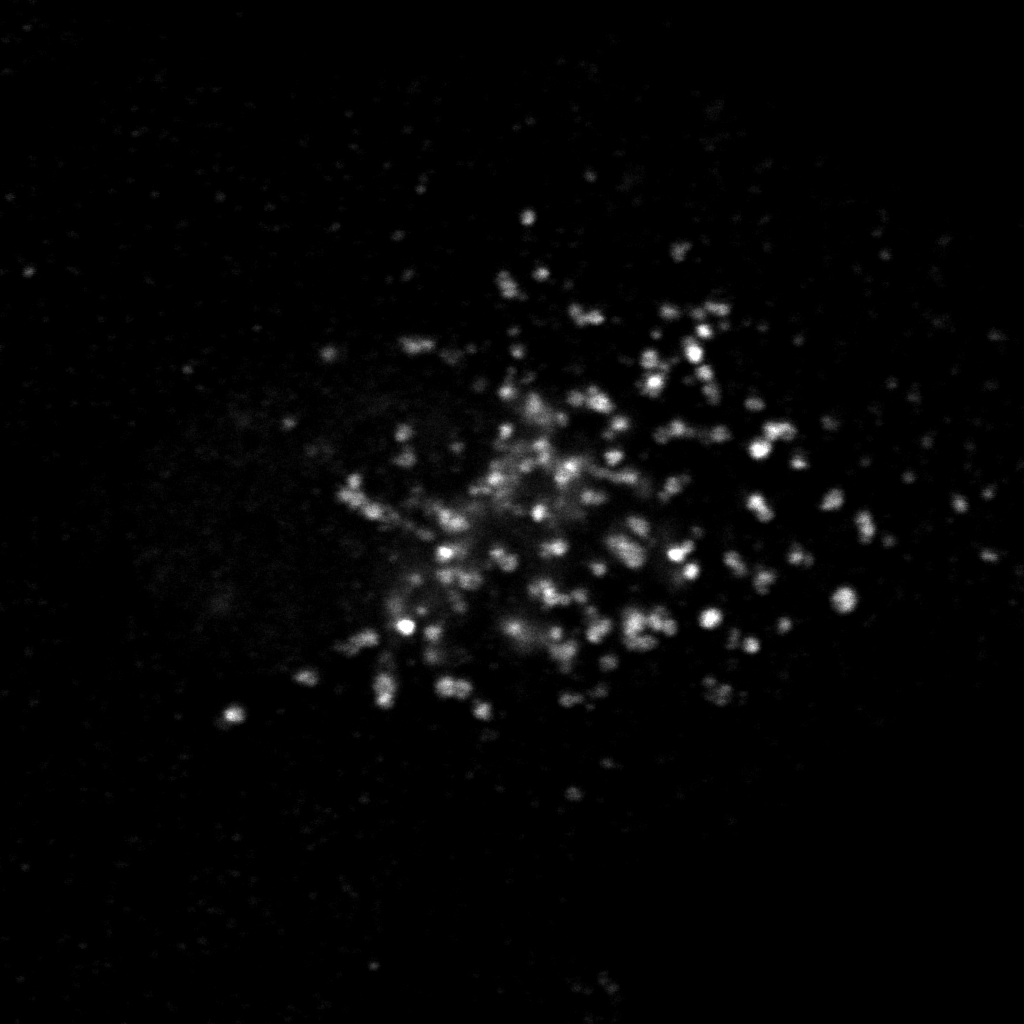

Supplement: Supplementary file 5 — Source Data for Figure 3 [file EMBR-24-e56841-s004.zip › Figure_3/3D/Image_Data/LAMP.tif]

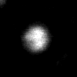

Supplement: Supplementary file 5 — Source Data for Figure 3 [file EMBR-24-e56841-s004.zip › Figure_3/3D/Image_Data/LAMP_zoom_1.tif]

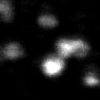

Supplement: Supplementary file 5 — Source Data for Figure 3 [file EMBR-24-e56841-s004.zip › Figure_3/3D/Image_Data/LAMP_zoom_2.tif]

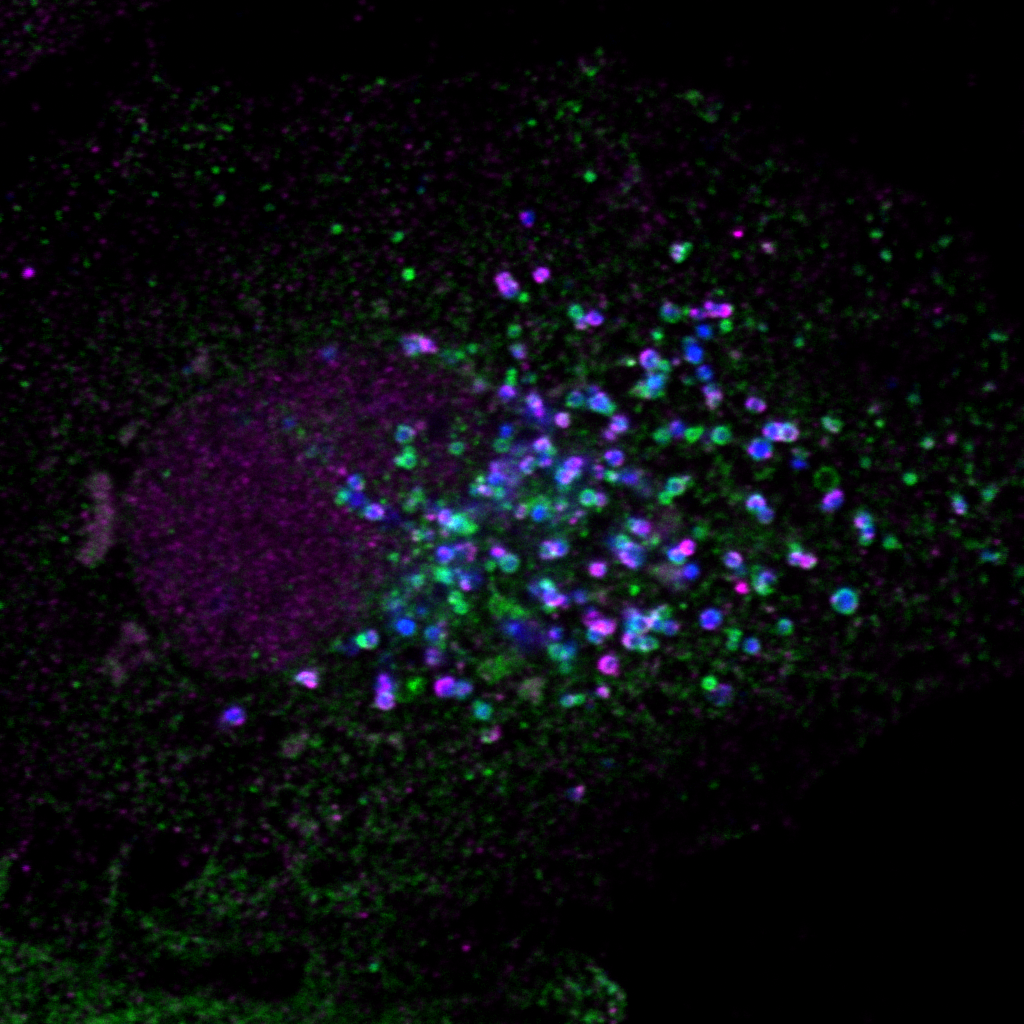

Supplement: Supplementary file 5 — Source Data for Figure 3 [file EMBR-24-e56841-s004.zip › Figure_3/3D/Image_Data/Merge.tif]

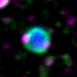

Supplement: Supplementary file 5 — Source Data for Figure 3 [file EMBR-24-e56841-s004.zip › Figure_3/3D/Image_Data/Merge_zoom_1.tif]

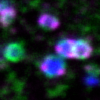

Supplement: Supplementary file 5 — Source Data for Figure 3 [file EMBR-24-e56841-s004.zip › Figure_3/3D/Image_Data/Merge_zoom_2.tif]

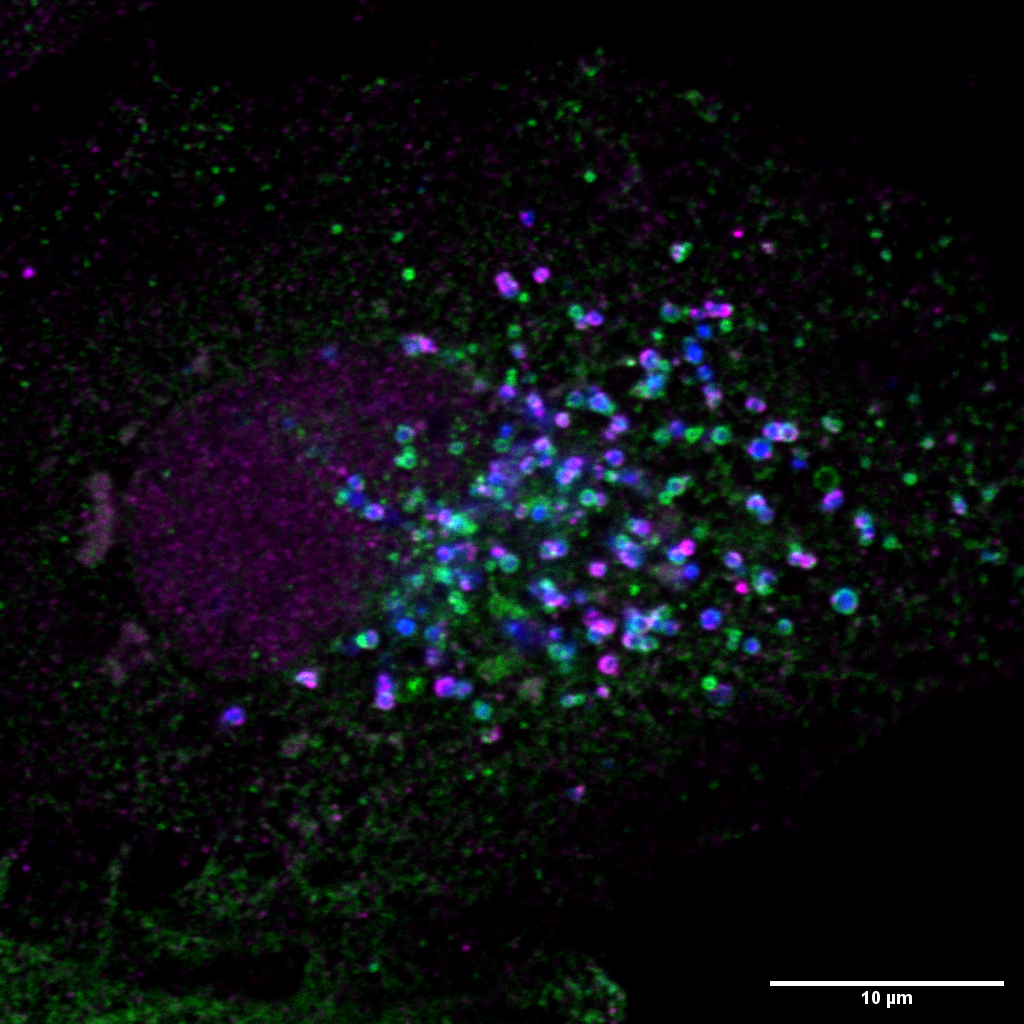

Supplement: Supplementary file 5 — Source Data for Figure 3 [file EMBR-24-e56841-s004.zip › Figure_3/3D/Image_Data/Scale.tif]

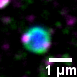

Supplement: Supplementary file 5 — Source Data for Figure 3 [file EMBR-24-e56841-s004.zip › Figure_3/3D/Image_Data/Scale_zoom_1.tif]

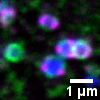

Supplement: Supplementary file 5 — Source Data for Figure 3 [file EMBR-24-e56841-s004.zip › Figure_3/3D/Image_Data/Scale_zoom_2.tif]

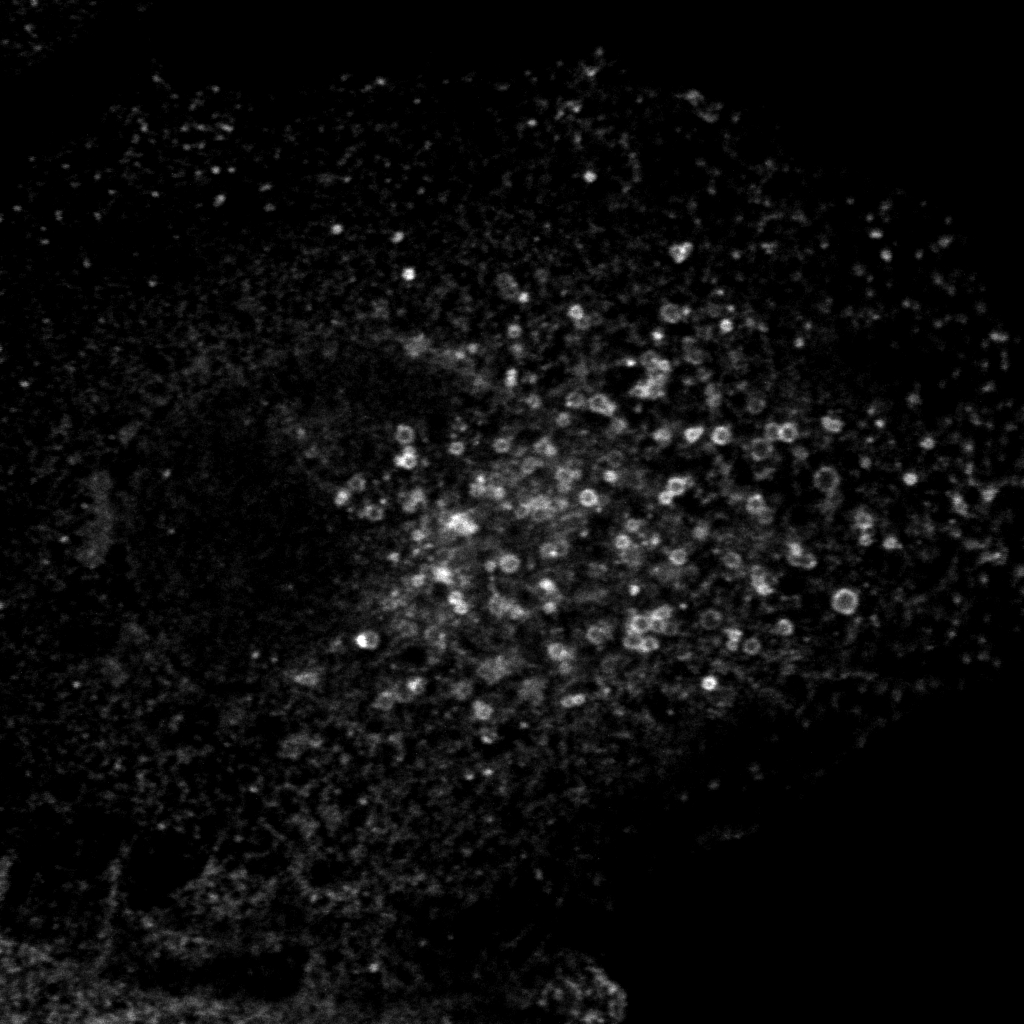

Supplement: Supplementary file 5 — Source Data for Figure 3 [file EMBR-24-e56841-s004.zip › Figure_3/3D/Image_Data/TECPR1.tif]

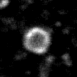

Supplement: Supplementary file 5 — Source Data for Figure 3 [file EMBR-24-e56841-s004.zip › Figure_3/3D/Image_Data/TECPR1_zoom_1.tif]

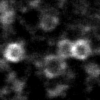

Supplement: Supplementary file 5 — Source Data for Figure 3 [file EMBR-24-e56841-s004.zip › Figure_3/3D/Image_Data/TECPR1_zoom_2.tif]

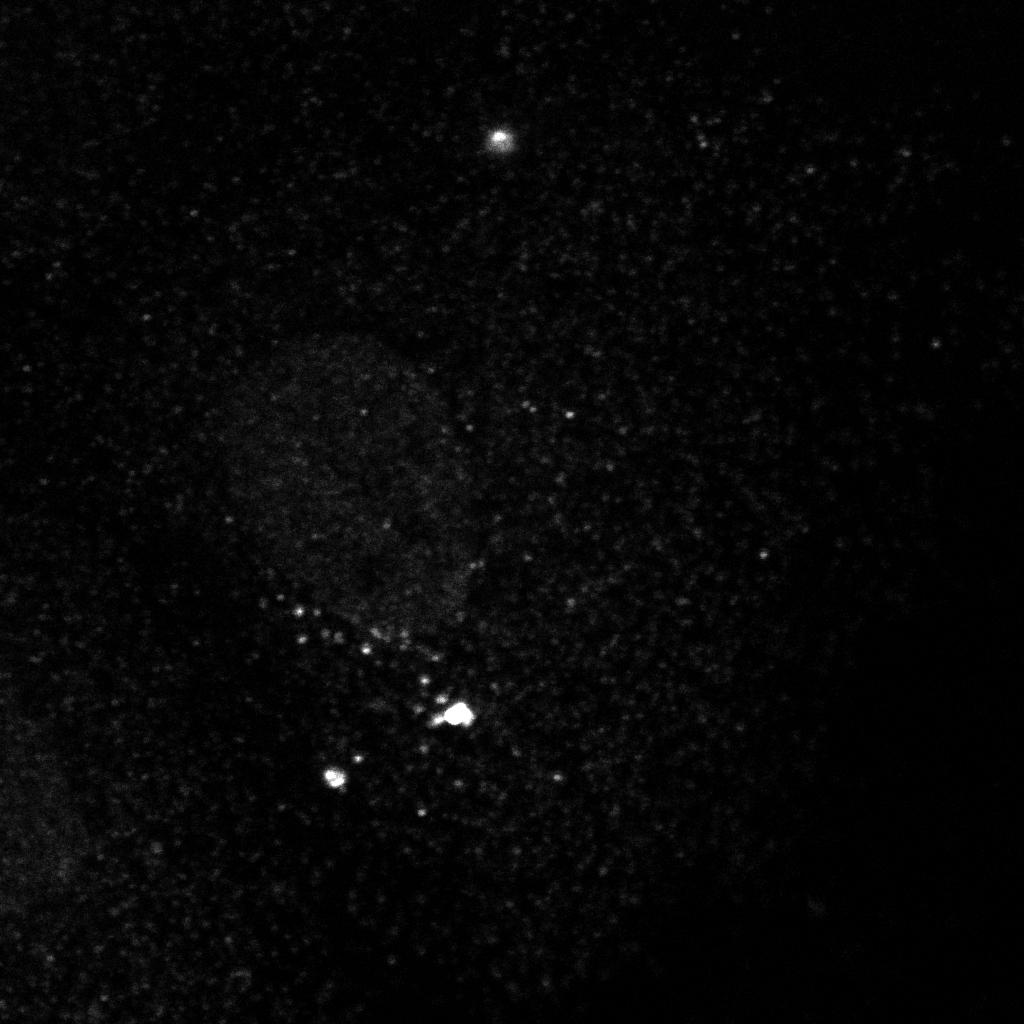

Supplement: Supplementary file 5 — Source Data for Figure 3 [file EMBR-24-e56841-s004.zip › Figure_3/3E/0min_ALIX_magenta.tif]

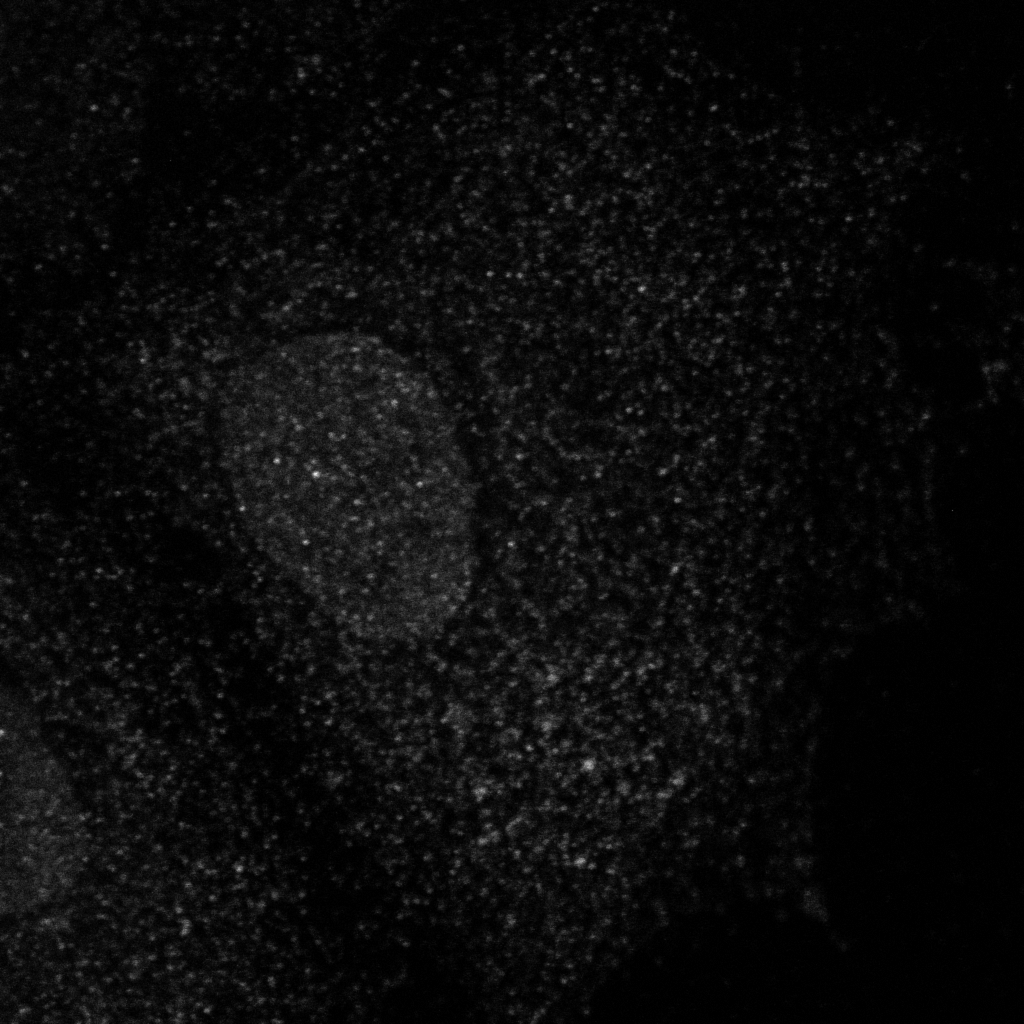

Supplement: Supplementary file 5 — Source Data for Figure 3 [file EMBR-24-e56841-s004.zip › Figure_3/3E/0min_Gal3_cyan.tif]

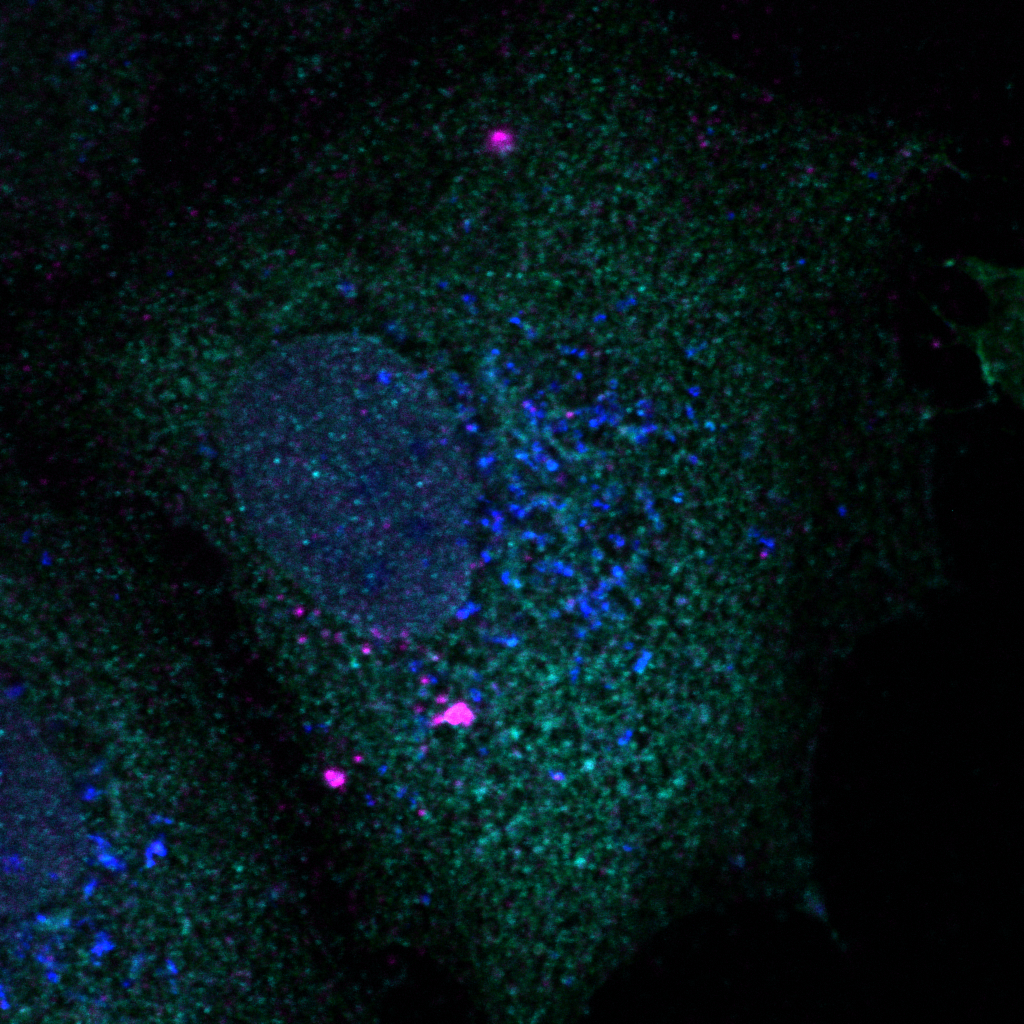

Supplement: Supplementary file 5 — Source Data for Figure 3 [file EMBR-24-e56841-s004.zip › Figure_3/3E/0min_merge.tif]

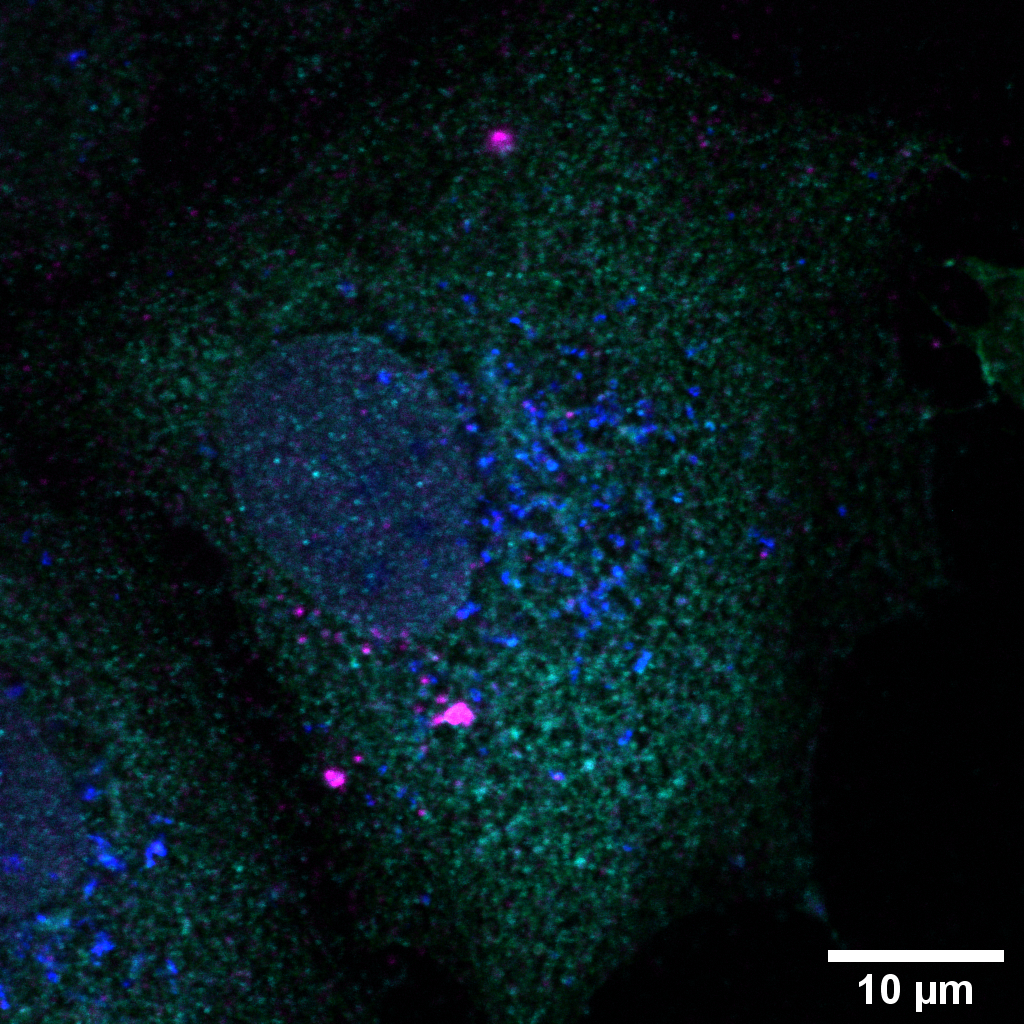

Supplement: Supplementary file 5 — Source Data for Figure 3 [file EMBR-24-e56841-s004.zip › Figure_3/3E/0min_scale.tif]

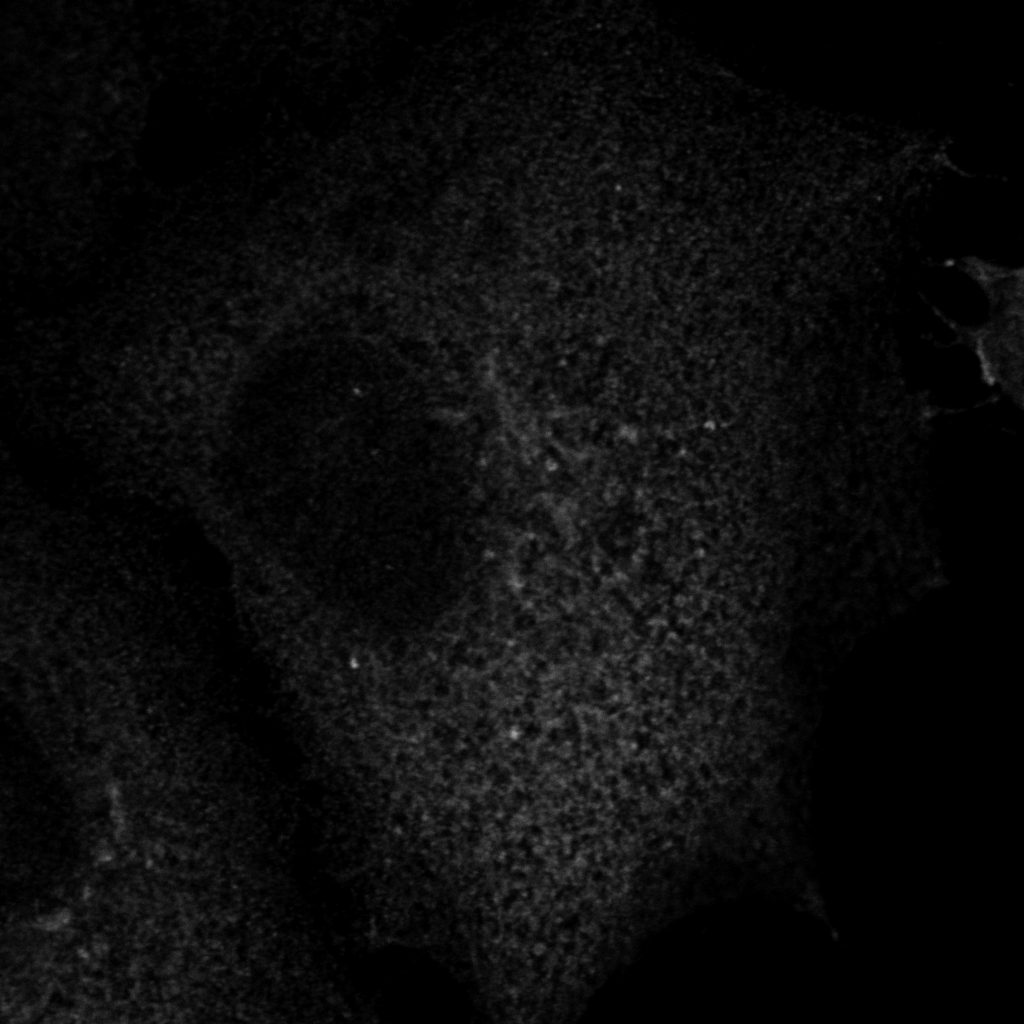

Supplement: Supplementary file 5 — Source Data for Figure 3 [file EMBR-24-e56841-s004.zip › Figure_3/3E/0min_TECPR1_green.tif]

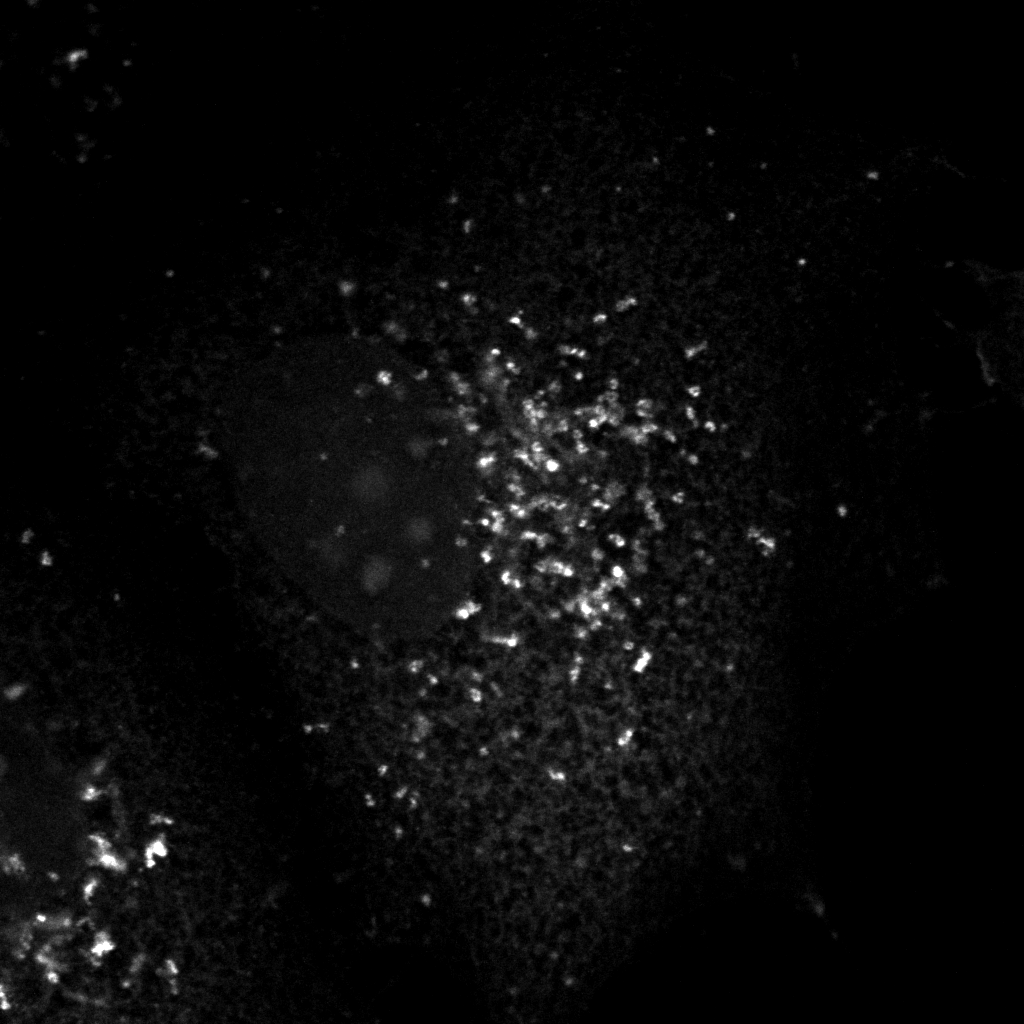

Supplement: Supplementary file 5 — Source Data for Figure 3 [file EMBR-24-e56841-s004.zip › Figure_3/3E/0min_TMEM_blue.tif]

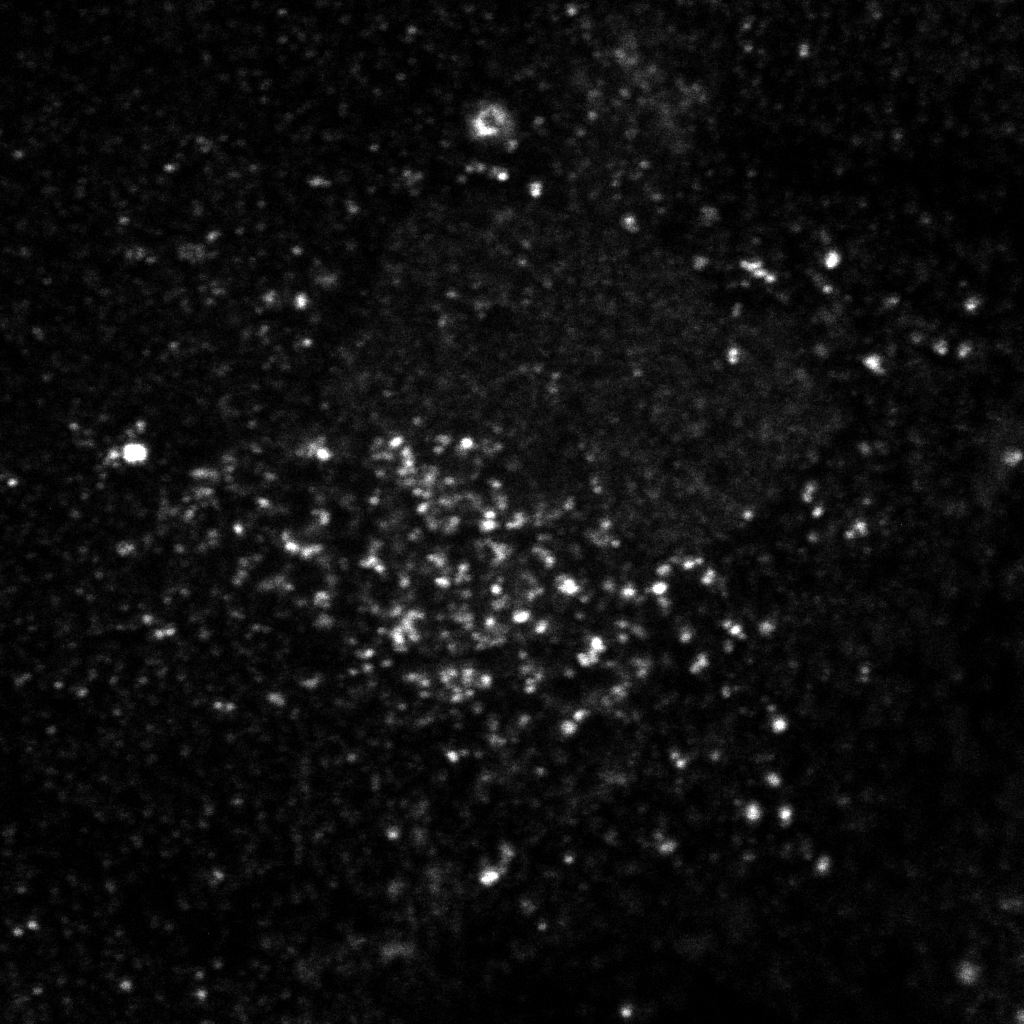

Supplement: Supplementary file 5 — Source Data for Figure 3 [file EMBR-24-e56841-s004.zip › Figure_3/3E/10min_ALIX_magenta.tif]

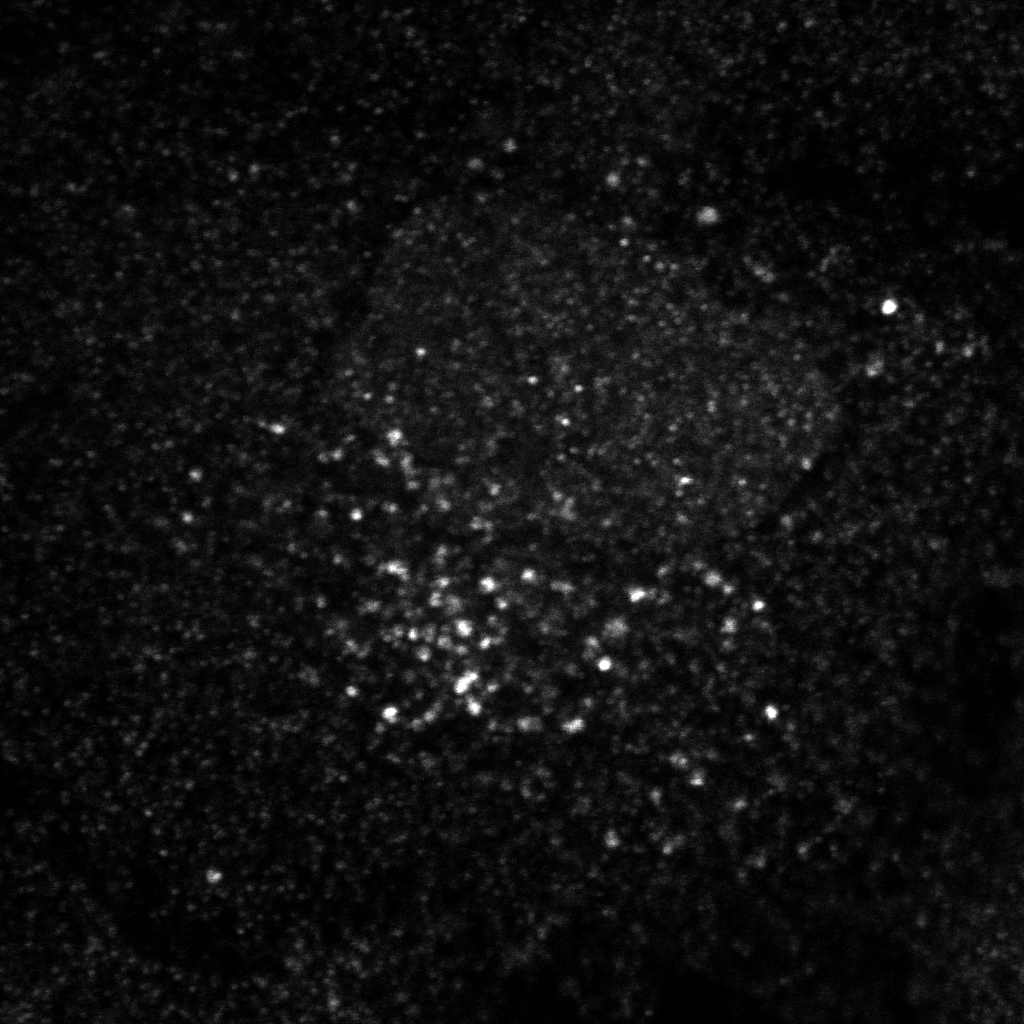

Supplement: Supplementary file 5 — Source Data for Figure 3 [file EMBR-24-e56841-s004.zip › Figure_3/3E/10min_Gal3_cyan.tif]

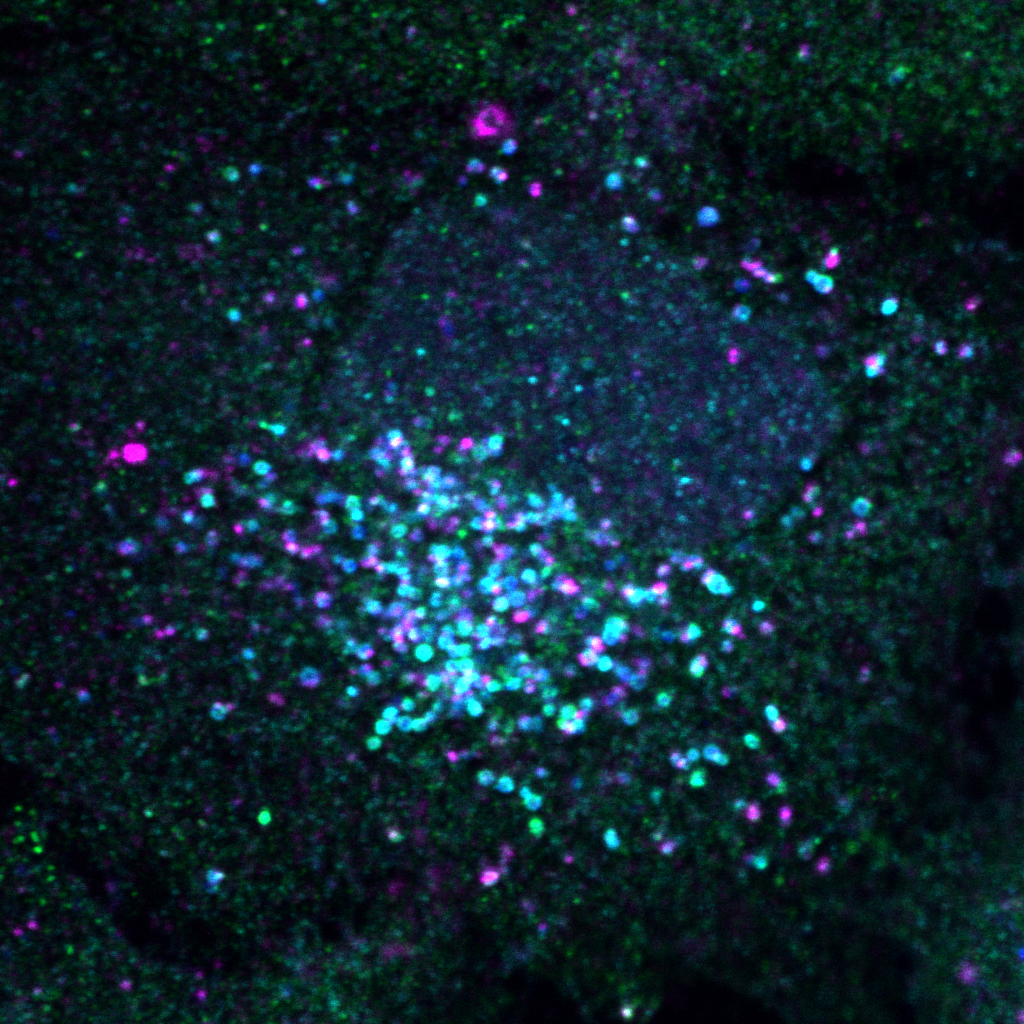

Supplement: Supplementary file 5 — Source Data for Figure 3 [file EMBR-24-e56841-s004.zip › Figure_3/3E/10min_merge.tif]

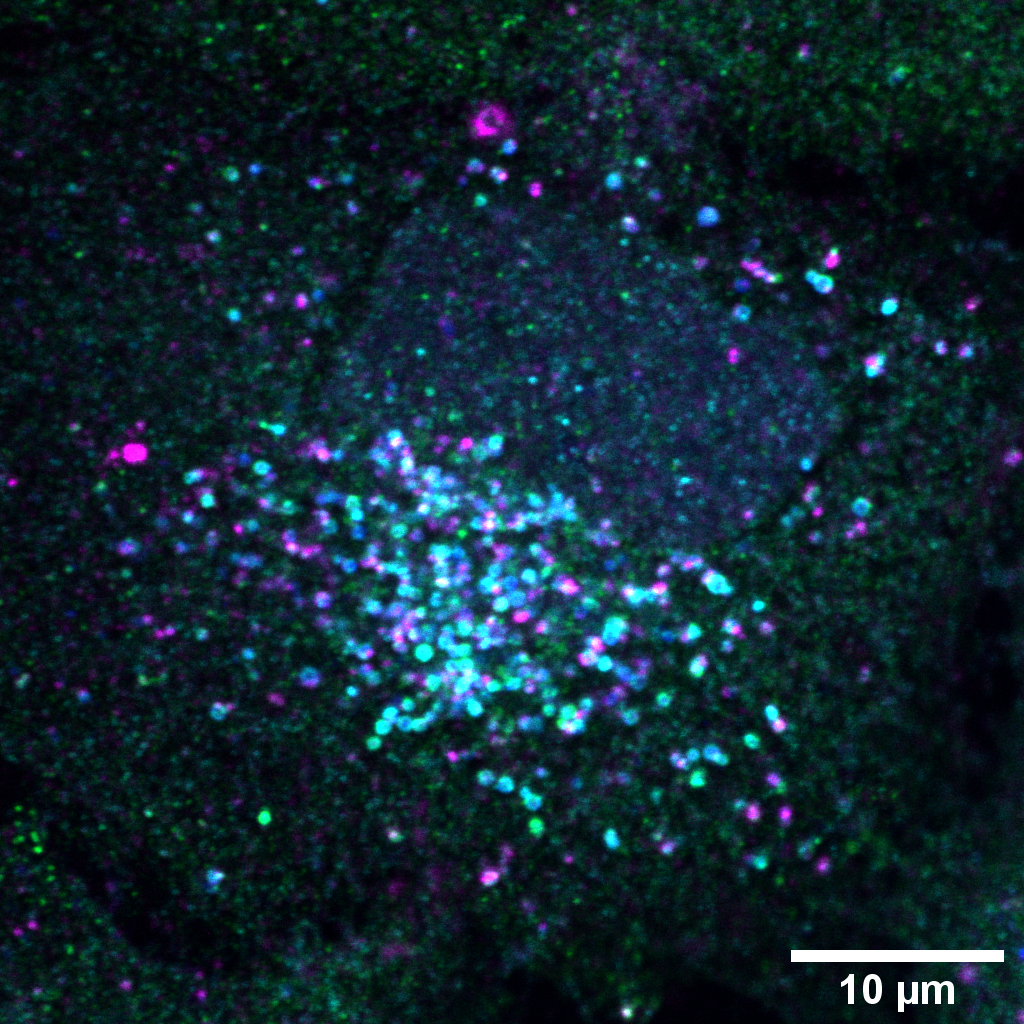

Supplement: Supplementary file 5 — Source Data for Figure 3 [file EMBR-24-e56841-s004.zip › Figure_3/3E/10min_scale.tif]

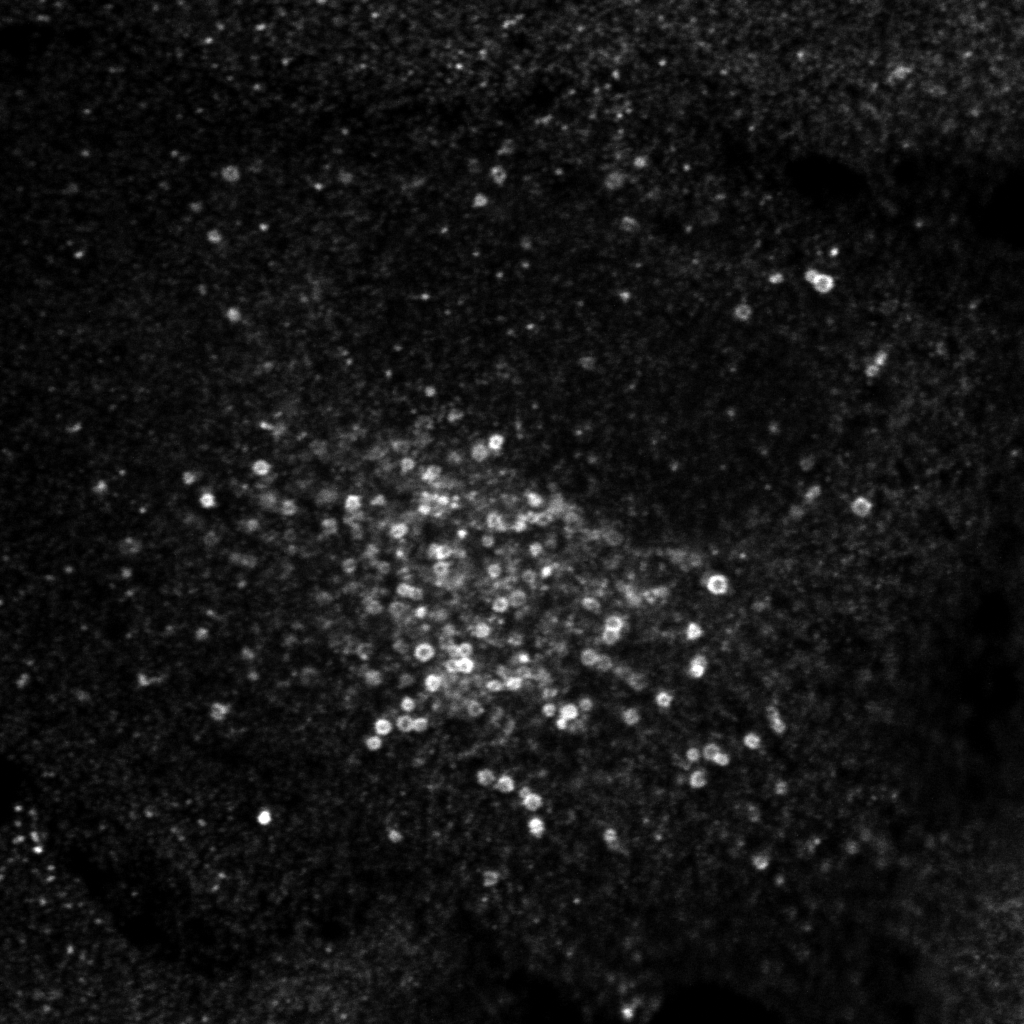

Supplement: Supplementary file 5 — Source Data for Figure 3 [file EMBR-24-e56841-s004.zip › Figure_3/3E/10min_TECPR1_green.tif]

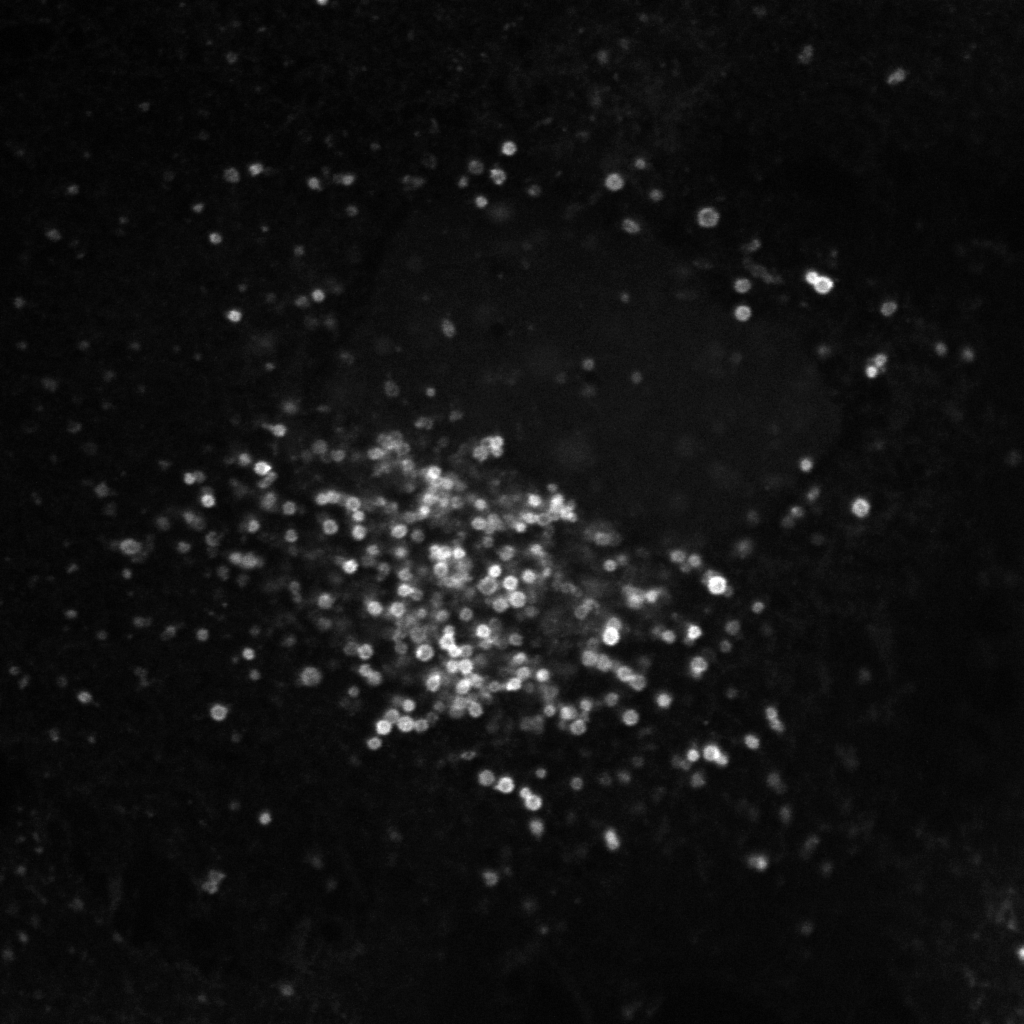

Supplement: Supplementary file 5 — Source Data for Figure 3 [file EMBR-24-e56841-s004.zip › Figure_3/3E/10min_TMEM_blue.tif]

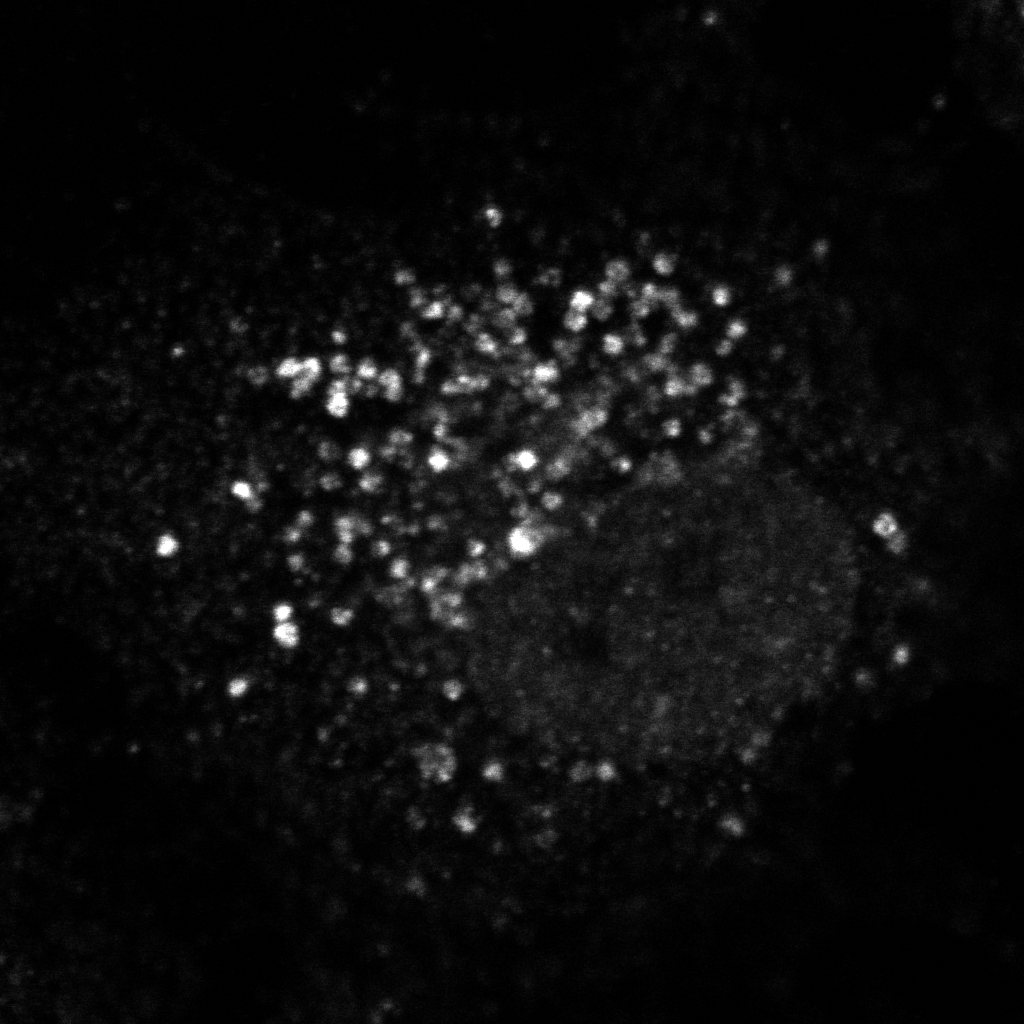

Supplement: Supplementary file 5 — Source Data for Figure 3 [file EMBR-24-e56841-s004.zip › Figure_3/3E/20min_ALIX_mag.tif]

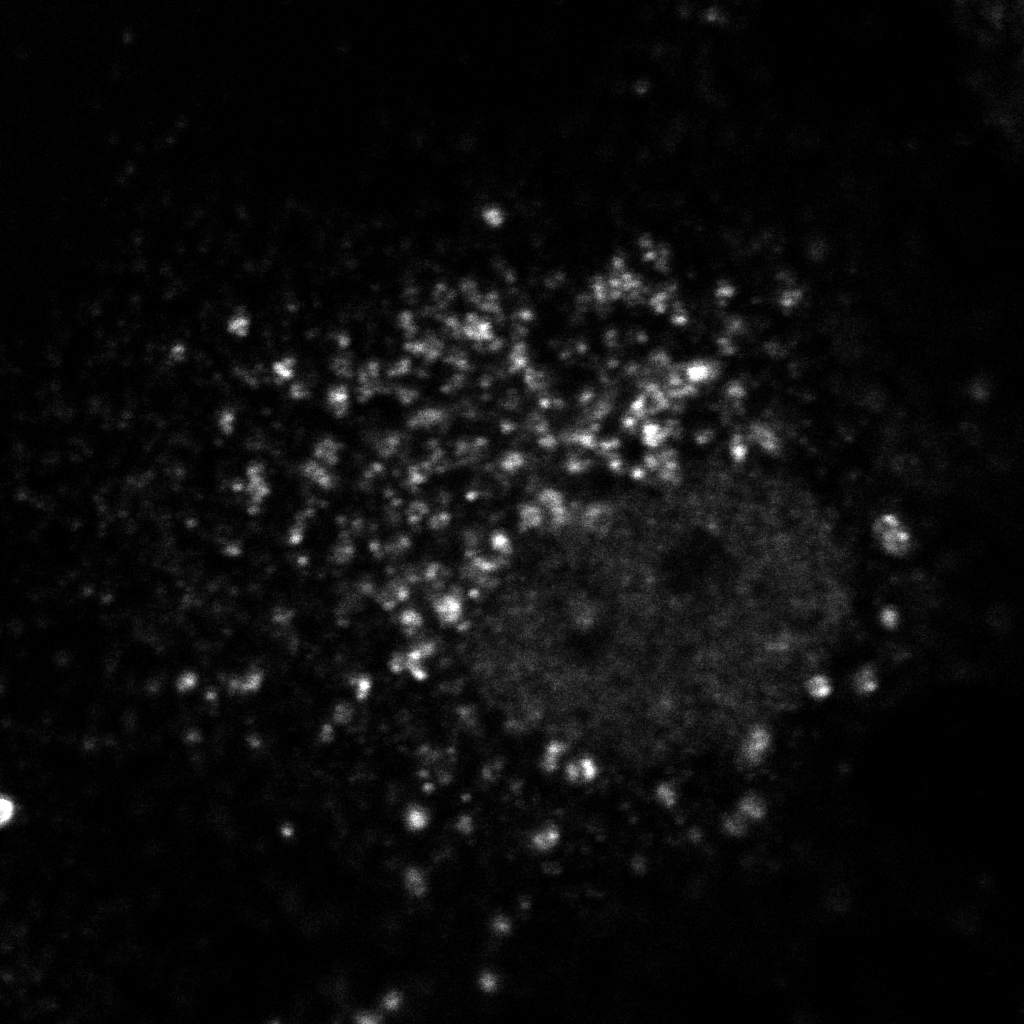

Supplement: Supplementary file 5 — Source Data for Figure 3 [file EMBR-24-e56841-s004.zip › Figure_3/3E/20min_Gal3_cyan.tif]

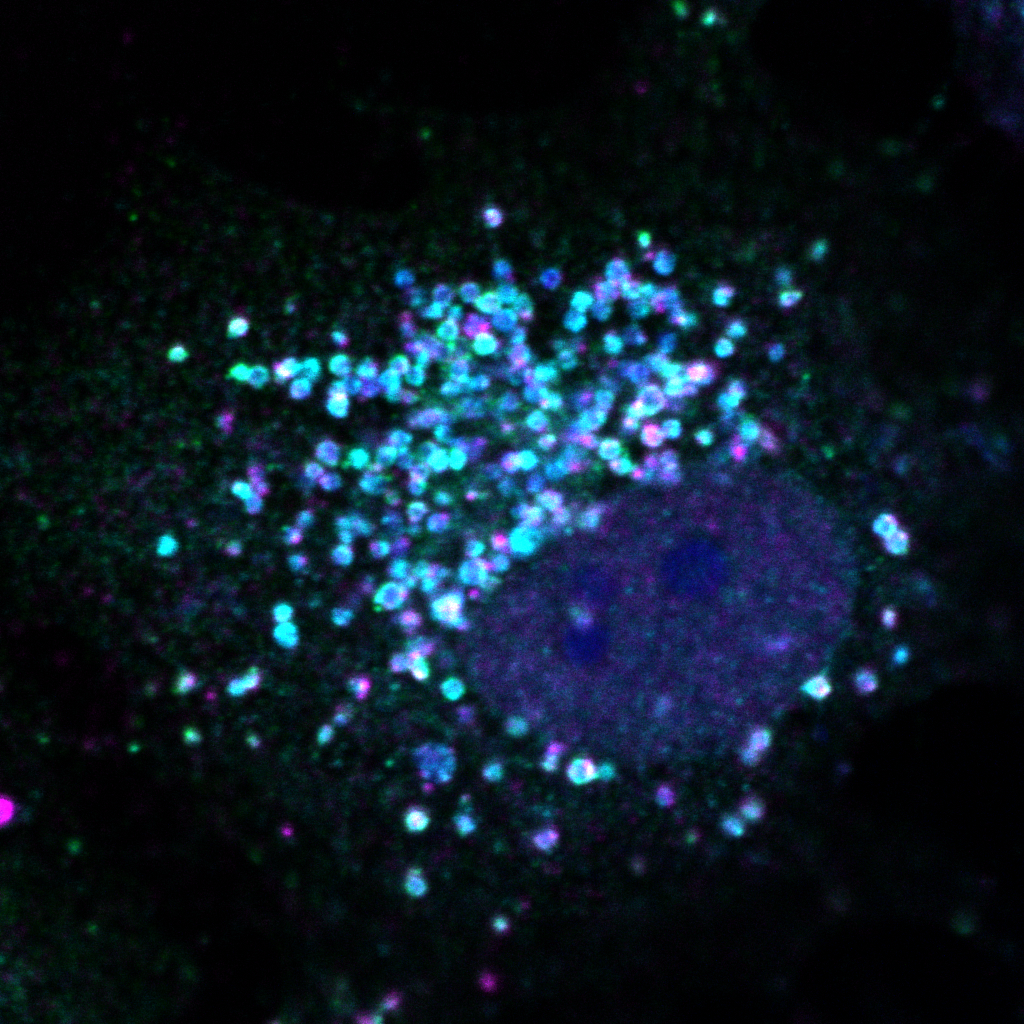

Supplement: Supplementary file 5 — Source Data for Figure 3 [file EMBR-24-e56841-s004.zip › Figure_3/3E/20min_merge.tif]

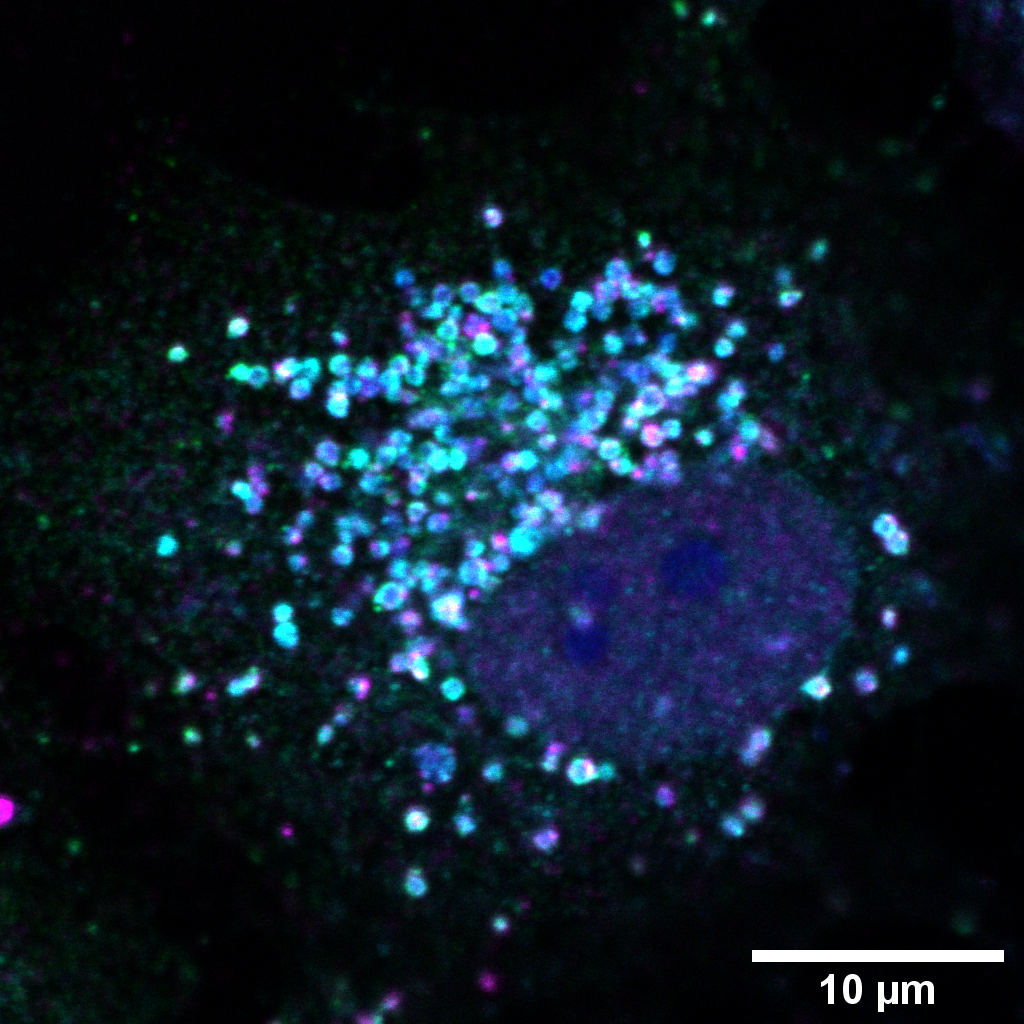

Supplement: Supplementary file 5 — Source Data for Figure 3 [file EMBR-24-e56841-s004.zip › Figure_3/3E/20min_scale.tif]

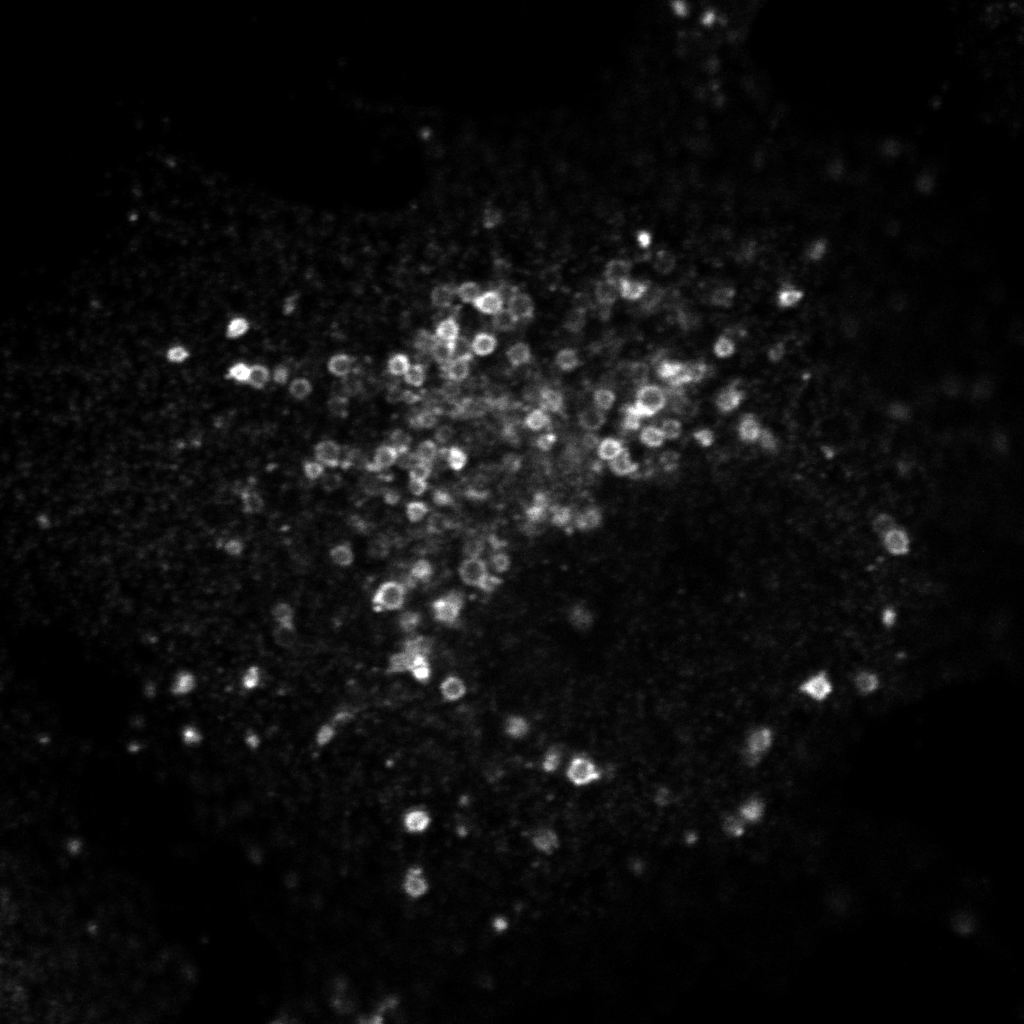

Supplement: Supplementary file 5 — Source Data for Figure 3 [file EMBR-24-e56841-s004.zip › Figure_3/3E/20min_TECPR1_green.tif]

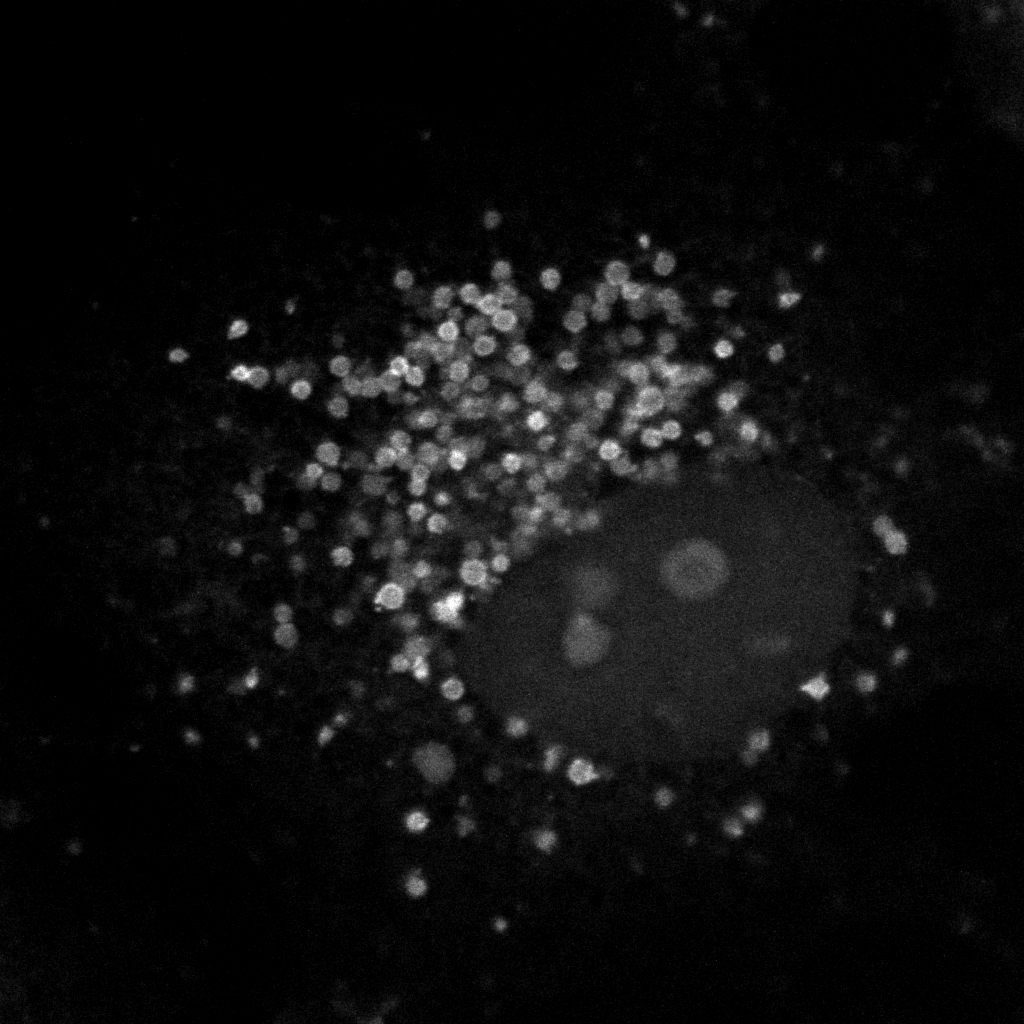

Supplement: Supplementary file 5 — Source Data for Figure 3 [file EMBR-24-e56841-s004.zip › Figure_3/3E/20min_TMEM_blue.tif]

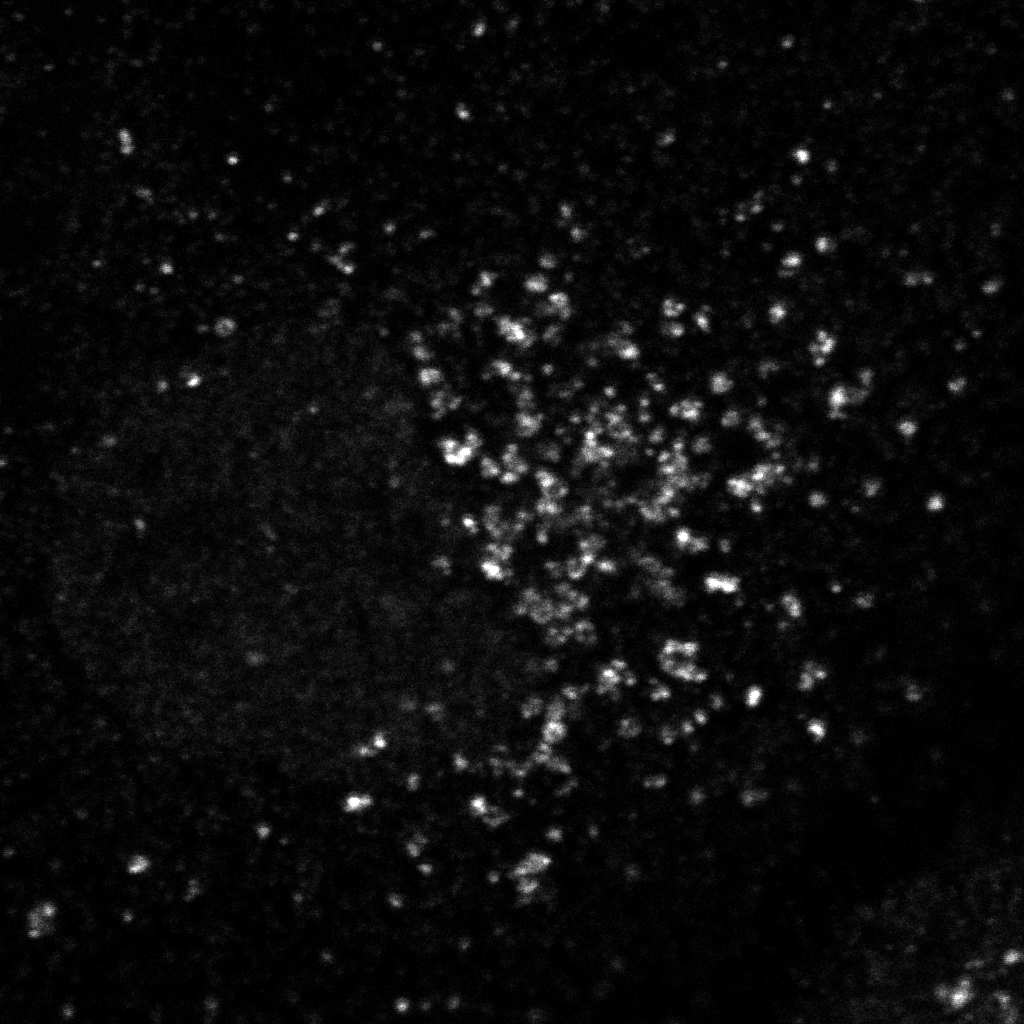

Supplement: Supplementary file 5 — Source Data for Figure 3 [file EMBR-24-e56841-s004.zip › Figure_3/3E/30min_ALIX_magenta.tif]

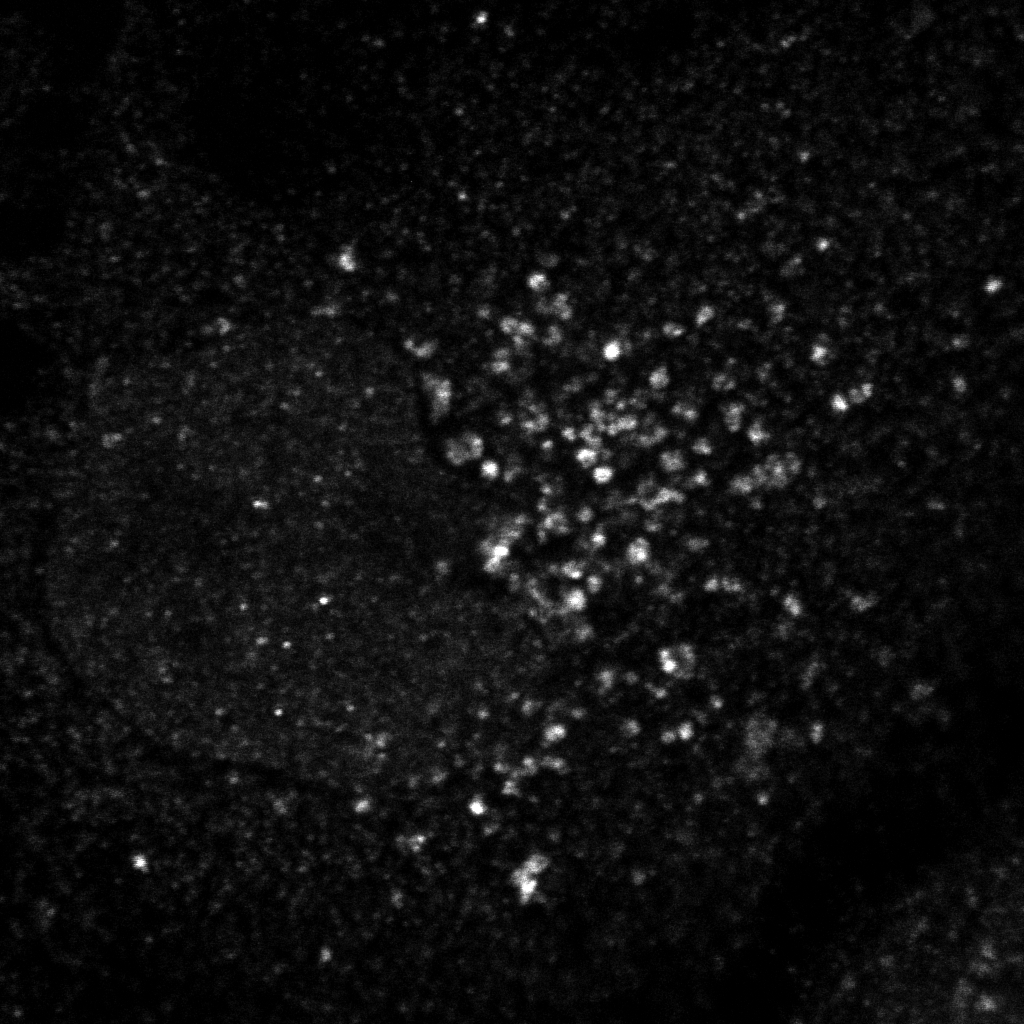

Supplement: Supplementary file 5 — Source Data for Figure 3 [file EMBR-24-e56841-s004.zip › Figure_3/3E/30min_Gal3_cyan.tif]

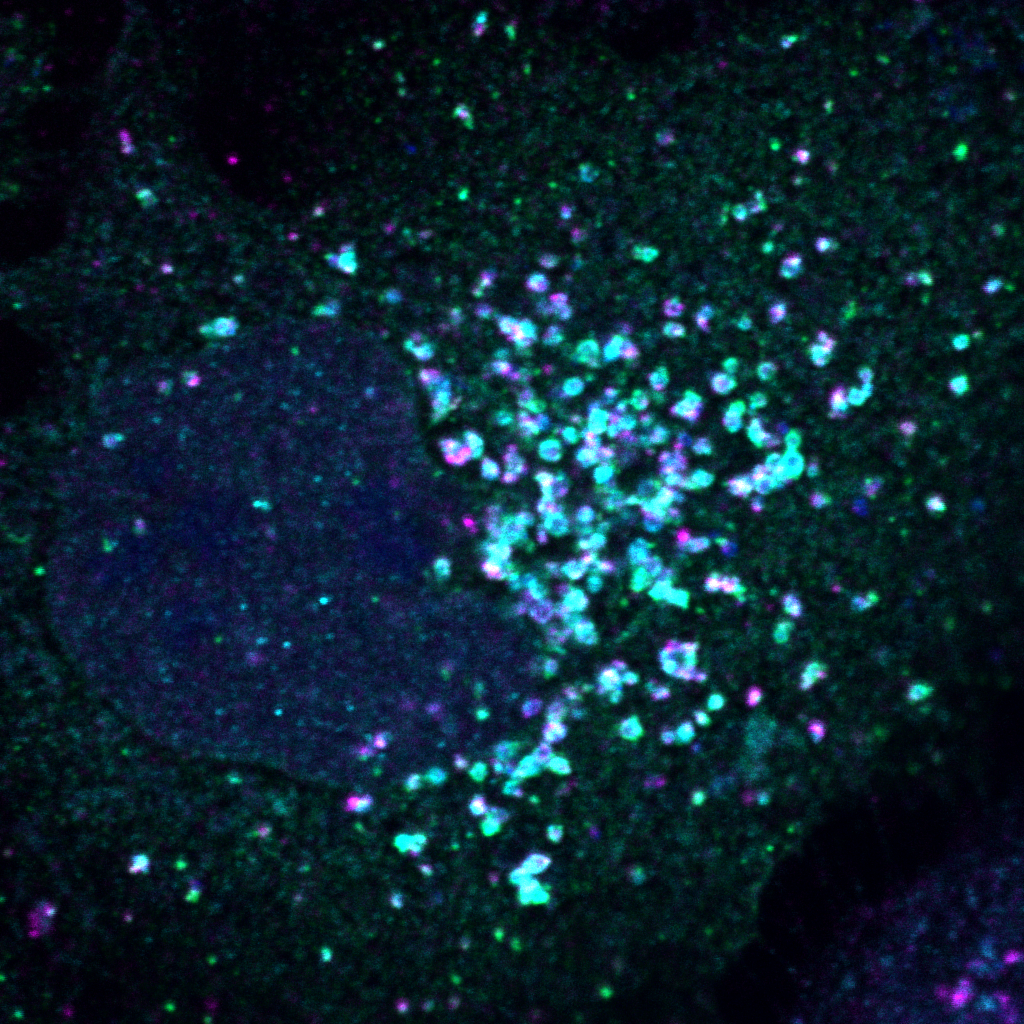

Supplement: Supplementary file 5 — Source Data for Figure 3 [file EMBR-24-e56841-s004.zip › Figure_3/3E/30min_merge.tif]

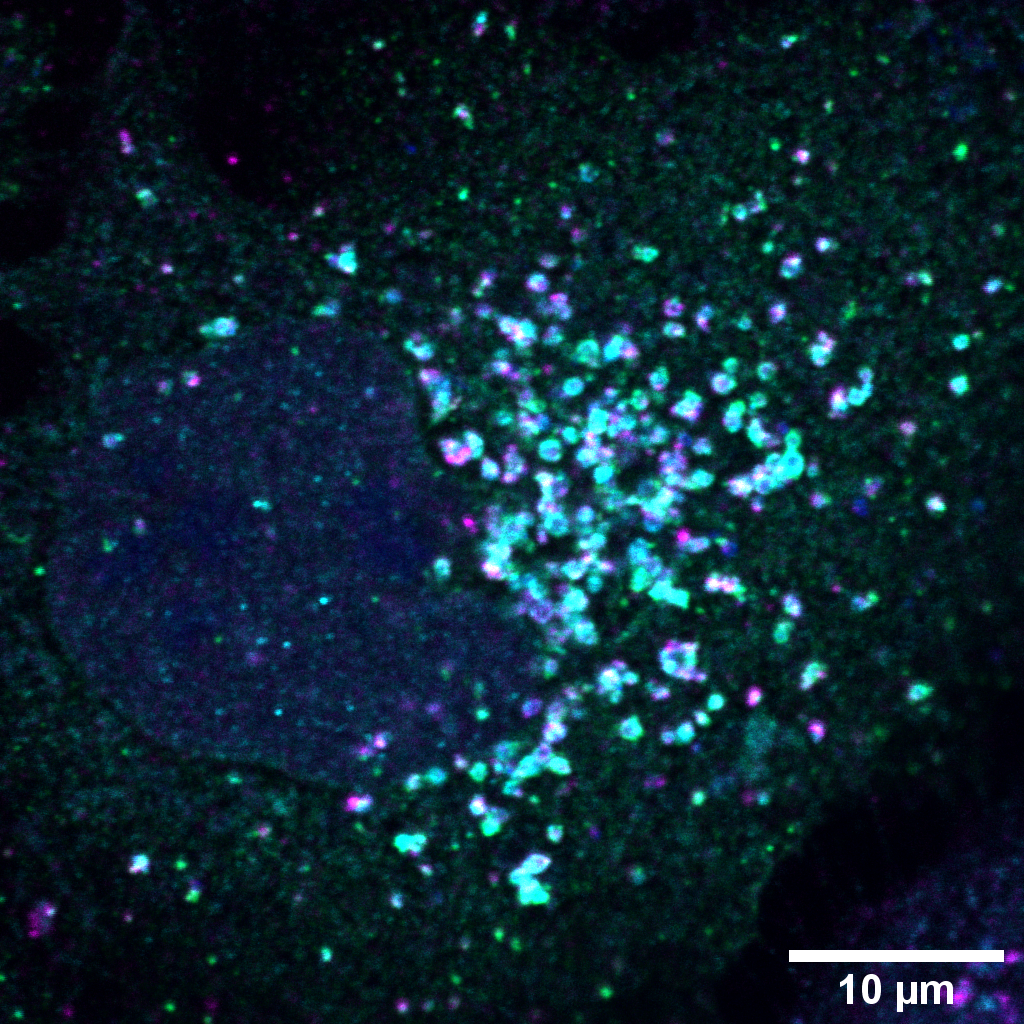

Supplement: Supplementary file 5 — Source Data for Figure 3 [file EMBR-24-e56841-s004.zip › Figure_3/3E/30min_scale.tif]

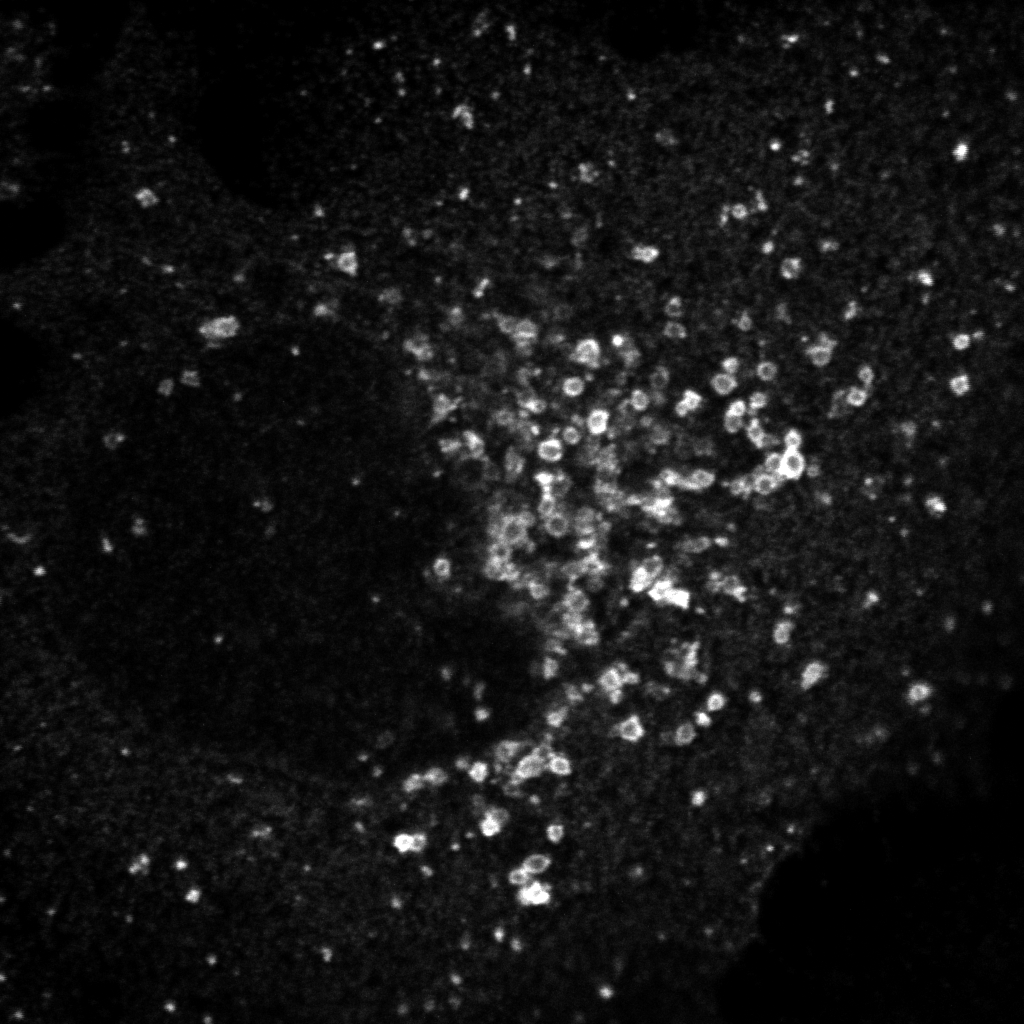

Supplement: Supplementary file 5 — Source Data for Figure 3 [file EMBR-24-e56841-s004.zip › Figure_3/3E/30min_TECPR1_green.tif]

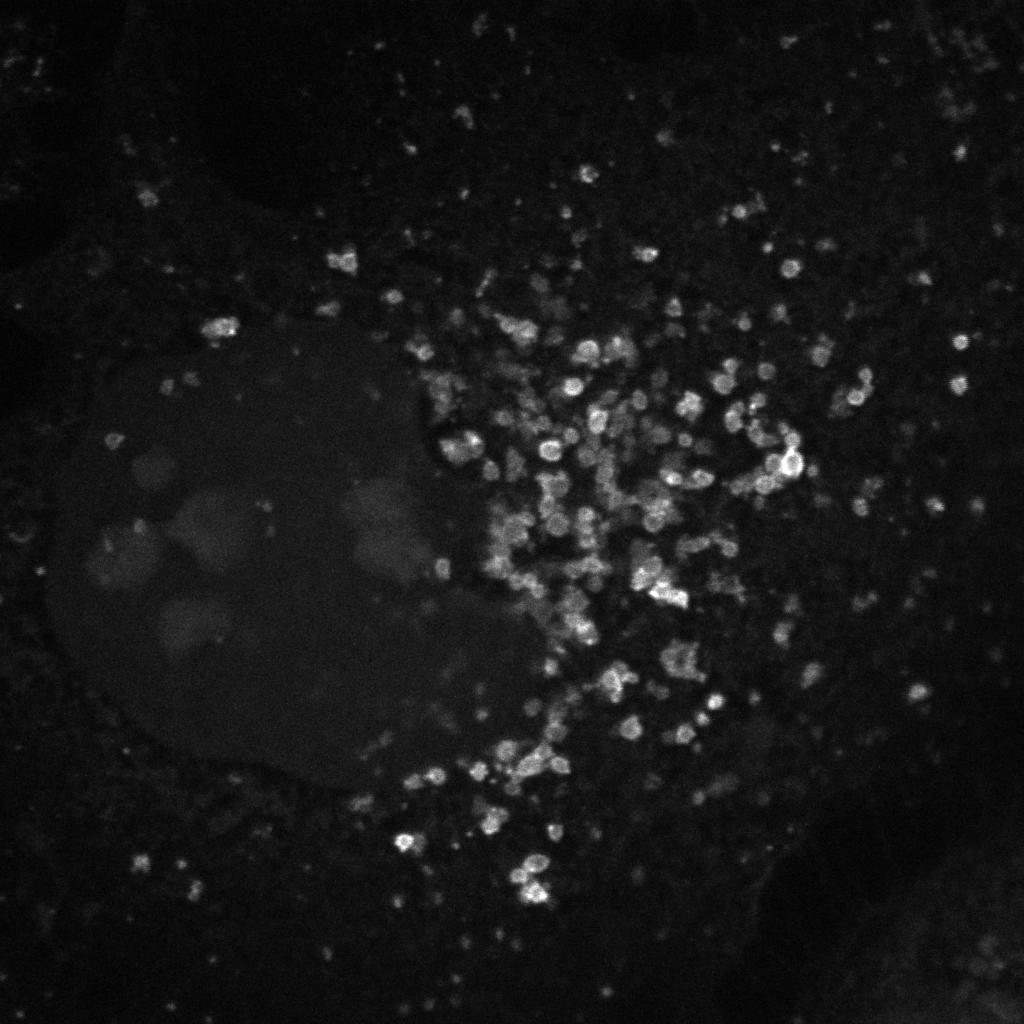

Supplement: Supplementary file 5 — Source Data for Figure 3 [file EMBR-24-e56841-s004.zip › Figure_3/3E/30min_TMEM_blue.tif]

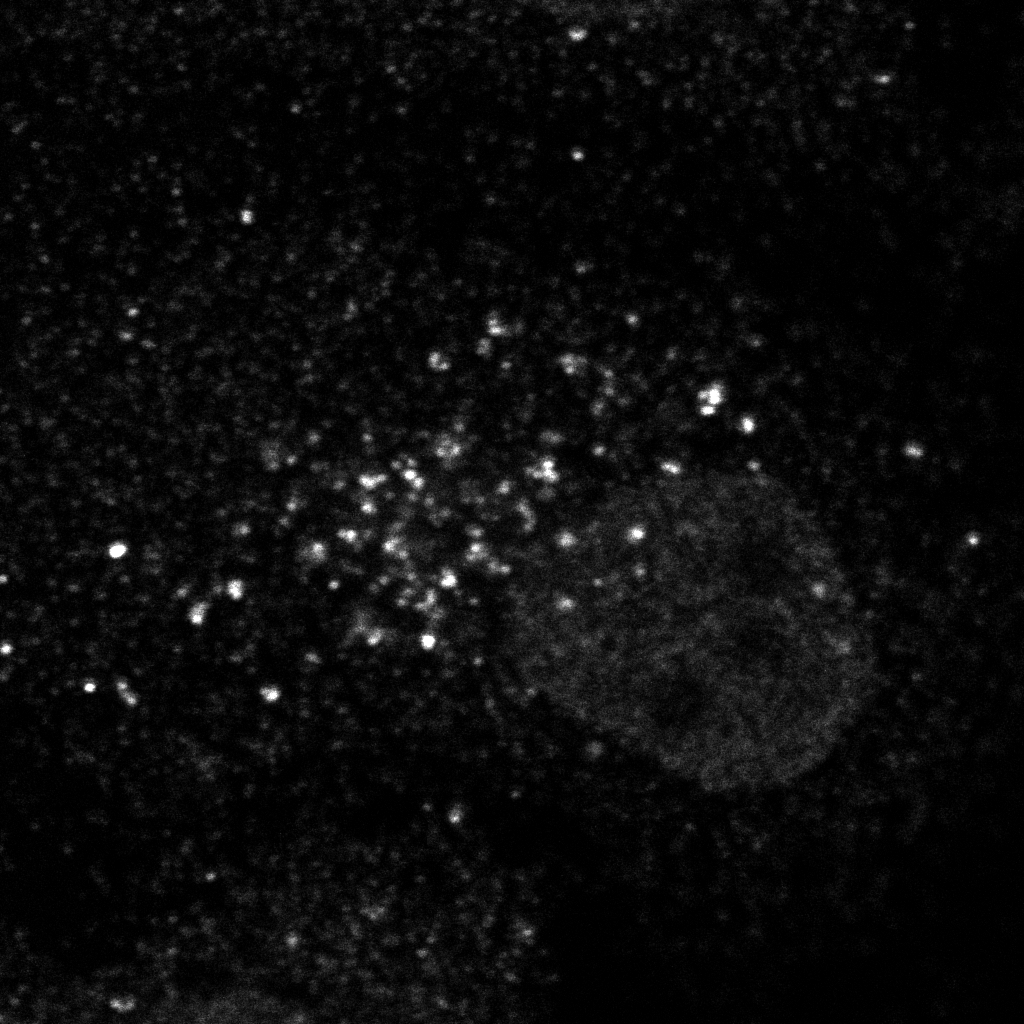

Supplement: Supplementary file 5 — Source Data for Figure 3 [file EMBR-24-e56841-s004.zip › Figure_3/3E/5min_ALIX_magenta.tif]

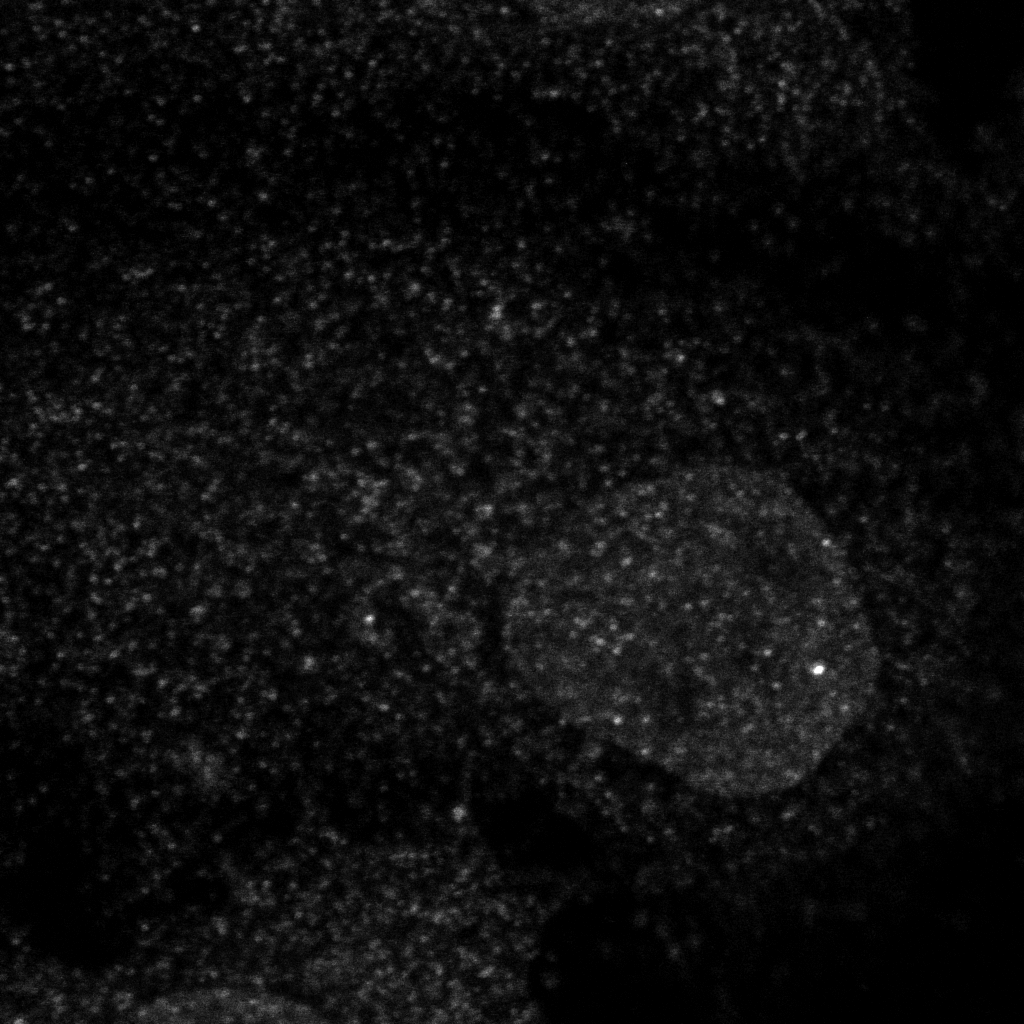

Supplement: Supplementary file 5 — Source Data for Figure 3 [file EMBR-24-e56841-s004.zip › Figure_3/3E/5min_Gal3_cyan.tif]

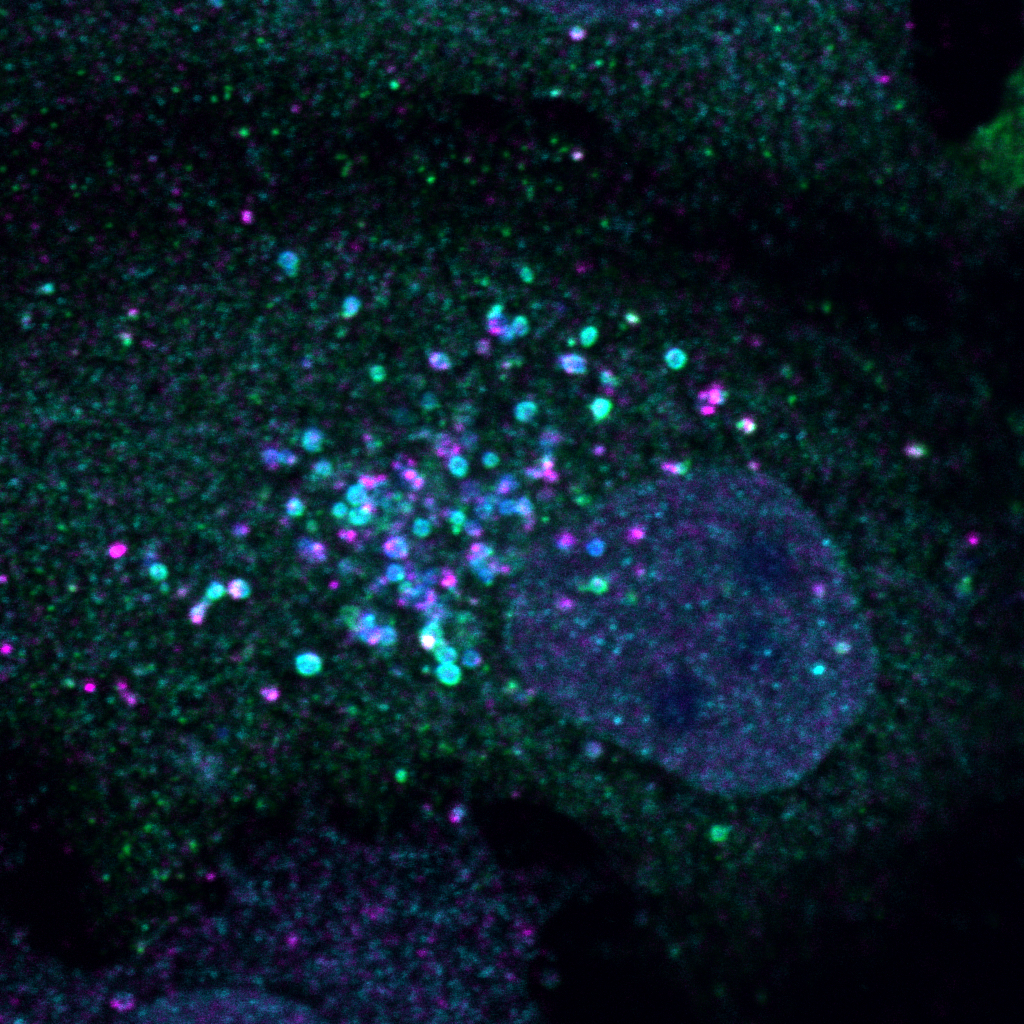

Supplement: Supplementary file 5 — Source Data for Figure 3 [file EMBR-24-e56841-s004.zip › Figure_3/3E/5min_merge.tif]

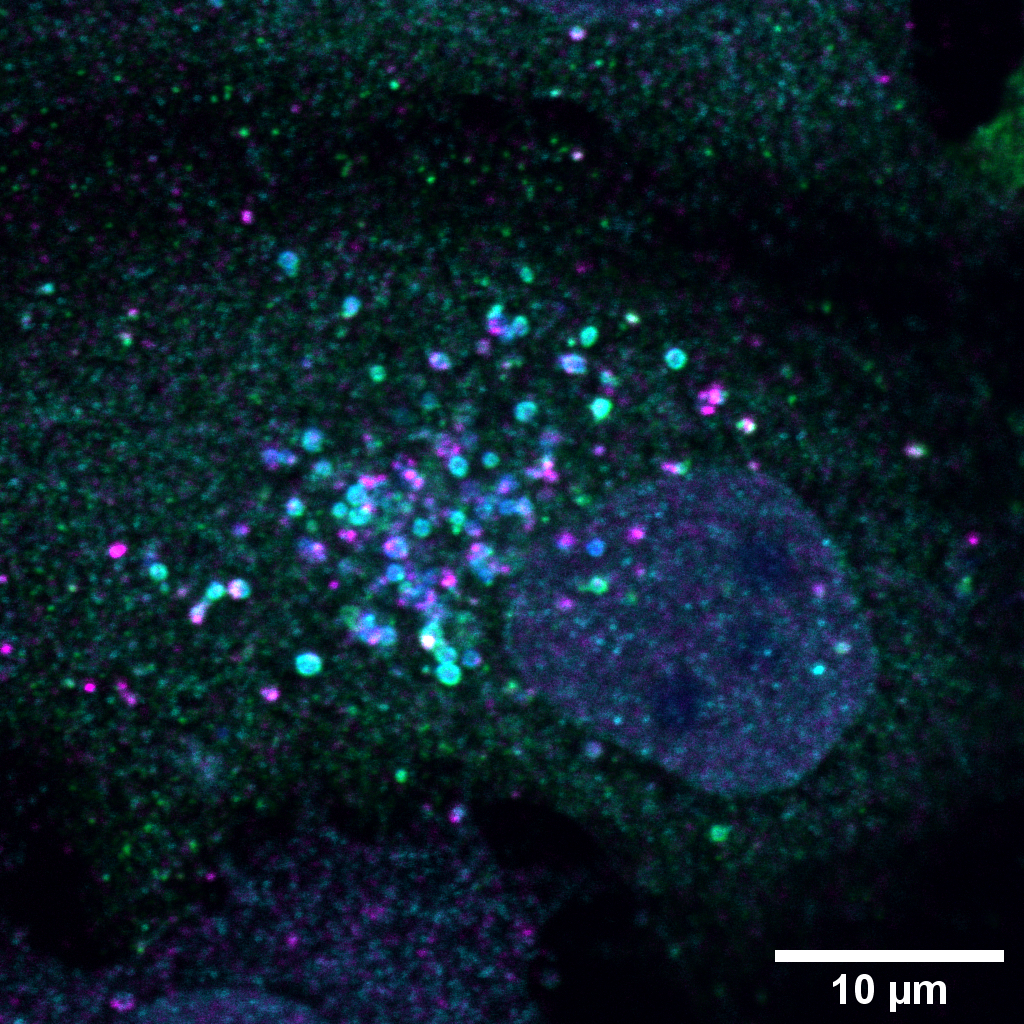

Supplement: Supplementary file 5 — Source Data for Figure 3 [file EMBR-24-e56841-s004.zip › Figure_3/3E/5min_scale.tif]

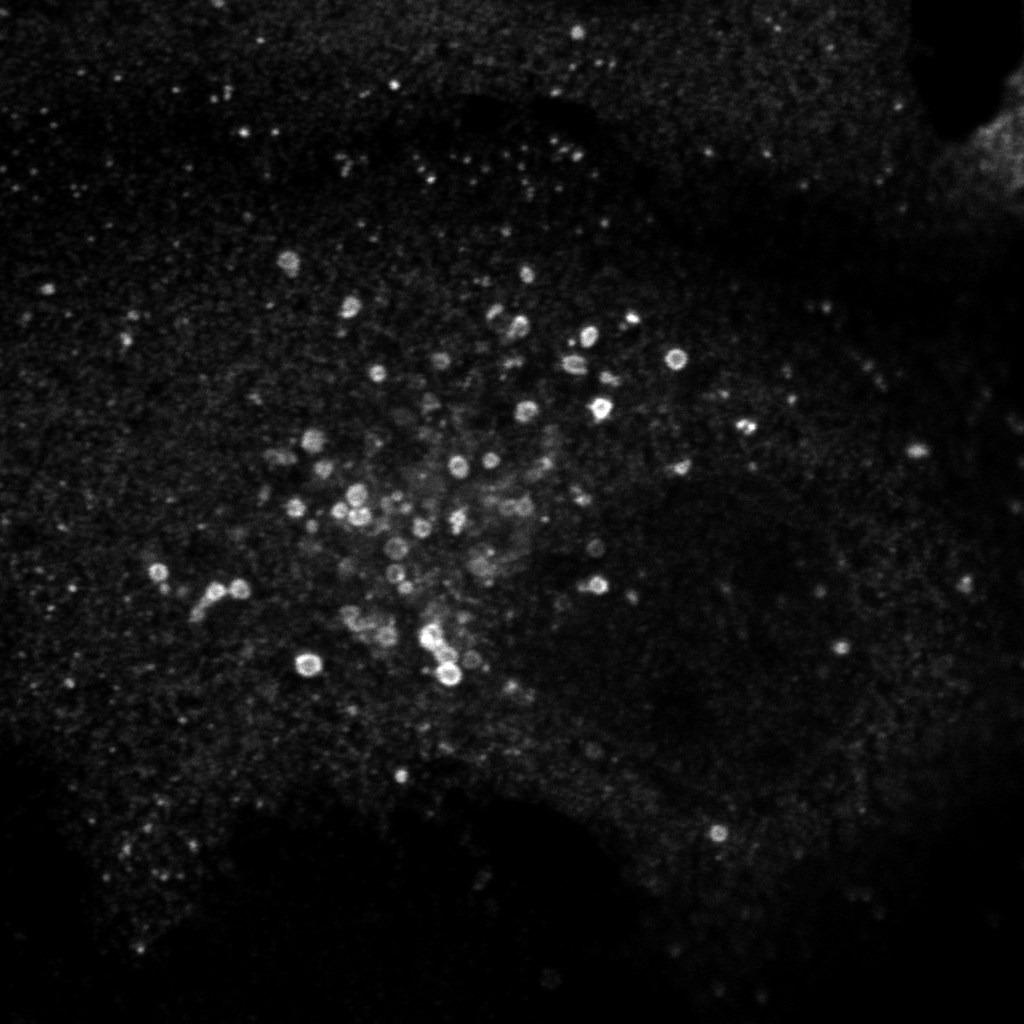

Supplement: Supplementary file 5 — Source Data for Figure 3 [file EMBR-24-e56841-s004.zip › Figure_3/3E/5min_TECPR1_green.tif]

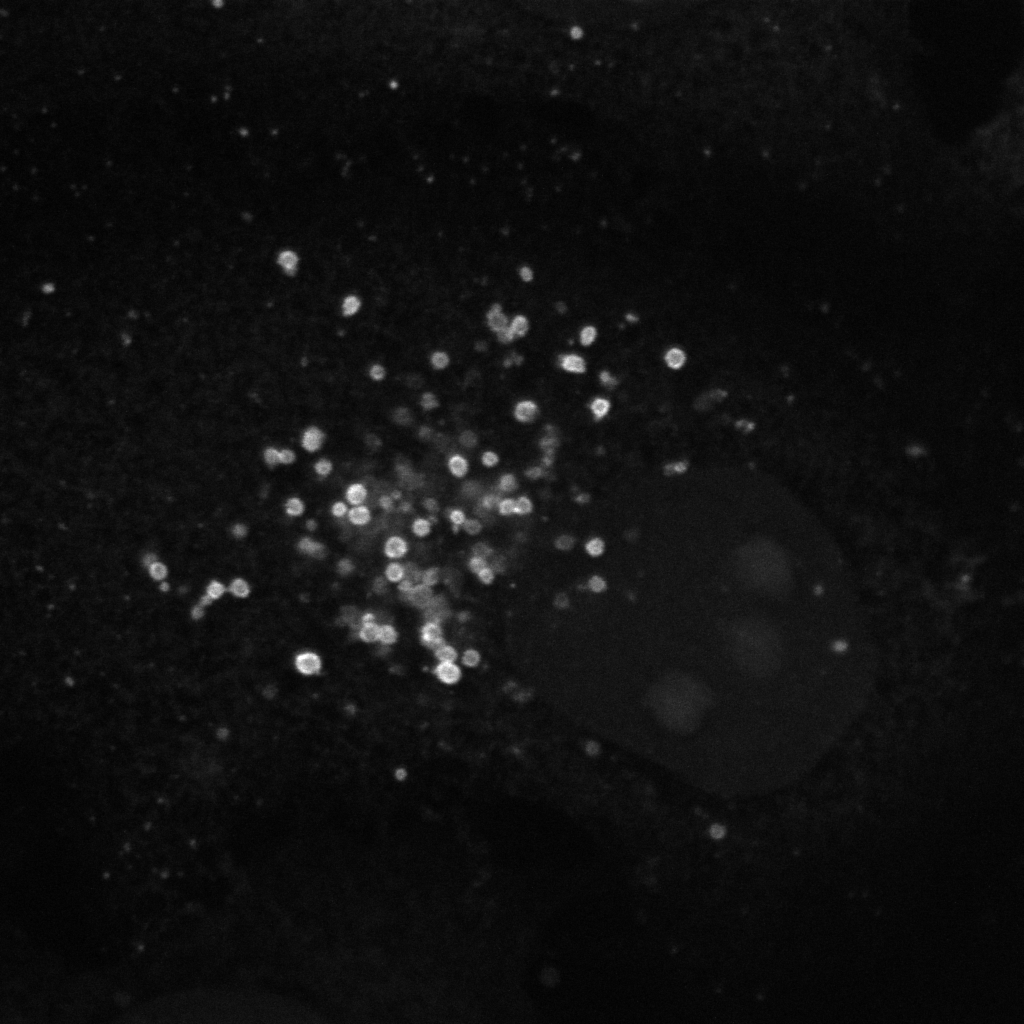

Supplement: Supplementary file 5 — Source Data for Figure 3 [file EMBR-24-e56841-s004.zip › Figure_3/3E/5min_TMEM_blue.tif]

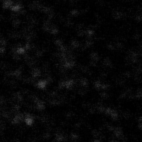

Supplement: Supplementary file 5 — Source Data for Figure 3 [file EMBR-24-e56841-s004.zip › Figure_3/3E/zoom/0min_ALIX.tif]

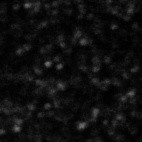

Supplement: Supplementary file 5 — Source Data for Figure 3 [file EMBR-24-e56841-s004.zip › Figure_3/3E/zoom/0min_Gal3.tif]

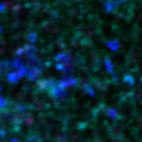

Supplement: Supplementary file 5 — Source Data for Figure 3 [file EMBR-24-e56841-s004.zip › Figure_3/3E/zoom/0min_merge.tif]

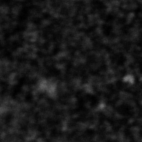

Supplement: Supplementary file 5 — Source Data for Figure 3 [file EMBR-24-e56841-s004.zip › Figure_3/3E/zoom/0min_TECPR1.tif]

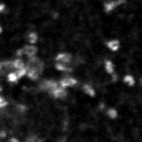

Supplement: Supplementary file 5 — Source Data for Figure 3 [file EMBR-24-e56841-s004.zip › Figure_3/3E/zoom/0min_TMEM.tif]

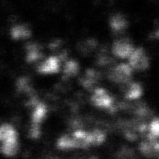

Supplement: Supplementary file 5 — Source Data for Figure 3 [file EMBR-24-e56841-s004.zip › Figure_3/3E/zoom/20min_ALIX.tif]

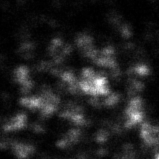

Supplement: Supplementary file 5 — Source Data for Figure 3 [file EMBR-24-e56841-s004.zip › Figure_3/3E/zoom/20min_Gal3.tif]

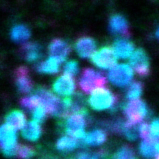

Supplement: Supplementary file 5 — Source Data for Figure 3 [file EMBR-24-e56841-s004.zip › Figure_3/3E/zoom/20min_merge.tif]

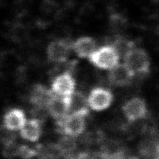

Supplement: Supplementary file 5 — Source Data for Figure 3 [file EMBR-24-e56841-s004.zip › Figure_3/3E/zoom/20min_TECPR1.tif]

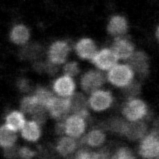

Supplement: Supplementary file 5 — Source Data for Figure 3 [file EMBR-24-e56841-s004.zip › Figure_3/3E/zoom/20min_TMEM.tif]

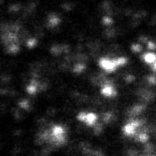

Supplement: Supplementary file 5 — Source Data for Figure 3 [file EMBR-24-e56841-s004.zip › Figure_3/3E/zoom/5min_ALIX.tif]

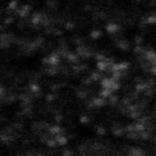

Supplement: Supplementary file 5 — Source Data for Figure 3 [file EMBR-24-e56841-s004.zip › Figure_3/3E/zoom/5min_Gal3.tif]

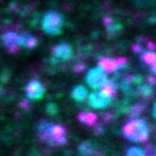

Supplement: Supplementary file 5 — Source Data for Figure 3 [file EMBR-24-e56841-s004.zip › Figure_3/3E/zoom/5min_merge.tif]

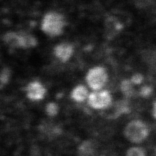

Supplement: Supplementary file 5 — Source Data for Figure 3 [file EMBR-24-e56841-s004.zip › Figure_3/3E/zoom/5min_TECPR1.tif]

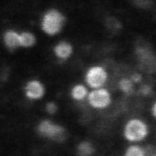

Supplement: Supplementary file 5 — Source Data for Figure 3 [file EMBR-24-e56841-s004.zip › Figure_3/3E/zoom/5min_TMEM.tif]

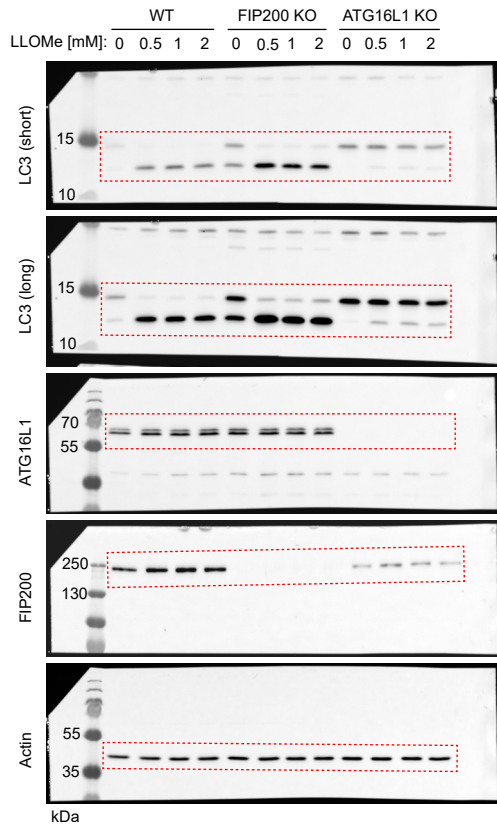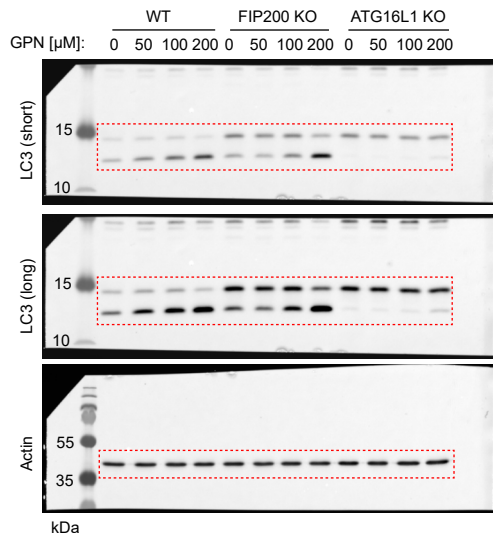

Supplement: Supplementary file 6 — Source Data for Figure 4 [file EMBR-24-e56841-s008.zip › Figure_4/4A/4A.pdf]

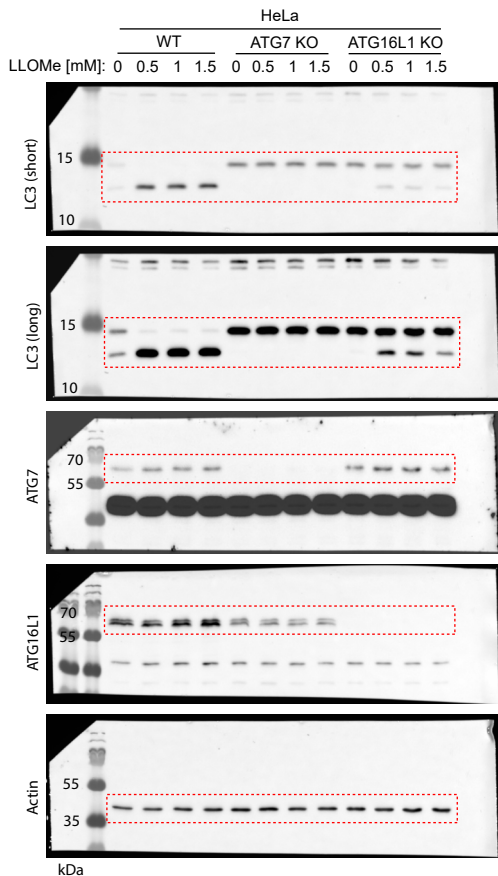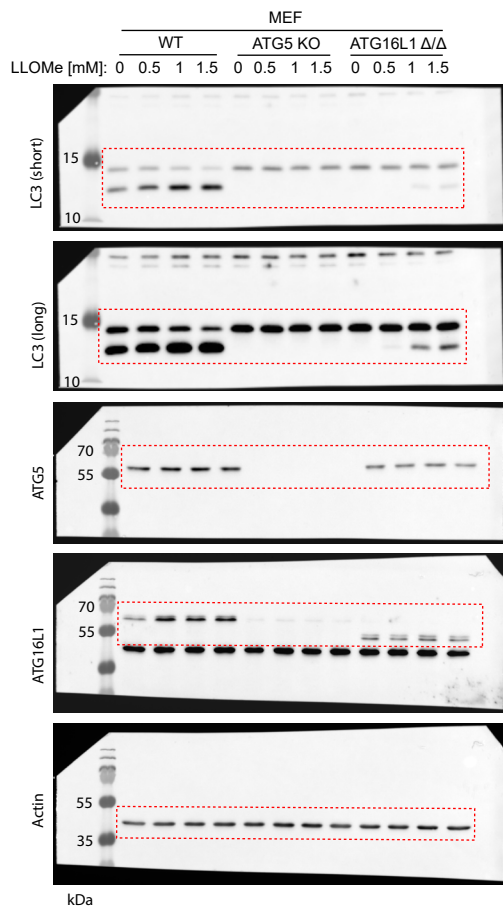

Supplement: Supplementary file 6 — Source Data for Figure 4 [file EMBR-24-e56841-s008.zip › Figure_4/4C/4C.pdf]

|                         |   |   |   |   |   |   |   |   |   |   |   |   |
|-------------------------|---|---|---|---|---|---|---|---|---|---|---|---|
| LLOMe pre-treat [1 mM]: | - | - | - | - | + | + | + | + | + | + | + | + |
| CQ [20 $\mu$ M]:        | - | + | + | + | - | - | - | - | - | + | + | + |
| Time post-washout (h):  | 0 | 4 | 6 | 8 | 0 | 4 | 6 | 8 | 0 | 4 | 6 | 8 |

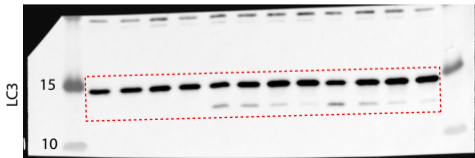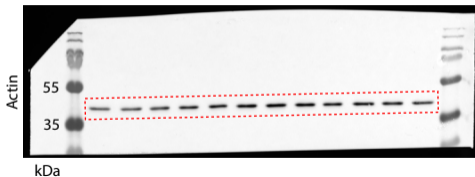

Supplement: Supplementary file 6 — Source Data for Figure 4 [file EMBR-24-e56841-s008.zip › Figure_4/4D/4D.pdf]

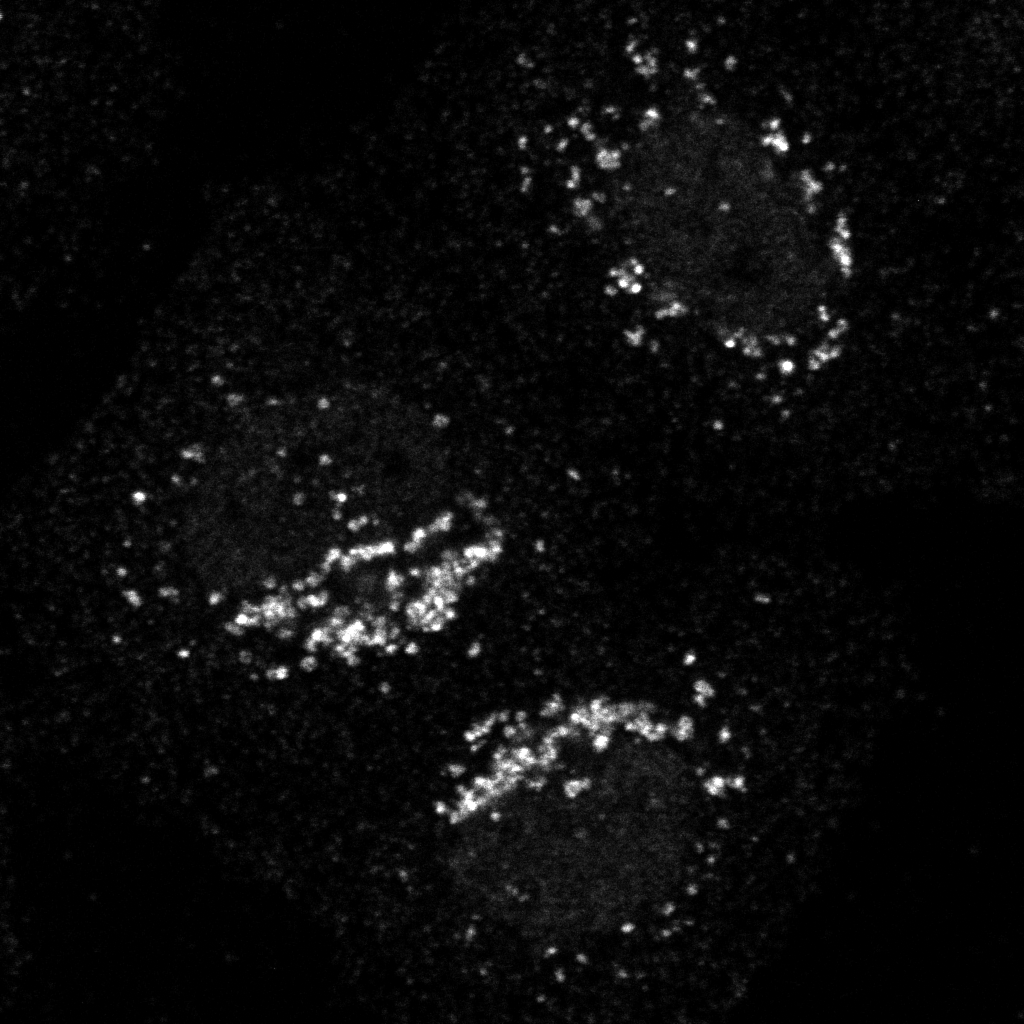

Supplement: Supplementary file 6 — Source Data for Figure 4 [file EMBR-24-e56841-s008.zip › Figure_4/4E/Image_Data/ATG16KO_LLOMe_LAMP.tif]

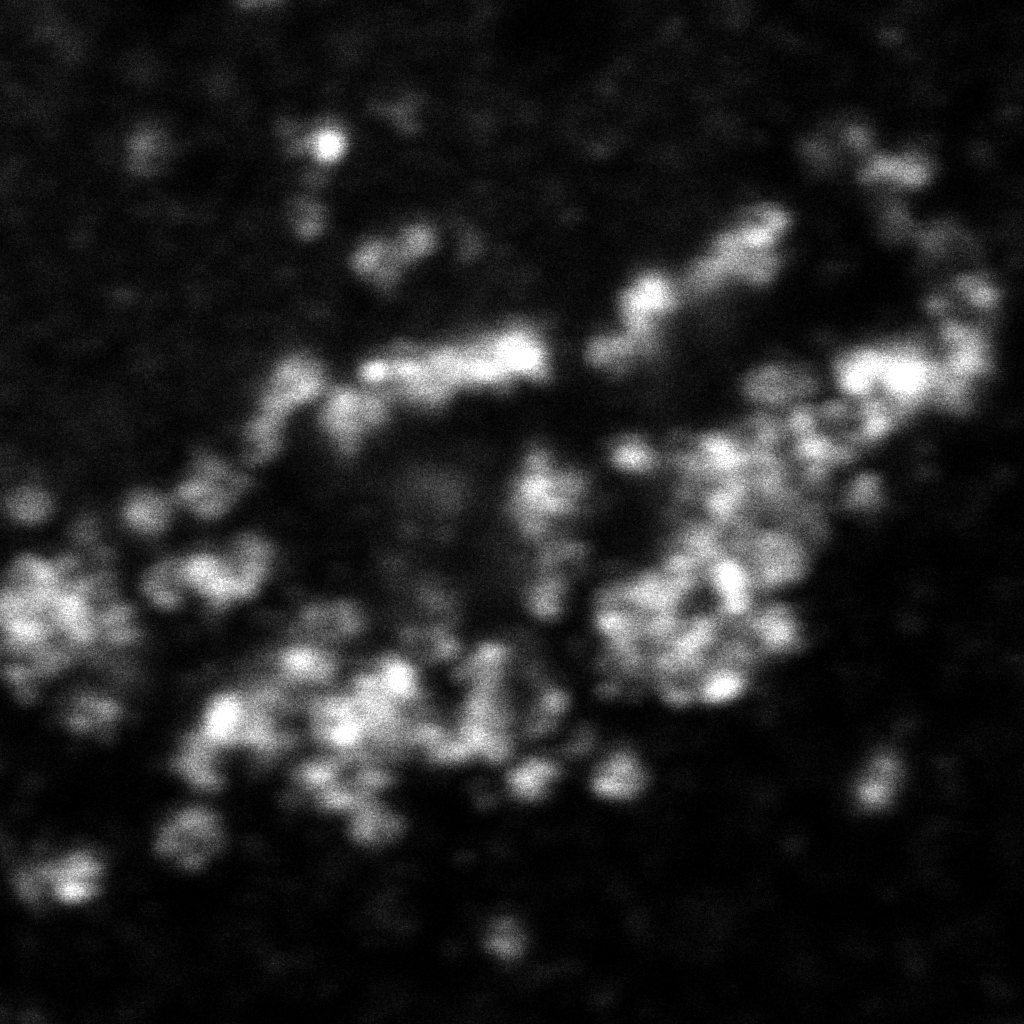

Supplement: Supplementary file 6 — Source Data for Figure 4 [file EMBR-24-e56841-s008.zip › Figure_4/4E/Image_Data/ATG16KO_LLOMe_LAMP_zoom.tif]

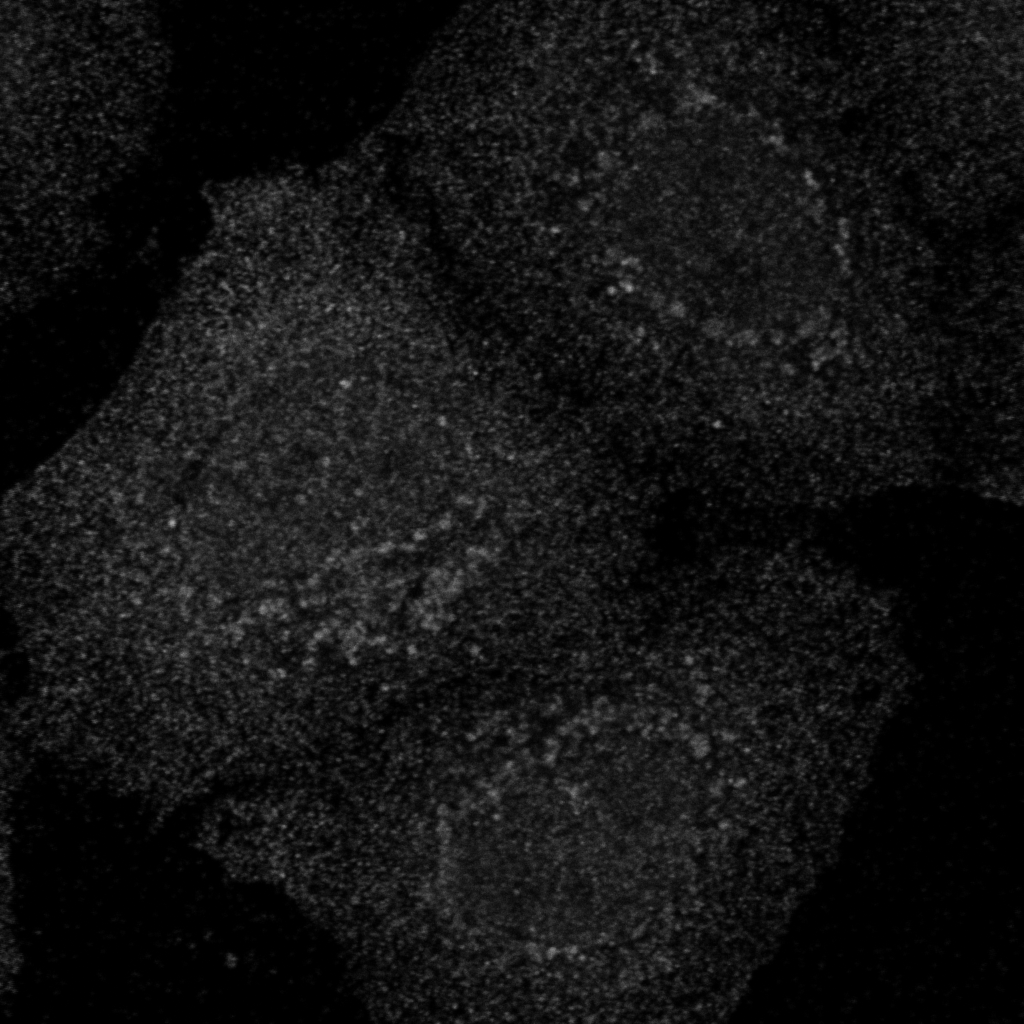

Supplement: Supplementary file 6 — Source Data for Figure 4 [file EMBR-24-e56841-s008.zip › Figure_4/4E/Image_Data/ATG16KO_LLOMe_LC3.tif]

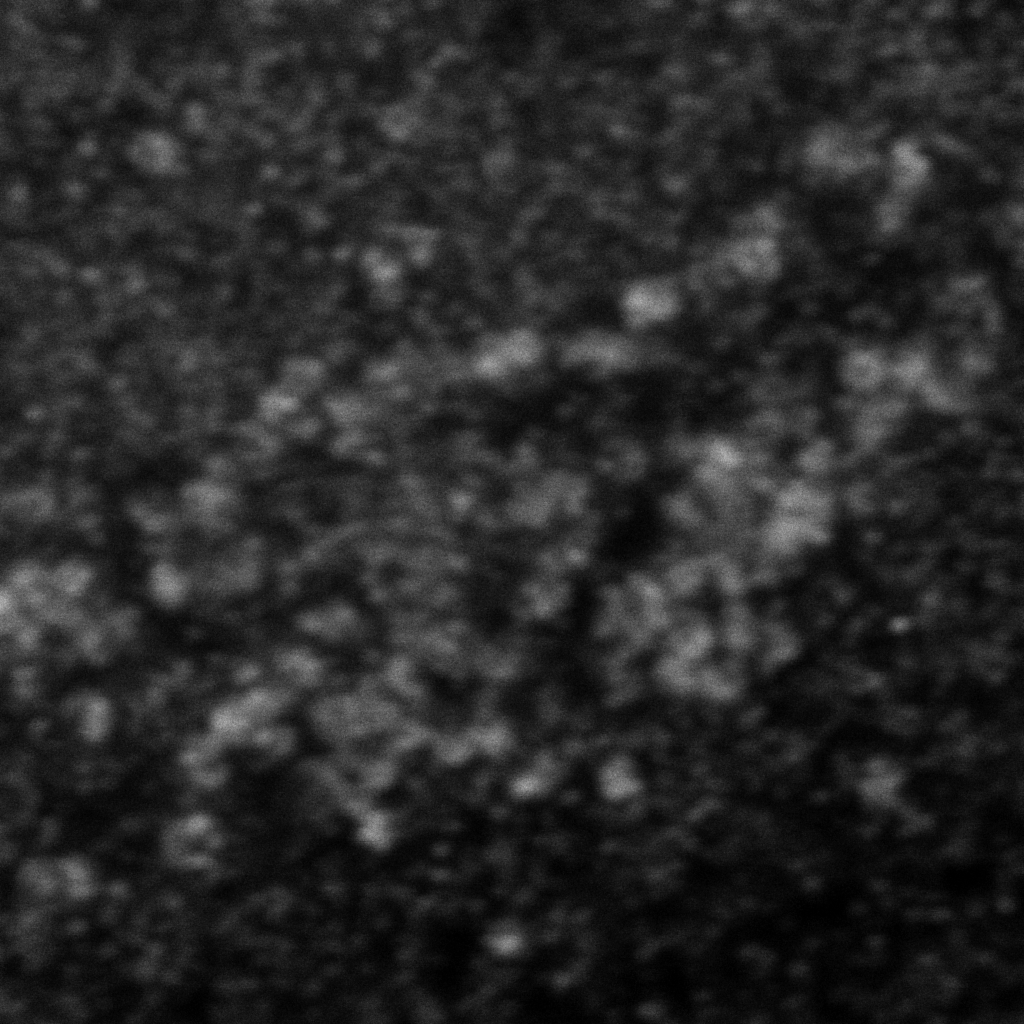

Supplement: Supplementary file 6 — Source Data for Figure 4 [file EMBR-24-e56841-s008.zip › Figure_4/4E/Image_Data/ATG16KO_LLOMe_LC3_zoom.tif]

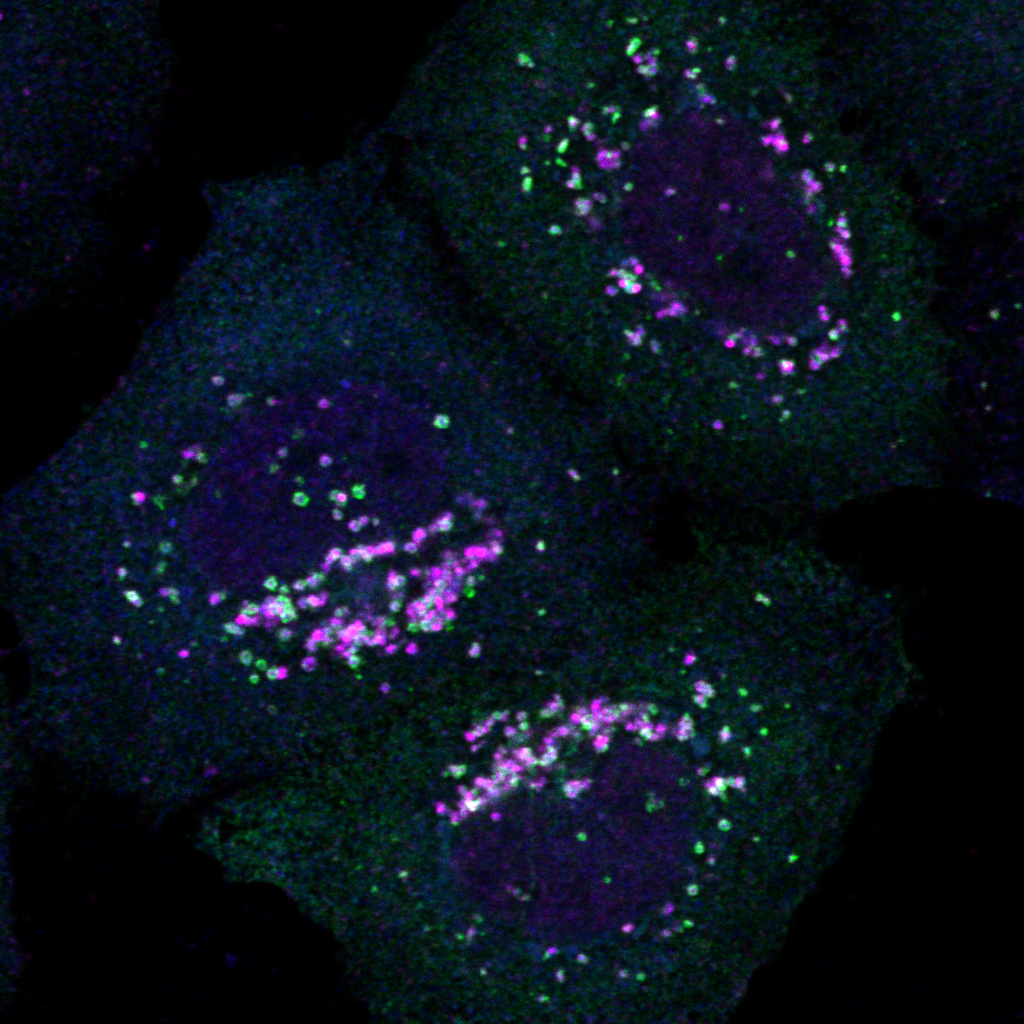

Supplement: Supplementary file 6 — Source Data for Figure 4 [file EMBR-24-e56841-s008.zip › Figure_4/4E/Image_Data/ATG16KO_LLOMe_merge.tif]

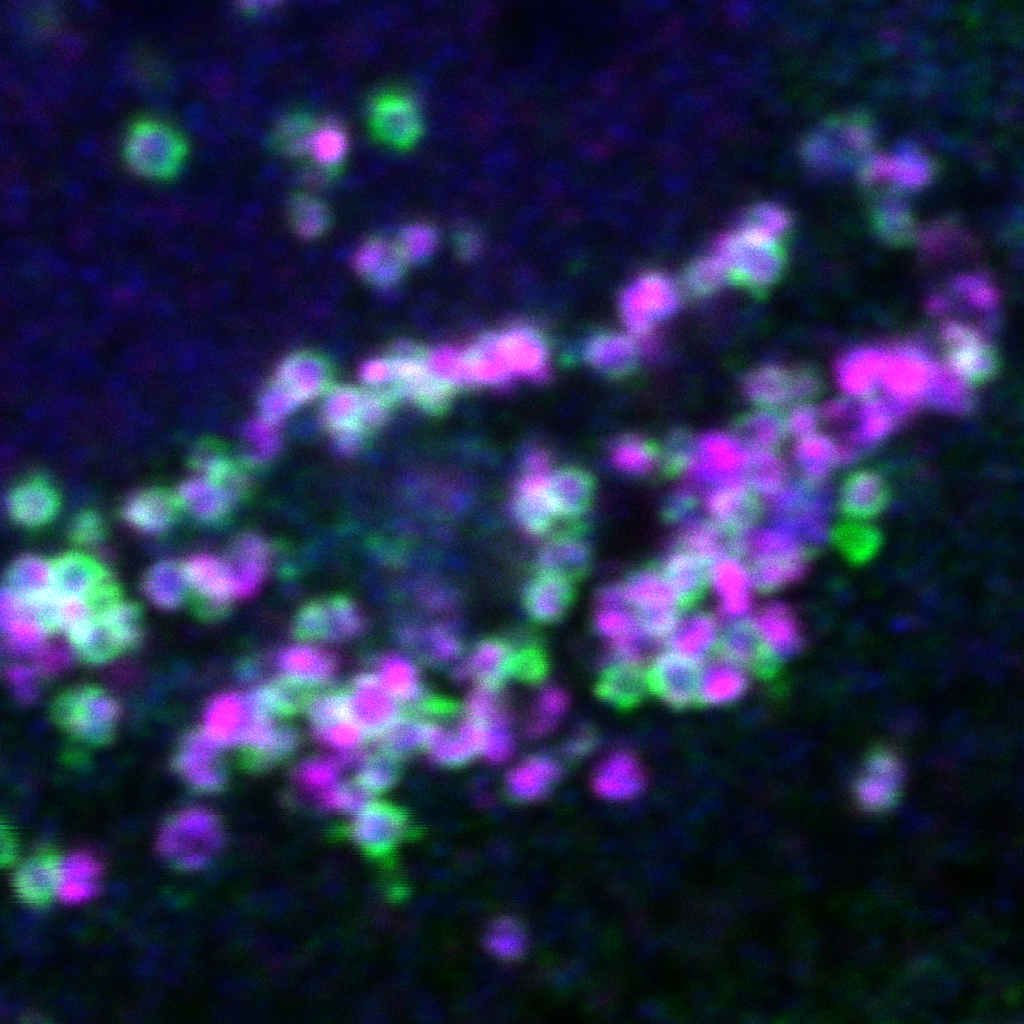

Supplement: Supplementary file 6 — Source Data for Figure 4 [file EMBR-24-e56841-s008.zip › Figure_4/4E/Image_Data/ATG16KO_LLOMe_merge_zoom.tif]

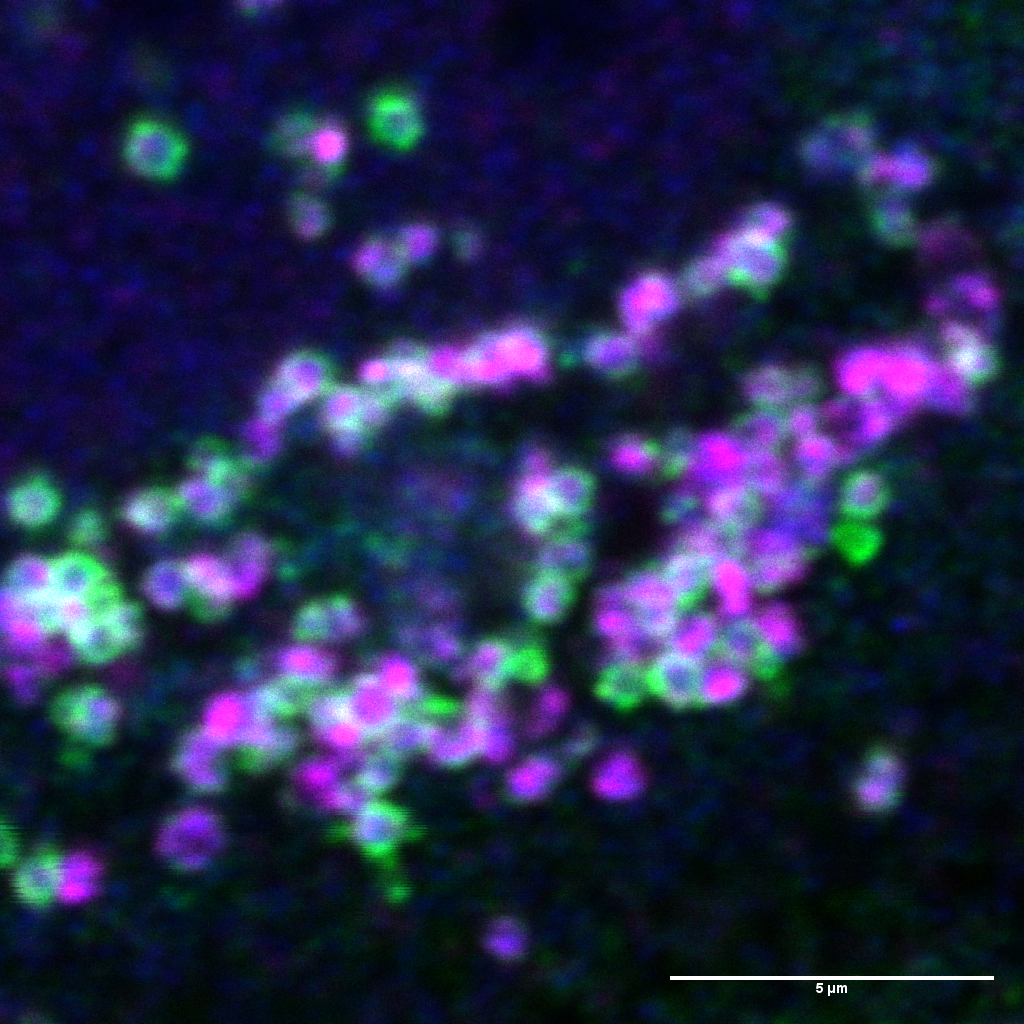

Supplement: Supplementary file 6 — Source Data for Figure 4 [file EMBR-24-e56841-s008.zip › Figure_4/4E/Image_Data/ATG16KO_LLOMe_merge_zoom_scale.tif]

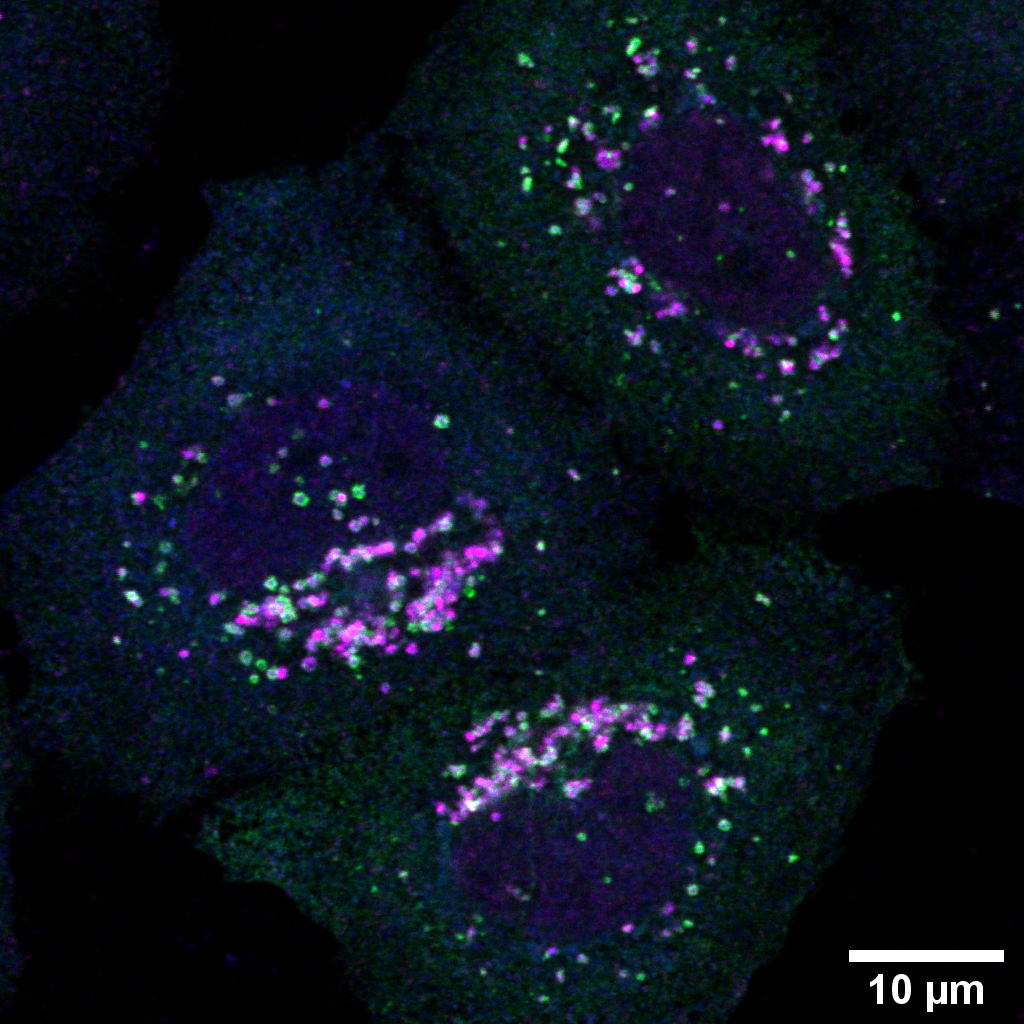

Supplement: Supplementary file 6 — Source Data for Figure 4 [file EMBR-24-e56841-s008.zip › Figure_4/4E/Image_Data/ATG16KO_LLOMe_scale.tif]

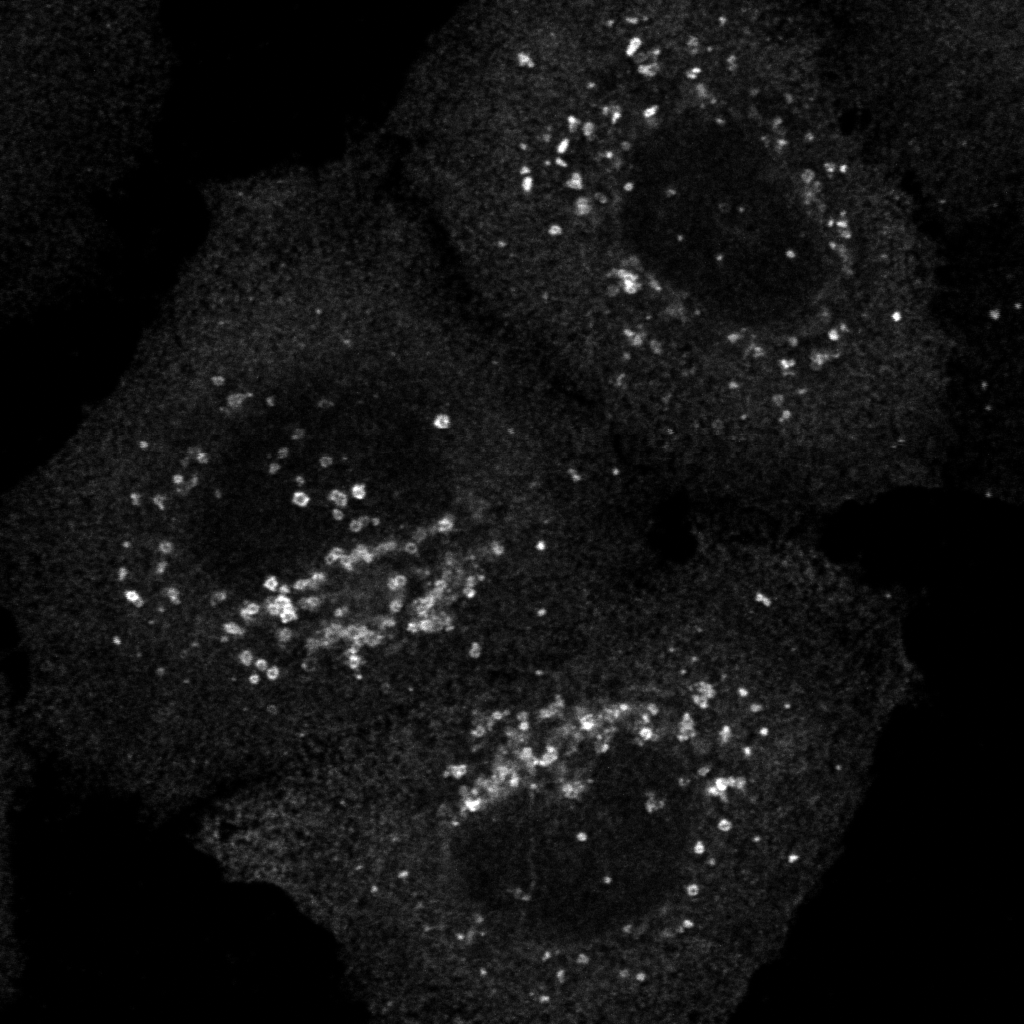

Supplement: Supplementary file 6 — Source Data for Figure 4 [file EMBR-24-e56841-s008.zip › Figure_4/4E/Image_Data/ATG16KO_LLOMe_TECPR1.tif]

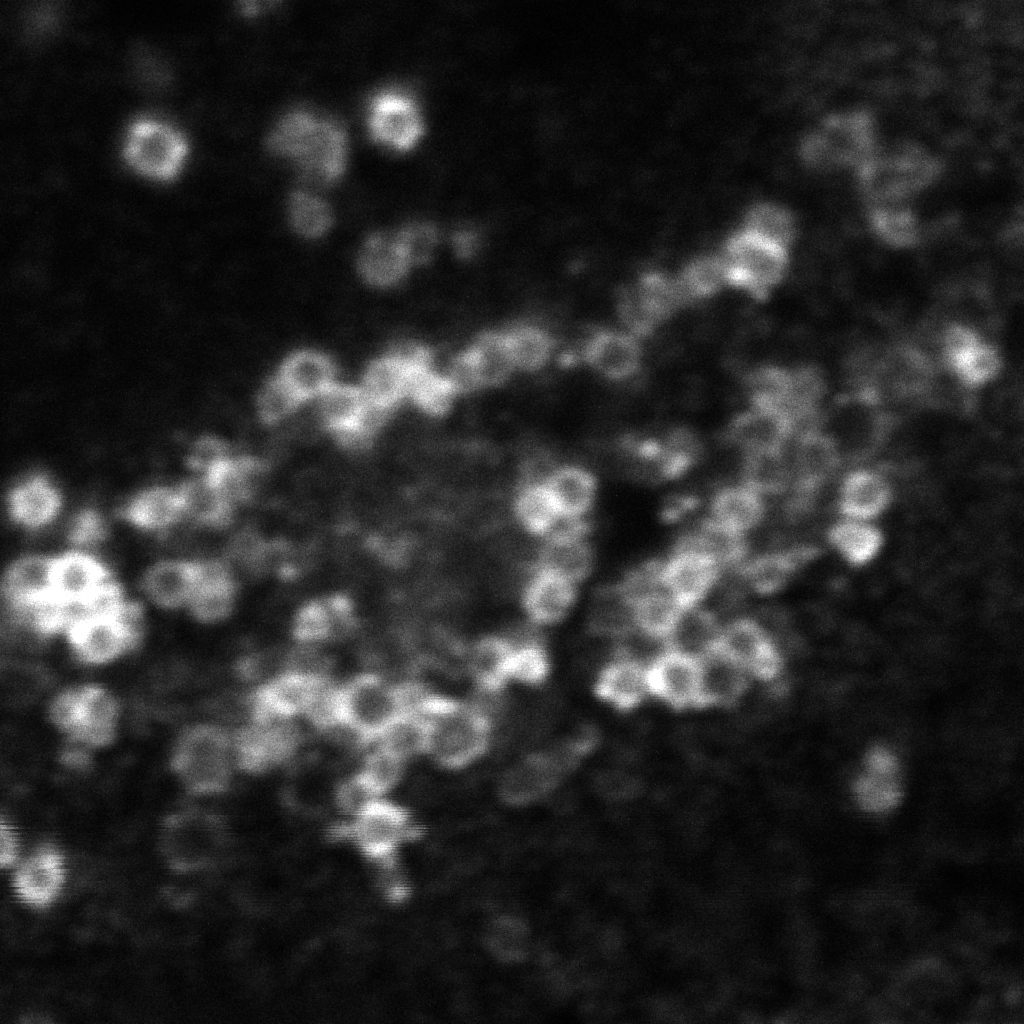

Supplement: Supplementary file 6 — Source Data for Figure 4 [file EMBR-24-e56841-s008.zip › Figure_4/4E/Image_Data/ATG16KO_LLOMe_TECPR1_zoom.tif]

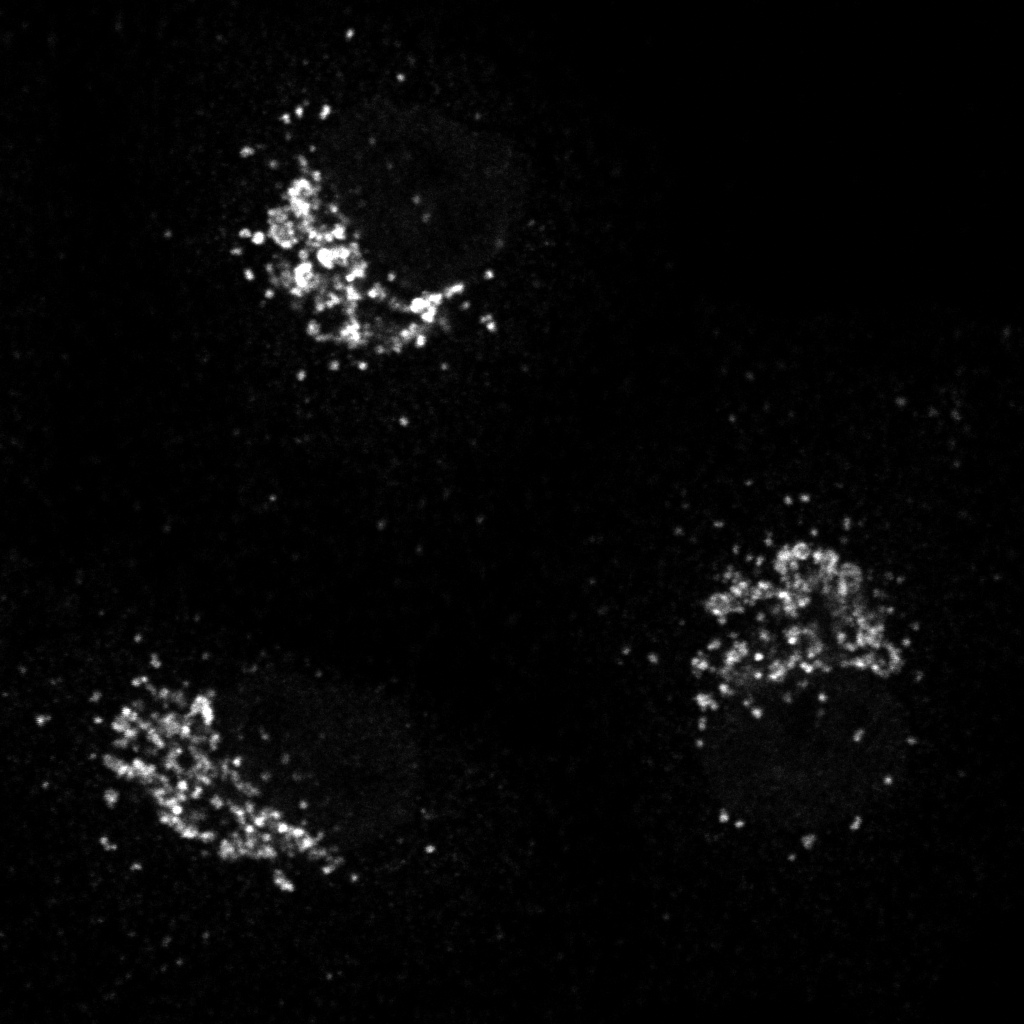

Supplement: Supplementary file 6 — Source Data for Figure 4 [file EMBR-24-e56841-s008.zip › Figure_4/4E/Image_Data/FIP200KO_LLOMe_LAMP.tif]

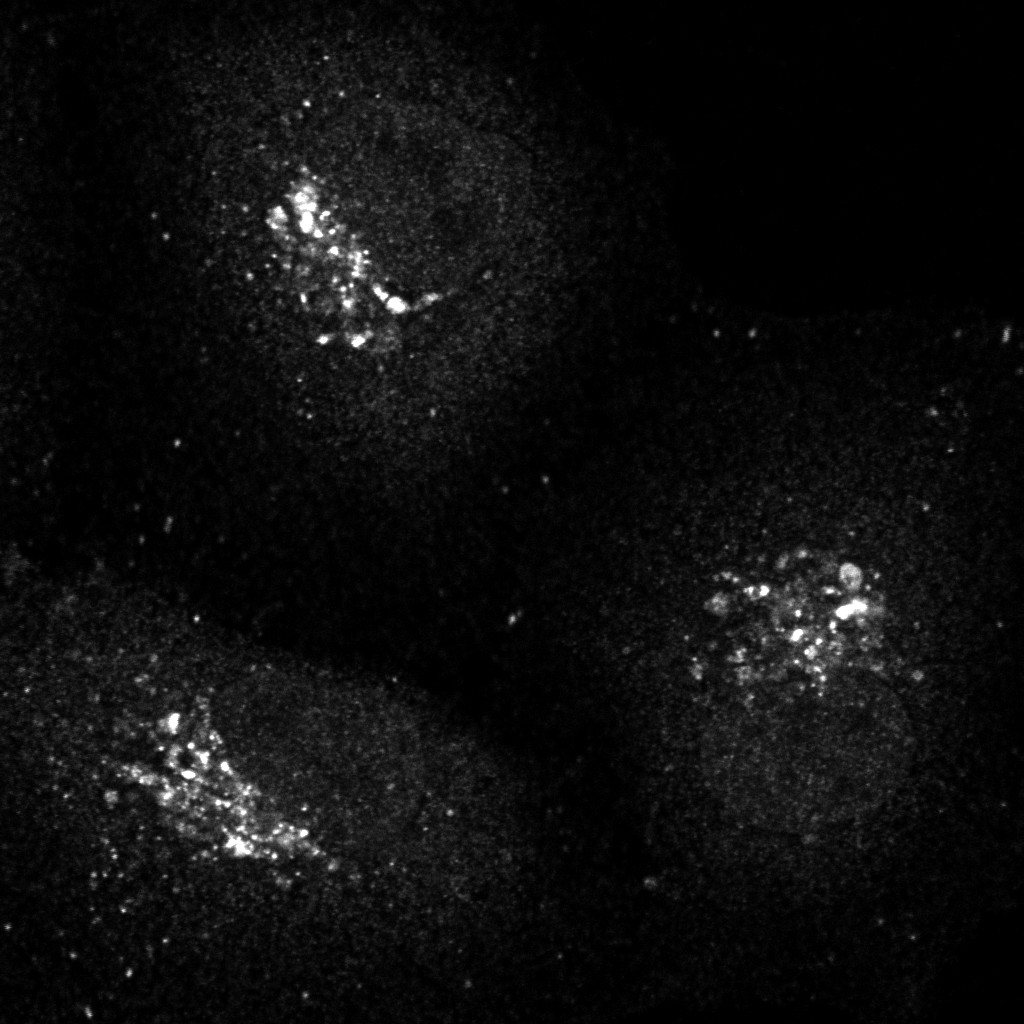

Supplement: Supplementary file 6 — Source Data for Figure 4 [file EMBR-24-e56841-s008.zip › Figure_4/4E/Image_Data/FIP200KO_LLOMe_LC3.tif]

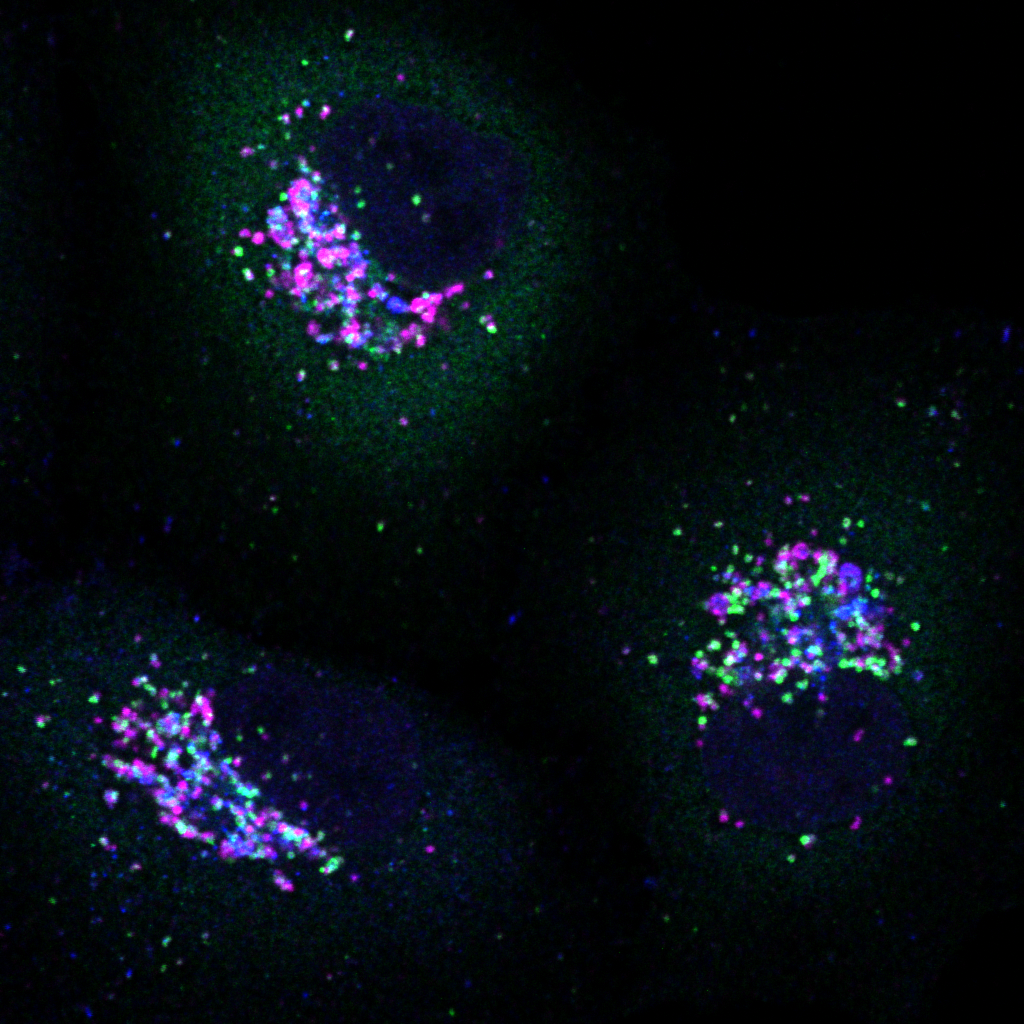

Supplement: Supplementary file 6 — Source Data for Figure 4 [file EMBR-24-e56841-s008.zip › Figure_4/4E/Image_Data/FIP200KO_LLOMe_merge.tif]

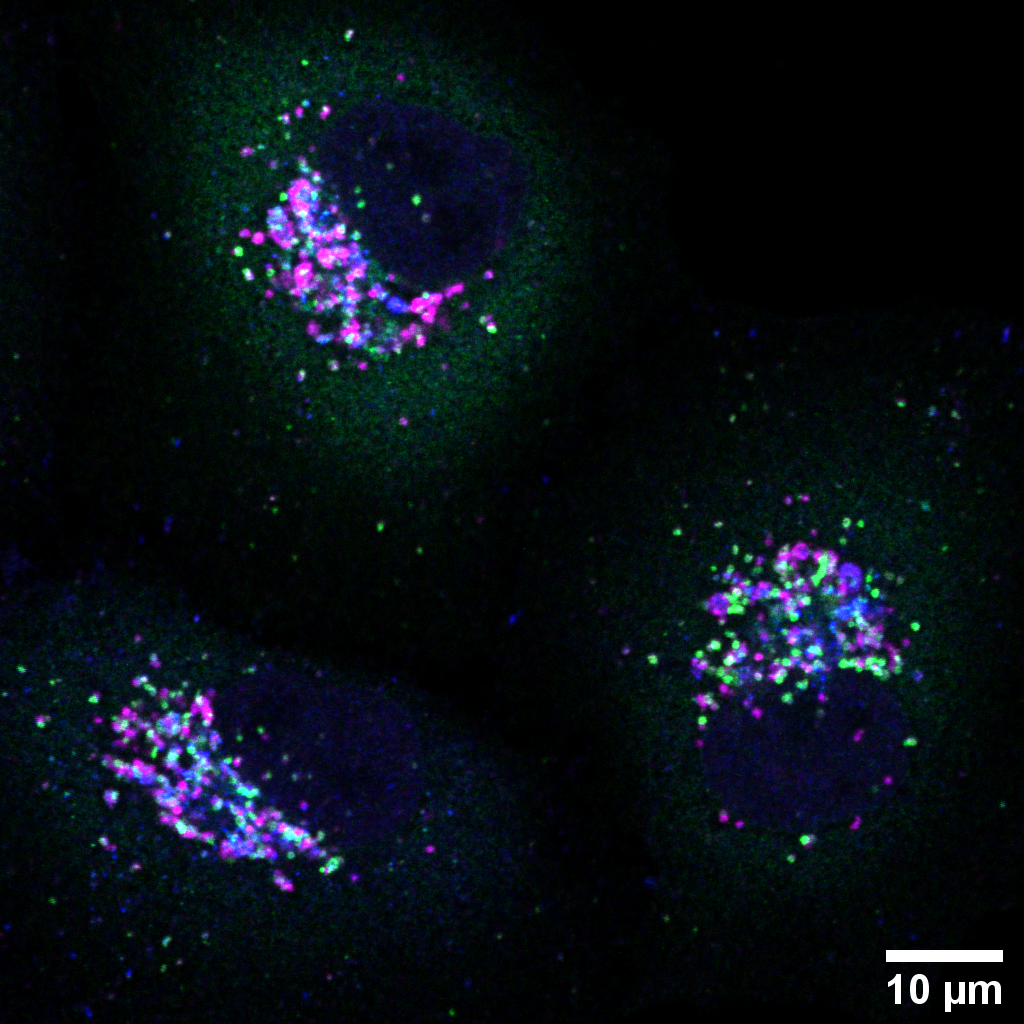

Supplement: Supplementary file 6 — Source Data for Figure 4 [file EMBR-24-e56841-s008.zip › Figure_4/4E/Image_Data/FIP200KO_LLOMe_scale.tif]

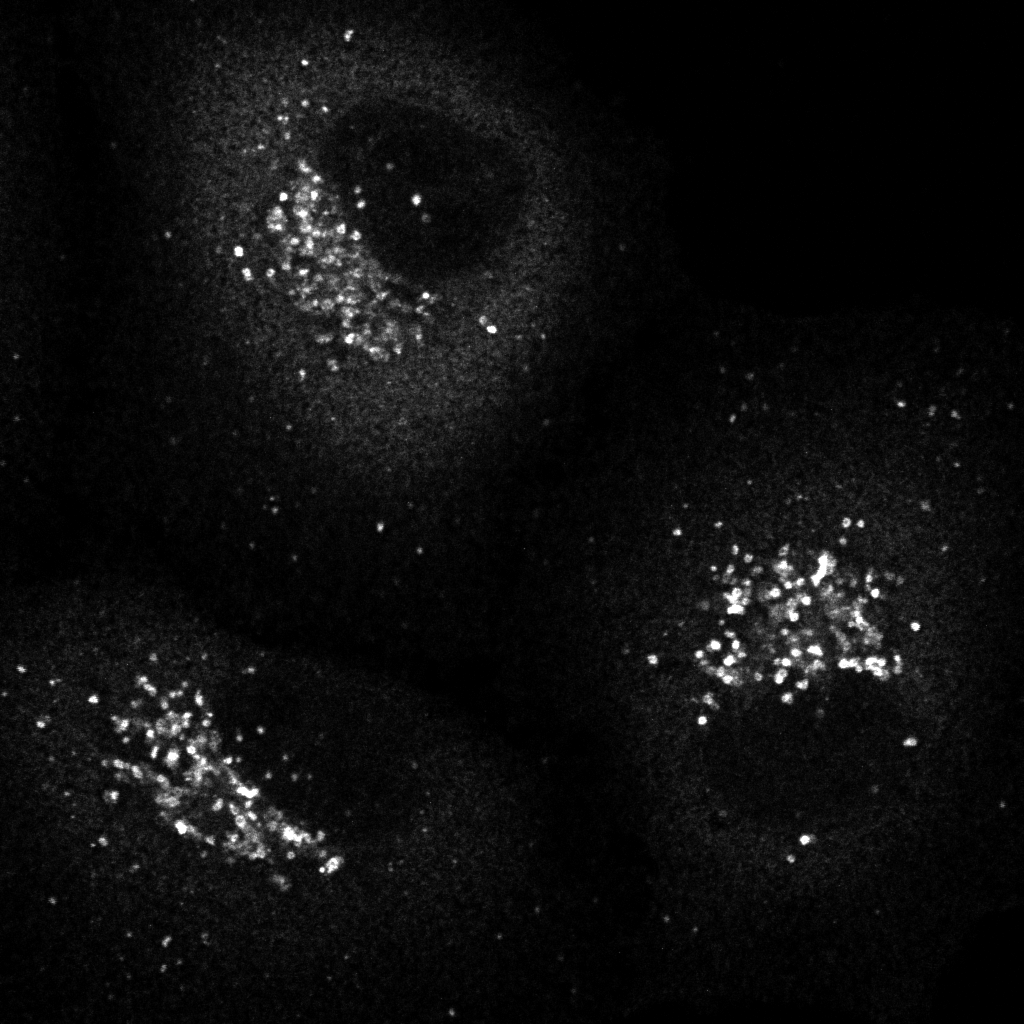

Supplement: Supplementary file 6 — Source Data for Figure 4 [file EMBR-24-e56841-s008.zip › Figure_4/4E/Image_Data/FIP200KO_LLOMe_TECPR1.tif]

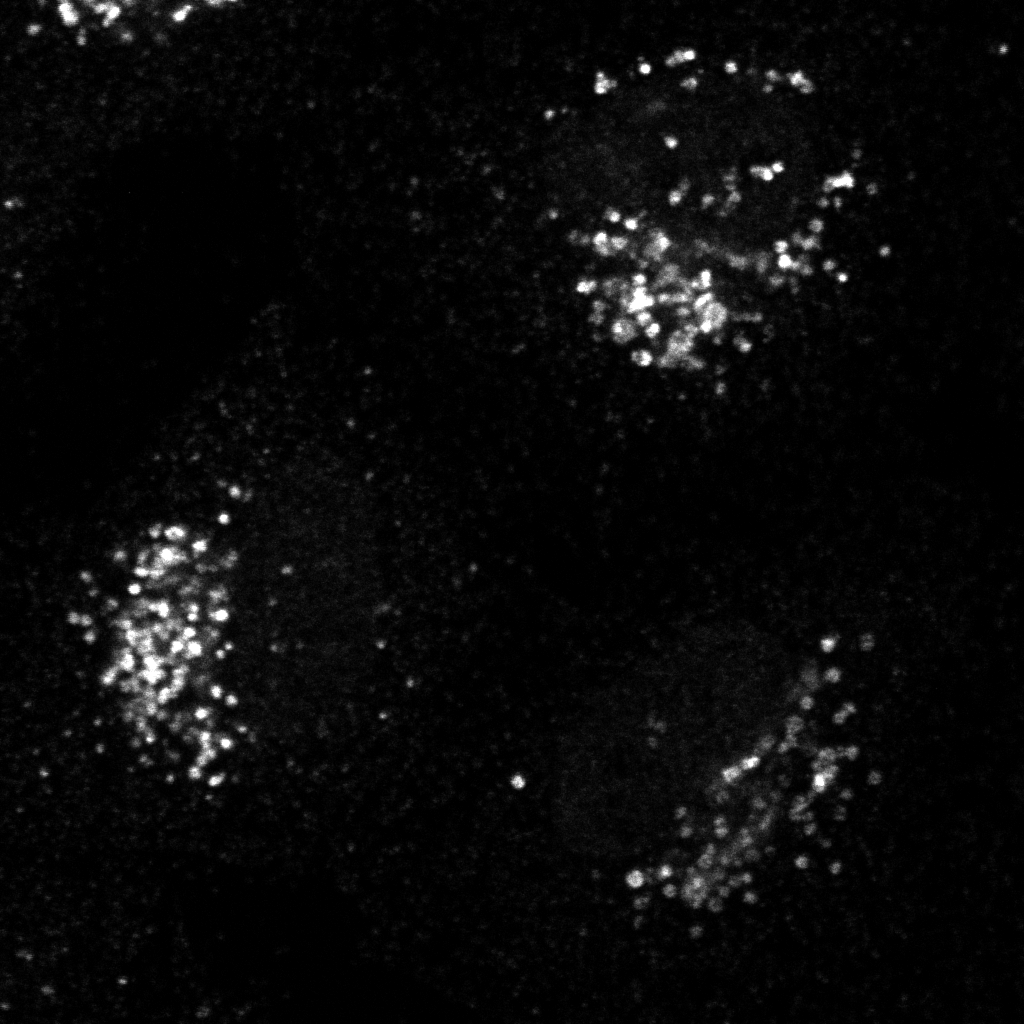

Supplement: Supplementary file 6 — Source Data for Figure 4 [file EMBR-24-e56841-s008.zip › Figure_4/4E/Image_Data/WT_LLOMe_LAMP.tif]

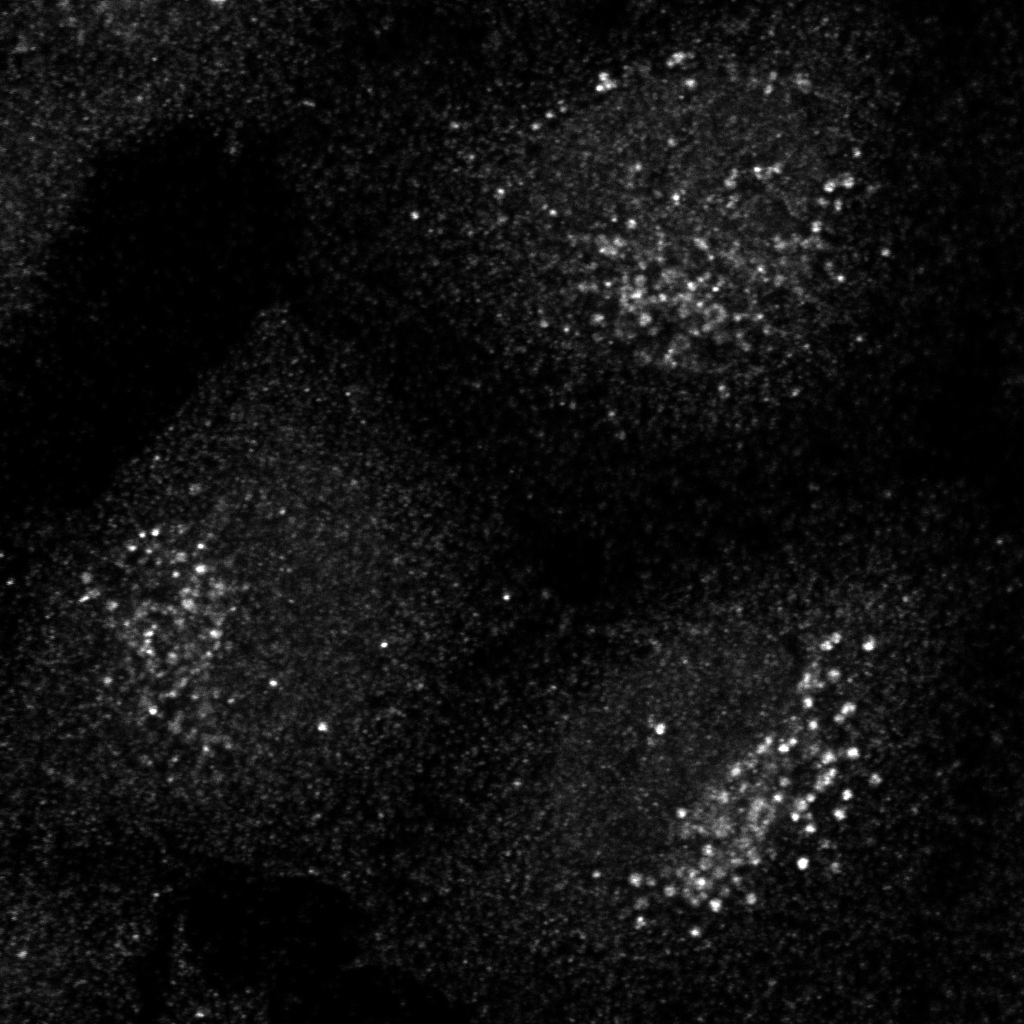

Supplement: Supplementary file 6 — Source Data for Figure 4 [file EMBR-24-e56841-s008.zip › Figure_4/4E/Image_Data/WT_LLOMe_LC3.tif]

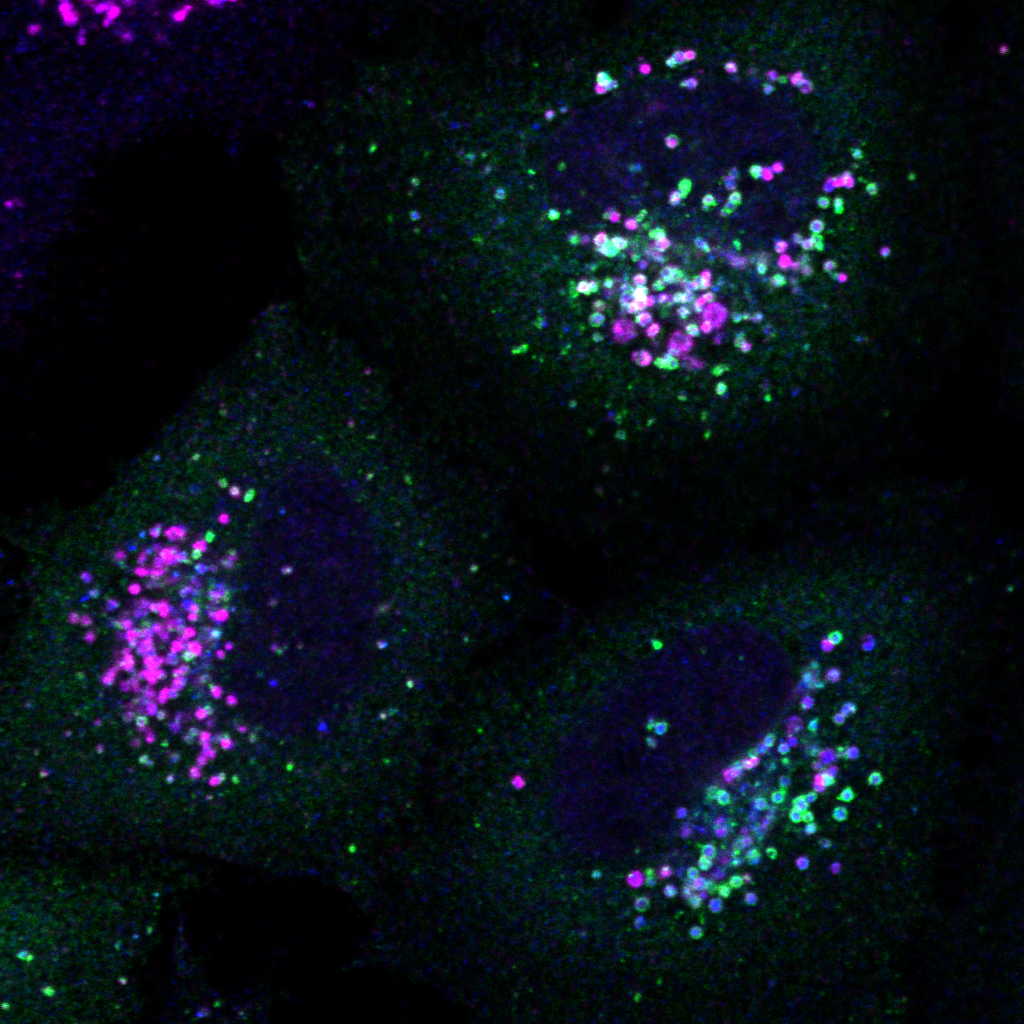

Supplement: Supplementary file 6 — Source Data for Figure 4 [file EMBR-24-e56841-s008.zip › Figure_4/4E/Image_Data/WT_LLOMe_merge.tif]

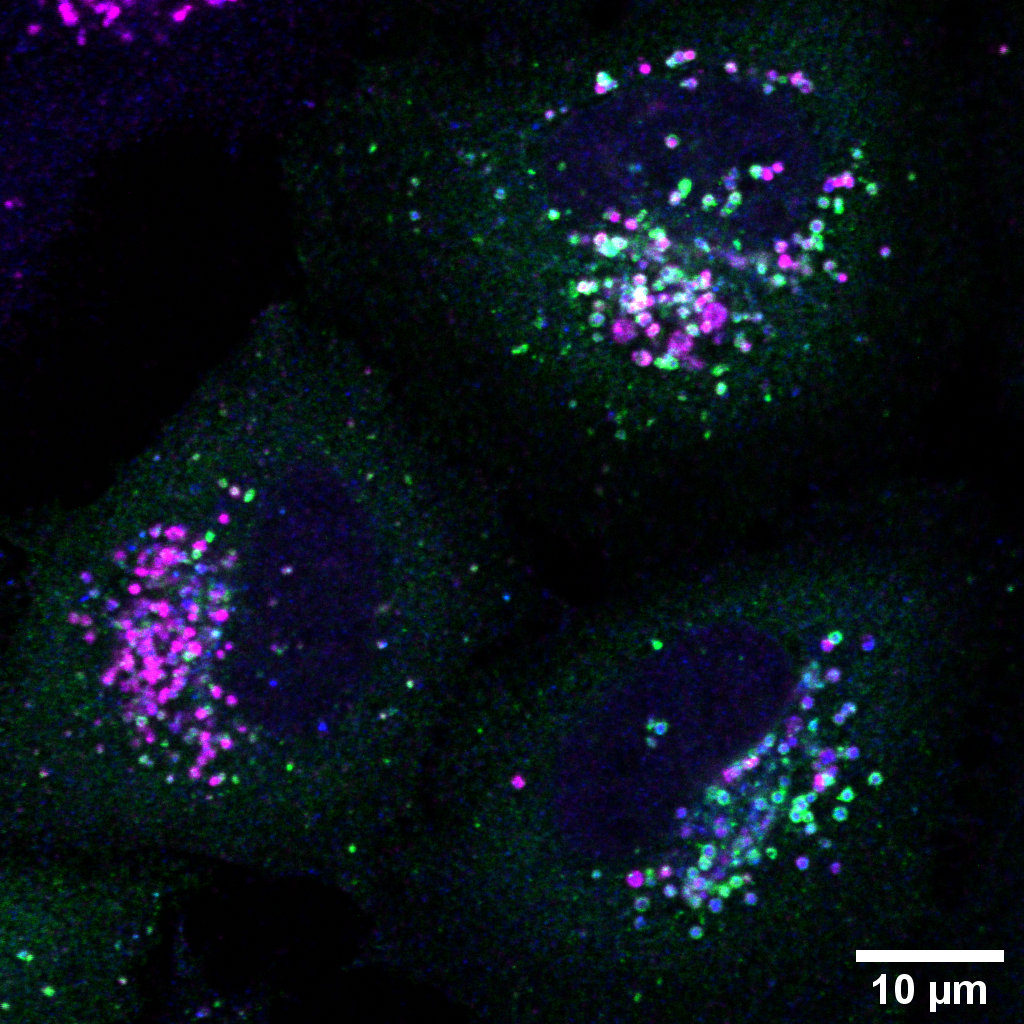

Supplement: Supplementary file 6 — Source Data for Figure 4 [file EMBR-24-e56841-s008.zip › Figure_4/4E/Image_Data/WT_LLOMe_scale.tif]

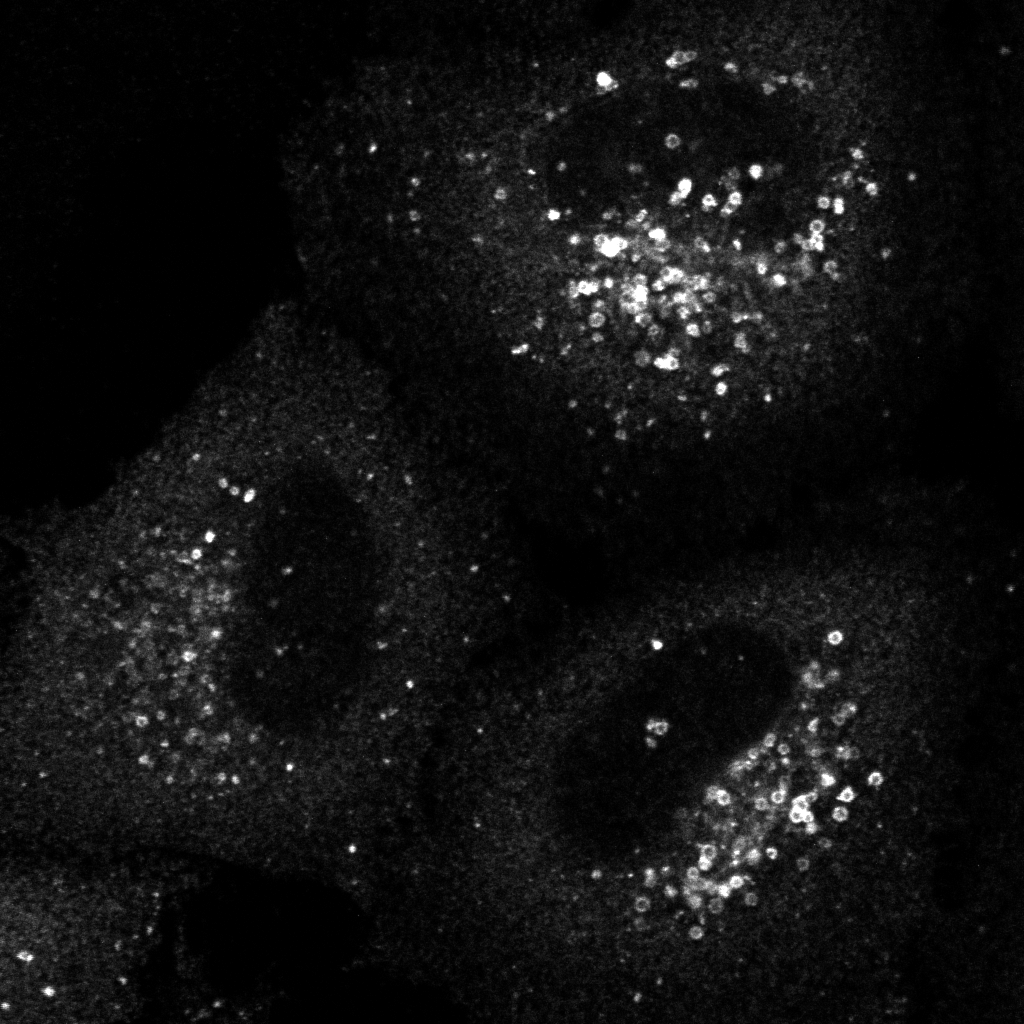

Supplement: Supplementary file 6 — Source Data for Figure 4 [file EMBR-24-e56841-s008.zip › Figure_4/4E/Image_Data/WT_LLOMe_TECPR1.tif]

# ATG16L1 KO

INPUT

LysolP

LLOMe:

-

+

-

+

LAMP1

130

100

Gal3

35

25

LC3B

15

10

Actin

55

35

← Gal3

kDa

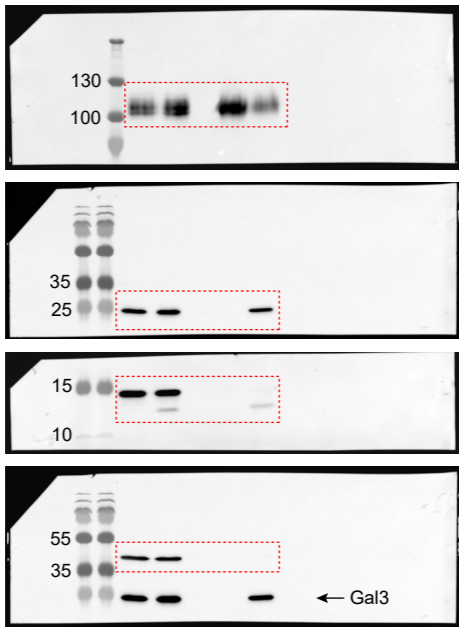

Supplement: Supplementary file 6 — Source Data for Figure 4 [file EMBR-24-e56841-s008.zip › Figure_4/4F/Image_data/4F.pdf]

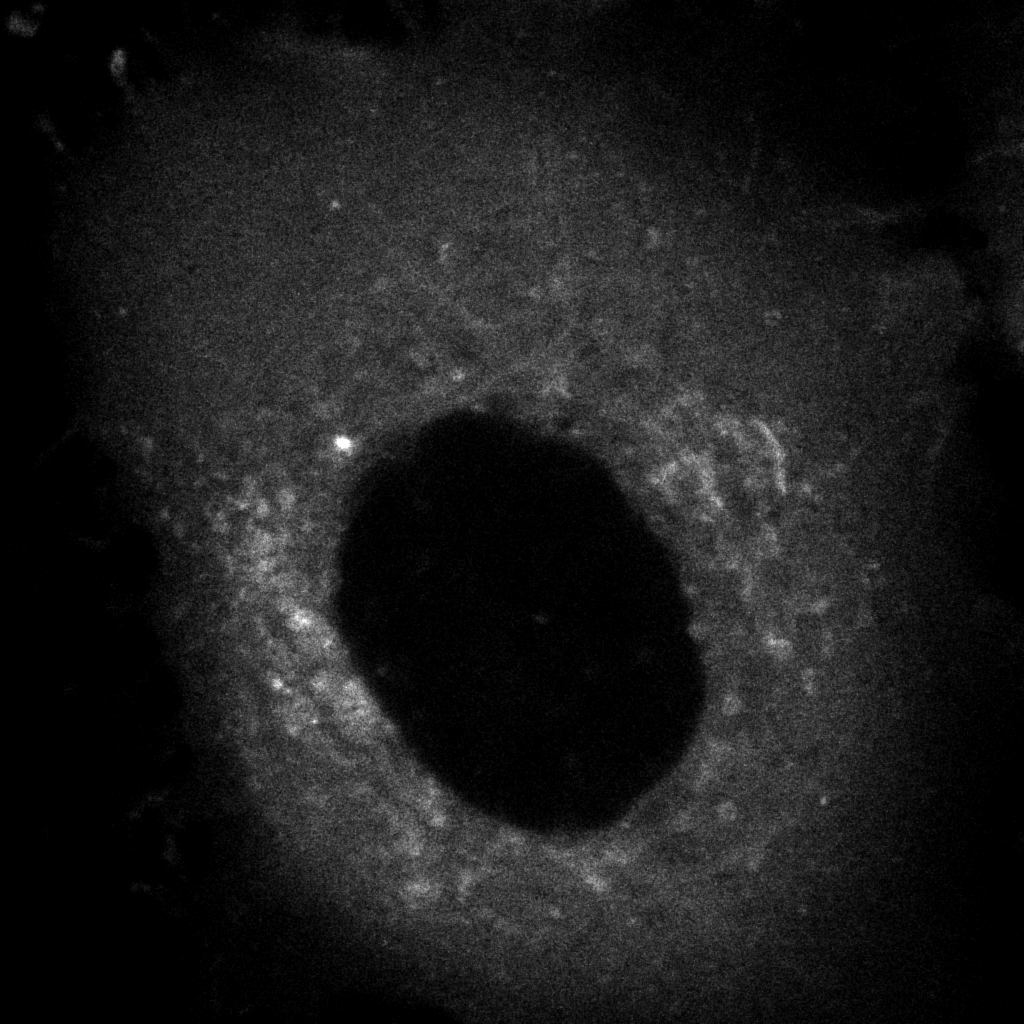

Supplement: Supplementary file 6 — Source Data for Figure 4 [file EMBR-24-e56841-s008.zip › Figure_4/4G/Image_Data/0min_ATG5.tif]

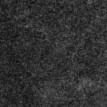

Supplement: Supplementary file 6 — Source Data for Figure 4 [file EMBR-24-e56841-s008.zip › Figure_4/4G/Image_Data/0min_ATG5_zoom.tif]

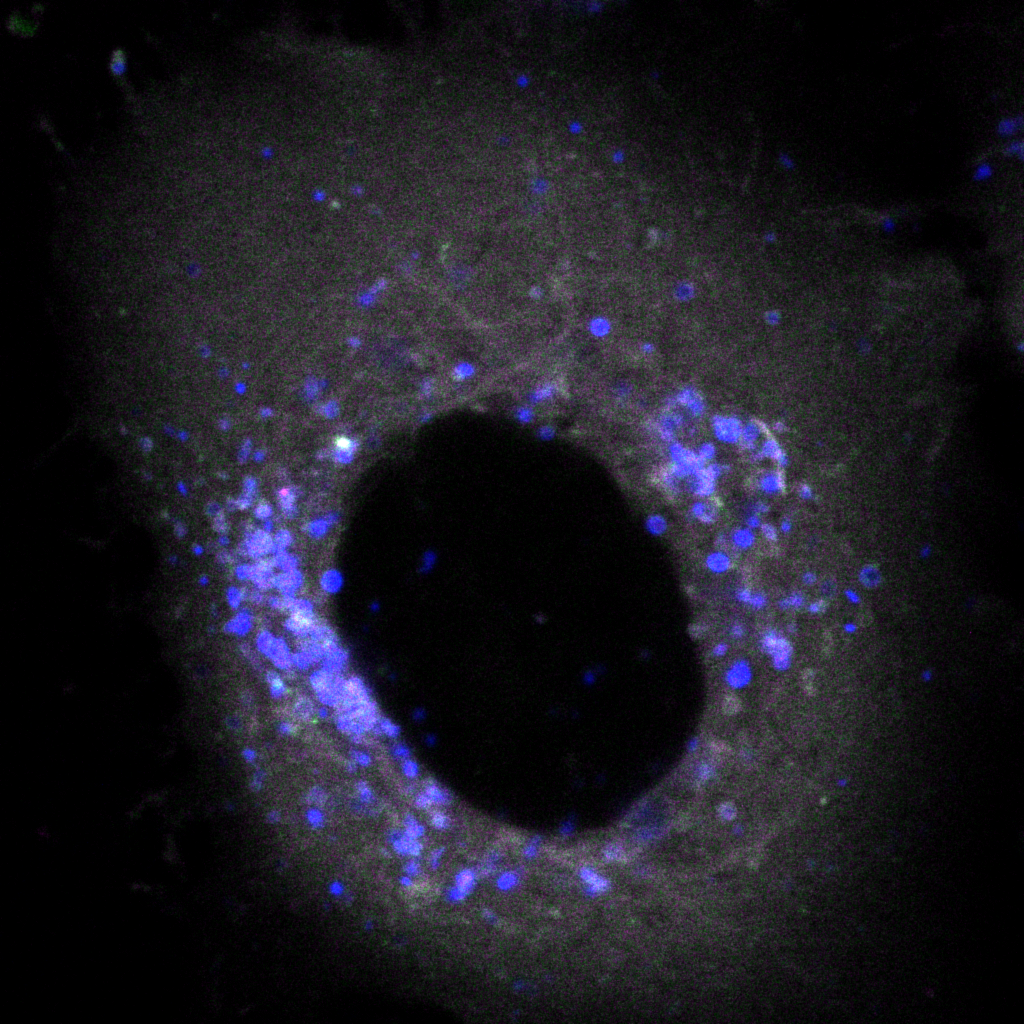

Supplement: Supplementary file 6 — Source Data for Figure 4 [file EMBR-24-e56841-s008.zip › Figure_4/4G/Image_Data/0min_merge.tif]

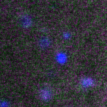

Supplement: Supplementary file 6 — Source Data for Figure 4 [file EMBR-24-e56841-s008.zip › Figure_4/4G/Image_Data/0min_merge_zoom.tif]

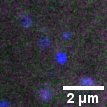

Supplement: Supplementary file 6 — Source Data for Figure 4 [file EMBR-24-e56841-s008.zip › Figure_4/4G/Image_Data/0min_scale_zoom.tif]

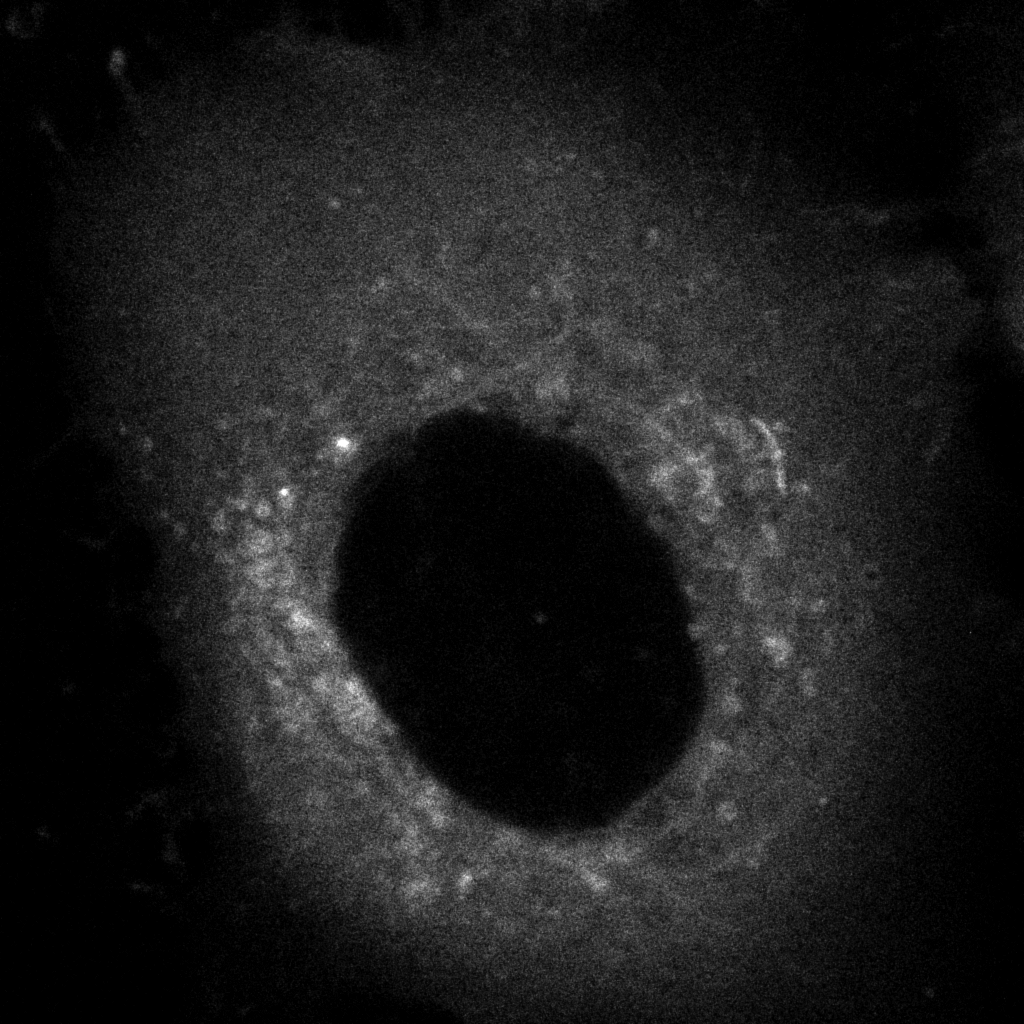

Supplement: Supplementary file 6 — Source Data for Figure 4 [file EMBR-24-e56841-s008.zip › Figure_4/4G/Image_Data/0min_TECPR1.tif]

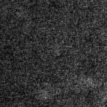

Supplement: Supplementary file 6 — Source Data for Figure 4 [file EMBR-24-e56841-s008.zip › Figure_4/4G/Image_Data/0min_TECPR1_zoom.tif]

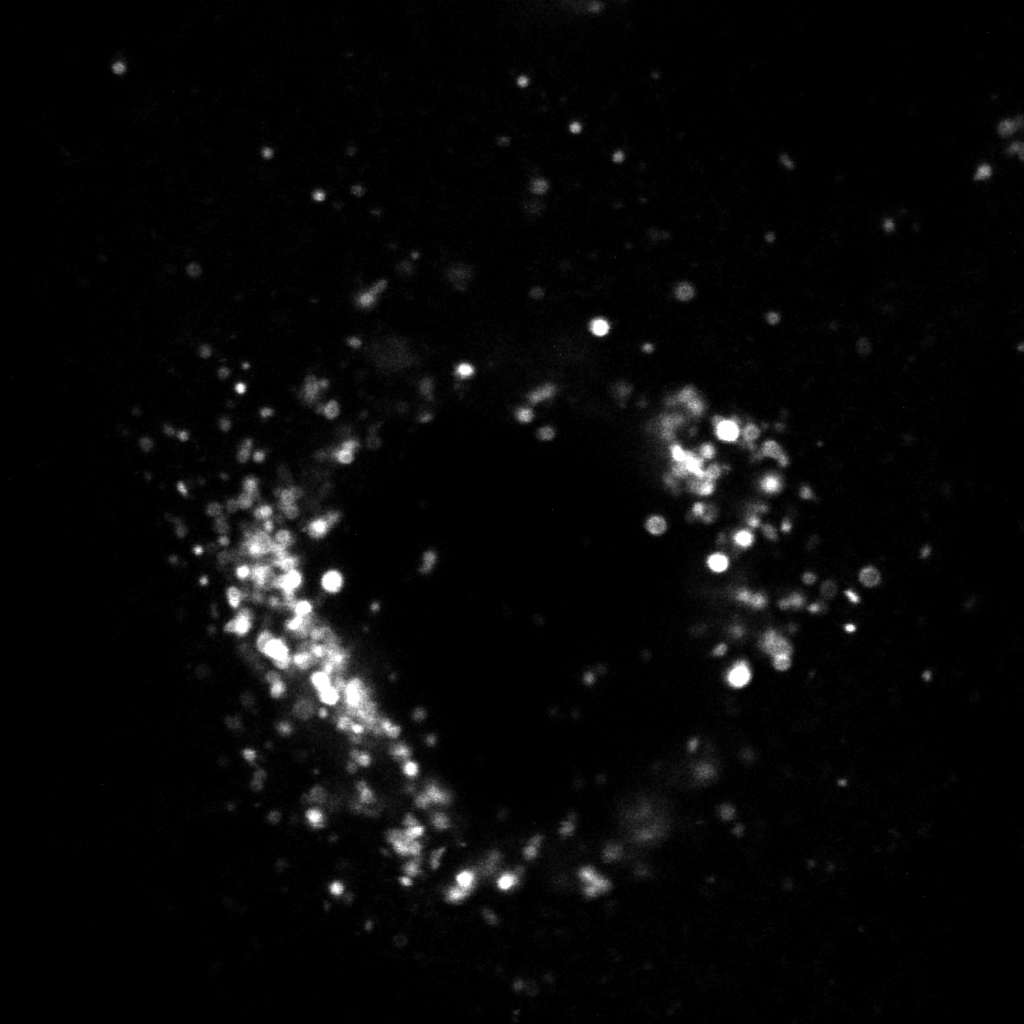

Supplement: Supplementary file 6 — Source Data for Figure 4 [file EMBR-24-e56841-s008.zip › Figure_4/4G/Image_Data/0min_TMEM.tif]

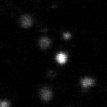

Supplement: Supplementary file 6 — Source Data for Figure 4 [file EMBR-24-e56841-s008.zip › Figure_4/4G/Image_Data/0min_TMEM_zoom.tif]

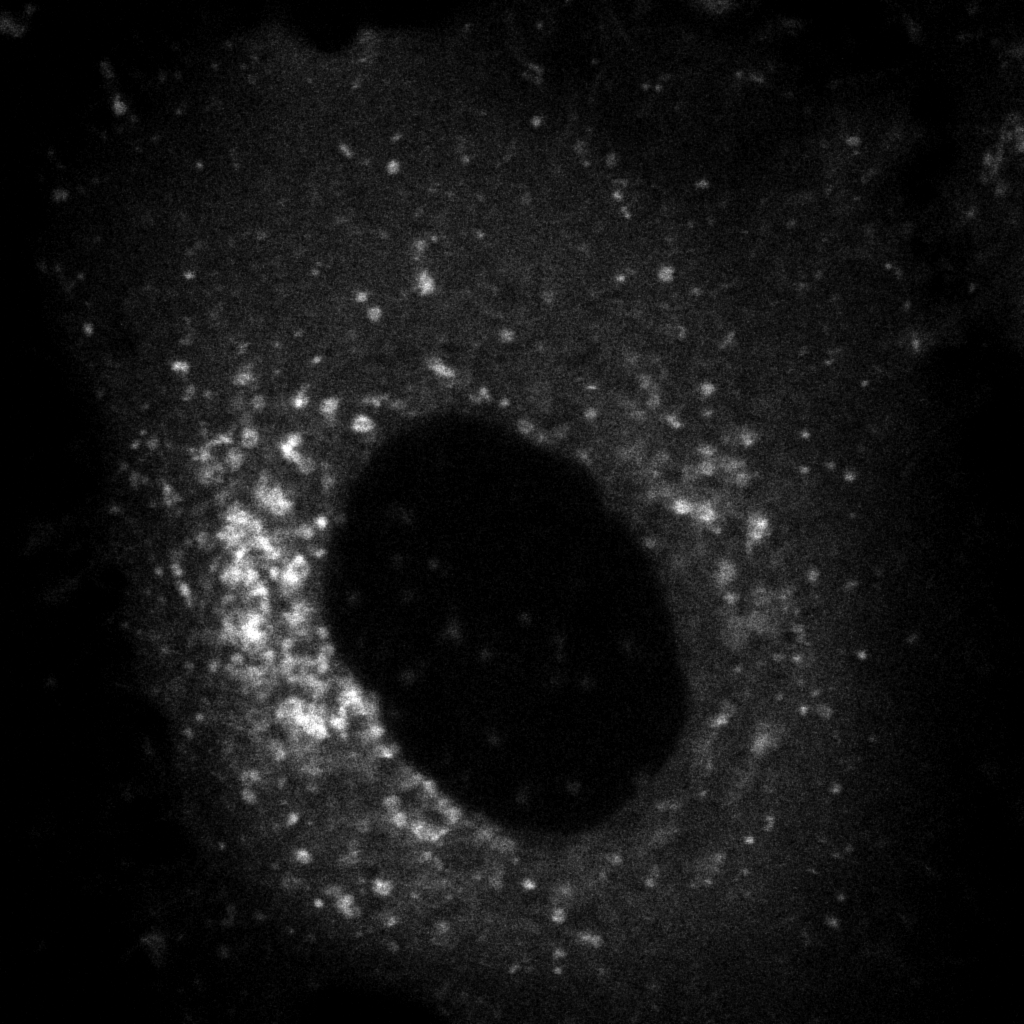

Supplement: Supplementary file 6 — Source Data for Figure 4 [file EMBR-24-e56841-s008.zip › Figure_4/4G/Image_Data/15min_ATG5.tif]

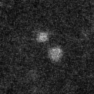

Supplement: Supplementary file 6 — Source Data for Figure 4 [file EMBR-24-e56841-s008.zip › Figure_4/4G/Image_Data/15min_ATG5_zoom.tif]

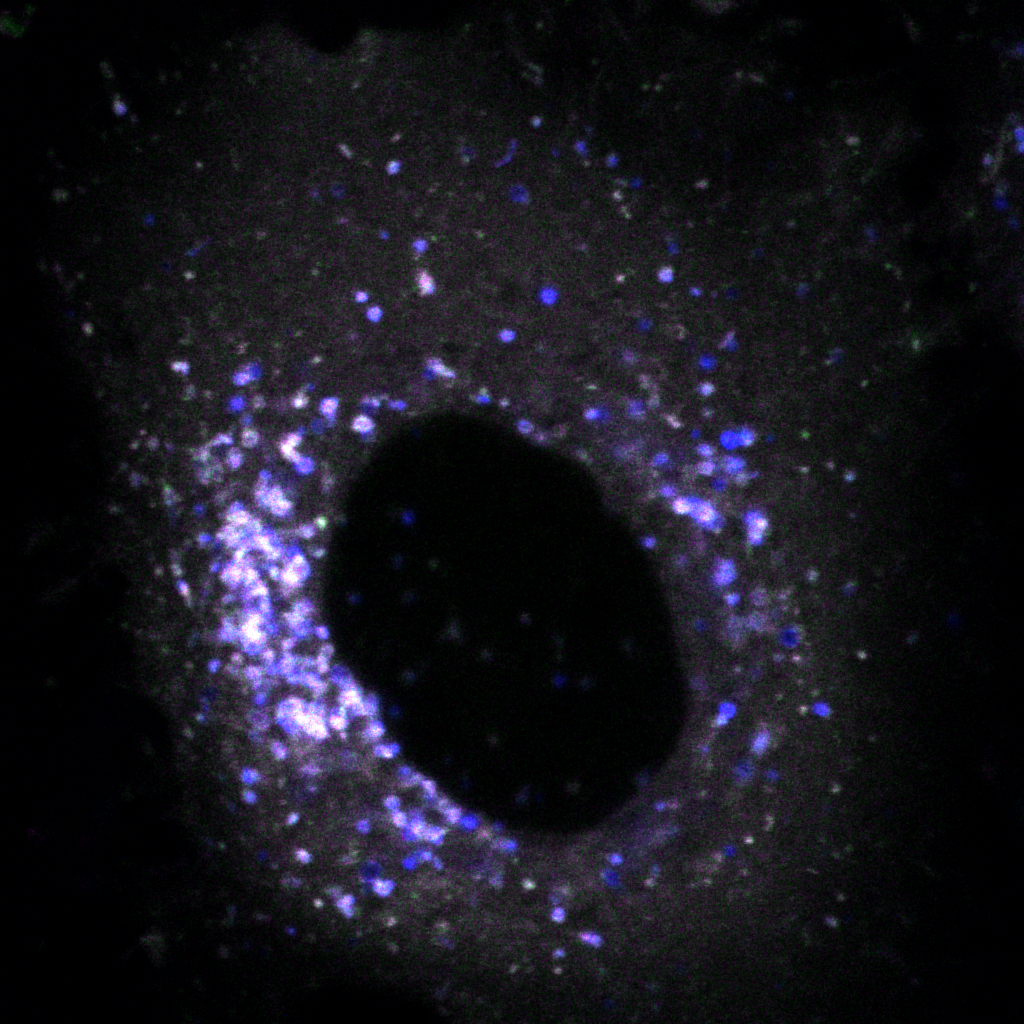

Supplement: Supplementary file 6 — Source Data for Figure 4 [file EMBR-24-e56841-s008.zip › Figure_4/4G/Image_Data/15min_merge.tif]

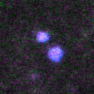

Supplement: Supplementary file 6 — Source Data for Figure 4 [file EMBR-24-e56841-s008.zip › Figure_4/4G/Image_Data/15min_merge_zoom.tif]

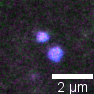

Supplement: Supplementary file 6 — Source Data for Figure 4 [file EMBR-24-e56841-s008.zip › Figure_4/4G/Image_Data/15min_scale_zoom.tif]

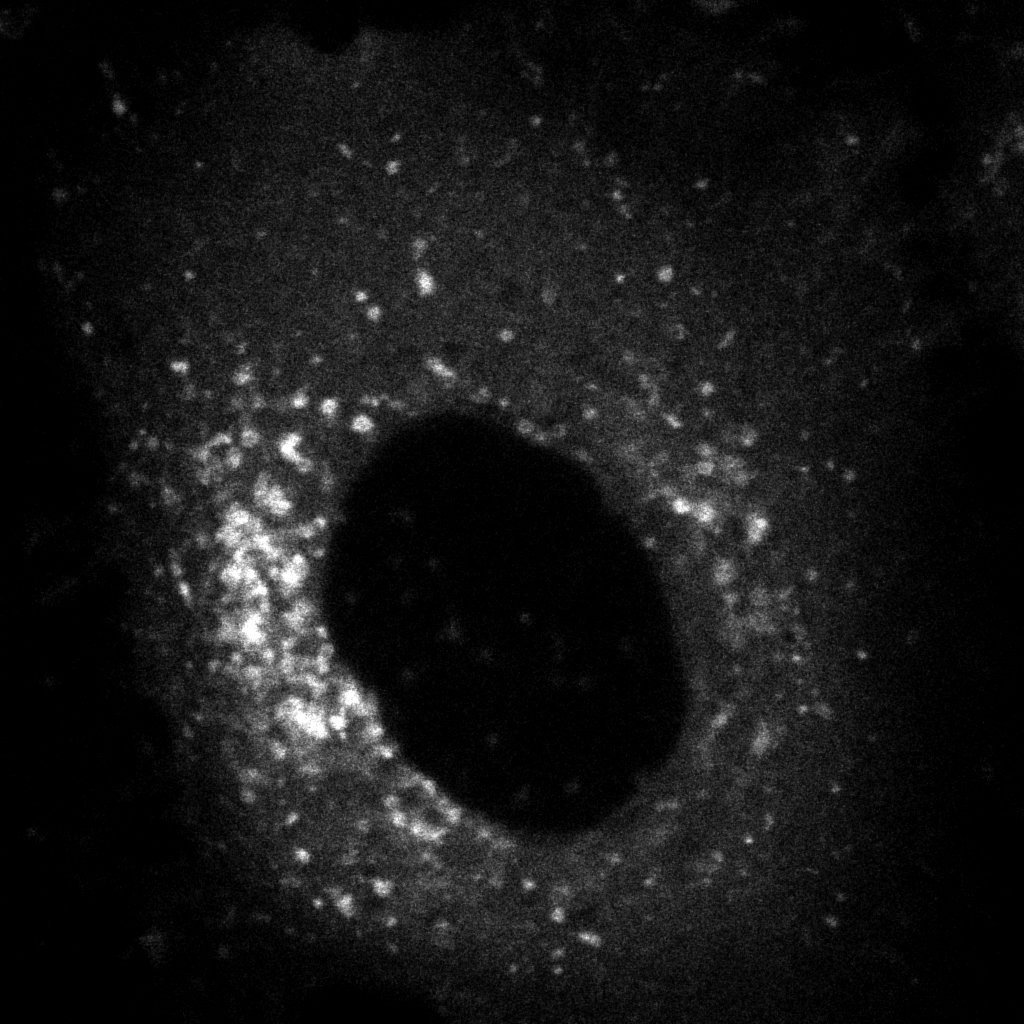

Supplement: Supplementary file 6 — Source Data for Figure 4 [file EMBR-24-e56841-s008.zip › Figure_4/4G/Image_Data/15min_TECPR1.tif]
