# Supplementary figures and images for: An ATG12‐ATG5‐TECPR1 E3‐like complex regulates unconventional LC3 lipidation at damaged lysosomes (part 4 of 4)
Source: EMBO Rep. 2023 Jun 29;24(9):e56841. doi: 10.15252/embr.202356841 (PMC10481663; doi:10.15252/embr.202356841)

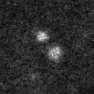

Supplement: Supplementary file 6 — Source Data for Figure 4 [file EMBR-24-e56841-s008.zip › Figure_4/4G/Image_Data/15min_TECPR1_zoom.tif]

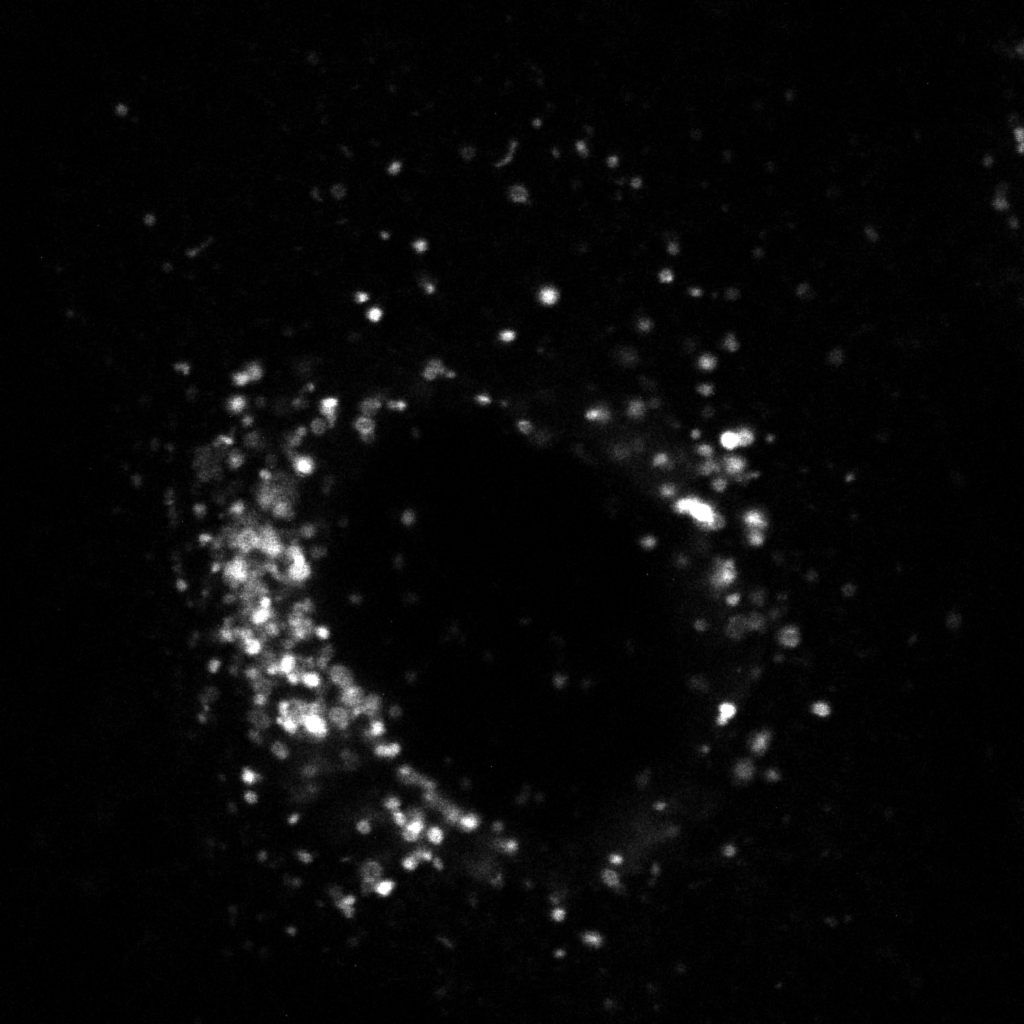

Supplement: Supplementary file 6 — Source Data for Figure 4 [file EMBR-24-e56841-s008.zip › Figure_4/4G/Image_Data/15min_TMEM.tif]

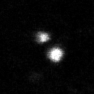

Supplement: Supplementary file 6 — Source Data for Figure 4 [file EMBR-24-e56841-s008.zip › Figure_4/4G/Image_Data/15min_TMEM_zoom.tif]

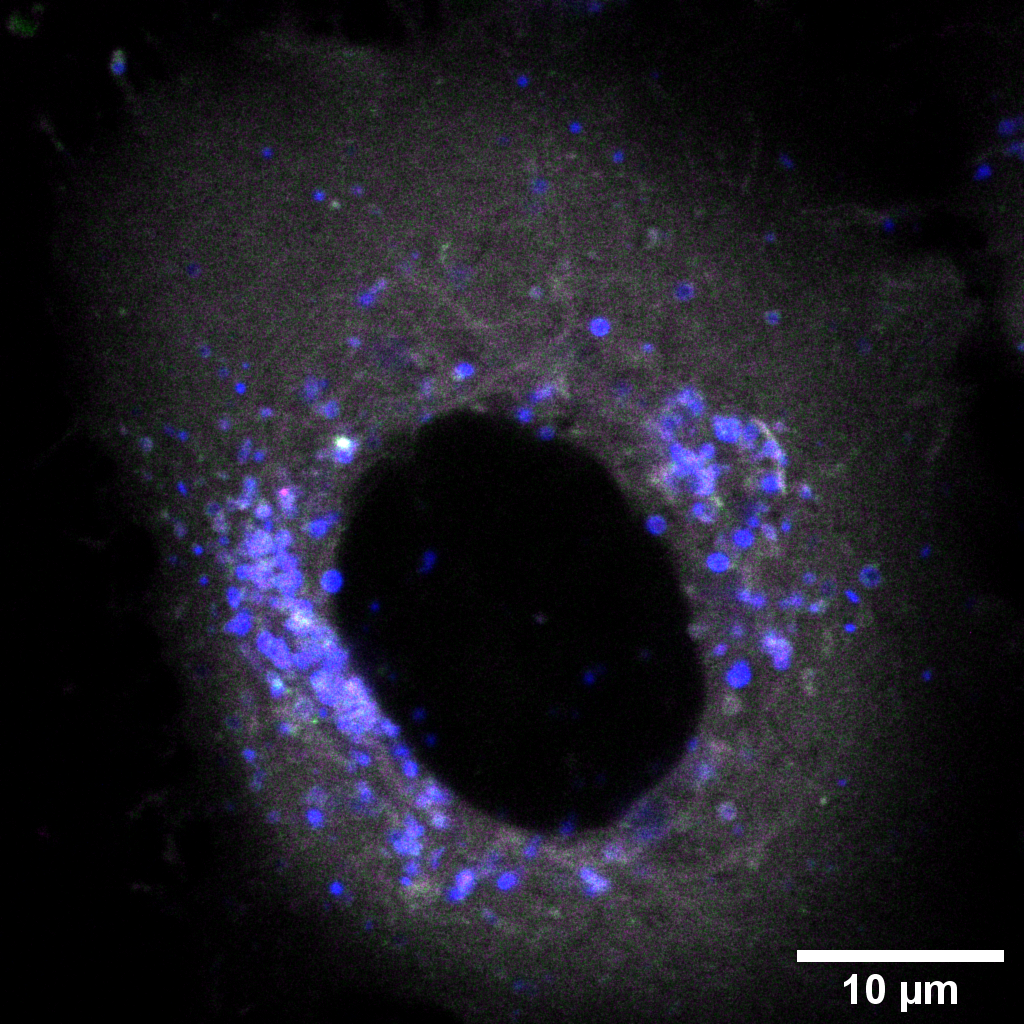

Supplement: Supplementary file 6 — Source Data for Figure 4 [file EMBR-24-e56841-s008.zip › Figure_4/4G/Image_Data/scale.tif]

|         | HEK WT |   |      |   |      |   | HEK ATG16L1 KO |   |      |   |      |   |
|---------|--------|---|------|---|------|---|----------------|---|------|---|------|---|
| TECPR1: | P      |   | KO#1 |   | KO#2 |   | P              |   | KO#1 |   | KO#2 |   |
| LLOMe:  | -      | + | -    | + | -    | + | -              | + | -    | + | -    | + |

LC3B

15  
10

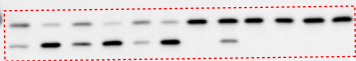

TECPR1

250  
130

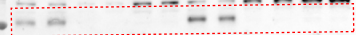

ATG16L1

70  
55

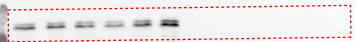

Actin

55  
35

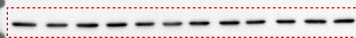

kDa

Supplement: Supplementary file 6 — Source Data for Figure 4 [file EMBR-24-e56841-s008.zip › Figure_4/4H/Image_data/4H.pdf]

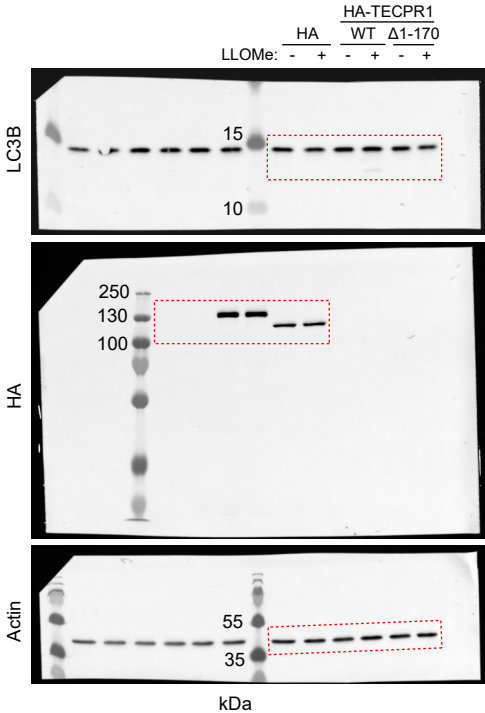

Supplement: Supplementary file 6 — Source Data for Figure 4 [file EMBR-24-e56841-s008.zip › Figure_4/4I/4I.pdf]

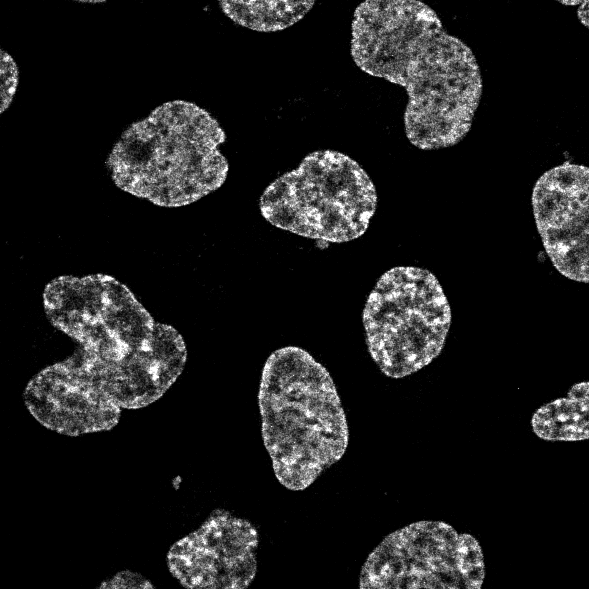

Supplement: Supplementary file 7 — Source Data for Figure 5 [file EMBR-24-e56841-s003.zip › Figure_5/5A/16KO_0.5h_Hoechst.tif]

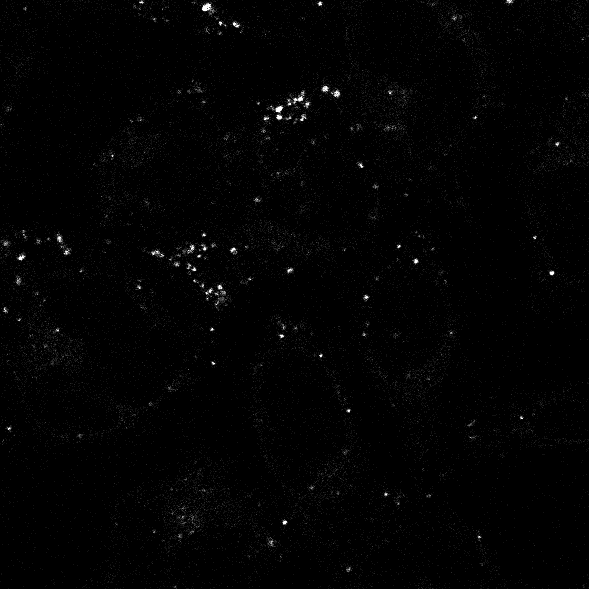

Supplement: Supplementary file 7 — Source Data for Figure 5 [file EMBR-24-e56841-s003.zip › Figure_5/5A/16KO_0.5h_LT.tif]

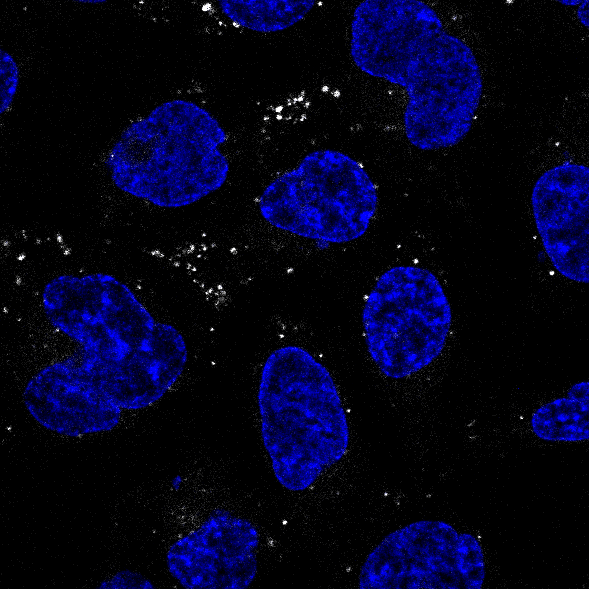

Supplement: Supplementary file 7 — Source Data for Figure 5 [file EMBR-24-e56841-s003.zip › Figure_5/5A/16KO_0.5h_merge.tif]

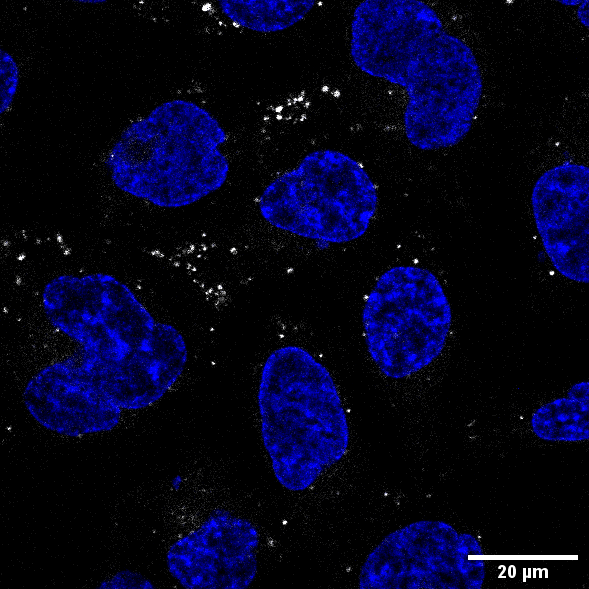

Supplement: Supplementary file 7 — Source Data for Figure 5 [file EMBR-24-e56841-s003.zip › Figure_5/5A/16KO_0.5h_scale.tif]

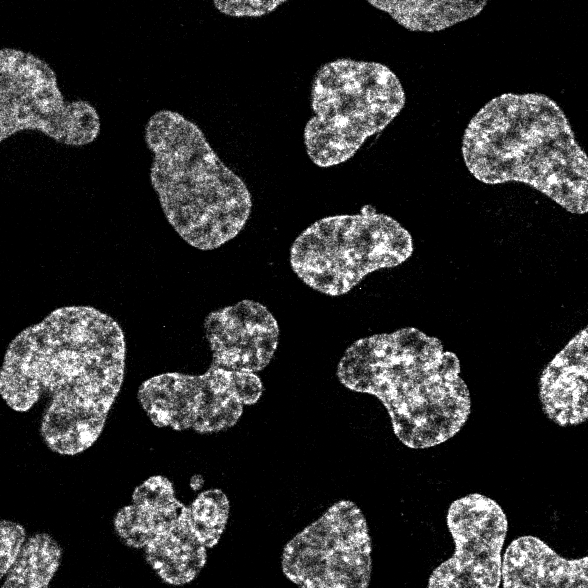

Supplement: Supplementary file 7 — Source Data for Figure 5 [file EMBR-24-e56841-s003.zip › Figure_5/5A/16KO_0h_Hoechst.tif]

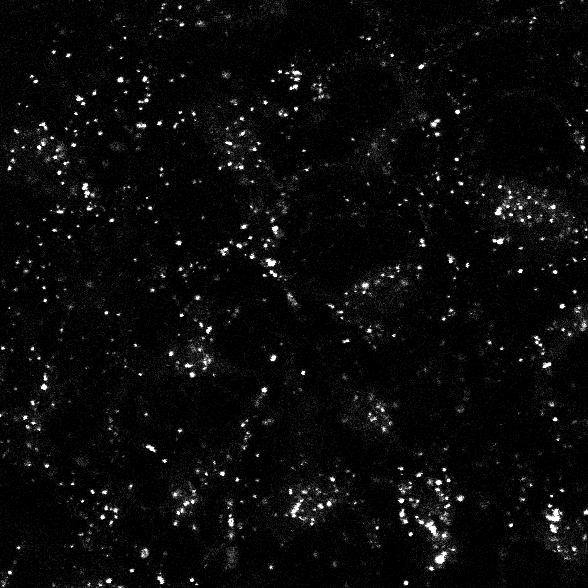

Supplement: Supplementary file 7 — Source Data for Figure 5 [file EMBR-24-e56841-s003.zip › Figure_5/5A/16KO_0h_LT.tif]

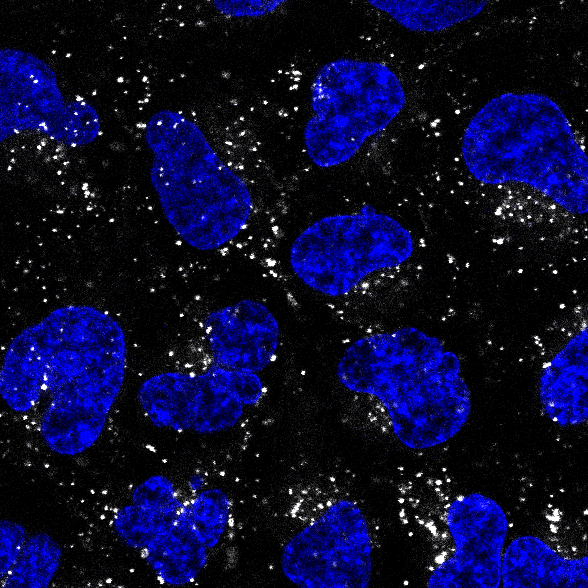

Supplement: Supplementary file 7 — Source Data for Figure 5 [file EMBR-24-e56841-s003.zip › Figure_5/5A/16KO_0h_merge.tif]

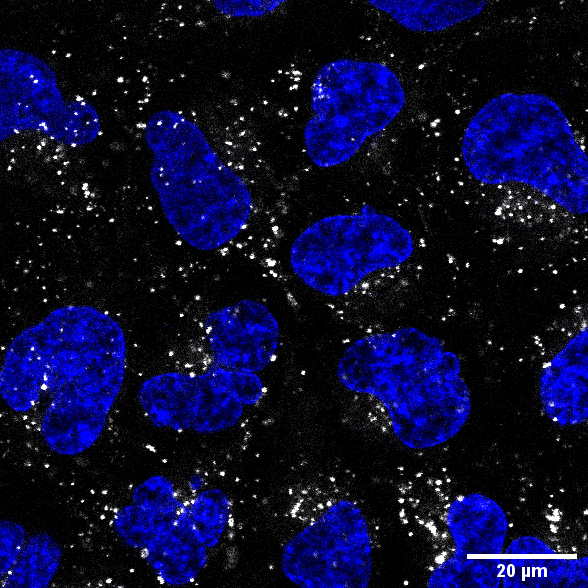

Supplement: Supplementary file 7 — Source Data for Figure 5 [file EMBR-24-e56841-s003.zip › Figure_5/5A/16KO_0h_scale.tif]

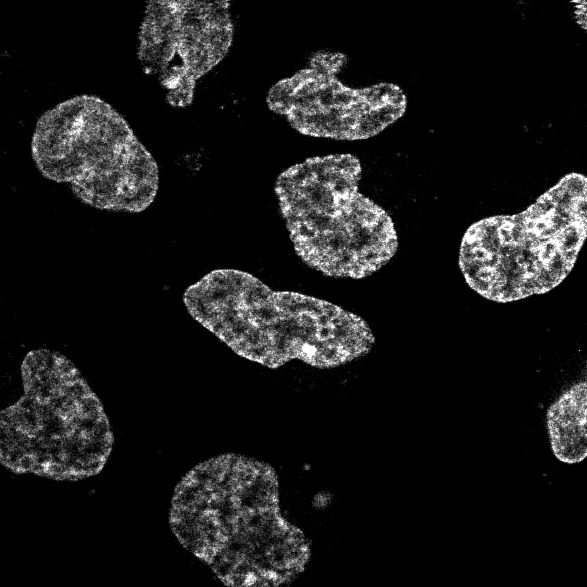

Supplement: Supplementary file 7 — Source Data for Figure 5 [file EMBR-24-e56841-s003.zip › Figure_5/5A/16KO_1h_Hoechst.tif]

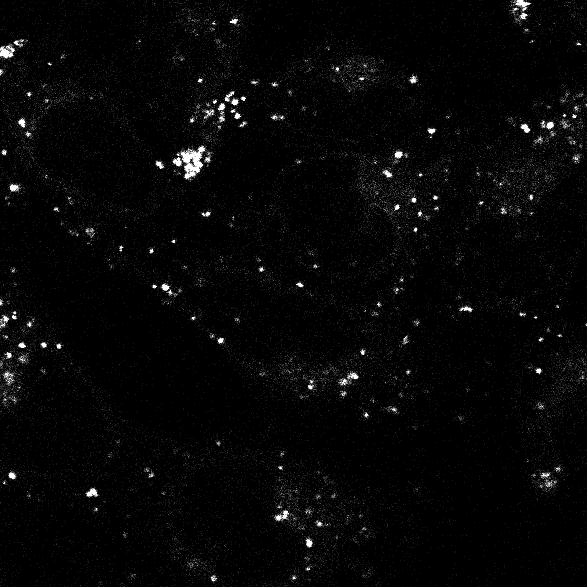

Supplement: Supplementary file 7 — Source Data for Figure 5 [file EMBR-24-e56841-s003.zip › Figure_5/5A/16KO_1h_LT.tif]

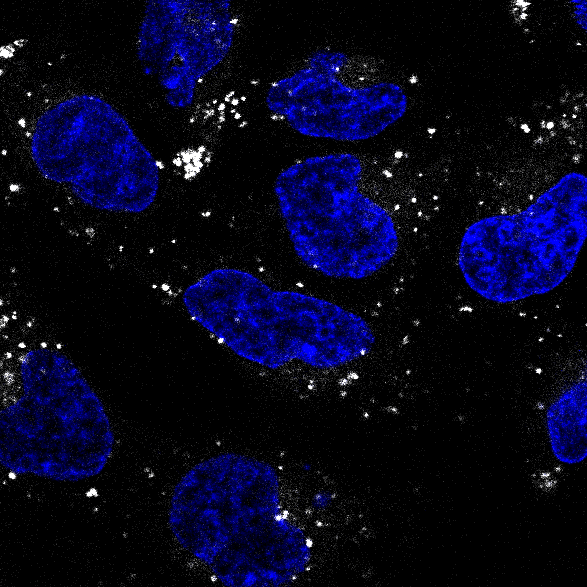

Supplement: Supplementary file 7 — Source Data for Figure 5 [file EMBR-24-e56841-s003.zip › Figure_5/5A/16KO_1h_merge.tif]

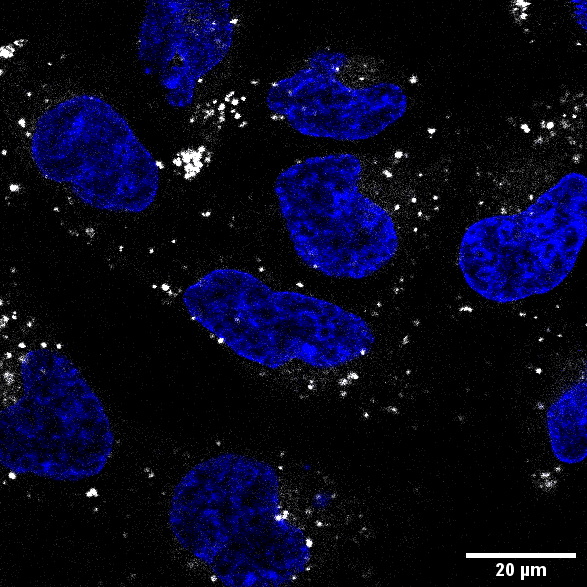

Supplement: Supplementary file 7 — Source Data for Figure 5 [file EMBR-24-e56841-s003.zip › Figure_5/5A/16KO_1h_scale.tif]

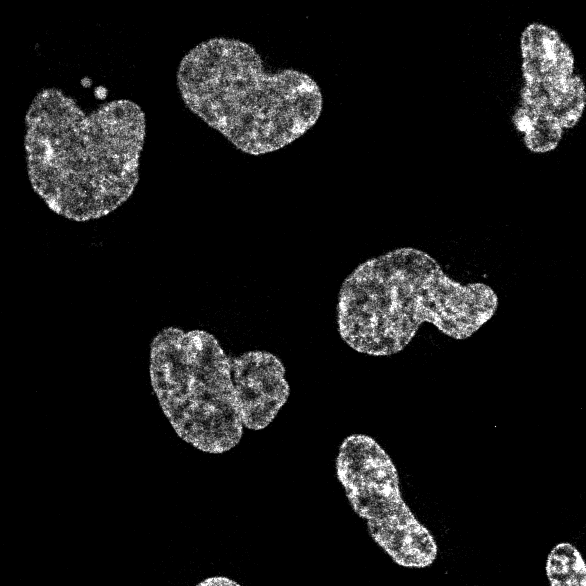

Supplement: Supplementary file 7 — Source Data for Figure 5 [file EMBR-24-e56841-s003.zip › Figure_5/5A/16KO_LLOMe_Hoechst.tif]

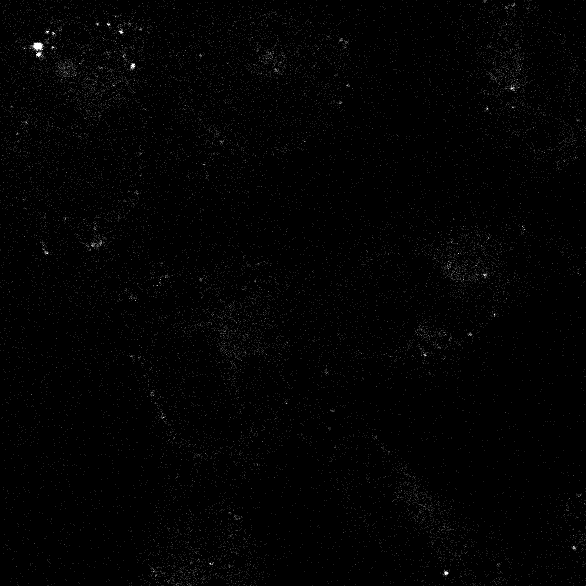

Supplement: Supplementary file 7 — Source Data for Figure 5 [file EMBR-24-e56841-s003.zip › Figure_5/5A/16KO_LLOMe_LT.tif]

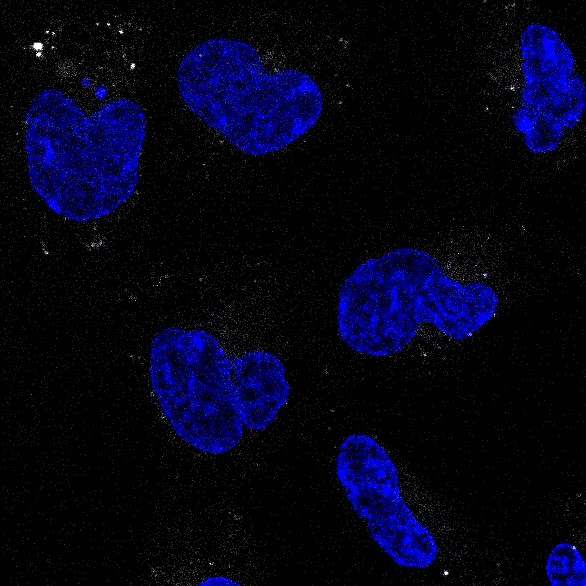

Supplement: Supplementary file 7 — Source Data for Figure 5 [file EMBR-24-e56841-s003.zip › Figure_5/5A/16KO_LLOMe_merge.tif]

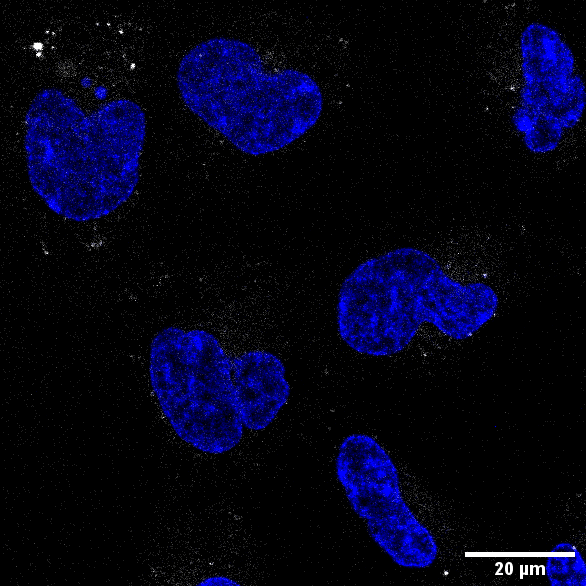

Supplement: Supplementary file 7 — Source Data for Figure 5 [file EMBR-24-e56841-s003.zip › Figure_5/5A/16KO_LLOMe_scale.tif]

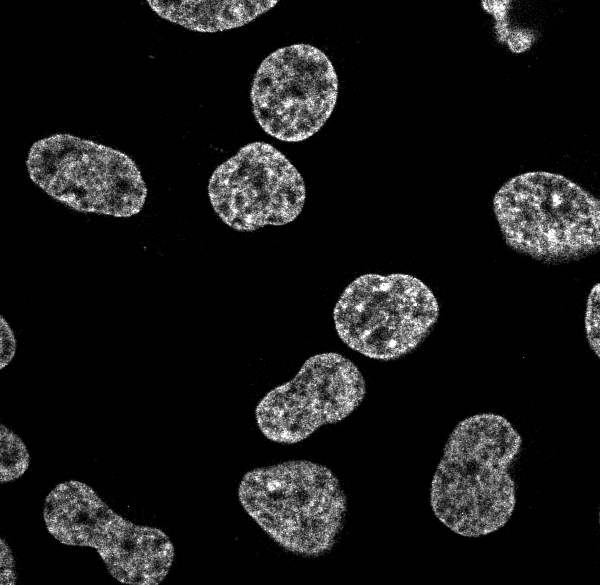

Supplement: Supplementary file 7 — Source Data for Figure 5 [file EMBR-24-e56841-s003.zip › Figure_5/5A/TECKO_0.5h_Hoechst.tif]

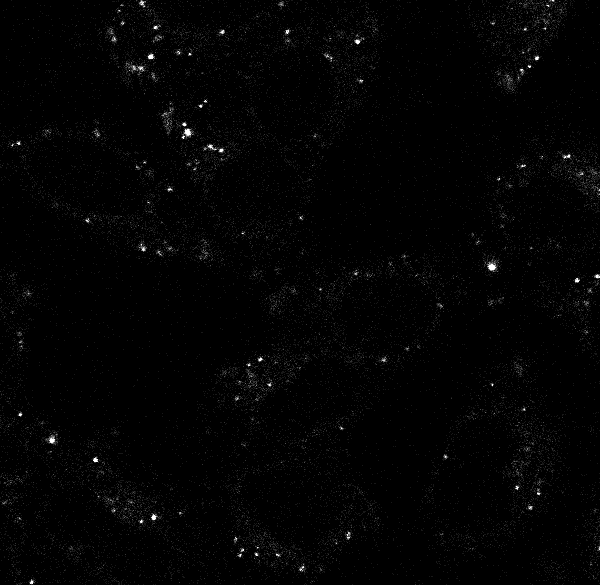

Supplement: Supplementary file 7 — Source Data for Figure 5 [file EMBR-24-e56841-s003.zip › Figure_5/5A/TECKO_0.5h_LT.tif]

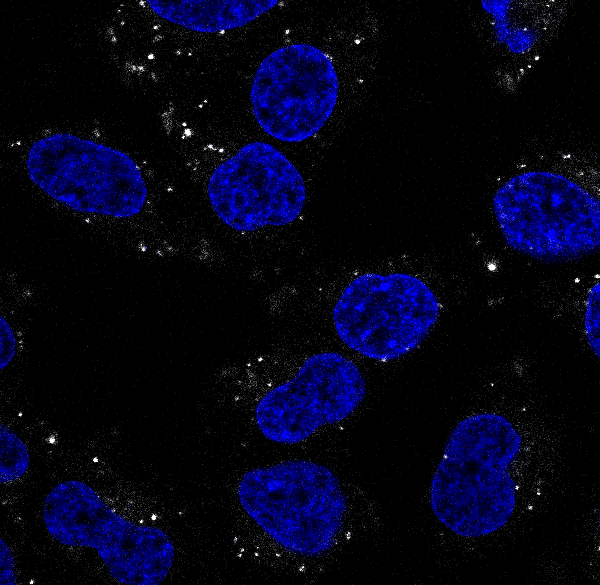

Supplement: Supplementary file 7 — Source Data for Figure 5 [file EMBR-24-e56841-s003.zip › Figure_5/5A/TECKO_0.5h_merge.tif]

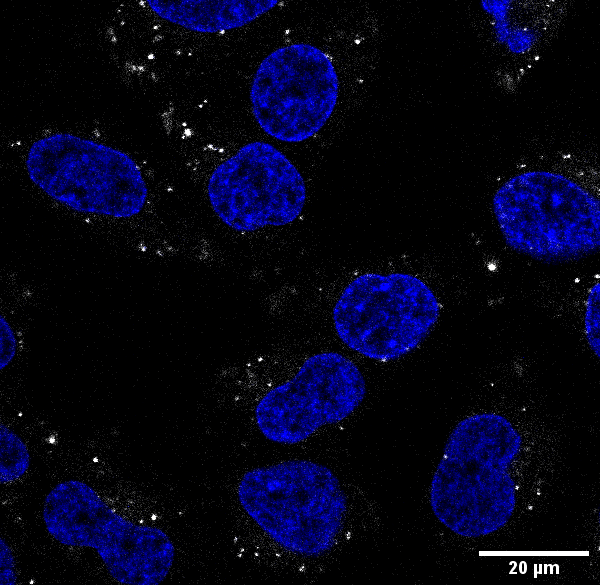

Supplement: Supplementary file 7 — Source Data for Figure 5 [file EMBR-24-e56841-s003.zip › Figure_5/5A/TECKO_0.5h_scale.tif]

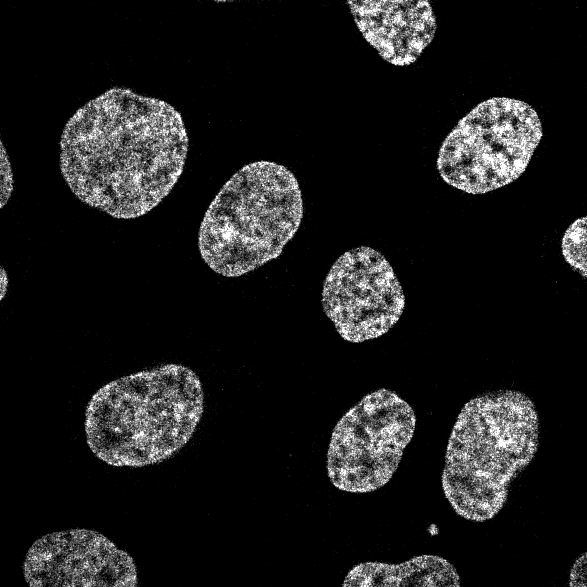

Supplement: Supplementary file 7 — Source Data for Figure 5 [file EMBR-24-e56841-s003.zip › Figure_5/5A/TECKO_0h_Hoechst.tif]

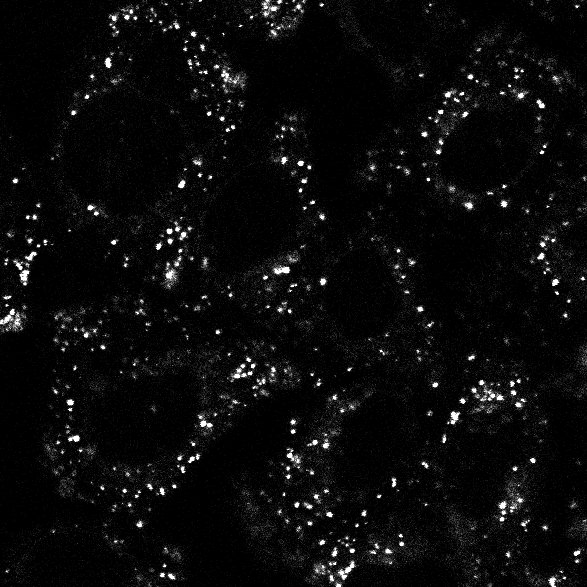

Supplement: Supplementary file 7 — Source Data for Figure 5 [file EMBR-24-e56841-s003.zip › Figure_5/5A/TECKO_0h_LT.tif]

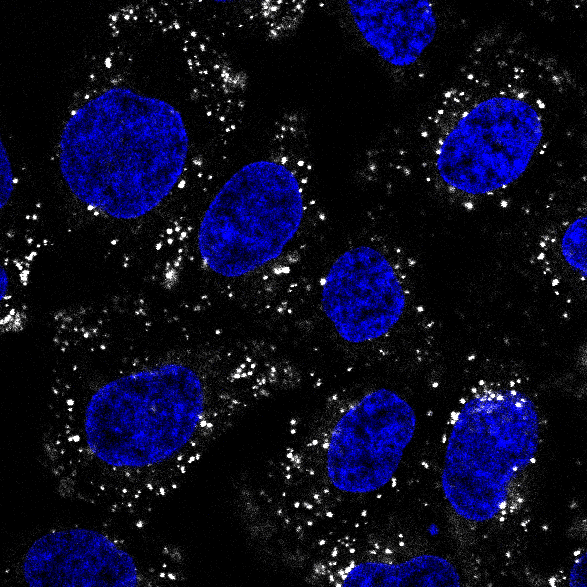

Supplement: Supplementary file 7 — Source Data for Figure 5 [file EMBR-24-e56841-s003.zip › Figure_5/5A/TECKO_0h_merge.tif]

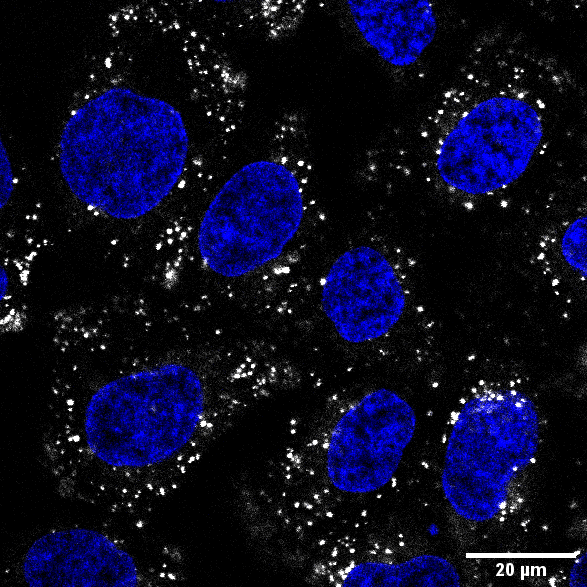

Supplement: Supplementary file 7 — Source Data for Figure 5 [file EMBR-24-e56841-s003.zip › Figure_5/5A/TECKO_0h_scale.tif]

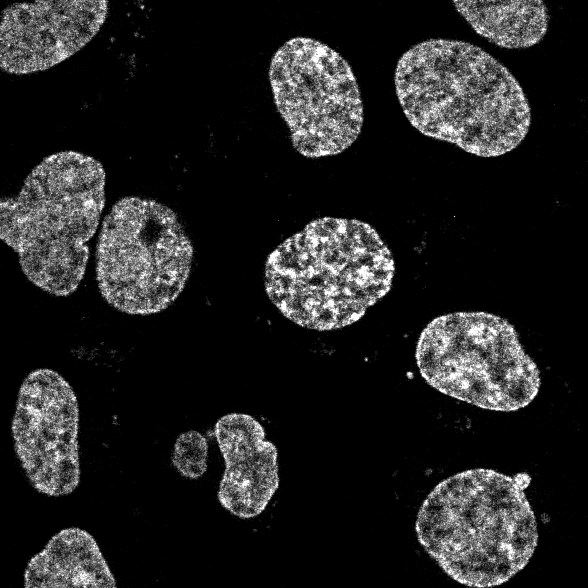

Supplement: Supplementary file 7 — Source Data for Figure 5 [file EMBR-24-e56841-s003.zip › Figure_5/5A/TECKO_1h_Hoechst.tif]

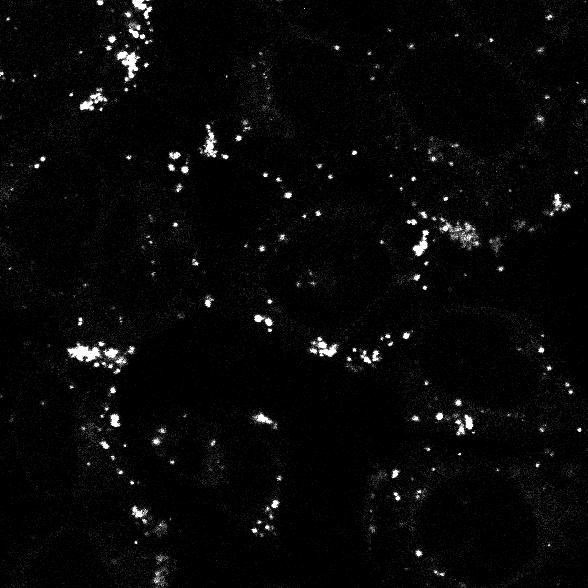

Supplement: Supplementary file 7 — Source Data for Figure 5 [file EMBR-24-e56841-s003.zip › Figure_5/5A/TECKO_1h_LT.tif]

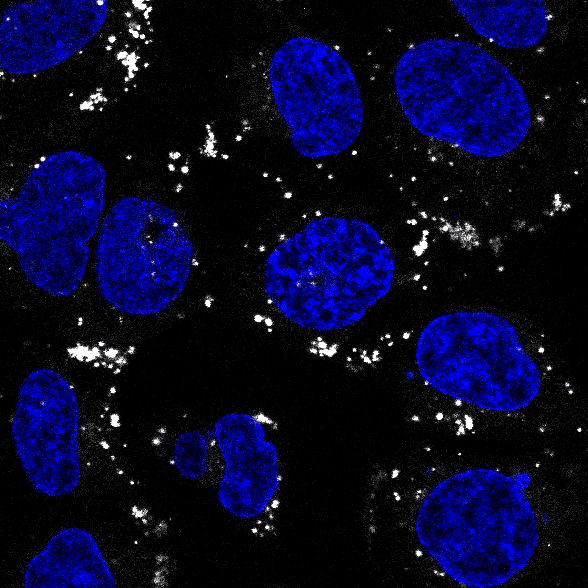

Supplement: Supplementary file 7 — Source Data for Figure 5 [file EMBR-24-e56841-s003.zip › Figure_5/5A/TECKO_1h_merge.tif]

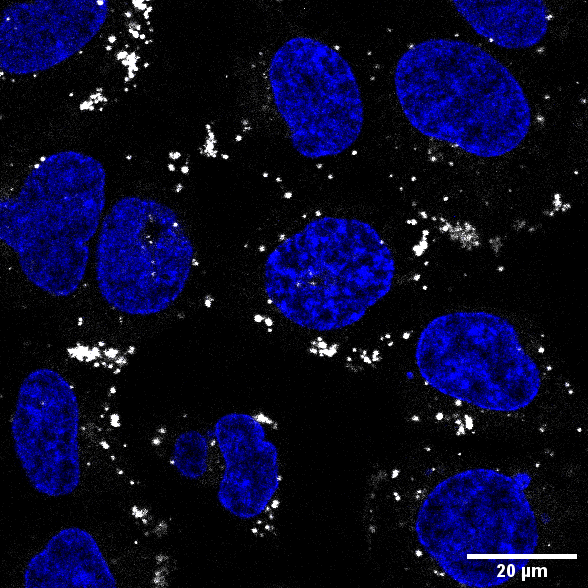

Supplement: Supplementary file 7 — Source Data for Figure 5 [file EMBR-24-e56841-s003.zip › Figure_5/5A/TECKO_1h_scale.tif]

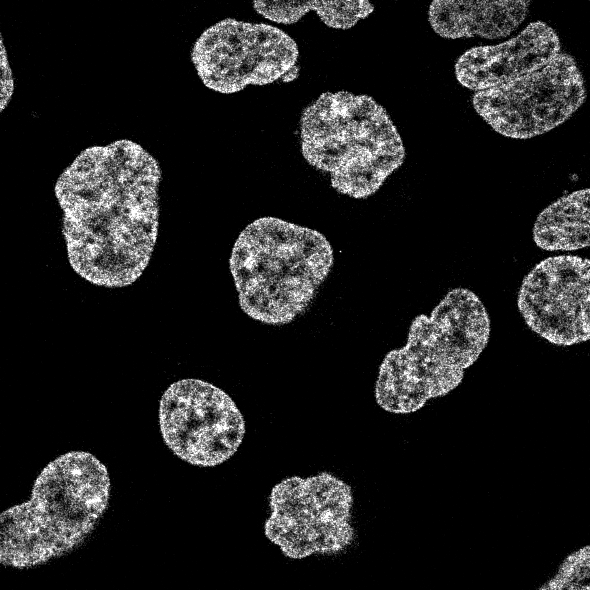

Supplement: Supplementary file 7 — Source Data for Figure 5 [file EMBR-24-e56841-s003.zip › Figure_5/5A/TECKO_LLOMe_Hoechst.tif]

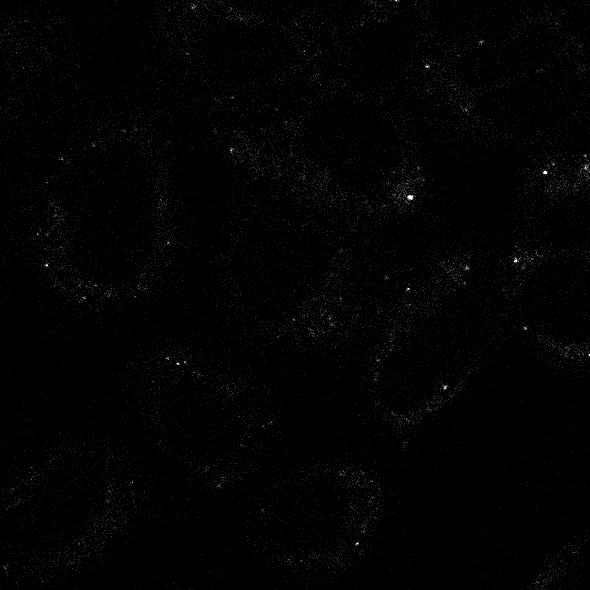

Supplement: Supplementary file 7 — Source Data for Figure 5 [file EMBR-24-e56841-s003.zip › Figure_5/5A/TECKO_LLOMe_LT.tif]

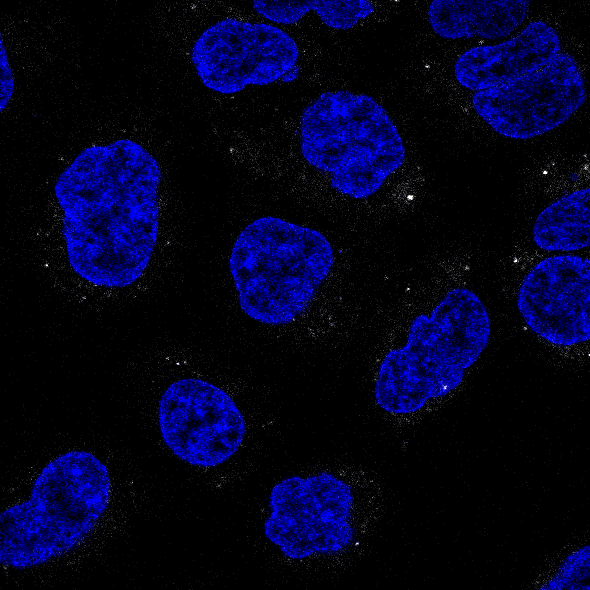

Supplement: Supplementary file 7 — Source Data for Figure 5 [file EMBR-24-e56841-s003.zip › Figure_5/5A/TECKO_LLOMe_merge.tif]

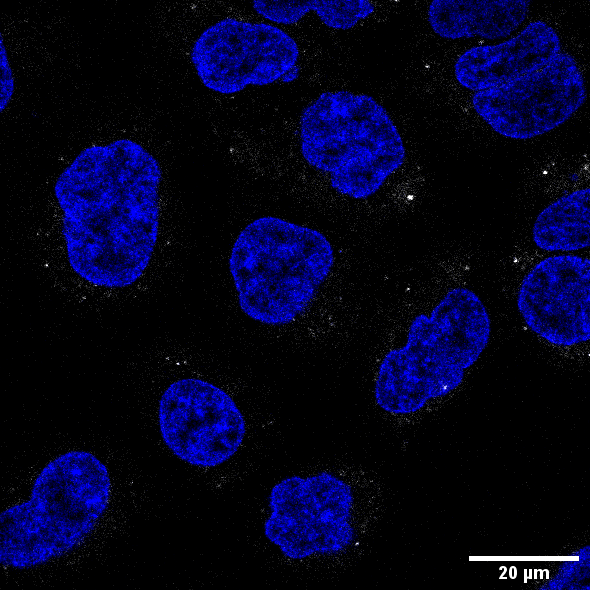

Supplement: Supplementary file 7 — Source Data for Figure 5 [file EMBR-24-e56841-s003.zip › Figure_5/5A/TECKO_LLOMe_scale.tif]

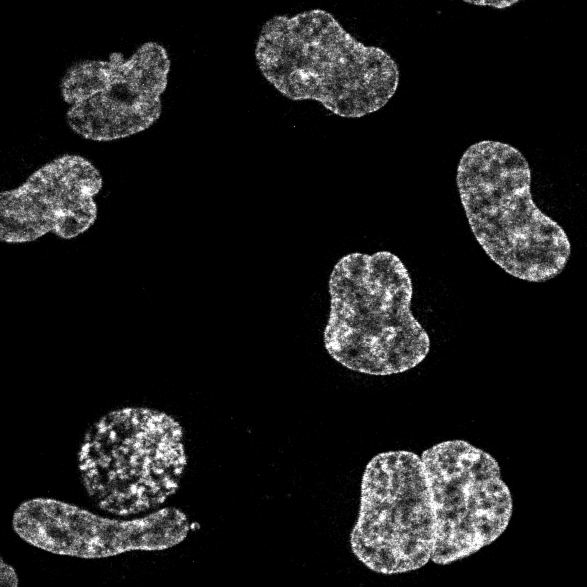

Supplement: Supplementary file 7 — Source Data for Figure 5 [file EMBR-24-e56841-s003.zip › Figure_5/5A/TEC_16_DKO_0.5h_Hoechst.tif]

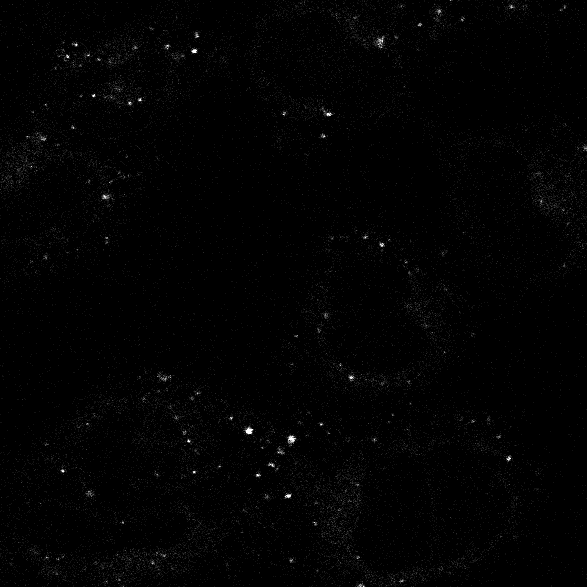

Supplement: Supplementary file 7 — Source Data for Figure 5 [file EMBR-24-e56841-s003.zip › Figure_5/5A/TEC_16_DKO_0.5h_LT.tif]

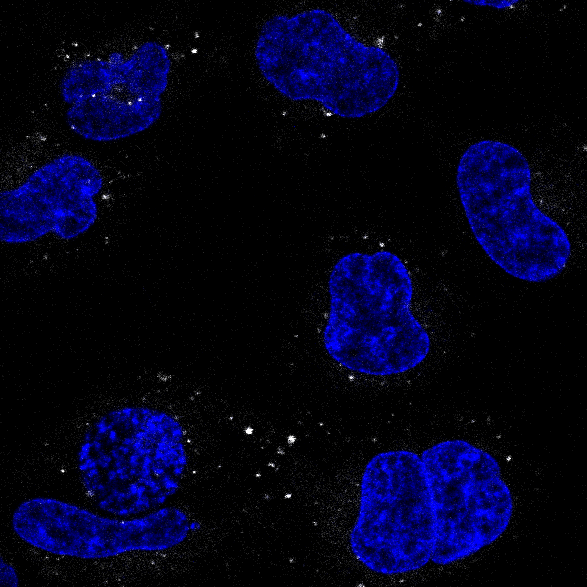

Supplement: Supplementary file 7 — Source Data for Figure 5 [file EMBR-24-e56841-s003.zip › Figure_5/5A/TEC_16_DKO_0.5h_merge.tif]

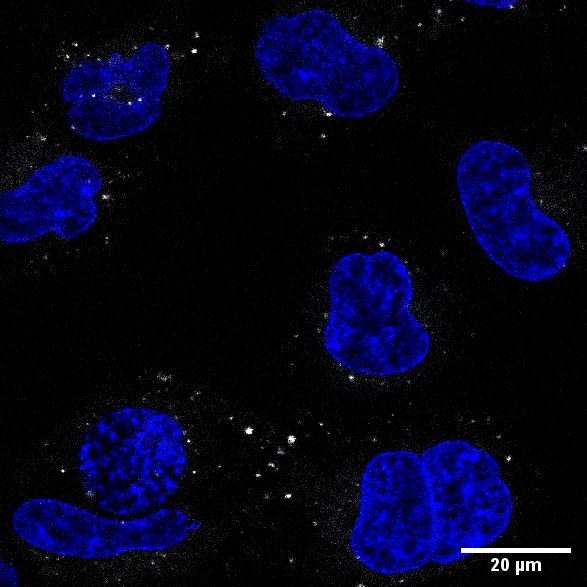

Supplement: Supplementary file 7 — Source Data for Figure 5 [file EMBR-24-e56841-s003.zip › Figure_5/5A/TEC_16_DKO_0.5h_scale.tif]

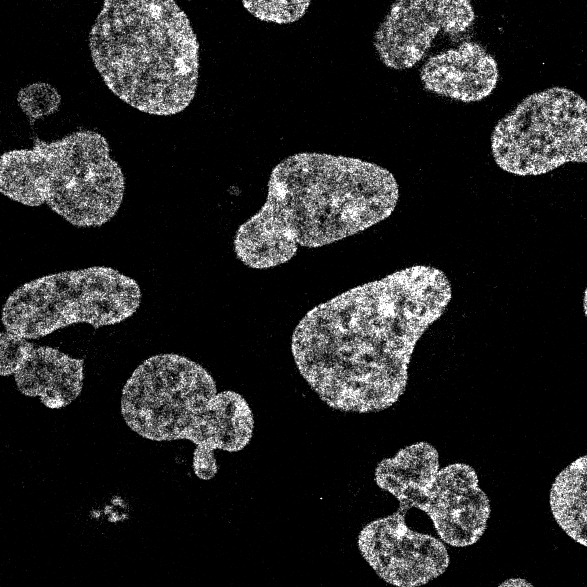

Supplement: Supplementary file 7 — Source Data for Figure 5 [file EMBR-24-e56841-s003.zip › Figure_5/5A/TEC_16_DKO_0h_Hoechst.tif]

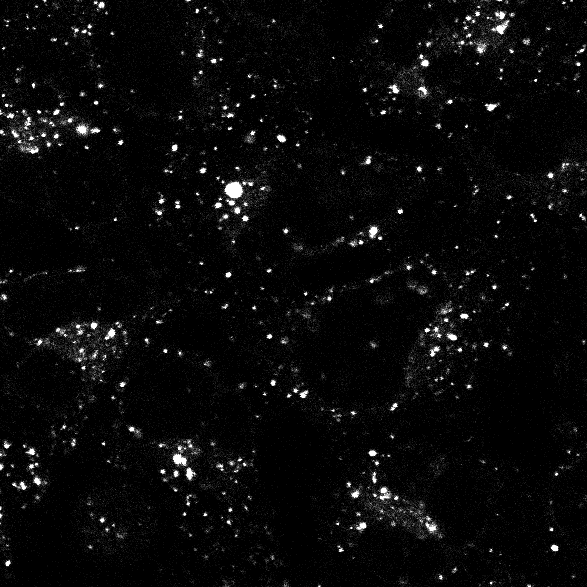

Supplement: Supplementary file 7 — Source Data for Figure 5 [file EMBR-24-e56841-s003.zip › Figure_5/5A/TEC_16_DKO_0h_LT.tif]

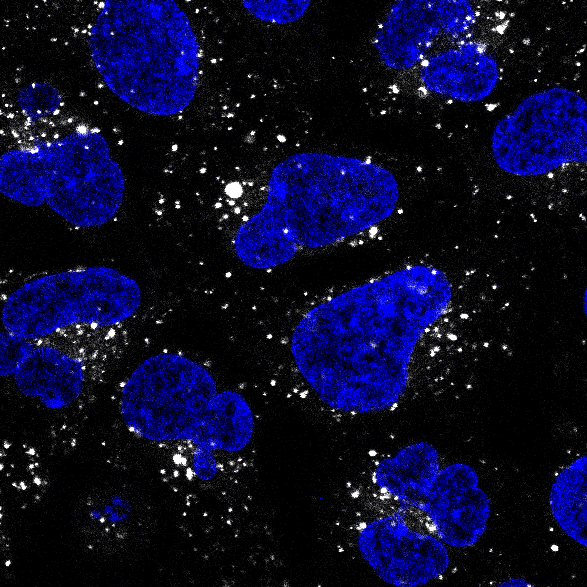

Supplement: Supplementary file 7 — Source Data for Figure 5 [file EMBR-24-e56841-s003.zip › Figure_5/5A/TEC_16_DKO_0h_merge.tif]

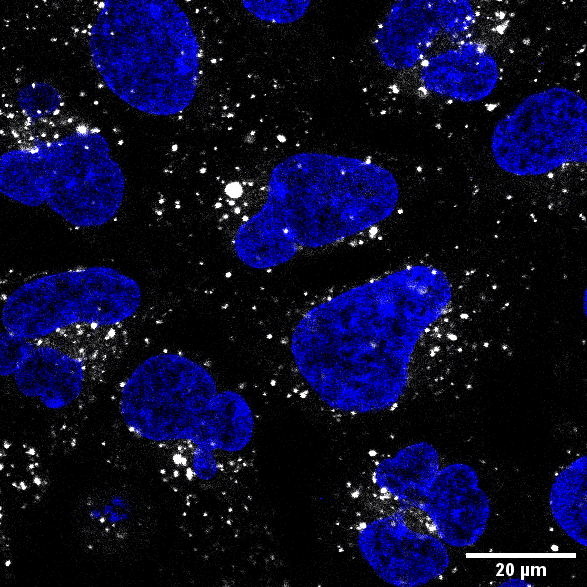

Supplement: Supplementary file 7 — Source Data for Figure 5 [file EMBR-24-e56841-s003.zip › Figure_5/5A/TEC_16_DKO_0h_scale.tif]

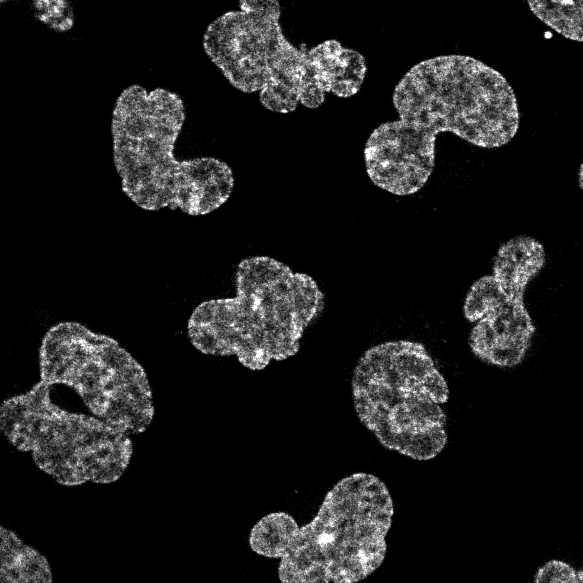

Supplement: Supplementary file 7 — Source Data for Figure 5 [file EMBR-24-e56841-s003.zip › Figure_5/5A/TEC_16_DKO_1h_Hoechst.tif]

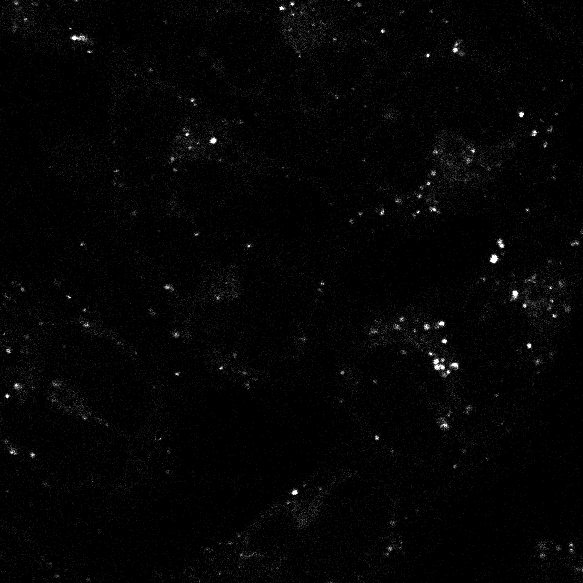

Supplement: Supplementary file 7 — Source Data for Figure 5 [file EMBR-24-e56841-s003.zip › Figure_5/5A/TEC_16_DKO_1h_LT.tif]

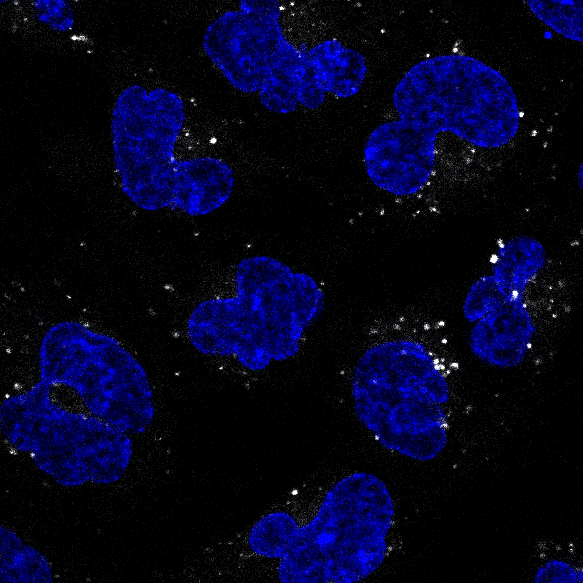

Supplement: Supplementary file 7 — Source Data for Figure 5 [file EMBR-24-e56841-s003.zip › Figure_5/5A/TEC_16_DKO_1h_merge.tif]

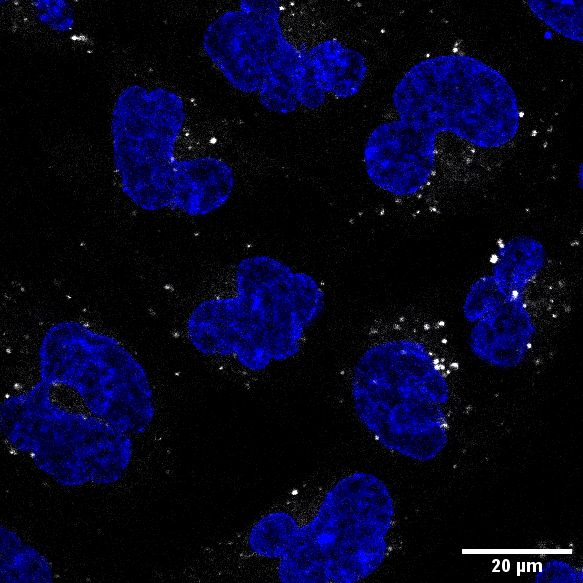

Supplement: Supplementary file 7 — Source Data for Figure 5 [file EMBR-24-e56841-s003.zip › Figure_5/5A/TEC_16_DKO_1h_scale.tif]

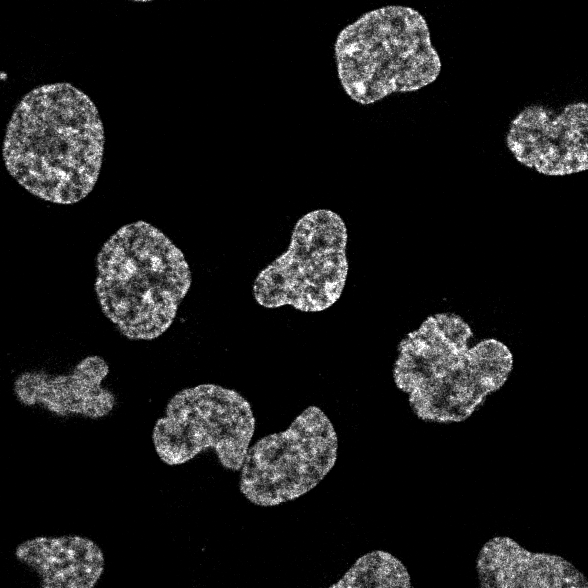

Supplement: Supplementary file 7 — Source Data for Figure 5 [file EMBR-24-e56841-s003.zip › Figure_5/5A/TEC_16_DKO_LLOMe_Hoechst.tif]

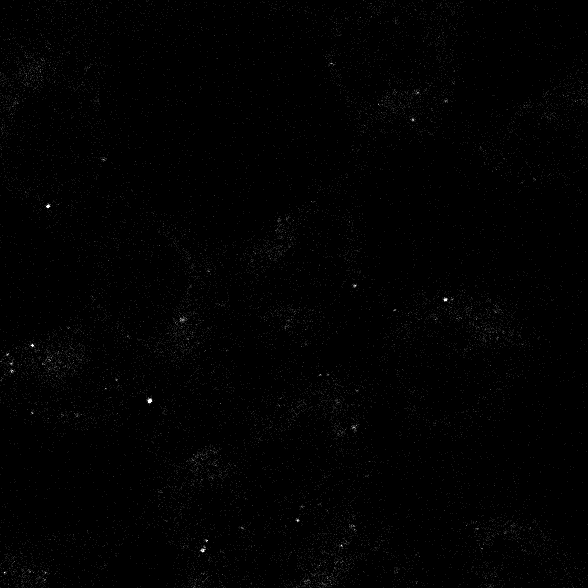

Supplement: Supplementary file 7 — Source Data for Figure 5 [file EMBR-24-e56841-s003.zip › Figure_5/5A/TEC_16_DKO_LLOMe_LT.tif]

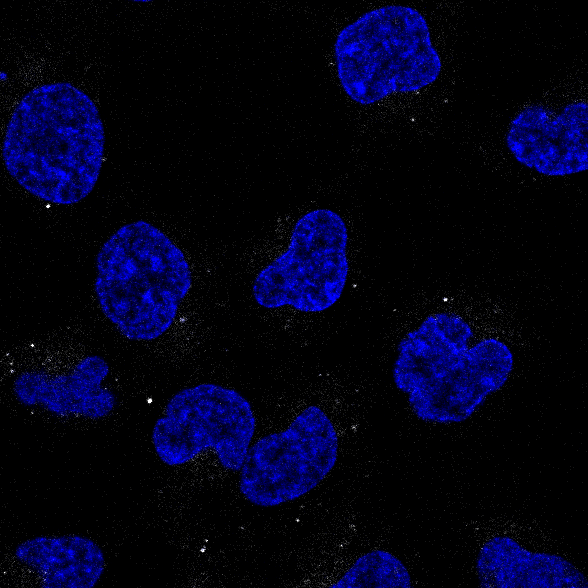

Supplement: Supplementary file 7 — Source Data for Figure 5 [file EMBR-24-e56841-s003.zip › Figure_5/5A/TEC_16_DKO_LLOMe_merge.tif]

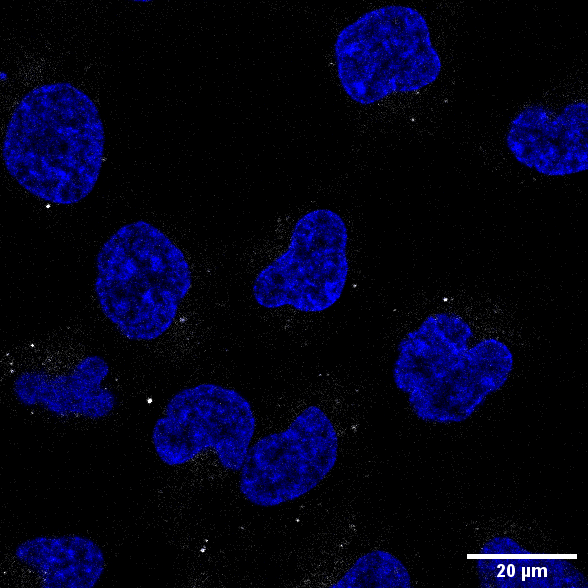

Supplement: Supplementary file 7 — Source Data for Figure 5 [file EMBR-24-e56841-s003.zip › Figure_5/5A/TEC_16_DKO_LLOMe_scale.tif]

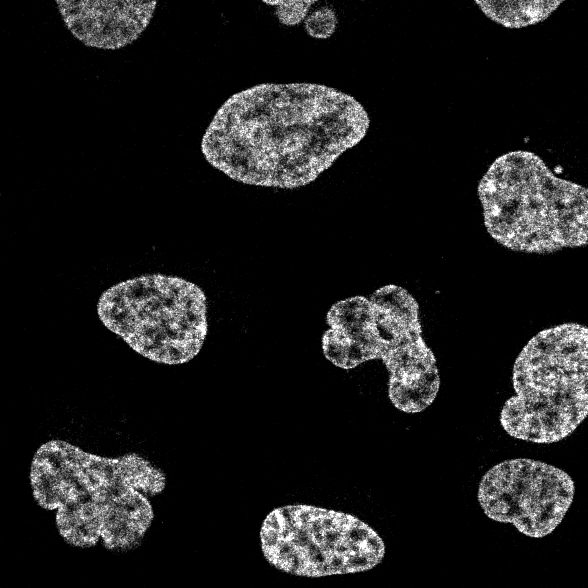

Supplement: Supplementary file 7 — Source Data for Figure 5 [file EMBR-24-e56841-s003.zip › Figure_5/5A/WT_0.5h_Hoechst.tif]

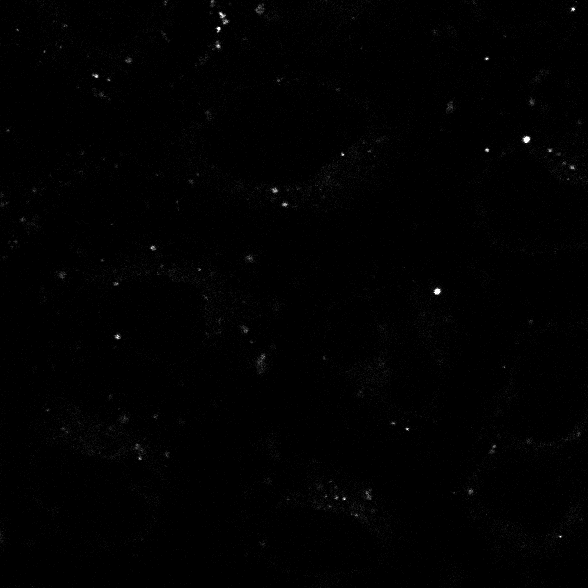

Supplement: Supplementary file 7 — Source Data for Figure 5 [file EMBR-24-e56841-s003.zip › Figure_5/5A/WT_0.5h_LT.tif]

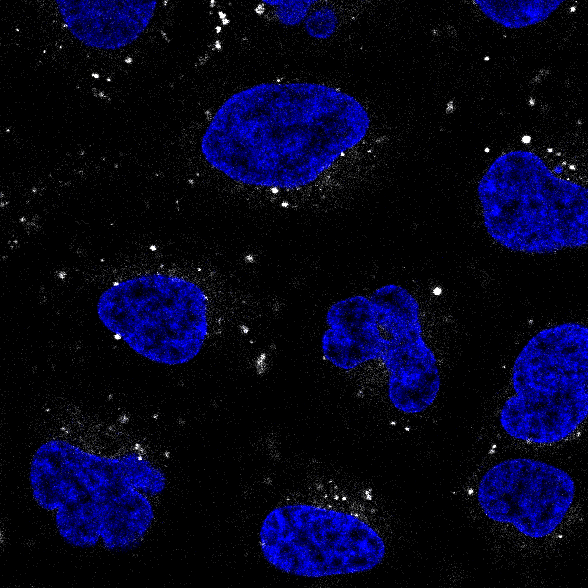

Supplement: Supplementary file 7 — Source Data for Figure 5 [file EMBR-24-e56841-s003.zip › Figure_5/5A/WT_0.5h_merge.tif]

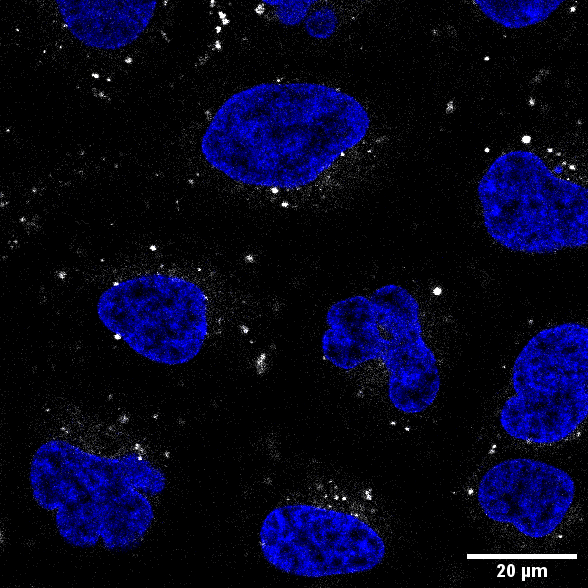

Supplement: Supplementary file 7 — Source Data for Figure 5 [file EMBR-24-e56841-s003.zip › Figure_5/5A/WT_0.5h_scale.tif]

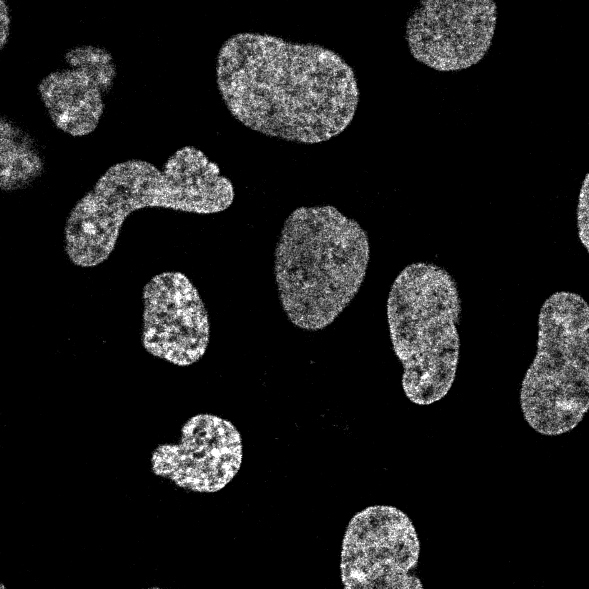

Supplement: Supplementary file 7 — Source Data for Figure 5 [file EMBR-24-e56841-s003.zip › Figure_5/5A/WT_0h_Hoechst.tif]

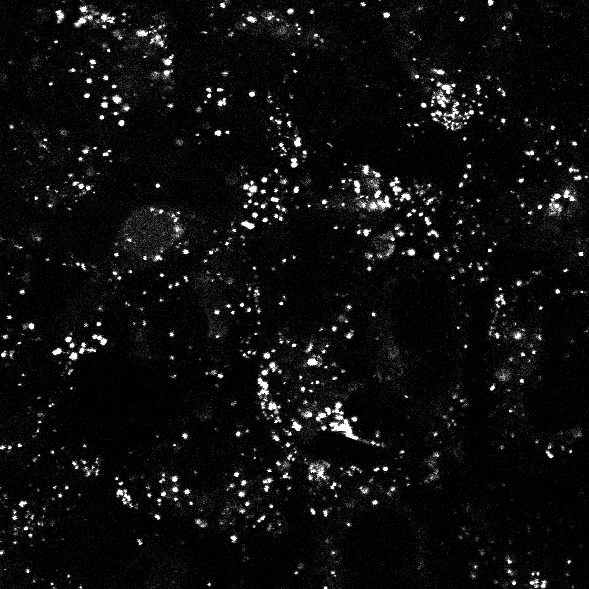

Supplement: Supplementary file 7 — Source Data for Figure 5 [file EMBR-24-e56841-s003.zip › Figure_5/5A/WT_0h_LT.tif]

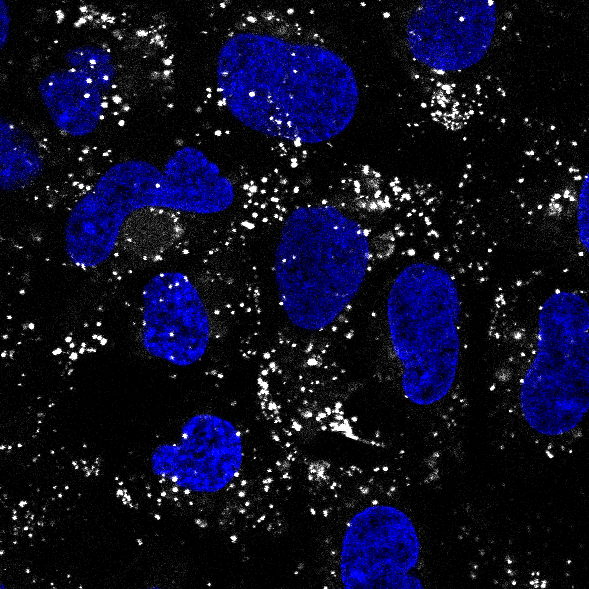

Supplement: Supplementary file 7 — Source Data for Figure 5 [file EMBR-24-e56841-s003.zip › Figure_5/5A/WT_0h_merge.tif]

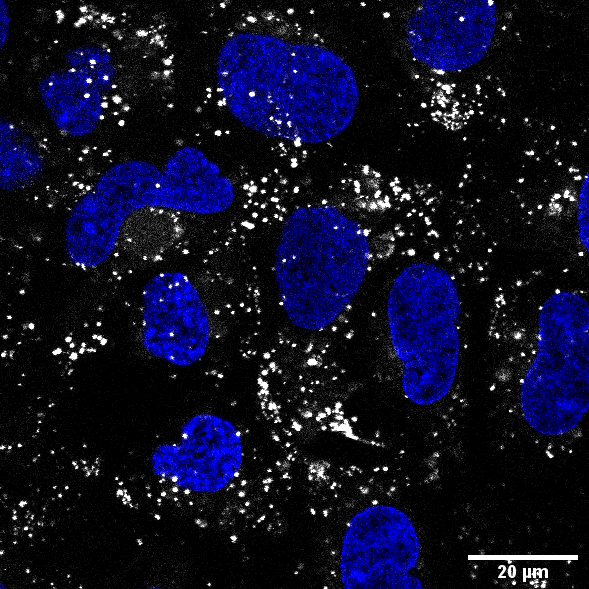

Supplement: Supplementary file 7 — Source Data for Figure 5 [file EMBR-24-e56841-s003.zip › Figure_5/5A/WT_0h_scale.tif]

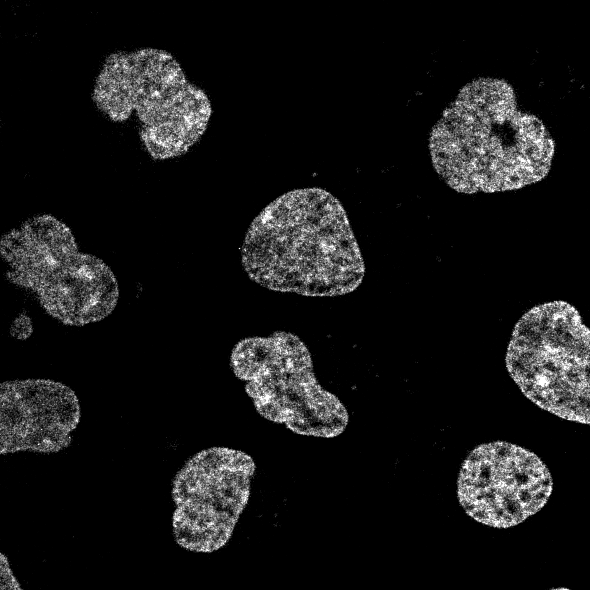

Supplement: Supplementary file 7 — Source Data for Figure 5 [file EMBR-24-e56841-s003.zip › Figure_5/5A/WT_1h_Hoechst.tif]

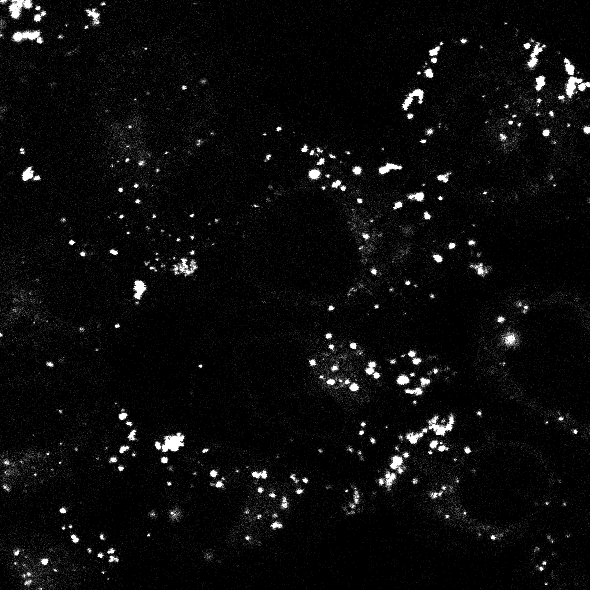

Supplement: Supplementary file 7 — Source Data for Figure 5 [file EMBR-24-e56841-s003.zip › Figure_5/5A/WT_1h_LT.tif]

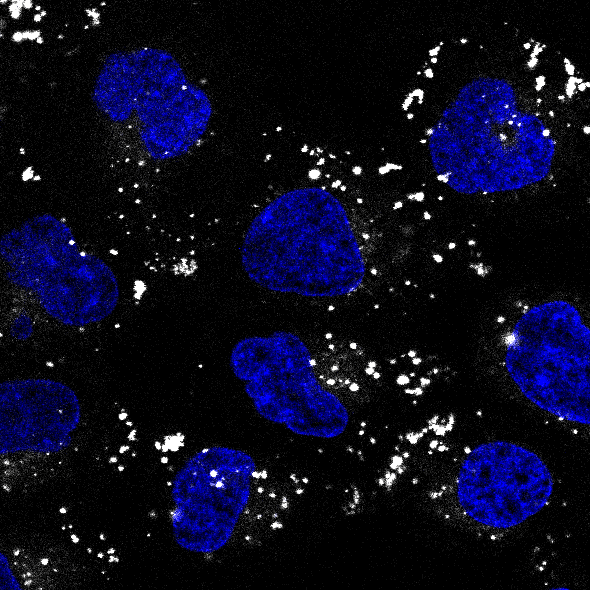

Supplement: Supplementary file 7 — Source Data for Figure 5 [file EMBR-24-e56841-s003.zip › Figure_5/5A/WT_1h_merge.tif]

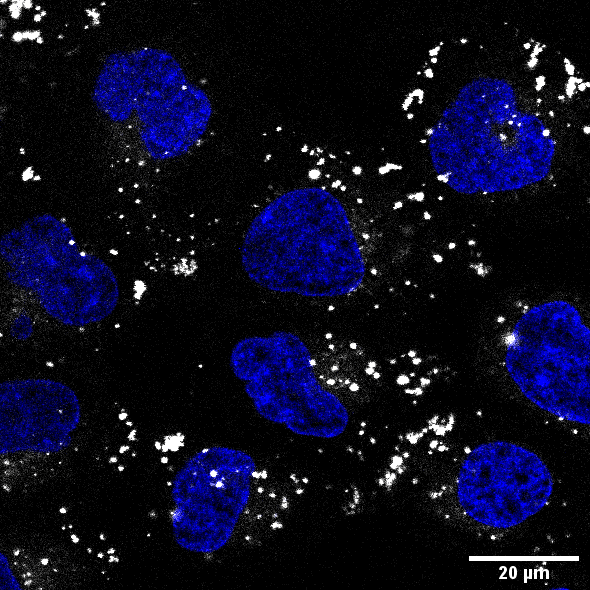

Supplement: Supplementary file 7 — Source Data for Figure 5 [file EMBR-24-e56841-s003.zip › Figure_5/5A/WT_1h_scale.tif]

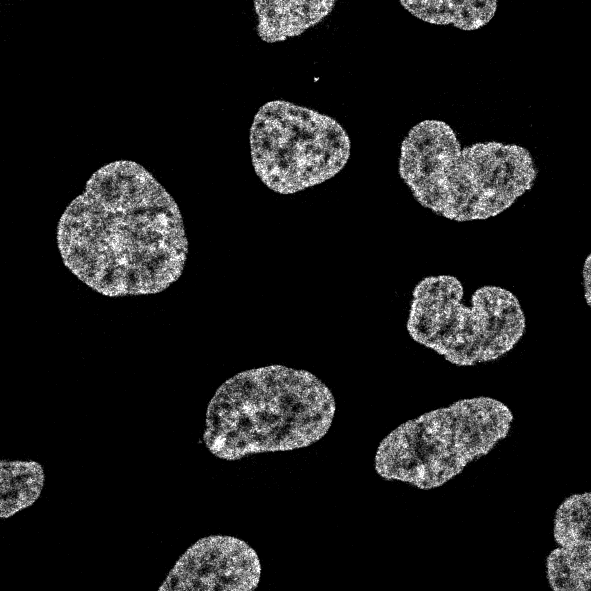

Supplement: Supplementary file 7 — Source Data for Figure 5 [file EMBR-24-e56841-s003.zip › Figure_5/5A/WT_LLOMe_Hoechst.tif]

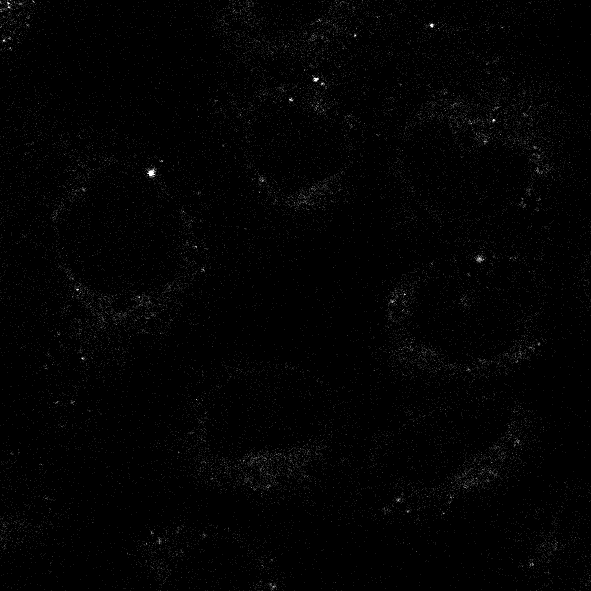

Supplement: Supplementary file 7 — Source Data for Figure 5 [file EMBR-24-e56841-s003.zip › Figure_5/5A/WT_LLOMe_LT.tif]

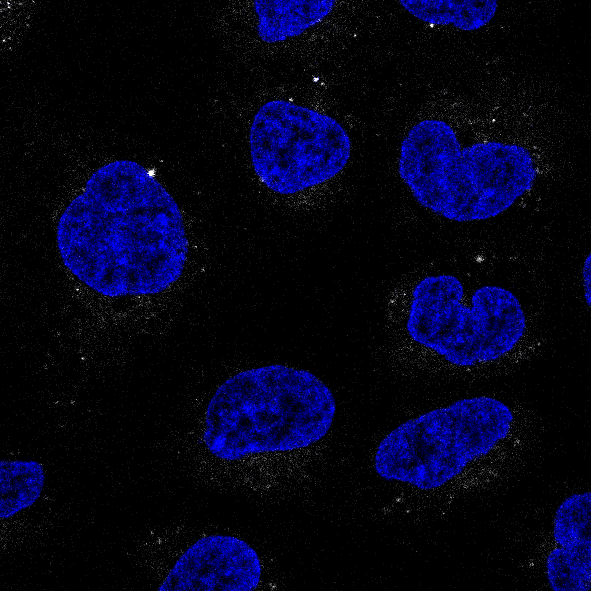

Supplement: Supplementary file 7 — Source Data for Figure 5 [file EMBR-24-e56841-s003.zip › Figure_5/5A/WT_LLOMe_merge.tif]

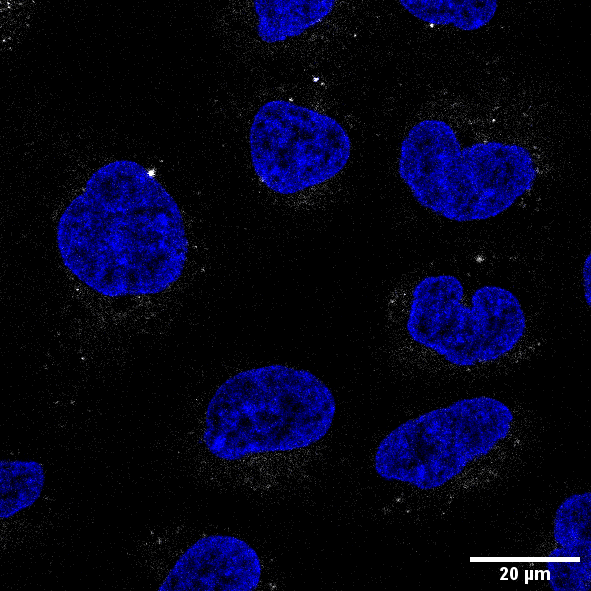

Supplement: Supplementary file 7 — Source Data for Figure 5 [file EMBR-24-e56841-s003.zip › Figure_5/5A/WT_LLOMe_scale.tif]
