# Supplementary material for: An ATG12‐ATG5‐TECPR1 E3‐like complex regulates unconventional LC3 lipidation at damaged lysosomes
Source: EMBO Rep. 2023 Jun 29;24(9):e56841. doi: 10.15252/embr.202356841 (PMC10481663; doi:10.15252/embr.202356841)
Supplement: Supplementary file 8 [file EMBR-24-e56841-s002.pdf]

EMBO reports

An ATG12-ATG5-TECPR1 E3-like complex regulates unconventional LC3 lipidation at damaged lysosomes

Dale Corkery, Sergio Castro-Gonzalez, Anastasia Knyazeva, Laura Herzog, and Yaowen Wu
DOI: 10.15252/embr.202356841

Corresponding author(s): Yaowen Wu (yaowen.wu@umu.se)

**Review Timeline:**		
	Submission Date:	16th Jan 23
	Editorial Decision:	27th Jan 23
	Revision Received:	26th Apr 23
	Editorial Decision:	12th Jun 23
	Revision Received:	12th Jun 23
	Accepted:	13th Jun 23

Editor: Martina Rembold

Transaction Report: This manuscript was transferred to EMBO reports following peer review at The EMBO Journal.

(Note: With the exception of the correction of typographical or spelling errors that could be a source of ambiguity, letters and reports are not edited. Depending on transfer agreements, referee reports obtained elsewhere may or may not be included in this compilation. Referee reports are anonymous unless the Referee chooses to sign their reports.)

Referee #1:

The authors of this manuscript investigated the function of TECPR1 in response to lysosomal membrane damage. They found that lysosomal damage induced by treatment of cells with LLOMe induced the recruitment of TECPR1 to lysosomes, which catalyzed lipidation of ATG8 proteins to lysosomal membranes. They found that the first of two DysF domains is essential for TECPR1 recruitment to damaged lysosomes. An accompanying manuscript by Randow and colleagues found that this domain binds sphingomyelin (SM). The authors investigated whether SM is also recognized during the TECPR1 mediated ATG8 lipidation on damaged lysosomes using a mutant described by Randow and colleagues. This mutant also abolished recruitment of TECPR1 to damaged lysosomes, showing that the TECPR1 mediated stress response is elicited by several different membrane damaging agents.
Overall, this manuscript falls short in revealing new insights into TECPR1 mediated stress response as major insights into the mechanism were provided by an accompanying manuscript. Most importantly, it remains unknown how TECPR1 mediated ATG8 lipidation resolves membrane damage and how important TECPR1 is compared to ATG16L1, given that the ATG16L1 mediated response appears to be much more potent. The study is too premature to be considered for publication in the EMBO J.

Major points:

1) The authors investigated the function of TECPR1 and its mutants using wildtype cells. To evaluate the function of the TECPR1 mutants, the authors should use TECPR1 knock out cells to avoid crosstalk of native TECPR1.
2) The authors compared TECPR1 and ATG16L1 mediated lipidation of ATG8 proteins. For these experiments TECPR1/ATG16L1 double knock out cells should be used to reveal the functional contributions of TECPR1 and ATG16L1, respectively.
3) The significance of TECPR1 mediated ATG8 lipidation for membrane damage repair remains uncharacterized. To provide evidence for the importance of TECPR1, the authors should use pulse chase experiments and induce damage by LLOMe treatment of TECPR1 KO and ATG16L1/TECPR1 DKO cells, followed by chase periods of different times and evaluation of remaining lysosomal damage.

Referee #2:

Damaged lysosomes are harmful for cells. Cells have developed several defense systems including lysophagy, ESCRT dependent lysosomal repair and TFEB activation to maintain lysosomal integrity. In the current study, Corkery et al. identified a novel role of TECPR1 during lysosomal damage. They found that TECPR1 is recruited on damaged lysosomes via N terminal dysferlin domain prior to induction of lysophagy. They found that TECPR1 function as an alternative E3 for ATG16L1 and mediate lipidation of LC3. Although authors' findings are potentially interesting, the reviewer finds the present work is too preliminary for EMBO J. Authors need to show the biological significance of TECPR1 dependent LC3 lipidation during lysosomal damage.

Major

1. Does TECPR1 have a critical role of lysosomal homeostasis after the damage? Authors need to check if the recovery of lysosomal functions (as revealed by lysotracker, MagicRed staining etc.) is impaired by TECPR1 knockdown after LLOMe wash-off. If so, TECPR1 work through any of endo-lysosomal damage response pathways (such as Myoferlin, TFEB, lysophagy etc.)?

2. Is there any functional difference between ATG16L1 and TECPR1 dependent LC3 lipidation? Does TECPR1 preferentially mediate lipidation of specific ATG8 subfamilies? Recent evidence suggests that LC3 conjugates to PS in addition to PE during non-canonical lipidation (Durgan et al., Mol Cell, 2021). Is TECPR1 preferentially lipidated to any of these?

3. Does TECPR1 indeed interact with ATG5-ATG12 complex during lysosomal damage in autophagy deficient cells? The complex mediates autophagosome-lysosome fusion through canonical LC3 lipidation on autophagosomes?

4. Does other lysosomal stress by Monensin, Nigericin and lysosomal calcium efflux induce the recruitment of TECPR1 on lysosome ?

5. In addition to the localization of TECPR1 W154 mutant shown in Figure EV1, the authors need to check whether TECPR1 lysosomal enrichment is suppressed with ectopic expression of nSMase2 to fully explain sphingomyelin-dependent TECPR1 recruitment to lysosomes.

6. The authors need to show the correlation of sphingomyelin exposure to cytosol and TECPR1 recruitment on lysosomes.

7. Does the exposure of sphingomyelin trigger TECPR1-mediated ATG8 lipidation specifically?

8. In Fig.4F, ATG5 recruitment on lysosomes also occurs independently from autophagosome formation? The authors need to show the depletion of TECPR1 suppress ATG5 recruitment to lysosomes.

Minor

1.Quantitative data is missing in Fig.1B, Fig.3A, D, and Fig.4 E, F.

Referee #3:

Corkery et al present a study in which they uncover an unexpected role for the TECPR1-ATG5 axis in driving non-canonical autophagy/CASM to damaged membranes. Authors find that upon lysosome damage with LLOMe, overexpressed TECPR1 is rapidly recruited to lysosomes in a manner dependent on the dysferlin domains. This recruitment precedes larger scale membrane damage and galectin recruitment. Strikingly, they show that damage involves ATG8/LC3 lipidation to lysosomes independent of canonical autophagy. Furthermore, the lipidation is partially independent of ATG16L1 while being completely reliant on ATG5 and ATG7. Authors suggest that the residual LC3 lioidation seen in ATG16L1 KO cells is supported by TECPR1-ATG5 recruitment.
This a well presented study, although it feels less advanced and somewhat less developed, with no exploration as to the function of the lipidation.

Major points

1. Authors should include some controls that their FIP200 and ATG16L1 KO are able to block LC3 lipidation to canonical autophagy induction (ie mTor inhibitor + bafilonmycin).
2. It is unclear as to the extent that the TECPR1 axis plays in the LLOME LC3 lipdiation response. Can authors deplete TECPR1 in wild type cells and test for the effect on LC3 lipidation?
3. Authors should acknowledge and comment on previous publications which demonstrated that LLOMe induced LC3 lipidation was blocked by ATG16L1 KO, with no residual lipidation remaining (Nakamura et al. PMID: 32989250). Furthermore, blocking of the ATG16L1-v-ATPase axis by SopF was previously seen to inhibit LLOMe induced lipidation (Xu et al. PMID:35046574). What are the differences between these studies.

Minor point
1. It is unclear as to what antibodies were used to for western blot against LC3A, GABARRAP and GABARAPL1 in Fig EV2. These are not mentioned in the materials and methods.

1st Editorial Decision **27th Jan 2023**

Dear Yaowen,

Thank you once more for the transfer of your research manuscript to our journal. As discussed, we would like to invite you to revise your manuscript for potential publication in EMBO Reports based on the referee reports obtained during peer review at The EMBO Journal.

As outlined earlier, it will be important that you 1) show biological significance by monitoring lysosomal damage and homeostasis and that you 2) provide some more data on the respective contribution of ATG16L1 and TECPR1 to LC3 lipidation.

As discussed further, I agree that question 2 from referee 2 is work for the future but I recommend addressing Q5, i.e., the nSMase2 treatment.

Please address all referee concerns in a complete point-by-point response. Acceptance of the manuscript will depend on a positive outcome of a second round of review. It is EMBO reports policy to allow a single round of revision only and acceptance or rejection of the manuscript will therefore depend on the completeness of your responses included in the next, final version of the manuscript.

We realize that it is difficult to revise to a specific deadline. In the interest of protecting the conceptual advance provided by the work, we recommend a revision within 3 months (April 27). Please discuss the revision progress ahead of this time with the editor if you require more time to complete the revisions.

I am also happy to discuss the revision further via e-mail or a video call, if you wish.

Your study has 4 figures and will therefore be published as a short report. For short reports, the revised manuscript should not exceed 27,000 characters (including spaces but excluding materials & methods and references) and 5 main plus 5 expanded view figures. The results and discussion sections must further be combined, which will help to shorten the manuscript text by eliminating some redundancy that is inevitable when discussing the same experiments twice.

*****IMPORTANT NOTE:
We perform an initial quality control of all revised manuscripts before re-review. Your manuscript will FAIL this control and the handling will be DELAYED if the following APPLIES:

1) A data availability section providing access to data deposited in public databases is missing. If you have not deposited any data, please add a sentence to the data availability section that explains that.

2) Your manuscript contains statistics and error bars based on n=2. Please use scatter blots in these cases. No statistics should be calculated if n=2.

When submitting your revised manuscript, please carefully review the instructions that follow below. Failure to include requested items will delay the evaluation of your revision.*****

When submitting your revised manuscript, we will require:

1) a .docx formatted version of the manuscript text (including legends for main figures, EV figures and tables). Please make sure that the changes are highlighted to be clearly visible.

2) individual production quality figure files as .eps, .tif, .jpg (one file per figure).
Please download our Figure Preparation Guidelines (figure preparation pdf) from our Author Guidelines pages https://www.embopress.org/page/journal/14693178/authorguide for more info on how to prepare your figures.

3) a .docx formatted letter INCLUDING the reviewers' reports and your detailed point-by-point responses to their comments. As part of the EMBO Press transparent editorial process, the point-by-point response is part of the Review Process File (RPF), which will be published alongside your paper.

4) a complete author checklist, which you can download from our author guidelines (). Please insert information in the checklist that is also reflected in the manuscript. The completed author checklist will also be part of the RPF.

5) Please note that all corresponding authors are required to supply an ORCID ID for their name upon submission of a revised manuscript (). Please find instructions on how to link your ORCID ID to your account in our manuscript tracking system in our Author guidelines

()

6) We replaced Supplementary Information with Expanded View (EV) Figures and Tables that are collapsible/expandable online. A maximum of 5 EV Figures can be typeset. EV Figures should be cited as 'Figure EV1, Figure EV2" etc... in the text and their respective legends should be included in the main text after the legends of regular figures.

- For the figures that you do NOT wish to display as Expanded View figures, they should be bundled together with their legends in a single PDF file called *Appendix*, which should start with a short Table of Content. Appendix figures should be referred to in the main text as: "Appendix Figure S1, Appendix Figure S2" etc. See detailed instructions regarding expanded view here:

- Additional Tables/Datasets should be labeled and referred to as Table EV1, Dataset EV1, etc. Legends have to be provided in a separate tab in case of .xls files. Alternatively, the legend can be supplied as a separate text file (README) and zipped together with the Table/Dataset file.

7) Please note that a Data Availability section at the end of Materials and Methods is now mandatory. In case you have no data that requires deposition in a public database, please state so instead of refereeing to the database.
See also < https://www.embopress.org/page/journal/14693178/authorguide#dataavailability>). Please note that the Data Availability Section is restricted to new primary data that are part of this study.

8) At EMBO Press we ask authors to provide source data for the main figures. Our source data coordinator will contact you to discuss which figure panels we would need source data for and will also provide you with helpful tips on how to upload and organize the files.

Additional information on source data and instruction on how to label the files are available .

9) The journal requires a statement specifying whether or not authors have competing interests (defined as all potential or actual interests that could be perceived to influence the presentation or interpretation of an article). In case of competing interests, this must be specified in your disclosure statement. Further information: https://www.embopress.org/competing-interests

10) Figure legends and data quantification:
The following points must be specified in each figure legend:

- the name of the statistical test used to generate error bars and P values,
- the number (n) of independent experiments (please specify technical or biological replicates) underlying each data point,
- the nature of the bars and error bars (s.d., s.e.m.)

- If the data are obtained from n {less than or equal to} 5, show the individual data points in addition to the SD or SEM.
- If the data are obtained from n {less than or equal to} 2, use scatter blots showing the individual data points.

Discussion of statistical methodology can be reported in the materials and methods section, but figure legends should contain a basic description of n, P and the test applied.

See also the guidelines for figure legend preparation:
https://www.embopress.org/page/journal/14693178/authorguide#figureformat

- Please also include scale bars in all microscopy images.

11) Our journal encourages inclusion of *data citations in the reference list* to directly cite datasets that were re-used and obtained from public databases. Data citations in the article text are distinct from normal bibliographical citations and should directly link to the database records from which the data can be accessed. In the main text, data citations are formatted as follows: "Data ref: Smith et al, 2001" or "Data ref: NCBI Sequence Read Archive PRJNA342805, 2017". In the Reference list, data citations must be labeled with "[DATASET]". A data reference must provide the database name, accession number/identifiers and a resolvable link to the landing page from which the data can be accessed at the end of the reference. Further instructions are available at .

12) As part of the EMBO publication's Transparent Editorial Process, EMBO reports publishes online a Review Process File to accompany accepted manuscripts. This File will be published in conjunction with your paper and will include the referee reports, your point-by-point response and all pertinent correspondence relating to the manuscript.

You are able to opt out of this by letting the editorial office know (emboreports@embo.org). If you do opt out, the Review Process File link will point to the following statement: "No Review Process File is available with this article, as the authors have chosen not to make the review process public in this case."

We would also welcome the submission of cover suggestions, or motifs to be used by our Graphics Illustrator in designing a cover.

I look forward to seeing a revised form of your manuscript when it is ready and please let me know if you have questions or comments regarding the revision.

Please use this link to submit your revision: https://embor.msubmit.net/cgi-bin/main.plex

Kind regards,

Martina

Martina Rembold, PhD
Senior Editor
EMBO reports

Point-by-point responses to Reviewers:

Referee #1:

The authors of this manuscript investigated the function of TECPR1 in response to lysosomal membrane damage. They found that lysosomal damage induced by treatment of cells with LLOMe induced the recruitment of TECPR1 to lysosomes, which catalyzed lipidation of ATG8 proteins to lysosomal membranes. They found that the first of two DysF domains is essential for TECPR1 recruitment to damaged lysosomes. An accompanying manuscript by Randow and colleagues found that this domain binds sphingomyelin (SM). The authors investigated whether SM is also recognized during the TECPR1 mediated ATG8 lipidation on damaged lysosomes using a mutant described by Randow and colleagues. This mutant also abolished recruitment of TECPR1 to damaged lysosomes, showing that the TECPR1 mediated stress response is elicited by several different membrane damaging agents.

Overall, this manuscript falls short in revealing new insights into TECPR1 mediated stress response as major insights into the mechanism were provided by an accompanying manuscript. Most importantly, it remains unknown how TECPR1 mediated ATG8 lipidation resolves membrane damage and how important TECPR1 is compared to ATG16L1, given that the ATG16L1 mediated response appears to be much more potent. The study is too premature to be considered for publication in the EMBO J.

Major points:

1) The authors investigated the function of TECPR1 and its mutants using wildtype cells. To evaluate the function of the TECPR1 mutants, the authors should use TECPR1 knock out cells to avoid crosstalk of native TECPR1.

Re1: The mutant TECPR1 data presented in Figure 2 was done simply to evaluate which domain of TECPR1 was required for LMP-induced recruitment to lysosomes. Thus, crosstalk with native TECPR1 is not a concern here. To investigate the function of TECPR1 we have now generated TECPR1 knock-out cells (**Fig EV3**) and performed an addback experiment with wild-type TECPR1 or the TECPR1 deletion mutant lacking the N-terminal dysferlin domain required for LMP-induced lysososmal recruitment (**Fig 4H and I**). This data confirms that TECPR1 recruitment to damaged lysosomal membranes is essential for ATG16L1-independent LC3 lipidation.

2) The authors compared TECPR1 and ATG16L1 mediated lipidation of ATG8 proteins. For these experiments TECPR1/ATG16L1 double knock out cells should be used to reveal the functional contributions of TECPR1 and ATG16L1, respectively.

Re2: We have generated TECPR1/ATG16L1 double knock out cells (**Fig EV3**) and provided a detailed characterization of LC3 lipidation (**Fig 4H**) and lysosomal recovery (**Fig 5**) after LMP. Our data demonstrates that, in the absence of ATG16L1, TECPR1 forms an E3-like complex with ATG5-ATG12 to promote LC3 lipidation at damaged lysosomal membranes. In the presence of ATG16L1, TECPR1 knockout did not significantly reduce LMP-induced LC3 lipidation as we would expect if the TECPR1-dependent pool of lipidated LC3 was distinct from LC3 lipidated via the ATG16L1 complex. However, loss of TECPR1 has been shown to impair autophagosome-lysosome fusion (Chen *et al.*, 2012), so we

can't exclude the possibility that loss of TECPR1-dependent LC3 lipidation is masked by elevated ATG16L1-dependent LC3 lipidation as a consequence of impaired autophagy flux. This caveat has been discussed in the text.

Notably, single knockout of either ATG16L1 or TECPR1 did not significantly impair lysosomal recovery (**Fig 5**). Knock-out of both led to a reduction in basal LysoTracker staining as well as a significant impairment in lysosomal recovery following LMP. This data confirms the requirement for lipidated LC3 in lysosomal recovery from LMP and suggests a certain degree of functional redundancy between ATG16L1 and TECPR1 E3-like complexes.

3) The significance of TECPR1 mediated ATG8 lipidation for membrane damage repair remains uncharacterized. To provide evidence for the importance of TECPR1, the authors should use pulse chase experiments and induce damage by LLOMe treatment of TECPR1 KO and ATG16L1/TECPR1 DKO cells, followed by chase periods of different times and evaluation of remaining lysosomal damage.

Re3: We thank the reviewer for this suggestion. See also Re2. We have performed an LLOMe pulse-chase experiment in WT, TECPR1-KO, ATG16L1-KO and TECPR1/ATG16L double-KO cell lines using LysoTracker staining as a readout of lysosomal recovery after damage (**Fig 5**). Single knockout of either TECPR1 or ATG16L1 did not significantly impair lysosomal recovery, while double knock-out did. This would suggest, in relation to point #2, that there is a degree of redundancy to the functional contributions of TECPR1 and ATG16L1. Thus, both important.

Referee #2:

Damaged lysosomes are harmful for cells. Cells have developed several defense systems including lysophagy, ESCRT dependent lysosomal repair and TFEB activation to maintain lysosomal integrity. In the current study, Corkery et al. identified a novel role of TECPR1 during lysosomal damage. They found that TECPR1 is recruited on damaged lysosomes via N terminal dysferlin domain prior to induction of lysophagy. They found that TECPR1 function as an alternative E3 for ATG16L1 and mediate lipidation of LC3. Although authors' findings are potentially interesting, the reviewer finds the present work is too preliminary for EMBO J. Authors need to show the biological significance of TECPR1 dependent LC3 lipidation during lysosomal damage.

Major

1. Does TECPR1 have a critical role of lysosomal homeostasis after the damage? Authors need to check if the recovery of lysosomal functions (as revealed by lysotracker, MagicRed staining etc.) is impaired by TECPR1 knockdown after LLOMe wash-off. If so, TECPR1 work through any of endo-lysosomal damage response pathways (such as Myoferlin, TFEB, lysophagy etc.)?

Re4: As described in response to point #3 from referee #1 (Re3), we have performed an LLOMe pulse-chase experiment in WT, TECPR1-KO, ATG16L1-KO and TECPR1/ATG16L double-KO cell lines using LysoTracker staining as a readout of lysosomal recovery after damage (**Fig 5**). The results of this experiment would suggest that TECPR1 and ATG16L1 play functionally redundant roles in the response to LMP. This, of course, doesn't mean that both E3-like complexes function through the same mechanism and future studies will explore potential structural/mechanistic differences in detail.

2. Is there any functional difference between ATG16L1 and TECPR1 dependent LC3 lipidation? Does TECPR1 preferentially mediate lipidation of specific ATG8 subfamilies? Recent evidence suggests that LC3 conjugates to PS in addition to PE during non-canonical lipidation (Durgan et al., Mol Cell, 2021). Is TECPR1 preferentially lipidated to any of these?

Re5: Excellent question. Recent reports have shown that lysosomal membrane damage induces PS enrichment (Radulovic et al, 2022; Tan & Finkel, 2022) at the damaged membrane so the stage appears set for PS-conjugation. Addressing the lipid preference of the two E3-like complexes is a large undertaking, outside the scope of this manuscript, but an avenue actively being pursued.

3. Does TECPR1 indeed interact with ATG5-ATG12 complex during lysosomal damage in autophagy deficient cells? The complex mediates autophagosome-lysosome fusion through canonical LC3 lipidation on autophagosomes?

Re6: The EGFP-ATG5 recruitment data (**Fig 4G**) is presented as confirmation of previous reports characterizing the TECPR1-ATG5 interaction (Behrends et al, 2010; Chen et al., 2012). We observe the same recruitment in ATG16L1KO cells.

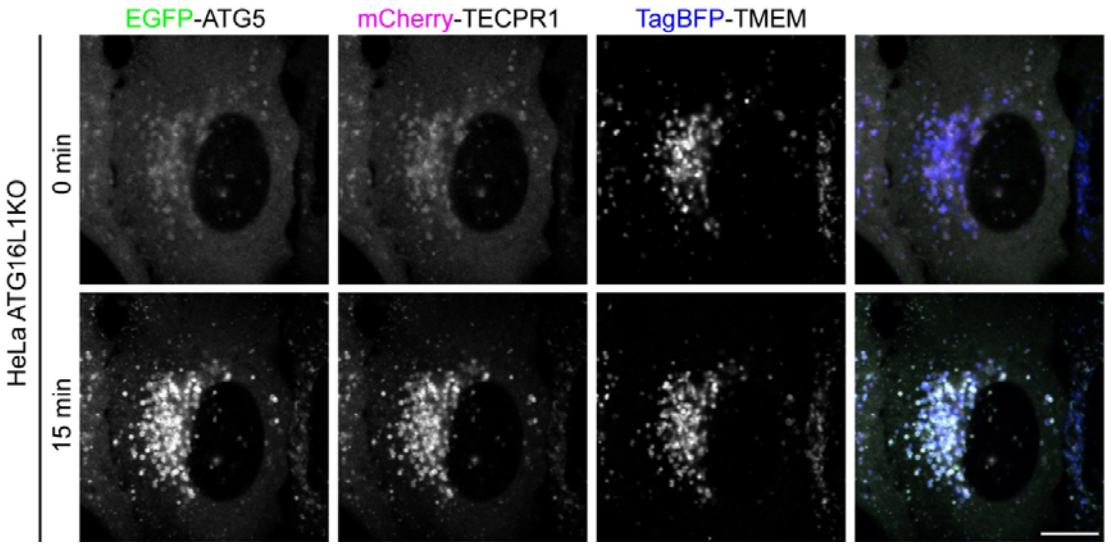

To confirm the importance of the TECPR1-ATG5 interaction, we have shown that ATG5 is essential for LMP-induced lipidation (**Fig 4C**), and that TECPR1 is essential for LC3 lipidation in autophagy deficient (ATG16L1KO) cells (**Fig 4H**). Adding back the TECPR1$^{\Delta 1\text{-}170}$ mutant lacking the N-terminal dysferlin domain required for LMP-induced lysosome recruitment does not restore LC3 lipidation in ATG16L1/TECPR1 double knockout cells (**Fig 4I**). This supports our claim that TECPR1 forms an E3-like complex with the ATG5-ATG12 conjugate to recruit the lipidation machinery to the damaged membranes. Whether this E3-like complex is constitutively assembled, or assembly occurs at the membrane in response to damage, remains to be determined.

4. Does other lysosomal stress by Monensin, Nigericin and lysosomal calcium efflux induce the recruitment of TECPR1 on lysosome?

Re7: We have expanded our investigation of TECPR1 lysosomal recruitment to include Monensin, Nigericin and the TRPML1 agonist, ML-SA1 (**Fig 1D/E**). Ionophore treatment is able to induce TECPR1 recruitment although it is less robust, and requires longer treatment times as compared to the

damaging agents (LLOMe/GPN). Calcium efflux, in our hands, had no effect. Interestingly, only the damaging agents appeared to induce ATG16L1-independent LC3 lipidation (**Fig EV2D**) at the treatment times/concentrations used in this study.

5. In addition to the localization of TECPR1 W154 mutant shown in Figure EV1, the authors need to check whether TECPR1 lysosomal enrichment is suppressed with ectopic expression of nSMase2 to fully explain sphingomyelin-dependent TECPR1 recruitment to lysosomes.

Re8: We thank the reviewer for this suggestion. We have now shown that ectopic expression of a sphingomyelinase (SMase) from *Bacillus cereus* targeted to the cytosolic surface of the lysosome is sufficient to inhibit LMP-dependent TECPR1 recruitment (**Fig EV1D/E**).

6. The authors need to show the correlation of sphingomyelin exposure to cytosol and TECPR1 recruitment on lysosomes.

Re9: Without a biosensor of sphingomyelin, we were unable to characterize the timing of TECPR1 recruitment relative to sphingomyelin exposure. This was however, covered extensively in the companion manuscript.

7. Does the exposure of sphingomyelin trigger TECPR1-mediated ATG8 lipidation specifically?

Re10: We have demonstrated that sphingomyelin exposure is essential for TECPR1 recruitment (**Fig EV1**) and that TECPR1 recruitment is essential for ATG16L1-independent ATG8 lipidation (**Fig 4H/I**). Whether or not the direct interaction between TECPR1 and sphingomyelin influences E3-like complex assembly/structure in a way that promotes its function/activity is actively under investigation.

8. In Fig.4F, ATG5 recruitment on lysosomes also occurs independently from autophagosome formation? The authors need to show the depletion of TECPR1 suppress ATG5 recruitment to lysosomes.

Re11: Immunofluorescence (**Fig 4E**) and Lyso-IP (**Fig 4F**) data confirm that LC3 is being conjugated to damaged lysosomal membranes in autophagy-deficient (ATG16L1 KO) cells. This conjugation is dependent on ATG5 (**Fig 4C**) suggesting the ATG5-ATG12 conjugate is recruited to the damaged membranes independently from autophagosome formation. Depletion of TECPR1 (**Fig 4H and EV4**) (a known ATG5-interacting protein), or inhibition of TECPR1 LMP-induced lysosomal recruitment (**Fig 4I**) is sufficient to prevent ATG16L1-independent LC3 lipidation. So, while technical challenges prevented us from directly exploring ATG5 recruitment in TECPR1 KO cells, we believe we have sufficiently demonstrated the importance of a TECPR1-ATG5-ATG12 complex in regulating ATG16L1-independent ATG8 lipidation.

Minor

1. Quantitative data is missing in Fig.1B, Fig.3A, D, and Fig.4 E, F.

-Fig 1B: quantified in panel C.

-Fig 3A: quantification of TECPR1/Gal3 recruitment timing has been added (3C).

-Fig 3D: fluorescence intensity profiles have been added to 3D.

-Fig 4E: fluorescence intensity profiles have been added to 4E. Additional evidence in support of lysosomal LC3 localization has been added by means of Lyso-IP (**Fig 4F**). ATG16L1 KO cells stably

expressing TMEM192-3xHA were treated with LLOMe and lysosomes extracted. Western blot analysis confirmed enrichment of LC3 II on lysosomal membranes after damage.

-Fig 4F (now 4G): quantification has been added

Referee #3:

Corkery et al present a study in which they uncover an unexpected role for the TECPR1-ATG5 axis in driving non-canonical autophagy/CASM to damaged membranes. Authors find that upon lysosome damage with LLOMe, overexpressed TECPR1 is rapidly recruited to lysosomes in a manner dependent on the dysferlin domains. This recruitment precedes larger scale membrane damage and galectin recruitment. Strikingly, they show that damage involves ATG8/LC3 lipidation to lysosomes independent of canonical autophagy. Furthermore, the lipidation is partially independent of ATG16L1 while being completely reliant on ATG5 and ATG7. Authors suggest that the residual LC3 lioidation seen in ATG16L1 KO cells is supported by TECPR1-ATG5 recruitment.
This a well presented study, although it feels less advanced and somewhat less developed, with no exploration as to the function of the lipidation.

Major points

1. Authors should include some controls that their FIP200 and ATG16L1 KO are able to block LC3 lipidation to canonical autophagy induction (ie mTor inhibitor + bafilonmycin).

Re12: The FIP200 and ATG16L1 KO cells used in this study were provided by Prof. Tomatsu Yoshimori (Osaka University). They have been described and extensively validated in a previous study (Nakamura et al., 2020). The knockout of respective proteins was verified in this study (**Fig 4A, C**). The same is true of all other cell lines used, with the exception of TECPR1 KO cells. Appropriate citations are included throughout the text.

2. It is unclear as to the extent that the TECPR1 axis plays in the LLOME LC3 lipdiation response. Can authors deplete TECPR1 in wild type cells and test for the effect on LC3 lipidation?

Re13: This is an excellent point. To address, we have generated TECPR1 KO cells in both wild type and ATG16L1 KO backgrounds (**Fig EV3**). In the absence of ATG16L1 it is clear that TECPR1 is regulating residual LC3 lipidation in response to LMP (**Fig 4H**). In the presence of ATG16L1, the results are less clear. TECPR1 knockout does not result in a significant reduction in LMP-induced LC3 lipidation as we expected. However, loss of TECPR1 has been shown to impair autophagosome-lysosome fusion (Chen *et al.*, 2012), so we can’t exclude the possibility that loss of TECPR1-dependent LC3 lipidation is masked by elevated ATG16L1-dependent LC3 lipidation as a consequence of impaired autophagy flux. To address this problem we will need to determine if the TECPR1-dependent pool of lipidated LC3 differs in any way from the ATG16L1-dependent pool. Only then can we explore its function in the wild type background. This work is ongoing.

As described in Re3 and Re4, we have performed an LLOMe pulse-chase experiment in WT, TECPR1-KO, ATG16L1-KO and TECPR1/ATG16L double-KO cell lines using LysoTracker staining as a readout of lysosomal recovery after damage (**Fig 5**). The results of this experiment would suggest that TECPR1 and ATG16L1 play functionally redundant roles in the response to LMP. This, of course, doesn’t mean

that both E3-like complexes function through the same mechanism and future studies will explore potential structural/mechanistic differences in detail.

3. Authors should acknowledge and comment on previous publications which demonstrated that LLOMe induced LC3 lipidation was blocked by ATG16L1 KO, with no residual lipidation remaining (Nakamura et al. PMID: 32989250). Furthermore, blocking of the ATG16L1-v-ATPase axis by SopF was previously seen to inhibit LLOMe induced lipidation (Xu et al. PMID: 35046574). What are the differences between these studies.

Re14: Closer examination of western blot data for ATG16L1KO cells treated with LLOMe from previous publications does in fact show a residual amount of lipidated LC3 (eg. Nakamura *et al.* Extended Data 2A). While the amount of lipidated LC3 is minor as compared to adjacent lanes (FIP200KO/ATG14KO), it is clearly present. Thus, it seems as though LMP-induced ATG16L1-independent ATG8 lipidation has been observed in the literature for some time, just overlooked due to its low abundance.

Similarly, blocking of the ATG16L1-v-ATPase axis by SopF does significantly reduce LLOMe-induced LC3 lipidation in wild type cells, but residual lipidation remains. Not surprisingly, we have found TECPR1-dependent LC3 lipidation to be independent of v-ATPase function (Bafilomycin a1-insensitive) suggesting residual LC3 lipidation in SopF expressing cells could be dependent on this alternative E3-like complex.

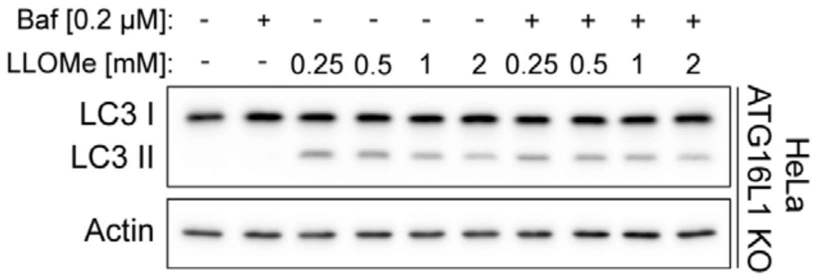

Minor point
1. It is unclear as to what antibodies were used to for western blot against LC3A, GABARRAP and GABARAPL1 in Fig EV2. These are not mentioned in the materials and methods.

Re: Missing antibody information has been added.

Manuscript number: EMBOR-2023-56841V2
Title: ATG12-ATG5-TECPR1 E3-like complex regulates unconventional LC3 lipidation at damaged lysosomes
Author(s): Dale Corkery, Sergio Castro-Gonzalez, Anastasia Knyazeva, Laura Herzog, and Yaowen Wu

Dear Yaowen,

Thank you for your patience while we have reviewed your revised manuscript. As you will see from the reports below, the referees are now all positive about its publication in EMBO reports. I am therefore writing with an 'accept in principle' decision, which means that I will be happy to accept your manuscript for publication once a few minor issues/corrections have been addressed, as follows.

1) Please remove the Author Contributions from the manuscript and make sure that the author contributions in our online submission system are correct and up-to-date. The information you specified in the system will be automatically retrieved and typeset into the article.

2) Please add callouts to Fig EV3A&B panels.

3) Please sort the figure legends into main figure legends first (header Figure legends) and EV figure legends in a second paragraph (header: 'Expanded View Figure Legends').

4) I attach to this email a related manuscript file with comments by our data editors. Please address all comments and upload a revised file with tracked changes with your final manuscript submission.

5) Finally, EMBO reports papers are accompanied online by A) a short (1-2 sentences) summary of the findings and their significance, B) 2-3 bullet points highlighting key results and C) a synopsis image that is 550x300-600 pixels large (width x height) in PNG for JPG format. You can either show a model or key data in the synopsis image. Please note that the size is rather small and that text needs to be readable at the final size. Please send us this information along with the revised manuscript.

Once you have made these minor revisions, please use the following link to submit your corrected manuscript:

Link Not Available

If all remaining corrections have been attended to, you will then receive an official decision letter from the journal accepting your manuscript for publication in the next available issue of EMBO reports. This letter will also include details of the further steps you need to take for the prompt inclusion of your manuscript in our next available issue.

Thank you for your contribution to EMBO reports.

Kind regards,

Martina

Martina Rembold, PhD
Senior Editor
EMBO reports

****

Referee #1:

The authors responded to all of my concerns I raised for the original manuscript in a satisfactory manner.

Referee #2:

The authors performed many new experiments to reveal the functional contributions of TECPR1 and ATG16L1 to lysosomal damage repair. Notably, the new knockout cell lines and new pulse chase experiments significantly strengthened the conclusion of the authors.
I therefore recommend publication of this study in EMBO reports.

Referee #3:

The authors have satisfactorily addressed my main concerns with the manuscript. Furthermore, authors have begun to address functional consequences of unconventional LC3 lipidation mediated by TECPR1. I feel that this study deserves to be published in EMBO Reports.

The authors have addressed all minor editorial requests.

Prof. Yaowen Wu
Umeå University
Departmend of Chemistry, Umeå Centre for Microbial Research
Departmend of Chemistry
Umeå 90187
Sweden

Dear Yaowen,

I am very pleased to accept your manuscript for publication in the next available issue of EMBO reports. Thank you for your contribution to our journal.

At the end of this email I include important information about how to proceed. Please ensure that you take the time to read the information and complete and return the necessary forms to allow us to publish your manuscript as quickly as possible.

As part of the EMBO publication's Transparent Editorial Process, EMBO reports publishes online a Review Process File to accompany accepted manuscripts. As you are aware, this File will be published in conjunction with your paper and will include the referee reports, your point-by-point response and all pertinent correspondence relating to the manuscript.

If you do NOT want this File to be published, please inform the editorial office within 2 days, if you have not done so already, otherwise the File will be published by default [contact: emboreports@embo.org]. If you do opt out, the Review Process File link will point to the following statement: "No Review Process File is available with this article, as the authors have chosen not to make the review process public in this case."

Should you be planning a Press Release on your article, please get in contact with emboreports@wiley.com as early as possible, in order to coordinate publication and release dates.

Thank you again for your contribution to EMBO reports and congratulations on a successful publication. Please consider us again in the future for your most exciting work.

Kind regards,

Martina

Martina Rembold, PhD
Senior Editor
EMBO reports

****

THINGS TO DO NOW:

Please note that you will be contacted by Wiley Author Services to complete licensing and payment information. The required 'Page Charges Authorization Form' is available here: https://www.embopress.org/pb-assets/embo-site/er_apc.pdf - please download and complete the form and return to embopressproduction@wiley.com

EMBO Press participates in many Publish and Read agreements that allow authors to publish Open Access with reduced/no publication charges. Check your eligibility: https://authorservices.wiley.com/author-resources/Journal-Authors/open-access/affiliation-policies-payments/index.html

You will receive proofs by e-mail approximately 2-3 weeks after all relevant files have been sent to our Production Office; you should return your corrections within 2 days of receiving the proofs.

Please inform us if there is likely to be any difficulty in reaching you at the above address at that time. Failure to meet our deadlines may result in a delay of publication, or publication without your corrections.

All further communications concerning your paper should quote reference number EMBOR-2023-56841V3 and be addressed to emboreports@wiley.com.

Should you be planning a Press Release on your article, please get in contact with emboreports@wiley.com as early as possible, in order to coordinate publication and release dates.

EMBO Press Author Checklist

Corresponding Author Name: Yao-wen Wu	
Journal Submitted to: EMBO Reports	
Manuscript Number: EMBOR-2023-56841	

USEFUL LINKS FOR COMPLETING THIS FORM
The EMBO Journal - Author Guidelines
EMBO Reports - Author Guidelines
Molecular Systems Biology - Author Guidelines
EMBO Molecular Medicine - Author Guidelines

Reporting Checklist for Life Science Articles (updated January 2022)
This checklist is adapted from Materials Design Analysis Reporting (MDAR) Checklist for Authors. MDAR establishes a minimum set of requirements in transparent reporting in the life sciences (see Statement of Task: 10.31222/osf.io/9sm4x). Please follow the journal's guidelines in preparing your manuscript.
Please note that a copy of this checklist will be published alongside your article.

Abridged guidelines for figures
1. Data
The data shown in figures should satisfy the following conditions:
- the data were obtained and processed according to the field's best practice and are presented to reflect the results of the experiments in an accurate and unbiased manner.
- ideally, figure panels should include only measurements that are directly comparable to each other and obtained with the same assay.
- plots include clearly labeled error bars for independent experiments and sample sizes. Unless justified, error bars should not be shown for technical replicates.
- if n<5, the individual data points from each experiment should be plotted. Any statistical test employed should be justified.
- Source Data should be included to report the data underlying figures according to the guidelines set out in the authorship guidelines on Data Presentation.

2. Captions
Each figure caption should contain the following information, for each panel where they are relevant:
- a specification of the experimental system investigated (eg cell line, species name).
- the assay(s) and method(s) used to carry out the reported observations and measurements.
- an explicit mention of the biological and chemical entity(ies) that are being measured.
- an explicit mention of the biological and chemical entity(ies) that are altered/varied/perturbed in a controlled manner.
- the exact sample size (n) for each experimental group/condition, given as a number, not a range;
- a description of the sample collection allowing the reader to understand whether the samples represent technical or biological replicates (including how many animals, litters, cultures, etc.).
- a statement of how many times the experiment shown was independently replicated in the laboratory.
- definitions of statistical methods and measures:
  - common tests, such as t-test (please specify whether paired vs. unpaired), simple χ^2 tests, Wilcoxon and Mann-Whitney tests, can be unambiguously identified by name only, but more complex techniques should be described in the methods section;
  - are tests one-sided or two-sided?
  - are there adjustments for multiple comparisons?
  - exact statistical test results, e.g., P values = x but not P values < x;
  - definition of 'center values' as median or average;
  - definition of error bars as s.d. or s.e.m.

Please complete ALL of the questions below.
Select "Not Applicable" only when the requested information is not relevant for your study.

Materials

Newly Created Materials	Information included in the manuscript?	In which section is the information available? (Reagents and Tools Table, Materials and Methods, Figures, Data Availability Section)
New materials and reagents need to be available; do any restrictions apply?	Not Applicable	

Antibodies	Information included in the manuscript?	In which section is the information available? (Reagents and Tools Table, Materials and Methods, Figures, Data Availability Section)
For **antibodies** provide the following information:<br>- Commercial antibodies: RRID (if possible) or supplier name, catalogue number and or/clone number<br>- Non-commercial: RRID or citation	Yes	Materials and Methods

DNA and RNA sequences	Information included in the manuscript?	In which section is the information available? (Reagents and Tools Table, Materials and Methods, Figures, Data Availability Section)
**Short novel DNA or RNA including primers, probes**: provide the sequences.	Yes	Materials and Methods

Cell materials	Information included in the manuscript?	In which section is the information available? (Reagents and Tools Table, Materials and Methods, Figures, Data Availability Section)
**Cell lines:** Provide species information, strain. Provide accession number in repository **OR** supplier name, catalog number, clone number, and/**OR** RRID.	Yes	Materials and Methods
**Primary cultures:** Provide species, strain, sex of origin, genetic modification status.	Not Applicable	
Report if the cell lines were recently **authenticated** (e.g., by STR profiling) and tested for mycoplasma contamination.	Yes	Materials and Methods

Experimental animals	Information included in the manuscript?	In which section is the information available? (Reagents and Tools Table, Materials and Methods, Figures, Data Availability Section)
**Laboratory animals or Model organisms:** Provide species, strain, sex, age, genetic modification status. Provide accession number in repository **OR** supplier name, catalog number, clone number, **OR** RRID.	Not Applicable	
**Animal observed in or captured from the field:** Provide species, sex, and age where possible.	Not Applicable	
Please detail **housing and husbandry conditions**.	Not Applicable	

Plants and microbes	Information included in the manuscript?	In which section is the information available? (Reagents and Tools Table, Materials and Methods, Figures, Data Availability Section)
**Plants:** provide species and strain, ecotype and cultivar where relevant, unique accession number if available, and source (including location for collected wild specimens).	Not Applicable	
**Microbes:** provide species and strain, unique accession number if available, and source.	Not Applicable	

Human research participants	Information included in the manuscript?	In which section is the information available? (Reagents and Tools Table, Materials and Methods, Figures, Data Availability Section)
If collected and within the bounds of privacy constraints report on age, sex and gender or ethnicity for all study participants.	Not Applicable	

Core facilities	Information included in the manuscript?	In which section is the information available? (Reagents and Tools Table, Materials and Methods, Figures, Data Availability Section)
If your work benefited from core facilities, was their service mentioned in the acknowledgments section?	Yes	Acknowledgements

Design

Study protocol	Information included in the manuscript?	In which section is the information available? (Reagents and Tools Table, Materials and Methods, Figures, Data Availability Section)
If study protocol has been **pre-registered, provide DOI in the manuscript**. For clinical trials, provide the trial registration number **OR** cite DOI.	Not Applicable	
Report the **clinical trial registration number** (at ClinicalTrials.gov or equivalent), where applicable.	Not Applicable	

Laboratory protocol	Information included in the manuscript?	In which section is the information available? (Reagents and Tools Table, Materials and Methods, Figures, Data Availability Section)
Provide DOI OR other citation details if **external detailed step-by-step protocols** are available.	Not Applicable	

Experimental study design and statistics	Information included in the manuscript?	In which section is the information available? (Reagents and Tools Table, Materials and Methods, Figures, Data Availability Section)
Include a statement about **sample size** estimate even if no statistical methods were used.	Yes	Figure Legends
Were any steps taken to minimize the effects of subjective bias when allocating animals/samples to treatment (e.g. **randomization procedure**)? If yes, have they been described?	Not Applicable	
Include a statement about **blinding** even if no blinding was done.	Yes	Materials and Methods
Describe **inclusion/exclusion criteria** if samples or animals were excluded from the analysis. Were the criteria pre-established?<br><br>If sample or data points were omitted from analysis, report if this was due to attrition or intentional exclusion and provide justification.	Not Applicable	
For every figure, are **statistical tests** justified as appropriate? Do the data meet the assumptions of the tests (e.g., normal distribution)? Describe any methods used to assess it. Is there an estimate of variation within each group of data? Is the variance similar between the groups that are being statistically compared?	Yes	Materials and Methods/Figure Legends

Sample definition and in-laboratory replication	Information included in the manuscript?	In which section is the information available? (Reagents and Tools Table, Materials and Methods, Figures, Data Availability Section)
In the figure legends: state number of times the experiment was **replicated** in laboratory.	Yes	Figure Legends
In the figure legends: define whether data describe **technical or biological replicates**.	Yes	Figure Legends

Ethics

Ethics	Information included in the manuscript?	In which section is the information available? (Reagents and Tools Table, Materials and Methods, Figures, Data Availability Section)
Studies involving **human participants**: State details of **authority granting ethics approval** (IRB or equivalent committee(s), provide reference number for approval.	Not Applicable	
Studies involving **human participants**: Include a statement confirming that **informed consent** was obtained from all subjects and that the experiments conformed to the principles set out in the WMA Declaration of Helsinki and the Department of Health and Human Services Belmont Report.	Not Applicable	
Studies involving **human participants:** For publication of **patient photos**, include a statement confirming that consent to publish was obtained.	Not Applicable	
Studies involving experimental **animals**: State details of **authority granting ethics approva**l (IRB or equivalent committee(s), provide reference number for approval. Include a statement of compliance with ethical regulations.	Not Applicable	
Studies involving **specimen and field samples:** State if relevant **permits** obtained, provide details of authority approving study; if none were required, explain why.	Not Applicable	

Dual Use Research of Concern (DURC)	Information included in the manuscript?	In which section is the information available? (Reagents and Tools Table, Materials and Methods, Figures, Data Availability Section)
Could your study fall under dual use research restrictions? Please check biosecurity documents and list of **select agents and toxins** (CDC): https://www.selectagents.gov/sat/list.htm	Not Applicable	
If you used a select agent, is the security level of the lab appropriate and reported in the manuscript?	Not Applicable	
If a study is subject to dual use research of concern regulations, is the name of the **authority granting approval and reference number** for the regulatory approval provided in the manuscript?	Not Applicable	

Reporting

The MDAR framework recommends adoption of discipline-specific guidelines, established and endorsed through community initiatives. Journals have their own policy about requiring specific guidelines and recommendations to complement MDAR.

Adherence to community standards	Information included in the manuscript?	In which section is the information available? (Reagents and Tools Table, Materials and Methods, Figures, Data Availability Section)
State if relevant guidelines or checklists (e.g., **ICMJE, MIBBI, ARRIVE, PRISMA**) have been followed or provided.	Not Applicable	
For **tumor marker prognostic studies,** we recommend that you follow the **REMARK** reporting guidelines (see link list at top right). See author guidelines, under 'Reporting Guidelines'. Please confirm you have followed these guidelines.	Not Applicable	
For **phase II and III randomized controlled trials**, please refer to the **CONSORT** flow diagram (see link list at top right) and submit the CONSORT checklist (see link list at top right) with your submission. See author guidelines, under 'Reporting Guidelines'. Please confirm you have submitted this list.	Not Applicable	

Data Availability

Data availability	Information included in the manuscript?	In which section is the information available? (Reagents and Tools Table, Materials and Methods, Figures, Data Availability Section)
Have **primary datasets** been deposited according to the journal's guidelines (see 'Data Deposition' section) and the respective accession numbers provided in the Data Availability Section?	Not Applicable	
Were **human clinical and genomic datasets** deposited in a public access-controlled repository in accordance to ethical obligations to the patients and to the applicable consent agreement?	Not Applicable	
Are **computational models** that are central and integral to a study available without restrictions in a machine-readable form? Were the relevant accession numbers or links provided?	Not Applicable	
If publicly available data were reused, provide the respective **data citations in the reference list**.	Not Applicable	
